# Synthesis of *N*‑Substituted Acenaphtho[1,2‑*b*]pyrroles and Dibenzo[*e,g*]indoles with Promising Antileukemic Activity from Morita–Baylis–Hillman Adducts

**DOI:** 10.1021/acsomega.5c11609

**Published:** 2026-03-06

**Authors:** João Arantes, Manoel T. Rodrigues, Giovani Rosendo, Rafael Porreca, Hugo P. Vicari, Hugo Santos, João A. Machado-Neto, Fernando Coelho

**Affiliations:** † Department of Organic Chemistry, Chemistry Institute, 28132 University of Campinas, Campinas, SP 13083-970, Brazil; ‡ Department of Pharmacology, Institute de Biomedical Sciences, 28133 University of São Paulo, São Paulo, SP 05508-900, Brazil

## Abstract

This study investigates a novel synthetic pathway for *N*-substituted polycyclic heterocycles with potential antileukemic activity. The strategy employs Morita–Baylis–Hillman (MBH) adducts derived from polycyclic 1,2-diketones such as acenaphthoquinone and phenanthrene-9,10-dione. The key steps involve acetylation of the MBH adducts followed by cyclization with primary amines to afford *N*-heterocycles, specifically acenaphtho­[1,2-*b*]­pyrroles and dibenzo­[*e,g*]­indoles. The synthesized compounds were evaluated in vitro against leukemia cell lines (Jurkat and NB4). Several derivatives exhibited promising activity, with compound 4b showing particularly strong potency against the NB4 cell line. Overall, this work advances the development of novel antileukemic agents and underscores the potential of *N*-substituted polycyclic heterocycles in leukemia therapy.

## Graphical abstract

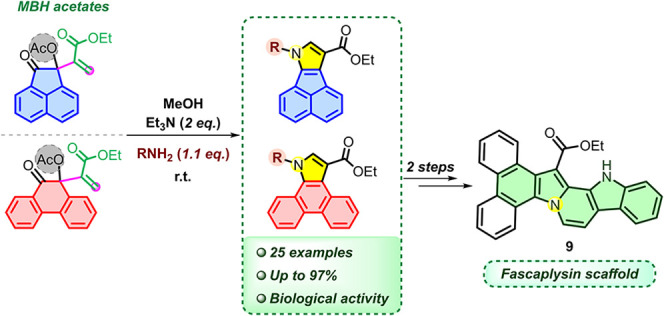

## Introduction

*N-*Heterocyclic polycycles are widely distributed in nature, with indoles and pyrroles standing out as prominent examples found in numerous natural products.^1^ Additionally, some *N*-heterocyclic polycycles exhibit biological activity^2–4^ as well as electrical and photoluminescent properties.^5–9^ For this reason, the synthesis of these compounds has been explored in recent years.^10–16^

The compounds shown in Figure 1 illustrate the core structures of the polycycles acenaphthylene (**1**) and phenanthrene (**2**), both of which are commonly encountered in a wide range of natural products.^17–19^ These moieties are highly versatile in terms of functionality, as they can exhibit anticancer activity (Figure 1), as well as diverse photoelectronic properties.^20–22^

**Figure 1:**
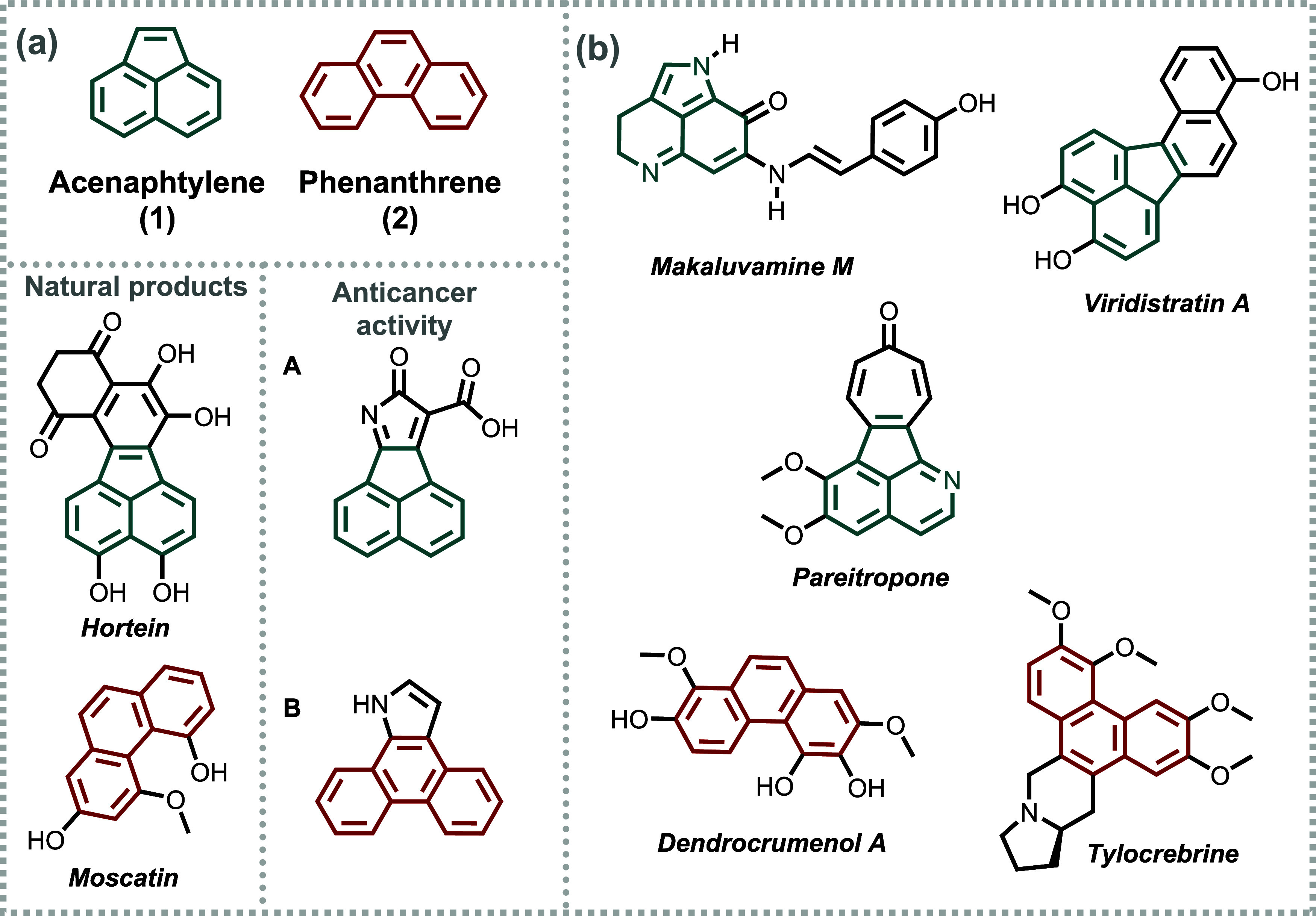
(a) Acenaphtylene (**1**) and phenanthrene (**2**) cores and their occurrences in natural products and molecules with anticancer activity. (b) Other natural products containing structurally similar moieties. (A) Reprinted (Adapted in part) with permission from [Xie, L.; Xiao, Y.; Wang, F.; Xu, Y.; Qian, X.; Zhang, R.; Cui, J.; Liu, J. *Bioorg. Med. Chem. 17*, 7615–7521]. Copyright [**2009**]­[Elsevier]. (B) Reprinted (Adapted in part) with permission from [Jones, G. B; Mathews, E. *J. Tetrahedron 53*, 14599–14614]­[**1997**]­[Elsevier].

1,2-Diketones can be interesting building blocks for the formation of a wide variety of heterocycles.^23,24^ Polycyclic 1,2-diketones have also been explored as substrates to form new heterocyclic arrangements, among which 1,2-acenaphthylenequinone and 9,10-phenanthrenedione stand out.^25,26^ In recent years, it has been shown that *N*-heterocyclic derivatives of these polycyclic structures can exhibit interesting properties, increasing their potential applications in materials and medicinal areas.^27–29^ This motivated us to seek pathways to obtain new heterocyclic arrangements involving these two structural patterns mentioned above.

Some methods that achieve *N*-heterocycles containing the structural pattern of acenaphthene and phenanthrene are shown below (Scheme 1). Mathews et al. used azides to obtain the polycyclic indole in xylene, under reflux, allowing thermolysis via nitrene insertion, yielding only one analogous product. Nicolaides et al.^30^ only mentioned the formation of a side product using phosphorus ylides with low yield. Azizian et al. developed a methodology that enables access to a series of highly substituted pyrroles using triphenylphosphine, ammonium acetate, and butynedioates at room temperature.^31^

**Scheme 1:**
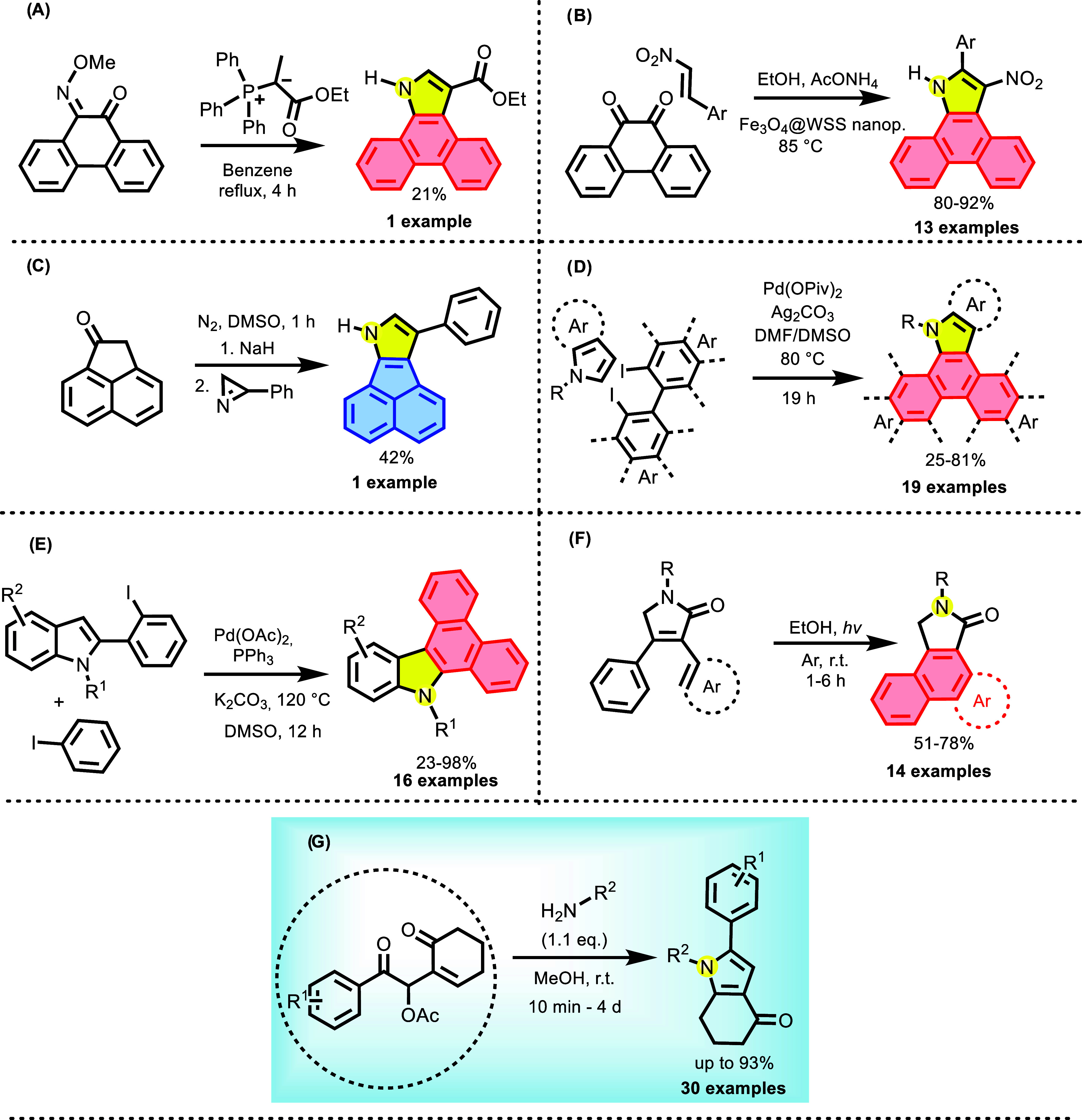
Methodologies for the Synthesis of Pyrrole-Containing *N*-Heterocyclic Polycyclic Derivatives^a^

a (A) Reprinted (Adapted in part) with permission from ref 30. Copyright [**2001**] [Elsevier]. (B) Reprinted (Adapted in part) with permission from [Ahmadian, M.; Rad-Moghadam, K.; Dehghanian, A.; Jafari, M. *New. J. Chem*. 46, 2940-2951]. Copyright [**2022**] [Royal Sociey of Chemistry]. (C) Reprinted (Adapted in part) with permission from ref 32 b­[**2018**] [Elsevier]. (D) Reprinted (Adapted in part) with permission from ref 34 [Royal Society of Chemistry]. (E) Reprinted (Adapted in part) with permission from ref 35 [Royal Society of Chemistry]. (F) Reprinted (Adapted in part) with permission from ref 36 [Elsevier]. (G) Reprinted (Adapted in part) with permission from ref 37 [John Wiley & Sons].

In 2018, Jiang et al. used the methodology described by Zhao and Carreira to develop new dyes, derived from acenaphthene, which are polycyclic fluorescent compounds of the aza-BODIPY class.^32a,33^ The methodology involves the synthesis of a pyrrole via cyclization induced by the formation of a carbanion between a ketone and a substituted azirine at the second position; however, only one example was explored with the acenaphthylene core. In 2022, Ahmadian et al. developed the synthesis of substituted dibenzo­[*e,g*]­indoles, using Fe_3_O_4_-containing nanoparticles in ethanol at 85 °C (when other polycyclic ketones were tested, each led to the formation of a complex mixture).^32b^

However, none of these methodologies allow for the direct synthesis of *N*-substituted heterocycles, since all sources of nitrogen lead to the *N–H* product, which would require an additional step involving amine alkylation. In 2017, Kitano et al. synthesized *N*-substituted polycyclic carbazoles using palladium acetate in a basic medium under heating.^34^ Wu et al. obtained *N*-substituted dibenzo­[*e,g*]­indoles with free positions at 2 and 3, but with yields ranging from low to medium, also using palladium.^35^ Kang et al. employed photolytic conditions in an inert atmosphere to fuse their polycyclic systems.^36^ Finally, Batchu and Batra synthesized *N*-substituted pyrroles from Morita–Baylis–Hillman (MBH) acetates; these products were substituted at position 2 and unsubstituted at position 3.^37^

Polycyclic and aliphatic diketones have been explored as potential electrophiles in MBH reactions. In 2010, Basavaiah et al. developed a methodology using titanium chloride, allowing the synthesis of adducts from various polycyclic diketones and cyclopentenones (Scheme 2).^38^ In 2012, Khalafi-Nezhad and Mohammadi used ionic liquids as catalysts for the same type of electrophile, but employing acrylates, achieving excellent yields and reaction times. However, recovery of the ionic liquid requires evaporation from an aqueous phase, a process that takes approximately 5 h, according to the authors.^39^

**Scheme 2:**
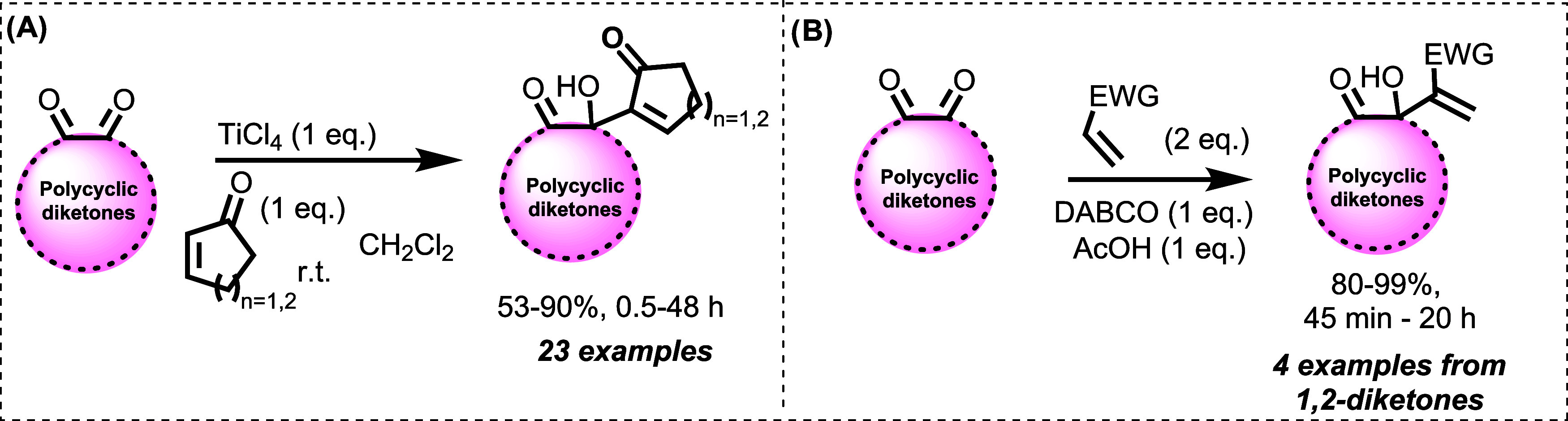
MBH Reactions and Different Methodologies Involving Polycyclic Diketones^a^

a (A) Reprinted (Adapted in part) with permission from ref 38. Copyright [**2010**] [Elsevier]. A. Reprinted (Adapted in part) with permission from ref 40. Copyright [**2022**]­[John Wiley & Sons].

Since our group aims to synthesize new, relevant heterocycles, these diketones could serve as interesting building blocks for the formation of polycyclic molecules. Additionally, MBH adducts could effectively play their roles as potential synthetic intermediates to achieve more complex molecules.

Recently, our research group described a methodology that encompasses various types of electrophiles in MBH reactions, achieving excellent yields and good reaction times (Scheme 2).^40,41^ However, examples of polycyclic dicarbonyl compounds were limited to 1,2-acenaphthylenedione and isatins, and their synthetic applications were not explored in this work. The 1,4-system of MBH adducts, linked to the versatility of conjugate addition of amines, is a well-established and widely explored chemistry, but still holds significant synthetic potential.^42–47^

Expanding on the methodology reported by Batchu et al.^37^ we present a synthetic strategy for polycyclic *N*-heterocycles, including acenaphtho­[1,2-*b*]­pyrroles and dibenzo­[*e,g*]­indoles.

## Results and Discussion

This study investigated MBH reactions using two different 1,2-dicarbonyl analogues. We began by exploring 1,2-diketones as electrophiles. Specifically, acenaphthoquinone (**1a**) was subjected to an MBH reaction with ethyl acrylate, DABCO, and acetic acid (AcOH), following the methodology developed by our laboratory (**General Procedure A**). The reaction was monitored by TLC, and complete consumption of the starting material was observed after 5 h under stirring. Notably, the only liquid components used in this procedure are AcOH and the acrylate. After extraction and purification via column chromatography, the MBH adduct (**2a**) was isolated with an 82% yield (Scheme 3). Subsequently, phenanthrene-9,10-dione (**1b**) was also tested under the same conditions. However, its consumption was slower compared to **1a**, likely due to its low solubility in the reaction medium. The reaction was allowed to proceed for 5 days, resulting in the formation of adduct **2b** with a 75% yield. Attempts to improve solubility by adding various solvents (methanol, acetonitrile, dichloromethane, and chloroform) were unsuccessful in optimizing the yield. Increasing the amount of ethyl acrylate to a large excess (20 equiv) significantly improved the reaction time and yield, with the reaction completing in 3 days and a yield of 95%, still using AcOH and DABCO. Adduct **2c** was also synthesized, albeit with a lower yield even with extended reaction time.

**Scheme 3:**
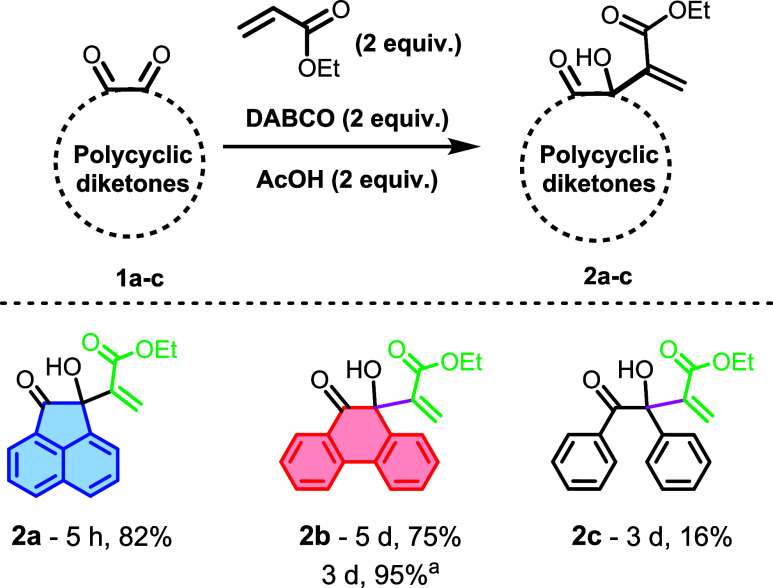
MBH Reactions between Different Polycyclic Diketones and Acrylates^a^

a Yield using large excess (20 equiv) of ethyl acrylate.

Adducts **2a** and **2b** were subjected to acetylation according to **General Procedure B**, producing the MBH acetates **3a** and **3b** (Scheme 4) with yields of 86% and 60%, respectively.

**Scheme 4:**
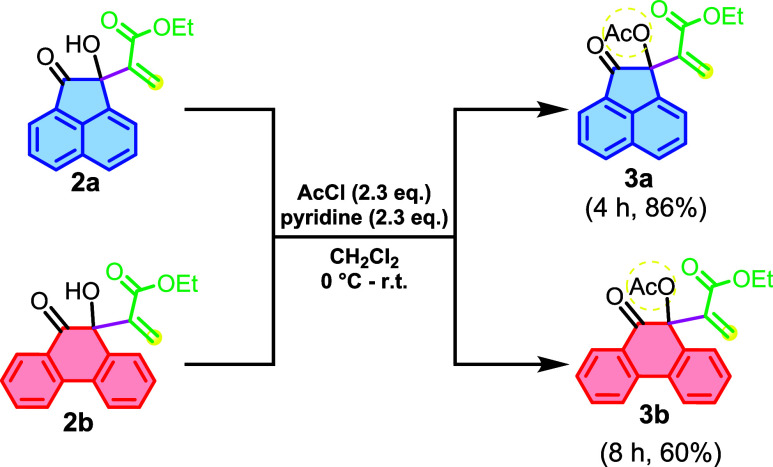
Acetylation Reaction of Polycyclic MBH Adducts

Our first attempt at forming the *N*-heterocycle using the reported methodology was successful.^37^ Starting from a methanolic solution of the MBH acetate **3a** in the presence of triethylamine and the corresponding primary amine, it was possible to access a highly substituted, polycyclic pyrrole with *N*-substitution in just one reaction step under mild conditions. A subsequent test demonstrated that, in the presence of a base, complete consumption of the starting material is observed, while in its absence, the reaction remains incomplete even after 12 h. The methodology developed by Batchu and Batra^37^ does not specify the need for an excess of base, using only 1.1 equiv of the corresponding primary amine.

In a single step, two new *N–C* σ bonds are formed, leading to the cyclization and aromatization of the pyrrole. This motivated us to test a series of primary amines combined with different MBH acetates. Thus, compounds **3a** and **3b** were subjected to cyclization reactions according to **General Procedure C**, forming, respectively, acenaphtho­[1,2-*b*]­pyrroles (**4a-n**) shown in Scheme 5 and dibenzo­[*e,g*]­indoles **(5a-o)**, shown in Scheme 6.

**Scheme 5:**
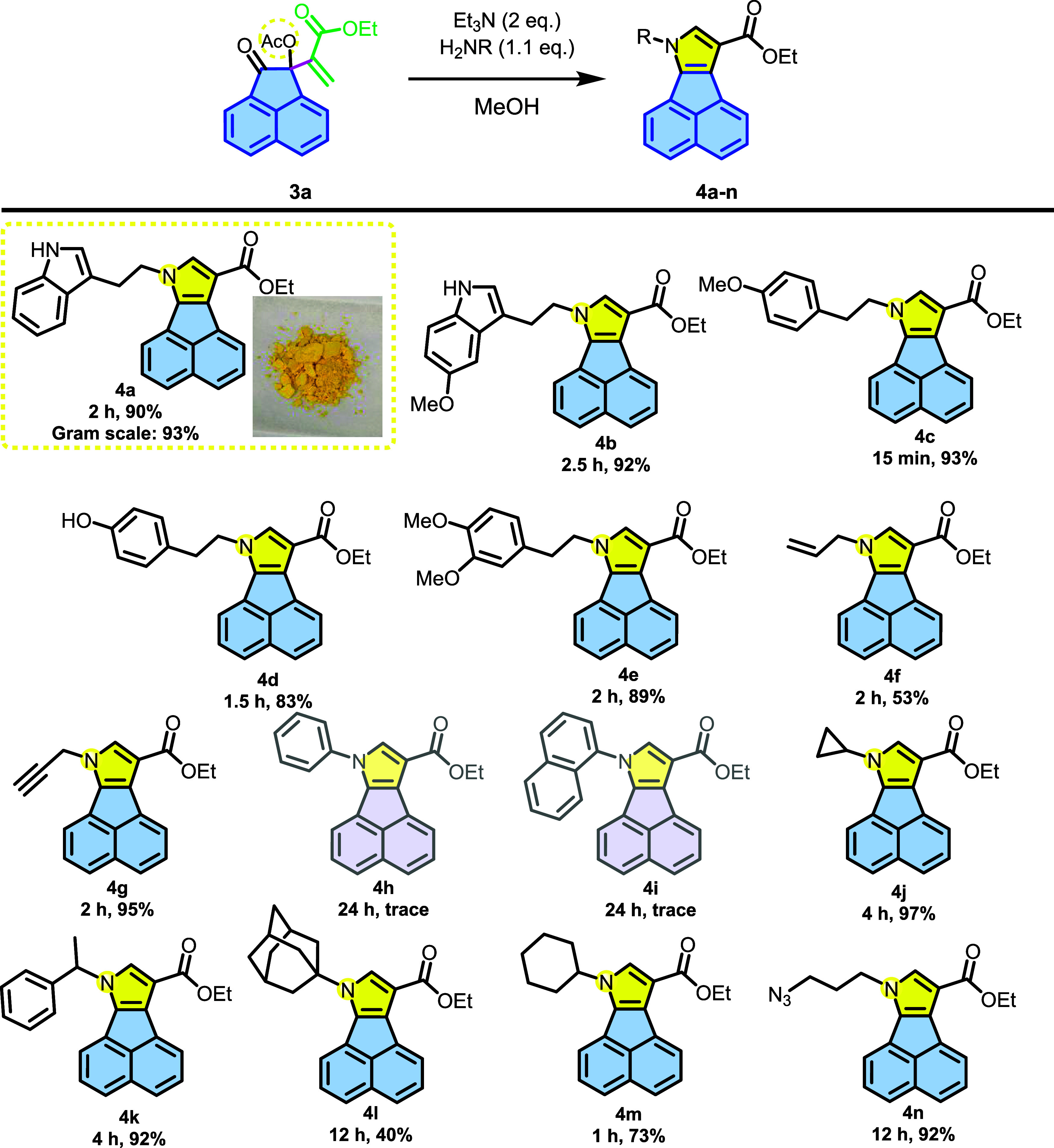
Scope of *N*-Substituted Acenaphtho­[1,2-*b*]­pyrroles^a^

a Traces of product **4i** detected by high-resolution mass spectrometry analysis of an aliquot of the crude reaction extract.

**Scheme 6:**
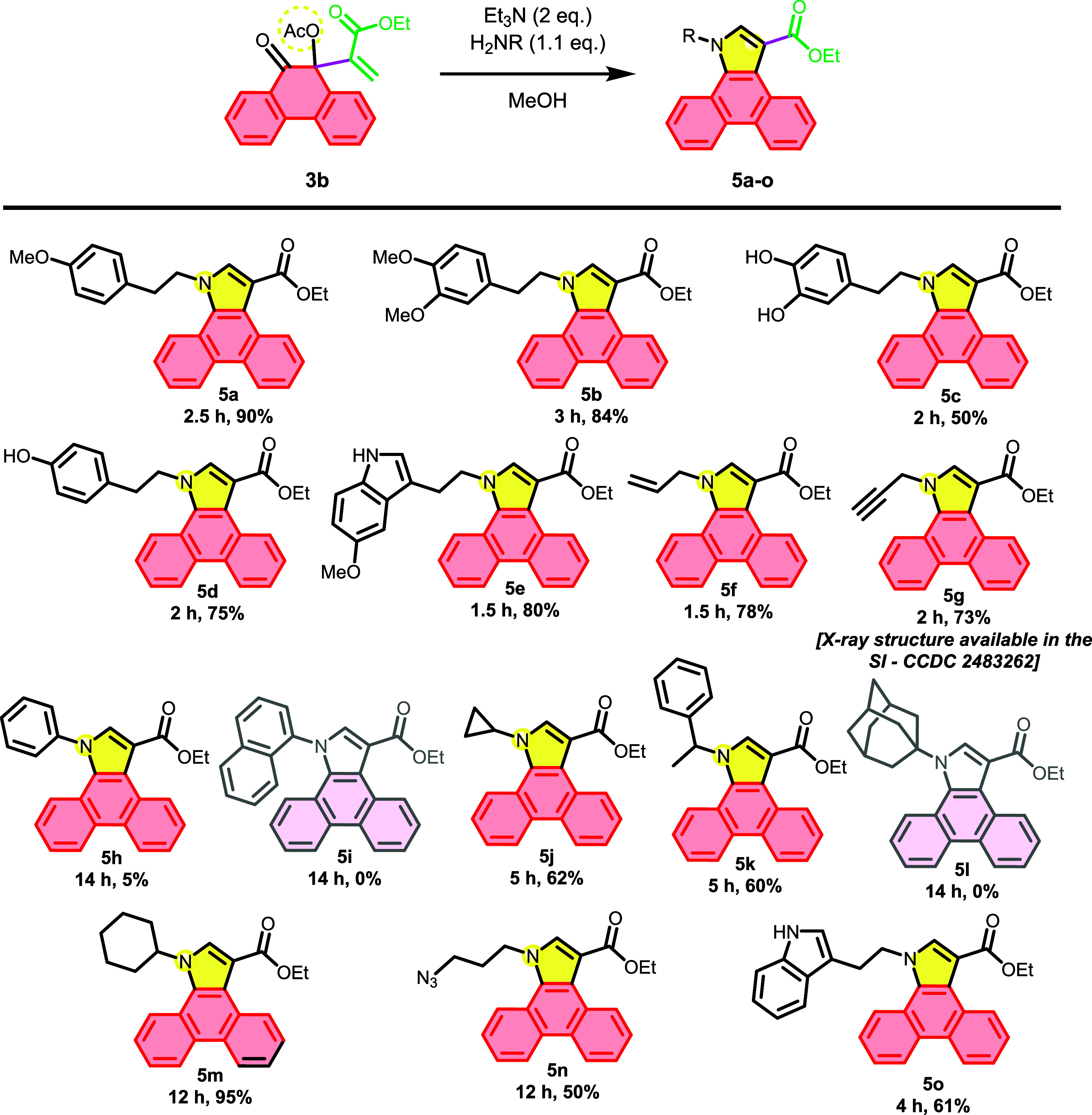
Scope of *N*-Substituted Dibenzo­[*e,g*]­indoles

In the case of MBH acetate **3a**, the cyclization reaction generally proceeded slightly faster than with adduct **3b**. However, it was not possible to isolate the derivative **4h** due to the formation of a complex mixture. Conversely, derivative **5h** was isolated, albeit in a very low yield (5%), likely due to reduced nucleophilicity of the nitrogen in aniline derivatives owing to resonance stabilization of its nonbonding electrons. The analogs **4i** and **5i** were not isolated, as no new product formation was observed in the TLC; however, traces of **4i** were detected by high-resolution mass spectrometry, while the formation of **5i** could not be confirmed. The analog **4l** was obtained with a 40% yield after a relatively long reaction time compared to other analogs. In contrast, compound **5l** was neither observed in the TLC nor detected by mass spectrometry. The absence of **5l** is attributed to steric hindrance caused by the adamantyl group. It is likely that the initial step of the cyclization involves a 1,4-addition of the primary amine to the Michael acceptor, a process that may be impeded by electronic repulsion between the bulky groups on both the amine and the phenanthrene.

A mechanistic proposal (Scheme 7) involves multiple steps, including conjugate addition and acetate elimination,^48^ followed by intramolecular cyclization on the carbonyl moiety and aromatization. We observed that the presence of a base in the reaction medium promotes the reaction’s completeness, whereas in its absence, even after 12 h, the starting material is not fully consumed. Based on these observations, we devised two possible explanations. The first hypothesis suggests that the role of the base is related to kinetically favoring a series of proton exchange steps. The primary amine itself could assist in these steps, but over time, it will be consumed, leading to a decrease in its concentration in the medium. Therefore, an excess of base ensures that species are deprotonated, facilitating the formation of the final product. The second hypothesis considers trietylamine (TEA) acting as a catalyst in the reaction. In this case, TEA would participate in the conjugate addition (*aza*-Michael) due to its higher concentration than that of the primary amines used. After TEA carries out the 1,4-addition, acetate elimination occurs. In the subsequent step, TEA would act as a leaving group, while the primary amine functions as a nucleophile in a substitution reaction. Further proton exchange, E1cB elimination, intramolecular formation of an iminium ion and aromatization lead to the observed product.

**Scheme 7:**
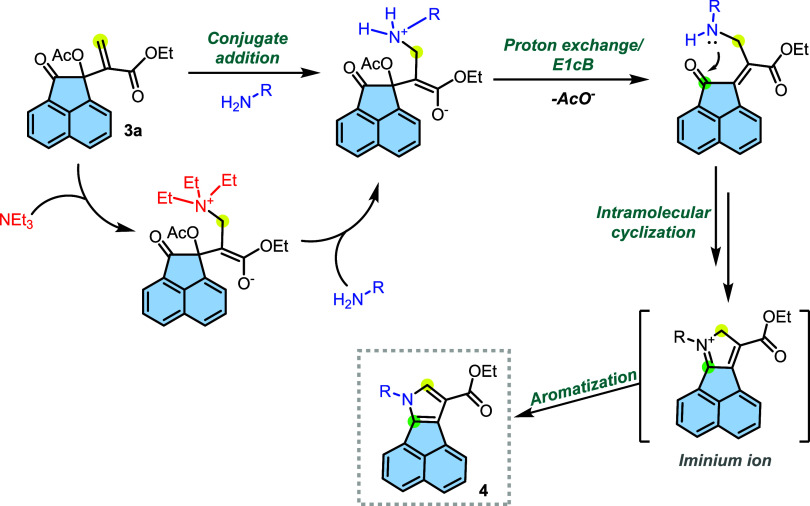
Mechanistic Proposal for the Formation of **4** from MBH Acetate **3a**

With this panel of synthesized indole and pyrrole derivatives, we decided to explore additional derivatizations. As the first challenge, we investigated click reactions involving the cycloaddition of the azide–alkyne pair, catalyzed by copper salts. These reactions, which readily lead to the formation of a heterocycle (triazole) with significant potential for applications in photophysical and pharmaceutical properties, motivated us to perform the first derivatization (**General Procedure D**). Copper-catalyzed functionalization of the terminal triple bonds of molecules **4g** and **5g** lead to products **6a** and **6b** in 12 h and good yields (Scheme 8). To assess the reactivity of the “azide–alkyne” pairs at the terminal position, the substitution pattern was reversed. For this purpose, polycyclic analogs containing terminal azides (**4n** and **5n**) were coupled with phenylacetylene, furnishing products **7a** and **7b** also in good yields (Scheme 9). It is worth noting that these “click” reactions produced few to no byproducts, according to TLC analyses, and required similar reaction times for both the synthesis of triazoles **6a**–**b** and **7a**–**b**.

**Scheme 8:**
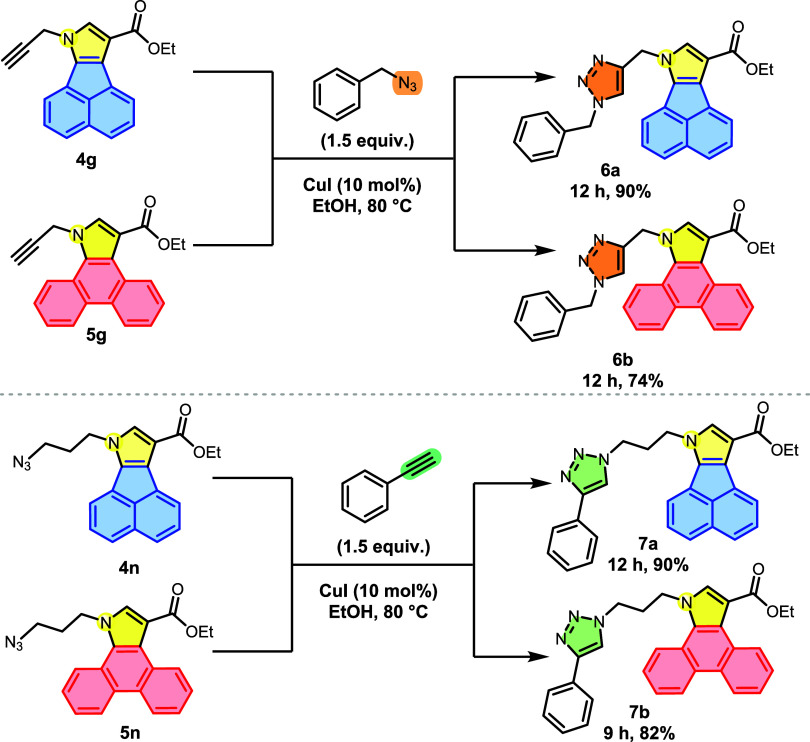
Derivatization of Polycyclic Heterocycles Bearing Terminal Azides via Click Reactions

**Scheme 9:**
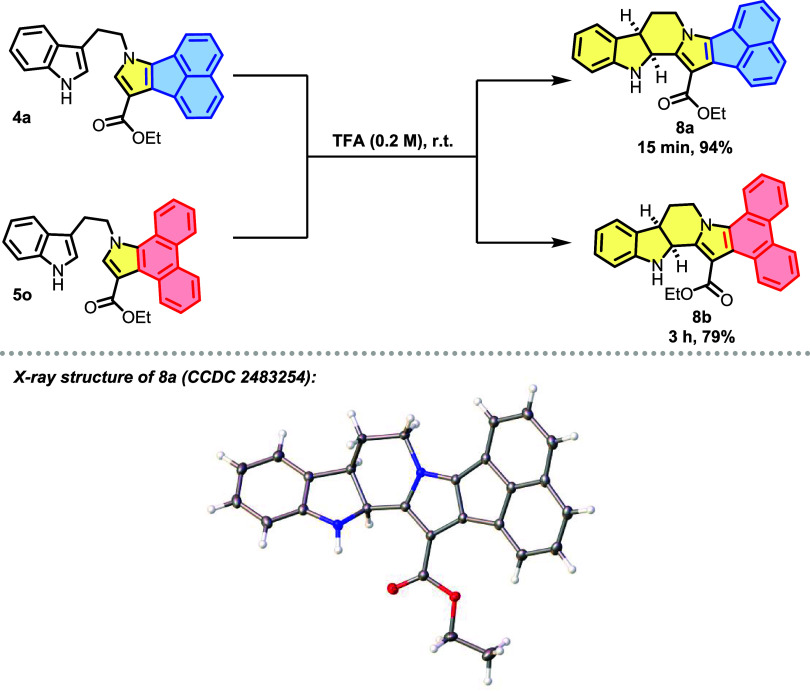
Cyclization Reactions of **4a** and **5o** under Acidic Conditions

Next, we investigated the Brønsted acid-promoted intramolecular cyclization of compounds **4a** and **5o** according to the procedure reported by Cicolini and co-workers.^49^ After treating derivative **4a** with trifluoroacetic acid (TFA) following **General Procedure E**, we obtained the indoline derivative **8a**, featuring an intriguing polycyclic skeleton, with an impressive yield of 94% (Scheme 9). This dearomatization reaction results in the formation of two stereocenters and rapidly establishes the entire polycyclic system in just 15 min, completely consuming the starting material. Fortunately, we were able to obtain single crystals suitable for single-crystal X-ray diffraction analysis. The collected data revealed that **8a** adopts a *cis* relative stereochemistry on the 2,3-fused indoline moiety. Additionally, the analogous **5o** was subjected to the same reaction conditions to obtain **8b**.

Finally, an oxidation methodology for β-carboline ester **8b** was developed using DDQ (2,3-Dichloro-5,6-dicyano-1,4-benzoquinone), which enabled the formation of the polyaromatic derivative **9** with good yield (Scheme 10).^50–52^ It is worth noting that compound **9** bears the structural core of the marine-derived alkaloid fascaplysin and, more specifically, is a structural analog of its metabolites homofascaplycins B and C, both natural products with several biological activities reported.^53–55^

**Scheme 10:**
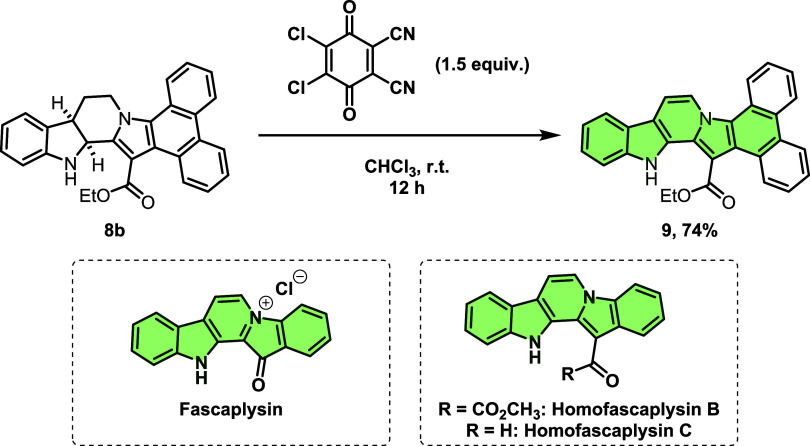
Oxidation Reaction of Polycyclic *N*-Heterocycle **8b** Using DDQ

Most synthesized molecules were used for in vitro testing against a cell panel of leukemia cell lines: Jurkat (acute lymphoblastic leukemia T, ALL-T) and NB4 (acute myeloid leukemia, AML). NB4 cells were kindly provided by from Prof. Eduardo M. Rego (University of Sao Paulo, Brazil); Jurkat cells from Prof. Sara T. Olalla Saad (University of Campinas, Brazil). The effects of molecules (0–50 μM) on cell viability were evaluated by methylthiazoletetrazolium (MTT, Sigma-Aldrich, St. Louis, MO, USA) assay as previously described.^56^ Inhibitory Concentration 50% (IC50) values were calculated by nonlinear regression using GraphPad Prism 8 (GraphPad Software, Inc. San Diego, CA, USA) (Figure S86). All structures and their respective activities against the leukemia cell lines are shown in Figures 2, 3 and 4. It is noteworthy that compounds with IC50 above 50 μM for both cell lines were marked in red. For molecules with IC50 between 50 and 20 μM, they were marked in yellow. In cases where activity was less than 20 μM for only one of the lines, the molecules were marked in green to indicate some selectivity. Finally, molecules marked in orange demonstrated activity against both cell lines, indicating potency but low selectivity.

**Figure 2:**
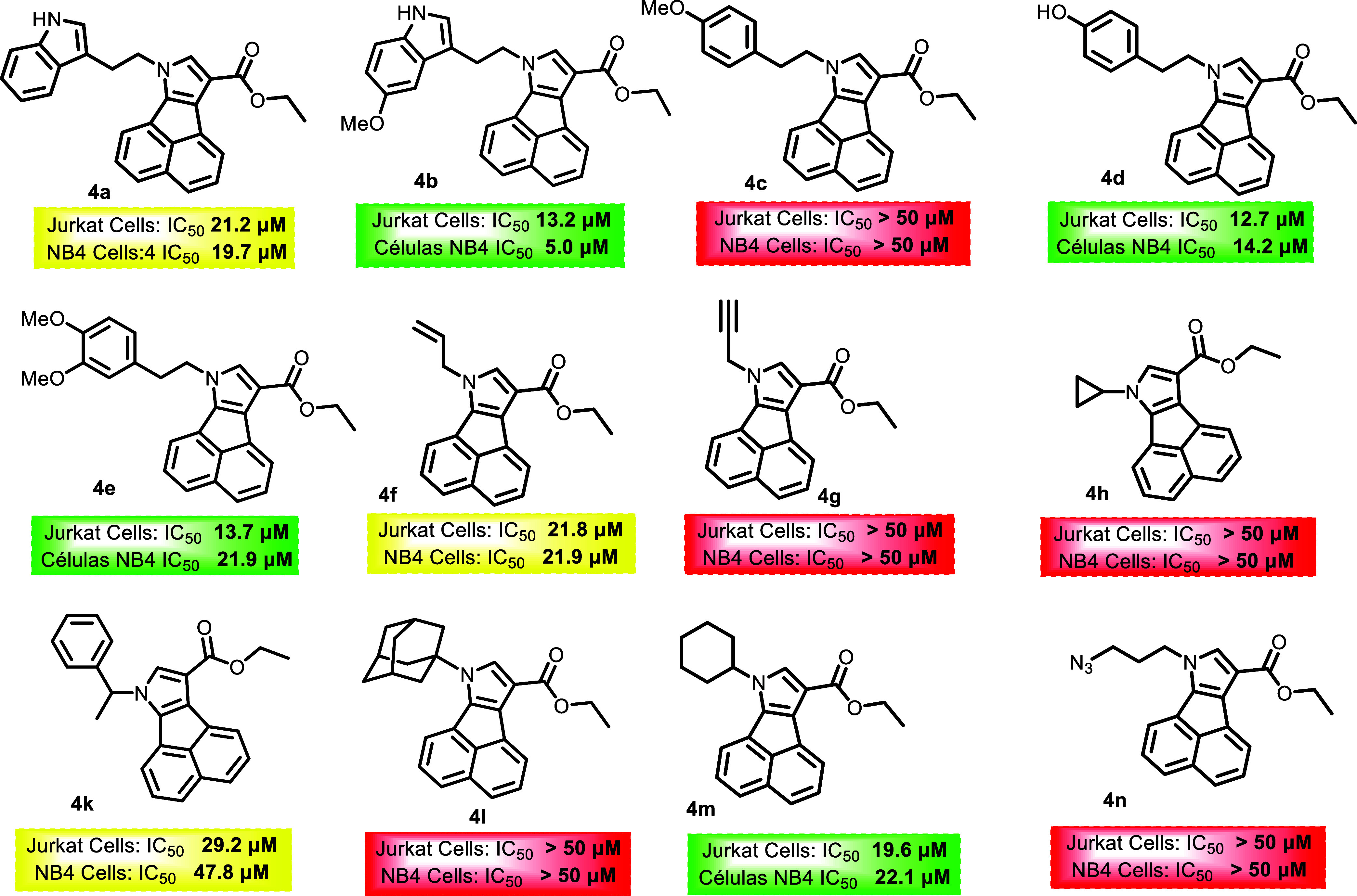
Results of the evaluation of *N*-substituted acenaphtho­[1,2-*b*]­pyrrole derivatives synthesized in proliferation cell lines, Expressed at IC_50_ (μM).

**Figure 3:**
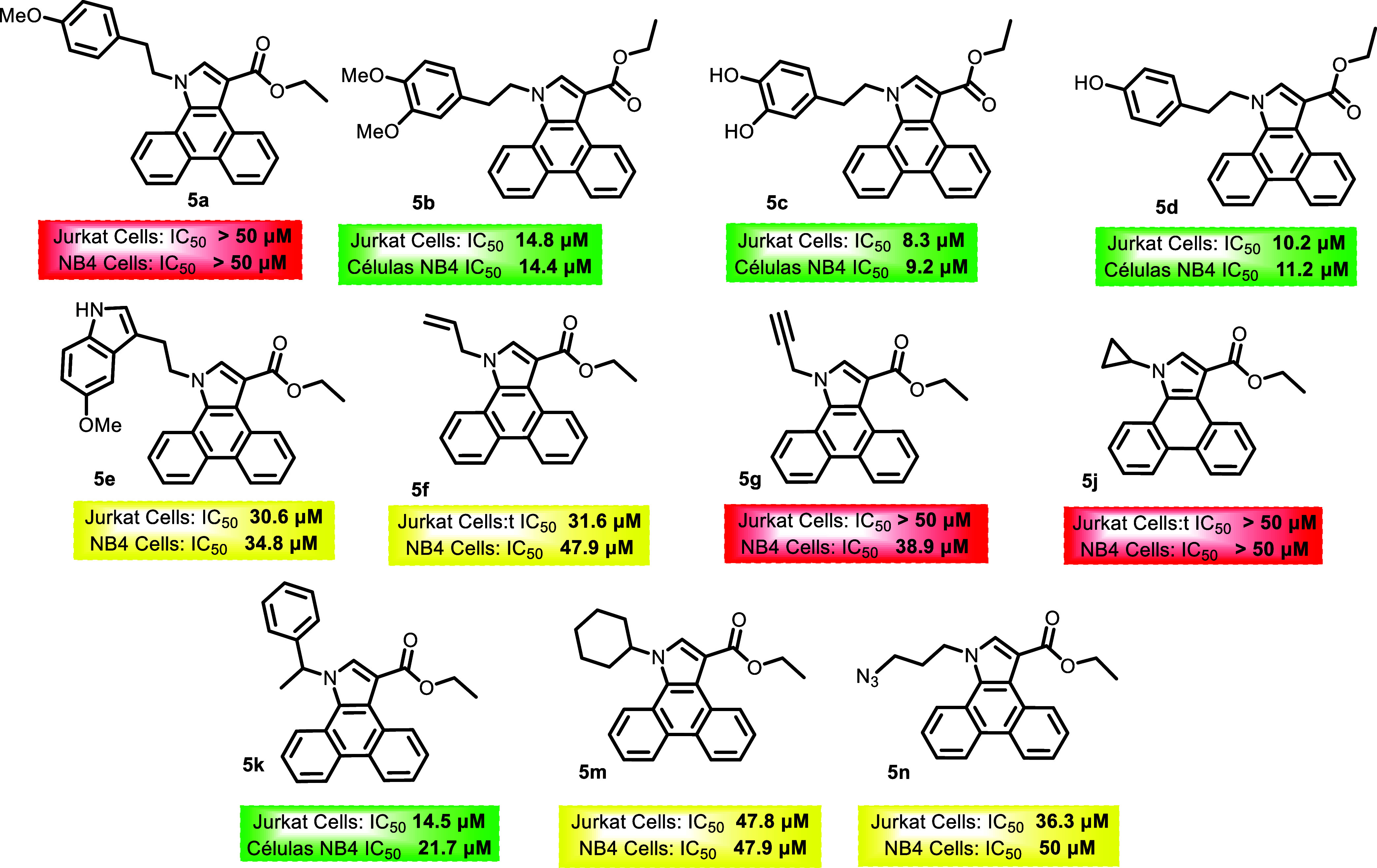
Results of the evaluation of *N*-substituted dibenzo­[*e,g*]­indoles derivatives synthesized in proliferation cell lines, Expressed at IC_50_ (μM).

**Figure 4:**
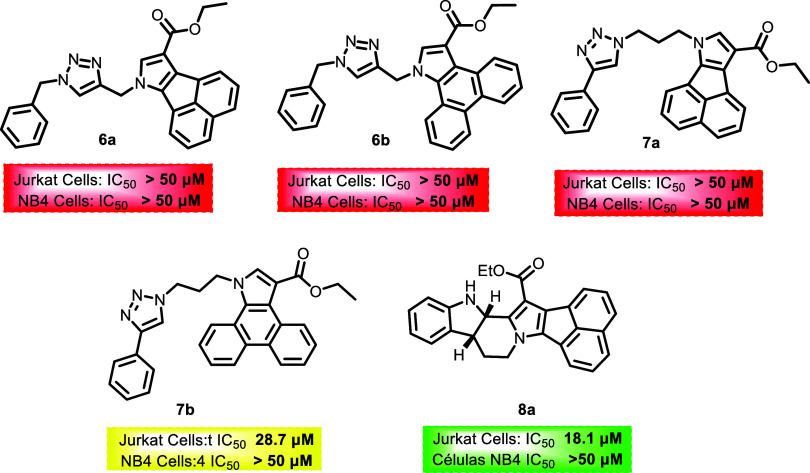
Results of the evaluation of triazole derivatives synthesized in proliferation cell lines, Expressed at IC_50_ (μM).

The triazole compounds prepared via click reactions did not demonstrate satisfactory activity, except for **7b**, with an IC_50_ of 28.7 μM for Jurkat cells. Although this value is above the cutoff, it shows some potential since it exhibited selectivity. Compound **4b** showed the greatest potency against the NB4 cell line, with an IC_50_ of 5.0 μM, while **5c** had an IC_50_ of 8.3 μM for the Jurkat cell line. It is important to note that none of these compounds demonstrated selectivity. Despite advances in the understanding of pathophysiological mechanisms and the development of targeted therapies, acute leukemias in adult patients remain a major therapeutic challenge.^56^ *N*-substituted polycyclic heterocycles are emerging as promising compounds for leukemia treatment, acting primarily through the inhibition of critical kinases (such as BCR::ABL1 and FLT3, targeted by imatinib and midostaurin, respectively). These diverse mechanisms enable innovative therapeutic strategies, particularly in resistant leukemias, although challenges such as selective toxicity, drug resistance, and synthetic complexity still need to be addressed.^57–60^ Therefore, the identification of new molecules with antileukemic activity is of great importance, and further studies will be conducted to expand upon our findings.

A limited and exploratory structure–activity relationship (SAR) analysis can be proposed based on the antiproliferative evaluation of the synthesized compounds. Overall, differences in biological activity were observed among the distinct polycyclic scaffolds investigated, indicating that the core structure significantly influences the cellular response, a principle that underlies structure–activity relationship-driven optimization in medicinal chemistry.^61^ In general terms, *N*-substituted acenaphtho­[1,2-*b*]­pyrrole derivatives showed a tendency toward lower IC_50_ values compared with the corresponding dibenzo­[*e,g*]­indole analogues, although this trend was not uniform across all substitutions. Variations in the *N*-substituent were also associated with changes in activity, suggesting that the nature of this substituent modulates the biological profile. However, no clear linear correlation between substituent size or electronic properties and potency could be established at this stage, consistent with the complex and often nonlinear nature of structure–activity relationships.^62^ The triazole derivatives obtained via azide–alkyne cycloaddition displayed, in most cases, reduced antiproliferative activity relative to their parent compounds, indicating that this specific derivatization does not enhance activity in the evaluated cell lines. This observation highlights that, while click chemistry is a versatile strategy for molecular diversification, it does not inherently guarantee improved biological potency.^63^ Although most compounds showed limited selectivity between Jurkat (T-ALL) and NB4 (LMA) cells, occasional differences in sensitivity were observed. These findings suggest that further structural refinement may enable modulation of both potency and selectivity, which remains a key objective in the development of targeted therapies for hematologic malignancies.^64^ Taken together, these observations represent preliminary SAR insights that provide a starting point for future optimization rather than definitive conclusions.

## Materials and Methods

All solvents and reagents were obtained from commercial suppliers and used without further purification. Reaction progress was monitored by thin-layer chromatography (TLC) on silica gel (aluminum plates), visualized under UV light at 254 or 366 nm, followed by revelation with an ethanolic anisaldehyde solution or a 2,4-dinitrophenylhydrazine (DNPH) solution. Product purification was carried out by flash chromatography on silica gel (70–230 mesh).

^1^H NMR spectra were recorded at 250, 300, 400, 500, and 600 MHz, while ^13^C NMR spectra were obtained at 63, 75, 100, 125, and 150 MHz, using CDCl_3_ or DMSO-*d*_6_ as solvents. Chemical shifts (δ) are reported in parts per million (ppm), and coupling constants (J) in Hertz (Hz). Signal multiplicities are designated as singlet (s), doublet (d), doublet of doublets (dd), triplet (t), doublet of triplets (dt), triplet of doublets (td), quartet (q), doublet of doublets of doublets (ddd), doublet of doublets of doublets of doublets (dddd), doublet of doublets of triplets (ddt), multiplet (m), and broad (br).

High-resolution mass spectra (HRMS) were obtained using a Q-Tof device configured with ESI-QqToF, with a resolution of 5,000 and an accuracy of 50.0 ppm in the TOF mass analyzer. Compounds were named according to IUPAC rules, using appropriate free software. Only spectroscopic data of novel compounds are included in the experimental section.

## Conclusions

In conclusion, this study explored an efficient synthetic route to structurally complex *N*-substituted polycyclic heterocycles—acenaphtho­[1,2-*b*]­pyrroles and dibenzo­[*e,g*]­indoles—with potential antileukemic activity. The approach employed Morita–Baylis–Hillman (MBH) adducts derived from polycyclic diketones, followed by acetylation and cyclization with primary amines. This methodology enabled the preparation of 25 unprecedented *N*-substituted polycyclic derivatives with yields up to 97% from the MBH acetates. We also explored the postfunctionalization of the resulting *N*-substituted polycyclic heterocycles via “click” reactions and Brønsted acid-promoted intramolecular cyclization. Several synthesized compounds, particularly compound **4b**, showed promising in vitro activity against leukemia cell lines. Although the results are encouraging, issues of selectivity and toxicity should be addressed in future studies.

## Supplementary Material







## Notes

2483262 (CIF)
2483254 (CIF)
Detailed molecular characterization (^1^H NMR, ^13^C NMR and HRMS analysis) and synthetic procedures (PDF)
