# Supplementary material for: Synthesis of N‑Substituted Acenaphtho[1,2‑b]pyrroles and Dibenzo[e,g]indoles with Promising Antileukemic Activity from Morita–Baylis–Hillman Adducts
Source: ACS Omega. 2026 Mar 6;11(10):16590–602. doi: 10.1021/acsomega.5c11609 (PMC13000790; doi:10.1021/acsomega.5c11609)
Supplement: Supplementary file 3 [file ao5c11609_si_003.pdf]

Supporting Information (S.I.)

SYNTHESIS OF *N*-SUBSTITUTED ACENAPHTHO[1,2-*b*]PYRROLES AND DIBENZO[*e,g*]INDOLES WITH PROMISING ANTI-LEUKEMIC ACTIVITY FROM MORITA–BAYLIS–HILLMAN ADDUCTS

João Arantes[a], Manoel T. Rodrigues Jr.[a], Giovani Rosendo[a], Rafael Porreca[a] Hugo P. Vicari[b], Hugo Santos[a], João A. Machado-Neto[b], and Fernando Coelho[a*]

[a] *Department of Organic Chemistry, Chemistry Institute, University of Campinas, Campinas, São Paulo, SP, 13083-970, Brazil.*

[b] *Department of Pharmacology, Institute de Biomedical Sciences, University of São Paulo, São Paulo, SP, 05508-900, Brazil.*

* *Corresponding author. Institute of Chemistry, Universidade Estadual de Campinas, PO Box 6154, 13083-970 Campinas, SP, Brazil.*

e-mail: facoelho@unicamp.br

SUPPORTING INFORMATION

S2	*General Information*
S3-S6	*General procedures A–F*
S7-S26	*Characterization data for synthesized compounds*
S27-S113	*Copies of ^{1}H NMR and ^{13}C NMR Spectra; NOESY, HSQC and HSQC*
S114	*Copies of dose-response curves for synthesized compounds*
S115-S119	*Crystallographic details*

General information

All solvents and reagents were obtained from commercial suppliers and used without further purification. Reaction progress was monitored by thin-layer chromatography (TLC) on silica gel (aluminum plates), visualized under UV light at 254 or 366 nm, followed by revelation with an ethanolic anisaldehyde solution or a 2,4-dinitrophenylhydrazine (DNPH) solution. Product purification was carried out by flash chromatography on silica gel (70–230 mesh).

^{1}H NMR spectra were recorded at 250, 300, 400, 500, and 600 MHz, while ^{13}C NMR spectra were obtained at 63, 75, 100, 125, and 150 MHz, using $CDCl_3$ or DMSO-d_6 as solvents. Chemical shifts (δ) are reported in parts per million (ppm), and coupling constants (J) in Hertz (Hz). Signal multiplicities are designated as singlet (s), doublet (d), doublet of doublets (dd), triplet (t), doublet of triplets (dt), triplet of doublets (td), quartet (q), doublet of doublets of doublets (ddd), doublet of doublets of doublets of doublets (dddd), doublet of doublets of triplets (ddt), multiplet (m), and broad (br).

High-resolution mass spectra (HRMS) were obtained using a Q-Tof device configured with ESI-QqToF, with a resolution of 5,000 and an accuracy of 50.0 ppm in the TOF mass analyzer. Compounds were named according to IUPAC rules, using appropriate free software. Only spectroscopic data of novel compounds are included in the experimental section.

General procedures A–F

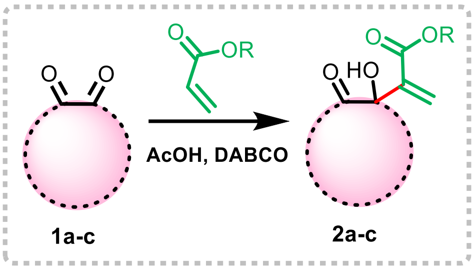

General procedure A – MBH reactions.

General procedure A for synthesizing compounds **2a-c** as exemplified for **2a** – (*ethyl 2-(1-hydroxy-2-oxo-1,2-dihydroacenaphthylen-1-yl)prop-2-enoate*): In a 100 mL round-bottom flask, a mixture of acenaphthene-1,2-dione (**1a**) (1.02 g; 5.6 mmol; 1.0 equiv), ethyl acrylate (1.19 mL; 11.2 mmol; 2.0 equiv), DABCO (1.26 g; 11.2 mmol; 2.0 equiv) and acetic acid (0.64 mL; 11.2 mmol; 2.0 equiv) was added. The mixture was stirred at room temperature for 5 h. The residual acrylate was removed under reduced pressure. Then 35 mL of distilled water were added, and the reaction was extracted with ethyl acetate (2 × 40 mL). The combined organic layers were washed with 1 M HCl (1 × 30 mL), saturated $NaHCO_3$ solution (2 × 30 mL) and brine (1 × 20 mL). The resulting crude product was purified by column chromatography using a gradient of ethyl acetate:hexane from 1:9 to 3:7.

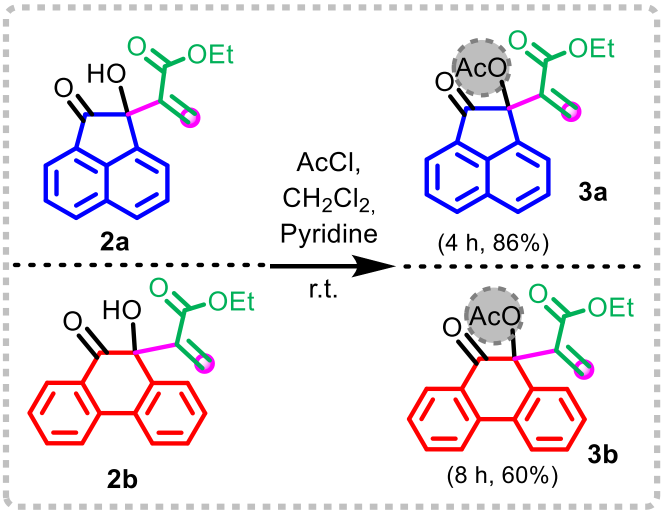

General procedure B – MBH acetate formation.

General procedure B for synthesizing compounds **3a** and **3b,** as exemplified for **3a** – (*ethyl 2-(1-acetoxy-2-oxo-1,2-dihydroacenaphthylene-1-yl)prop-2-enoate*): A 2.2 M

solution of the corresponding MBH adduct (**3a**) (1.0 equiv) in dichloromethane was prepared, to which pyridine (2.3 equiv) was added. Acetyl chloride (2.3 equiv) was then added to the mixture with stirring at 0 °C, after which the reaction was allowed to warm to room temperature. When no further reaction progress was observed by TLC, 25 mL of distilled water were added and the reaction was extracted with dichloromethane (3 × 25 mL). The combined organic layers were washed with brine (25 mL), dried over anhydrous Na_2SO_4, and concentrated under reduced pressure to give the crude product. The product was purified by column chromatography (ethyl acetate:hexane, from 5:95 to 15:85).

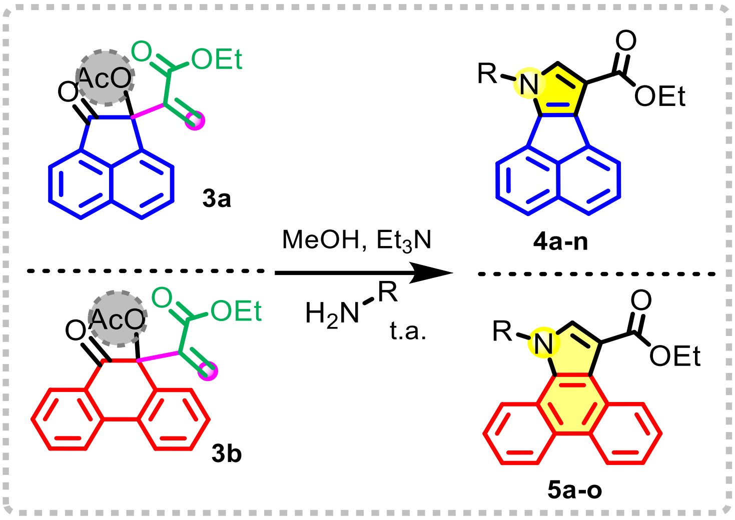

General procedure C – Synthesis of polycyclic *N*-heterocycles.

General procedure C for synthesizing polycyclic *N*–heterocycles **4a-n** and **5a-o** as exemplified for compound **4b** (*ethyl 7-[2-(5-methoxy-1H-indol-3-yl)ethyl]-7H-acenaphthyleno[1,2-b]pyrrole-9-carboxylate*): In a 100 mL round-bottom flask, a mixture of a 0.2 M methanolic solution of the acetylated adduct **3a** (1.0 equiv) and triethylamine (2.0 equiv) was added. The corresponding primary amine (1.05 equiv), in this case 5-methoxytryptamine, was slowly added. The reaction was concentrated under reduced pressure and purified by column chromatography (ethyl acetate:hexane, from 5:95 to 20:80).

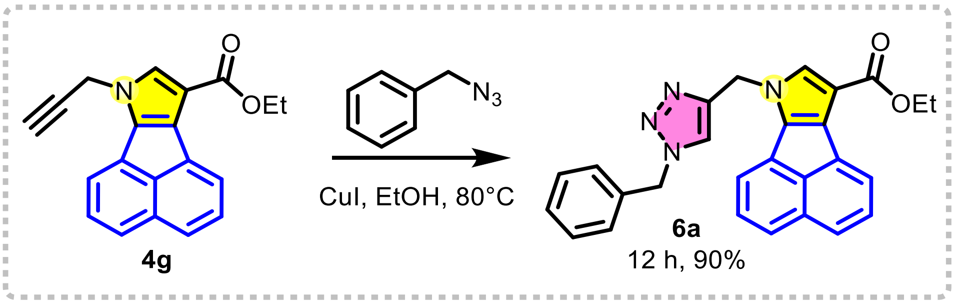

General procedure D – Click reactions.

General procedure D for synthesis of triazoles **6a-b** and **7a-b** from azide or alkyne derivatives as exemplified for compound **6a** (*ethyl 7-[(1-benzyl-1H-1,2,3-triazol-4-yl)methyl]-7H-acenaphthyleno[1,2-b]pyrrole-9-carboxylate*): In a 50 mL round-bottom flask, a mixture of the corresponding alkyne or azide was added; in this case the alkyne **4g** (1.0 equiv) was dissolved in absolute ethanol to give a final concentration of approximately 0.055 M. Copper(I) iodide (CuI) (0.1 equiv) and the corresponding azide (1.5 equiv) were then added, and the reaction mixture was stirred at 80 °C for 12 h until complete consumption of the starting material. The reaction mixture was concentrated under reduced pressure and purified by column chromatography (ethyl acetate:hexane, from 1:4 to 1:1).

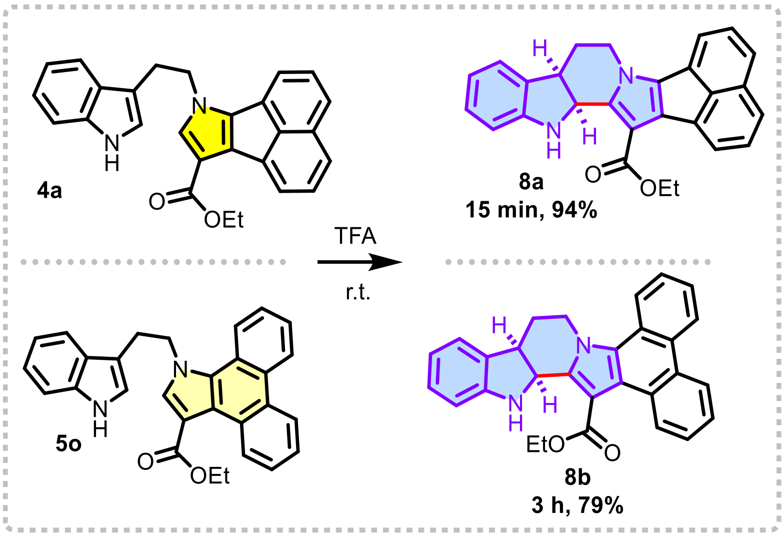

General procedure E – Dearomative cyclization reactions.

General procedure E for synthesizing compounds **8a** and **8b**.: Starting from the corresponding indole **4a** or **5o**, the substrate was placed in a 25 mL round-bottom flask, and a 0.2 M solution of trifluoroacetic acid (TFA) was added slowly with magnetic stirring. Fume evolution was observed—keep the fume hood on and closed. The reaction was monitored by TLC after performing a micro-extraction of an aliquot into saturated sodium bicarbonate solution and ethyl acetate. After complete consumption of the starting material was observed, saturated $NaHCO_3$ solution.

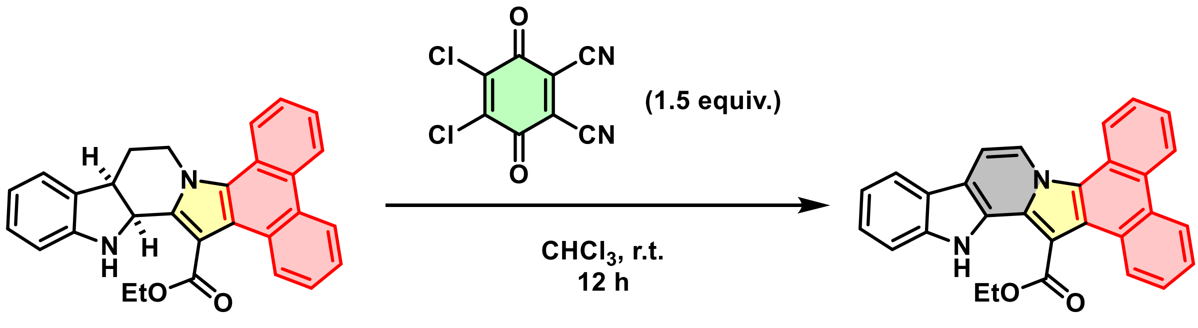

General procedure F – DDQ oxidation.

General procedure F for synthesizing compound **9**: Starting from the corresponding indoline **8b** (101.5 mg; 0.25 mmol; 1.0 equiv) was placed in a 100 mL round-bottom flask in the presence of DDQ (340 mg; 1.5 mmol; 6.0 equiv) in 25 mL of chloroform and stirred at room temperature. After 12 h, and when no further reaction progress was observed by TLC, the reaction mixture was concentrated under reduced pressure and purified by column chromatography (ethyl acetate:hexane, from 1:19 to 2:8). (Note: attempts to extract the reaction mixture with bisulfite, metabisulfite, bicarbonate, or sodium carbonate led to the formation of emulsions.)

Characterization data for synthesized compounds

***2a** – ethyl 2-(1-hydroxy-2-oxo-1,2-dihydroacenaphthylen-1-yl)prop-2-enoate.*

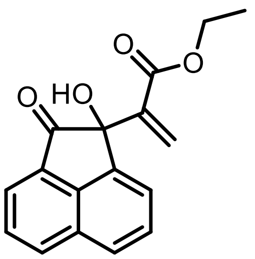

Following general procedure A, reaction for 6 h gave compound **2a**[1] in quantitative yield as a white solid; mp – 125-127 °C. **^{1}H NMR (250 MHz, CDCl$_3$)** δ 8.12 (dd, J = 8.2, 0.8 Hz, 1H), 8.01 (dd, J = 7.0, 0.8 Hz, 1H), 7.90 (dd, J = 8.4, 0.8 Hz, 1H), 7.75 (dd, J = 8.2, 7.0 Hz, 1H), 7.62 (dd, J = 8.4, 6.9 Hz, 1H), 7.47 (dd, J = 6.9, 0.8 Hz, 1H), 6.63 (d, J = 0.9 Hz, 1H), 6.48 (d, J = 0.9 Hz, 1H), 3.85 (q, J = 7.1 2H), 3.69 (s, 1H), 0.79 (t, J = 7.1 Hz, 3H).**^{13}C NMR (63 MHz, CDCl$_3$)** δ 202.1, 164.9, 142.1, 140.2, 139.1, 131.7, 131.6, 130.8, 128.6, 128.3, 127.6, 125.9, 122.5, 120.5, 79.9, 60.9, 13.4. HRMS (ESI): m/z calculated for $C_{17}H_{14}O_4Na^+$ $[M-Na]^+$: 305.0784, found 305.0781.

2b – *ethyl 2-(9-hydroxy-10-oxo-9,10-dihydrophenanthren-9-yl)prop-2-enoate.*

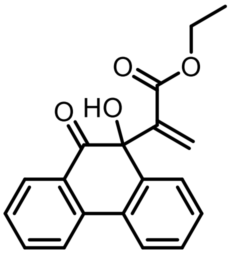

Following general procedure A, reaction for 5 days gave compound **2b** (75% yield) as an orange oil. **^{1}H NMR (250 MHz, CDCl$_3$)** δ 8.02 – 7.80 (m, 3H), 7.72 – 7.55 (m, 2H), 7.46 – 7.30 (m, 3H), 6.25 (s, 1H), 5.70 (s, 1H), 4.64 (bs, 1H), 4.06 (d, J = 7.0 Hz, 2H), 1.10 (t, J = 7.1 Hz, 3H). **^{13}C NMR (63 MHz, CDCl$_3$)** δ 199.9, 165.4, 142.8, 137.3, 137.0, 134.6, 130.8, 129.3, 129.1, 129.1, 128.6, 128.0, 127.7, 127.5, 123.9, 123.0,

[1] Khalafi-Nezhad, A. and Mohammadi, S. Highly Efficient Synthesis of Novel Morita–Baylis–Hillman Adducts from Activated Ketones Using a DABCO-Based Hydroxy Ionic Liquid (HIL) as a Recyclable Catalyst-Solvent Synthesis of Novel Morita–Baylis–Hillman Adducts Using HIL, *Synthesis* **2012**, 44, 1725-1735.

78.5, 61.2, 13.8. HRMS (ESI): m/z calculated for $C_{19}H_{16}O_4Na^+$ [M-Na]$^+$: 331.0941, found 331.0938.

2c – *methyl 3-hydroxy-2-methylene-4-oxo-3,4-diphenylbutanoate.*

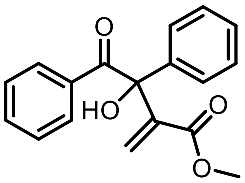

Following general procedure A, reaction for 3 days gave compound **2d** (16% yield) as a transparent oil. **^{1}H NMR (300 MHz, CDCl$_3$)** δ 7.94 (dd, J = 8.4, 1.4 Hz, 2H), 7.63 – 7.55 (m, 2H), 7.51 – 7.27 (m, 6H), 6.35 (s, 1H), 5.19 (s, 1H), 3.86 (s, 3H). **^{13}C NMR (75 MHz, CDCl$_3$)** δ 200.5, 169.0, 144.1, 137.1, 134.7, 132.7, 130.7, 128.7, 128.4, 128.4, 127.9, 126.6, 84.8, 52.6. HRMS (ESI): m/z calculated for $C_{18}H_{16}O_4Na^+$ [M-Na]$^+$: 319.0941, found 319.0929.

3a – *ethyl 2-(1-acetoxy-2-oxo-1,2-dihydroacenaphthylene-1-yl)prop-2-enoate.*

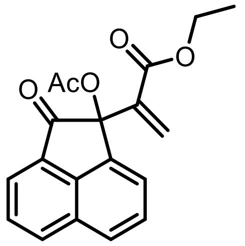

Following general procedure B, reaction for 4 h gave compound **3a** (86% yield) as an orange solid; mp – 111-113 °C. **^{1}H NMR (250 MHz, CDCl$_3$)** δ 8.06 (dd, J = 12.4, 7.6 Hz, 2H), 7.91 (d, J = 8.3 Hz, 1H), 7.76 (dd, J = 8.2, 7.0 Hz, 1H), 7.60 (dd, J = 8.3, 6.9 Hz, 1H), 7.48 (d, J = 6.9 Hz, 1H), 6.67 – 6.62 (m, 1H), 6.56 (s, 1H), 3.76 (q, J = 7.2 Hz, 2H), 2.08 (s, 3H), 0.68 (t, J = 7.2 Hz, 3H). **^{13}C NMR (63 MHz, CDCl$_3$)** δ 197.9, 168.2, 164.0, 141.7, 138.0, 136.6, 133.7, 130.8, 130.3, 128.2, 128.0, 127.6, 126.5, 121.3, 120.4, 83.7, 60.9, 20.6, 13.2. HRMS (ESI): m/z calculated for $C_{19}H_{17}O_5^+$ [M-H]$^+$: 325.1070, found 325.1071.

3b – *ethyl 2-[9-(acetyloxy)-10-oxo-9,10-dihydrophenanthren-9-yl]prop-2-enoate.*

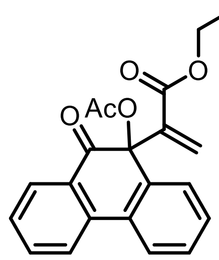

Following general procedure B, reaction for 6 h gave compound **3b** (60% yield) as a dark green oil. **^{1}H NMR (250 MHz, CDCl$_3$)** δ 8.27 – 8.05 (m, 3H), 7.69 (ddd, J = 8.0, 7.3, 1.5 Hz, 1H), 7.53 – 7.29 (m, 4H), 6.63 (dd, J = 5.9, 0.9 Hz, 2H), 3.77 (q, J = 7.2 Hz, 2H), 2.06 (s, 3H), 0.75 (t, J = 7.2 Hz, 3H). **^{13}C NMR (63 MHz, CDCl$_3$)** δ 192.4, 168.5, 163.9, 140.8, 136.6, 136.0, 133.9, 131.7, 130.8, 129.0, 128.7, 128.3, 127.7, 127.2, 126.5, 123.1, 123.0, 78.3, 61.1, 20.7, 13.1. HRMS (ESI): m/z calculated for $C_{20}H_{18}NO_2^+$ [M-H]$^+$: 351.1227, found 351.1220.

4a – *ethyl 7-[2-(1H-indol-3-yl)ethyl]-7H-acenaphthyleno[1,2-b]pyrrole-9-carboxylate.*

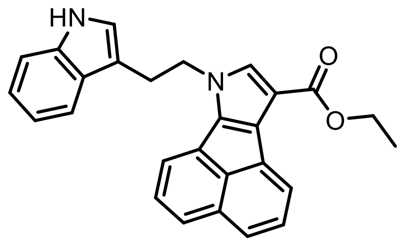

Following general procedure C, reaction for 2 h gave compound **4a** (90% yield) as an orange solid; mp – 164-165 °C. **^{1}H NMR (250 MHz, CDCl$_3$)** δ 8.12 (d, J = 6.8 Hz, 1H), 7.99 (s, 1H), 7.75 – 7.50 (m, 4H), 7.44 – 7.15 (m, 6H), 6.77 (d, J = 2.5 Hz, 1H), 4.45 (q, J = 6.8 Hz, 4H), 3.41 (t, J = 7.0 Hz, 2H), 1.50 (t, J = 7.1 Hz, 3H). **^{13}C NMR (63 MHz, CDCl$_3$)** δ 165.1, 138.4, 136.3, 132.7, 132.5, 130.3, 129.4, 128.9, 128.1, 127.0, 126.8, 126.0, 125.7, 123.1, 122.6, 122.3, 119.7, 118.2, 118.2, 111.5, 111.4, 111.1, 60.1, 50.0, 26.6, 14.8. HRMS (ESI): m/z calculated for $C_{27}H_{23}N_2O_2^+$ [M-H]$^+$: 407.1754, found 407.1750.

4b – *ethyl 7-[2-(5-methoxy-1H-indol-3-yl)ethyl]-7H-acenaphthyleno[1,2-b]pyrrole-9-carboxylate.*

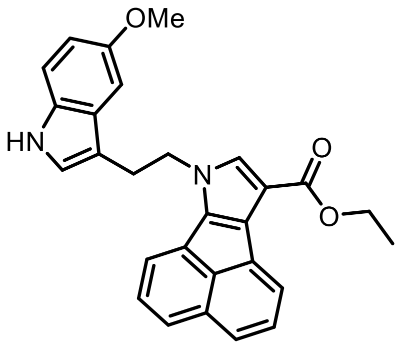

Following general procedure C, reaction for 2,5 h gave compound **4b** (92% yield) as an orange solid; mp – 194-196 °C. **^{1}H NMR (250 MHz, DMSO-d$_6$)** δ 10.68 (s, 1H), 7.97 (d, J = 6.8 Hz, 1H), 7.83 – 7.65 (m, 3H), 7.61 – 7.47 (m, 3H), 7.19 (d, J = 8.8 Hz, 1H), 7.10 (d, J = 2.4 Hz, 1H), 6.98 (d, J = 2.5 Hz, 1H), 6.68 (dd, J = 8.7, 2.4 Hz, 1H), 4.62 – 4.51 (m, 2H), 4.32 (q, J = 7.1 Hz, 2H), 3.68 (s, 3H), 3.28 (s, 2H), 1.38 (t, J = 7.1 Hz, 3H). **^{13}C NMR (151 MHz, DMSO-d$_6$)** δ 164.3, 153.6, 138.3, 132.6, 132.1, 131.7, 131.7, 129.5, 128.8, 128.4, 127.9, 127.8, 127.4, 126.4, 126.1, 124.4, 123.0, 119.6, 112.5, 111.8, 110.6, 110.5, 100.3, 59.9, 55.6, 49.9, 26.5, 15.0. HRMS (ESI): m/z calculated for $C_{28}H_{25}N_2O_3^+$ $[M-H]^+$: 437.1860, found 437.1859.

4c – *ethyl 7-[2-(4-methoxyphenyl)ethyl]-7H-acenaphthyleno[1,2-b]pyrrole-9-carboxylate.*

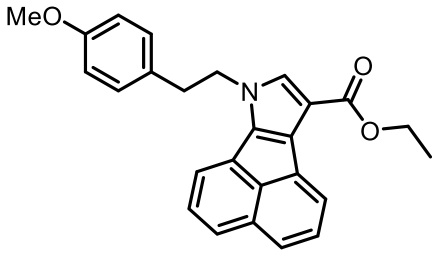

Following general procedure C, reaction for 10 min gave compound **4c** (93% yield) as a yellow solid; mp – 139-141 °C. **^{1}H NMR (250 MHz, DMSO-d$_6$)** δ 7.98 (d, J = 6.8 Hz, 1H), 7.75 (dt, J = 10.4, 5.5 Hz, 3H), 7.65 – 7.40 (m, 3H), 7.15 (d, J = 8.0 Hz, 2H), 6.81 (d, J = 8.1 Hz, 2H), 4.48 (t, J = 7.2 Hz, 2H), 4.32 (q, J = 7.1 Hz, 2H), 3.68 (s, 3H), 3.13 (t, J = 7.2 Hz, 2H), 1.38 (t, J = 7.1 Hz, 3H). **^{13}C NMR (63 MHz, DMSO-d$_6$)** δ 164.3, 158.5, 138.2, 132.6, 132.1, 131.6, 130.3, 130.1, 129.5, 128.7, 128.4, 127.9, 127.4,

126.4, 126.2, 123.0, 119.7, 114.3, 110.5, 60.0, 55.4, 50.5, 35.6, 15.0. HRMS (ESI): m/z calculated for $C_{26}H_{24}NO_3^+$ [M-H]$^+$: 398.1750, found 398.1749.

4d - *ethyl 7-[2-(4-hydroxyphenyl)ethyl]-7H-acenaphthyleno[1,2-b]pyrrole-9-carboxylate.*

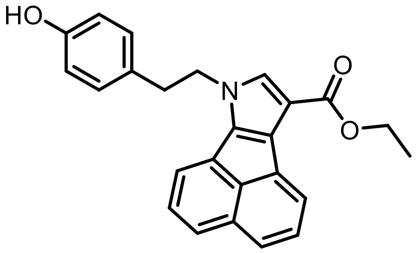

Following general procedure C, reaction for 1,5 h gave compound **4d** (83% yield) as a yellow solid; mp – 175-176 °C. **^{1}H NMR (250 MHz, DMSO-d$_6$)** δ 9.21 (s, 1H), 7.98 (d, J = 6.8 Hz, 1H), 7.84 – 7.68 (m, 3H), 7.56 (td, J = 7.3, 3.1 Hz, 2H), 7.45 (s, 1H), 7.08 – 6.99 (m, 2H), 6.69 – 6.60 (m, 2H), 4.46 (t, J = 7.2 Hz, 2H), 4.32 (q, J = 7.1 Hz, 2H), 3.08 (t, J = 7.3 Hz, 2H), 1.38 (t, J = 7.1 Hz, 3H). **^{13}C NMR (63 MHz, DMSO-d$_6$)** δ 164.3, 156.4, 138.2, 132.6, 132.1, 131.6, 130.2, 129.5, 128.7, 128.4, 128.3, 127.9, 127.4, 126.4, 126.2, 123.0, 119.7, 115.7, 110.4, 60.0, 50.7, 35.7, 15.0. HRMS (ESI): m/z calculated for $C_{25}H_{22}NO_3^+$ [M-H]$^+$: 384.1594, found 384.1592.

4e - *ethyl 7-[2-(3,4-dimethoxyphenyl)ethyl]-7H-acenaphthyleno[1,2-b]pyrrole-9-carboxylate.*

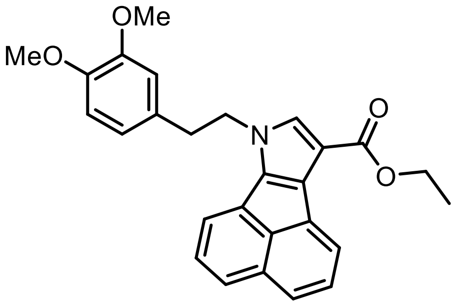

Following general procedure C, reaction for 2 h gave compound **4e** (89% yield) as a yellow solid; mp – 115-116°C. **^{1}H NMR (250 MHz, DMSO-d$_6$)** δ 7.98 (d, J = 6.8 Hz, 1H), 7.83 – 7.65 (m, 3H), 7.65 – 7.37 (m, 3H), 6.91 – 6.62 (m, 3H), 4.51 (t, J = 7.0 Hz, 2H), 4.32 (q, J = 7.1 Hz, 2H), 3.65 (s, 4H), 3.64 (s, 3H), 3.12 (t, J = 7.0 Hz, 2H), 1.38 (t, J = 7.1 Hz, 3H). **^{13}C NMR (63 MHz, DMSO-d$_6$)** δ 164.3, 149.1, 148.0, 138.3, 132.6, 132.1, 131.6, 130.7, 129.5, 128.7, 128.4, 127.8, 127.4, 126.4, 126.2, 123.0, 121.3, 119.7,

113.2, 112.3, 110.5, 60.0, 55.9, 55.7, 50.4, 36.1, 15.0. HRMS (ESI): m/z calculated for $C_{27}H_{26}NO_4^+$ [M-H]$^+$: 428.1856, found 428.1855.

4f - *ethyl 7-(prop-2-en-1-yl)-7H-acenaphthyleno[1,2-b]pyrrole-9-carboxylate.*

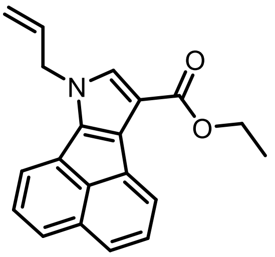

Following general procedure C, reaction for 2 h gave compound **4f** (58% yield) as a red solid; mp – 106-108 °C. **^{1}H NMR (250 MHz, $CDCl_3$)** δ 8.12 (dd, J = 6.8, 0.9 Hz, 1H), 7.75 – 7.64 (m, 2H), 7.56 (dd, J = 8.3, 6.8 Hz, 1H), 7.50 – 7.42 (m, 2H), 7.32 (s, 1H), 6.08 (ddt, J = 17.0, 10.5, 5.3 Hz, 1H), 5.51 – 5.16 (m, 2H), 4.74 (dt, J = 5.4, 1.7 Hz, 2H), 4.46 (q, J = 7.1 Hz, 2H), 1.51 (t, J = 7.1 Hz, 3H).**^{13}C NMR (126 MHz, $CDCl_3$)** δ 164.9, 138.6, 132.6, 132.5, 132.0, 130.0, 129.4, 128.8, 128.2, 128.0, 126.8, 126.1, 125.7, 123.2, 118.7, 118.5, 111.5, 60.1, 51.5, 14.8. HRMS (ESI): m/z calculated for $C_{20}H_{18}NO_2^+$ [M-H]$^+$: 304.1332, found 304.1331.

4g - *ethyl 7-(prop-2-yn-1-yl)-7H-acenaphthyleno[1,2-b]pyrrole-9-carboxylate.*

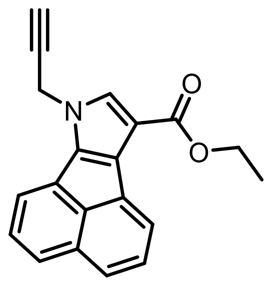

Following general procedure C, reaction for 2 h gave compound **4g** (95% yield) as a white solid; mp – 133-135 °C. **^{1}H NMR (250 MHz, $CDCl_3$)** δ 8.12 (dd, J = 6.9, 0.9 Hz, 1H), 7.72 (ddd, J = 8.3, 4.4, 0.8 Hz, 2H), 7.66 – 7.44 (m, 4H), 4.95 (d, J = 2.6 Hz, 2H), 4.46 (q, J = 7.1 Hz, 2H), 2.56 (t, J = 2.6 Hz, 1H), 1.51 (t, J = 7.1 Hz, 3H). **^{13}C NMR (126 MHz, $CDCl_3$)** δ 164.7, 138.2, 132.4, 132.4, 129.6, 129.4, 128.7, 128.5, 128.1, 126.8, 126.3, 125.9, 123.5, 119.0, 111.8, 76.2, 75.2, 60.1, 38.6, 14.7. HRMS (ESI): m/z calculated for $C_{20}H_{16}NO_2^+$ [M-H]$^+$: 302.1776, found 302.1774.

4j - *ethyl 7-cyclopropyl-7H-acenaphthyleno[1,2-b]pyrrole-9-carboxylate.*

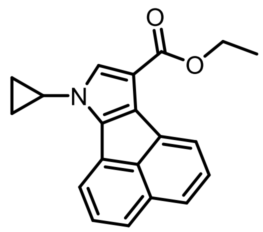

Following general procedure C, reaction for 4 h gave compound **4j** (97% yield) as a yellow solid; mp – 108-110 °C.**^{1}H NMR (250 MHz, CDCl$_3$)** δ 8.09 (dd, J = 6.9, 0.9 Hz, 1H), 7.77 – 7.66 (m, 3H), 7.53 (td, J = 8.4, 6.9 Hz, 2H), 7.40 (s, 1H), 4.45 (q, J = 7.2 Hz, 2H), 3.60 (tt, J = 6.0, 5.0 Hz, 1H), 1.50 (t, J = 7.1 Hz, 3H), 1.23 – 1.15 (m, 4H). **^{13}C NMR (63 MHz, CDCl$_3$)** δ 164.9, 139.9, 132.6, 132.4, 130.2, 129.4, 129.0, 128.0, 127.7, 126.9, 126.2, 125.7, 123.3, 119.3, 111.2, 60.1, 29.5, 14.7, 7.0. HRMS (ESI): m/z calculated for $C_{20}H_{17}NO_2^+$ [M-H]$^+$: 304.1332, found 304.1329.

4k - *ethyl 7-(1-phenylethyl)-7H-acenaphthyleno[1,2-b]pyrrole-9-carboxylate.*

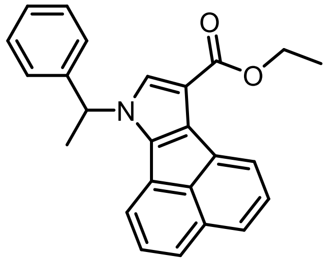

Following general procedure C, reaction for 4 h gave compound **4k** (92% yield) as a yellow solid; mp – 125-126 °C. **^{1}H NMR (500 MHz, CDCl$_3$)** δ 8.02 (d, J = 6.8 Hz, 1H), 7.57 – 7.49 (m, 2H), 7.47 – 7.41 (m, 2H), 7.31 – 7.14 (m, 6H), 7.06 (dd, J = 7.0, 1.3 Hz, 1H), 5.59 (q, J = 7.0 Hz, 1H), 4.43 – 4.32 (m, 2H), 1.89 (dd, J = 7.0, 1.4 Hz, 3H), 1.42 (td, J = 7.1, 1.4 Hz, 3H). **^{13}C NMR (126 MHz, CDCl$_3$)** δ 165.1, 141.0, 138.6, 132.5, 132.4, 129.4, 129.0, 128.9, 128.4, 128.0, 128.0, 127.9, 126.8, 126.0, 126.0, 125.8, 123.3, 119.1, 111.2, 60.1, 58.2, 21.8, 14.8. HRMS (ESI): m/z calculated for $C_{25}H_{22}NO_2^+$ [M-H]$^+$: 368.1650, found 368.1641.

4l - *ethyl 7-(adamantan-1-yl)-7H-acenaphthyleno[1,2-b]pyrrole-9-carboxylate.*

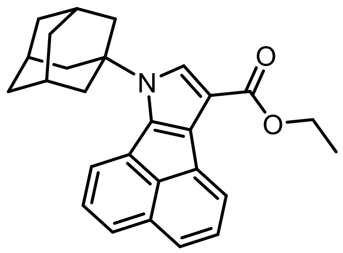

Following general procedure C, reaction for 12 h gave compound **4l** (40% yield) as a yellow solid; mp – 194-196 °C. **^{1}H NMR (500 MHz, CDCl$_3$)** δ 8.08 (d, J = 6.8 Hz, 1H), 7.72 (d, J = 7.1 Hz, 1H), 7.59 (dd, J = 8.2, 4.6 Hz, 2H), 7.50 – 7.38 (m, 3H), 4.35 (q, J = 7.1 Hz, 2H), 2.43 – 2.15 (m, 9H), 1.80 (d, J = 2.9 Hz, 6H), 1.40 (t, J = 7.1 Hz, 3H). **^{13}C NMR (126 MHz, CDCl$_3$)** δ 165.2, 136.5, 132.6, 132.4, 130.1, 130.0, 129.5, 127.9, 127.3, 126.7, 125.9, 125.8, 123.2, 121.7, 109.4, 59.9, 57.0, 41.3, 36.1, 29.6, 14.8. HRMS (ESI): m/z calculated for $C_{27}H_{28}NO_2^+$ $[M\text{-}H]^+$: 398.2120, found 398.2110.

4m - *ethyl 7-cyclohexyl-7H-acenaphthyleno[1,2-b]pyrrole-9-carboxylate.*

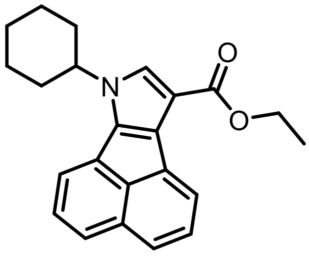

Following general procedure C, reaction for 1 h gave compound **4m** (73% yield) as a yellow solid; mp – 152-154 °C. **^{1}H NMR (500 MHz, CDCl$_3$)** δ 7.99 (dd, J = 6.9, 0.8 Hz, 1H), 7.58 (ddd, J = 10.8, 8.2, 0.8 Hz, 2H), 7.47 – 7.37 (m, 3H), 7.35 (s, 1H), 4.35 (q, J = 7.1 Hz, 2H), 4.14 (tt, J = 11.9, 3.8 Hz, 1H), 2.27 – 2.14 (m, 2H), 1.92 (dt, J = 13.9, 3.5 Hz, 2H), 1.72 (dtd, J = 24.9, 12.8, 3.4 Hz, 3H), 1.46 (qt, J = 13.2, 3.5 Hz, 2H), 1.40 (t, J = 7.1 Hz, 3H), 1.25 (qt, J = 13.1, 3.7 Hz, 1H).**^{13}C NMR (126 MHz, CDCl$_3$)** δ 165.1, 137.9, 132.8, 132.6, 129.5, 129.2, 128.1, 127.9, 126.8, 126.7, 126.0, 125.7, 123.1, 118.6, 110.9, 60.0, 58.7, 33.1, 25.7, 25.4, 14.8. HRMS (ESI): m/z calculated for $C_{23}H_{24}NO_2^+$ $[M\text{-}H]^+$: 346.1805, found 346.1798.

4n - ethyl 7-(2-azidoethyl)-7H-acenaphthyleno[1,2-b]pyrrole-9-carboxylate.

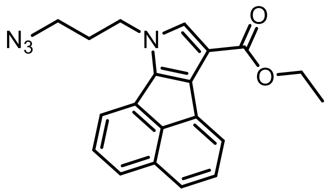

Following general procedure C, reaction for 12 h gave compound **4n** (92% yield) as a red solid; mp – 67-69 °C. **^{1}H NMR (600 MHz, CDCl$_3$)** δ 8.11 (d, J = 7.1 Hz, 1H), 7.71 (ddd, J = 11.1, 8.1, 0.8 Hz, 3H), 7.56 (dd, J = 8.3, 6.9 Hz, 1H), 7.54 (dd, J = 6.9, 0.8 Hz, 1H), 7.50 (dd, J = 8.0, 6.9 Hz, 1H), 4.46 (q, J = 7.1 Hz, 3H), 4.26 (t, J = 6.8 Hz, 3H), 3.39 (t, J = 6.2 Hz, 3H), 2.19 (p, J = 6.6 Hz, 3H), 1.51 (t, J = 7.1 Hz, 4H). **^{13}C NMR (151 MHz, CDCl$_3$)** δ 164.8, 138.2, 132.5, 132.5, 129.9, 129.5, 128.7, 128.4, 128.1, 126.9, 126.2, 125.8, 123.3, 118.3, 111.7, 60.1, 48.0, 46.1, 29.5, 14.7 HRMS (ESI): m/z calculated for $C_{20}H_{18}N_4O_2^+$ [M-H]$^+$: 347.1503, found 347.1502.

5a - *ethyl 3-[2-(4-methoxyphenyl)ethyl]-3-azatetracyclo-[11.4.0.0^{2,6}.0^{7,12}]-heptadeca-1(13),2(6),4,7(12),8,10,14,16-octaene-5-carboxylate.*

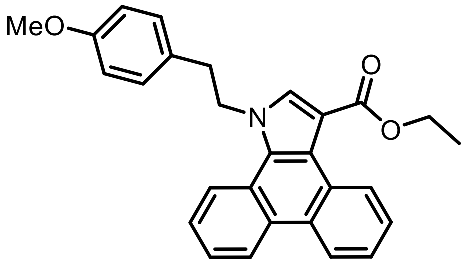

Following general procedure C, reaction for 2,5 h gave compound **5a** (90% yield) as a red solid; mp – 117-119 °C. **^{1}H NMR (250 MHz, CDCl$_3$)** δ 9.81 – 9.75 (m, 1H), 8.34 – 8.27 (m, 1H), 7.78 – 7.56 (m, 5H), 7.05 – 6.97 (m, 2H), 6.90 – 6.82 (m, 2H), 4.64 (dd, J = 8.4, 6.7 Hz, 2H), 4.43 (q, J = 7.1 Hz, 2H), 3.81 (s, 3H), 3.16 (dd, J = 8.4, 6.5 Hz, 2H), 1.46 (t, J = 7.1 Hz, 3H).**^{13}C NMR (63 MHz, CDCl$_3$)** δ 165.3, 158.7, 136.3, 129.7, 129.7, 129.2, 128.7, 128.5, 128.0, 127.4, 126.9, 126.6, 125.1, 124.8, 124.2, 123.6, 122.9, 121.7, 121.0, 114.2, 109.8, 60.1, 55.3, 53.3, 35.8, 14.6 HRMS (ESI): m/z calculated for $C_{28}H_{26}NO_3^+$ [M-H]$^+$: 424.1907, found 424.1905.

5b - *ethyl 3-[2-(3,4-dimethoxyphenyl)ethyl]-3-azatetracyclo-[11.4.0.0^{2,6}.0^{7,12}]-heptadeca-1(13),2(6),4,7(12),8,10,14,16-octaene-5-carboxylate.*

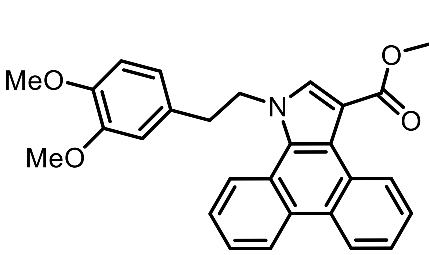

Following general procedure C, reaction for 3 h gave compound **5b** (84% yield) as a white solid; mp – 148-149 °C. **^{1}H NMR (250 MHz, DMSO-d$_6$)** δ 9.69 – 9.54 (m, 1H), 9.03 – 8.92 (m, 1H), 8.90 – 8.79 (m, 1H), 8.56 (d, J = 8.4 Hz, 1H), 8.03 (s, 1H), 7.87 – 7.51 (m, 4H), 6.86 (d, J = 8.0 Hz, 1H), 6.78 – 6.62 (m, 2H), 4.97 (t, J = 7.4 Hz, 2H), 4.32 (q, J = 7.1 Hz, 2H), 3.72 (s, 3H), 3.67 (s, 3H), 3.12 (t, J = 7.3 Hz, 2H), 1.34 (t, J = 7.1 Hz, 3H). **^{13}C NMR (63 MHz, DMSO-d$_6$)** δ 165.1, 149.2, 148.1, 138.0, 130.3, 129.2, 128.8, 128.3, 127.9, 127.6, 127.1, 127.1, 125.7, 125.6, 124.7, 123.8, 123.6, 122.0, 121.2, 121.0, 113.1, 112.4, 109.1, 60.1, 56.0, 55.8, 52.6, 35.9, 14.8. HRMS (ESI): m/z calculated for $C_{29}H_{28}NO_4^+$ [M-H]$^+$: 454.2013, found 454.2012.

5c - *ethyl 3-[2-(3,4-dihydroxyphenyl)ethyl]-3-azatetracyclo-[11.4.0.0^{2,6}.0^{7,12}]-heptadeca-1(13),2(6),4,7(12),8,10,14,16-octaene-5-carboxylate.*

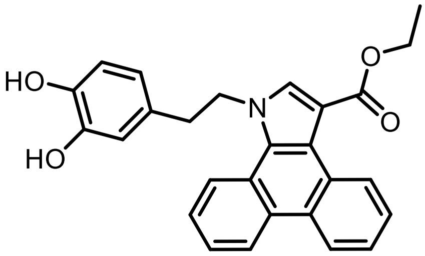

Following general procedure C, reaction for 2 h gave compound **5c** (50% yield) as a yellow solid; mp – 163-165 °C. **^{1}H NMR (600 MHz, DMSO-d$_6$)** δ 9.62 (dd, J = 8.1, 1.5 Hz, 1H), 8.98 – 8.91 (m, 1H), 8.88 – 8.81 (m, 2H), 8.79 (s, 1H), 8.57 – 8.50 (m, 1H), 8.10 (s, 1H), 7.79 (ddd, J = 8.3, 6.9, 1.2 Hz, 1H), 7.68 (ddd, J = 8.1, 6.9, 1.1 Hz, 1H), 7.62 (dd, J = 22.3, 8.3, 6.9, 1.4 Hz, 2H), 6.72 – 6.66 (m, 2H), 6.52 (dd, J = 8.0, 2.1 Hz, 1H), 4.89 (t, J = 7.8 Hz, 2H), 4.33 (q, J = 7.1 Hz, 2H), 3.03 (t, J = 7.8 Hz, 2H), 1.37 (t, J = 7.1 Hz, 3H). **^{13}C NMR (151 MHz, DMSO-d$_6$)** δ 165.2, 145.8, 144.5, 137.8, 129.2, 128.7, 128.4, 127.9, 127.6, 127.2, 127.1, 125.7, 125.6, 124.7, 123.7, 123.6, 121.9, 121.0, 119.9,

116.7, 116.1, 109.1, 60.1, 52.8, 35.9, 14.9. HRMS (ESI): m/z calculated for $C_{27}H_{24}NO_4^+$ [M-H]$^+$: 426.1700, found 426.1799.

5d - *ethyl 3-[2-(4-hydroxyphenyl)ethyl]-3-azatetracyclo-[11.4.0.0^{2,6}.0^{7,12}]-heptadeca-1(13),2(6),4,7(12),8,10,14,16-octaene-5-carboxylate.*

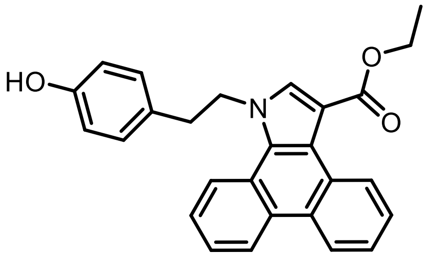

Following general procedure C, reaction for 2 h gave compound **5d** (75% yield) as a orange solid; mp – 166-168 °C. **^{1}H NMR (250 MHz, CDCl$_3$)** δ 9.71 (dd, J = 8.3, 1.4 Hz, 1H), 8.88 – 8.81 (m, 1H), 8.77 – 8.68 (m, 1H), 8.45 – 8.36 (m, 1H), 7.81 – 7.52 (m, 6H), 7.06 – 6.92 (m, 2H), 6.87 – 6.72 (m, 2H), 5.08 (s, 1H), 4.89 – 4.69 (m, 2H), 4.42 (q, J = 7.1 Hz, 2H), 3.22 (t, J = 7.4 Hz, 2H), 1.44 (t, J = 7.1 Hz, 3H). **^{13}C NMR (63 MHz, CDCl$_3$)** δ 165.5, 154.7, 136.4, 129.9, 129.7, 129.3, 128.8, 128.5, 128.0, 127.3, 126.9, 126.6, 125.2, 124.9, 124.3, 123.6, 122.9, 121.8, 121.0, 115.7, 109.9, 60.2, 53.4, 35.9, 14.5. HRMS (ESI): m/z calculated for $C_{27}H_{24}NO_3^+$ [M-H]$^+$: 410.1751, found 410.1749.

5e *- ethyl 3-[2-(5-methoxy-1H-indol-3-yl)ethyl]-3-azatetracyclo-[11.4.0.0^{2,6}.0^{7,12}]-heptadeca-1(13),2(6),4,7(12),8,10,14,16-octaene-5-carboxylate.*

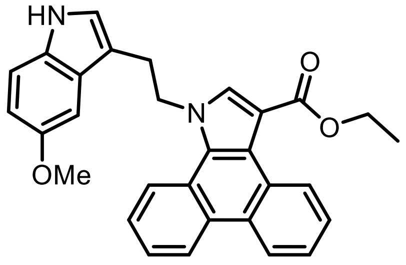

Following general procedure C, reaction for 1,5 h gave compound **5e** (80% yield) as a beige solid; mp – 146-148 °C. **^{1}H NMR (250 MHz, CDCl$_3$)** δ 9.74 (dd, J = 8.4, 1.4 Hz, 1H), 8.88 – 8.80 (m, 1H), 8.76 – 8.70 (m, 1H), 8.43 – 8.37 (m, 1H), 7.98 (s, 1H), 7.79 – 7.55 (m, 6H), 7.37 – 7.21 (m, 1H), 7.00 (d, J = 2.4 Hz, 1H), 6.92 (dd, J = 8.8, 2.4 Hz, 1H), 6.70 (d, J = 2.5 Hz, 1H), 4.82 (dd, J = 8.0, 6.5 Hz, 2H), 4.40 (q, J = 7.1 Hz, 2H), 3.84 (s, 3H), 3.40 (t, J = 7.2 Hz, 2H), 1.41 (t, J = 7.1 Hz, 3H).**^{13}C NMR (63 MHz, CDCl$_3$)** δ

165.5, 154.2, 136.5, 131.4, 129.7, 128.8, 128.5, 128.0, 127.5, 127.3, 126.9, 126.6, 125.1, 124.9, 124.2, 123.7, 123.2, 122.9, 121.7, 121.1, 112.4, 112.1, 111.0, 109.8, 100.3, 60.1, 55.9, 52.1, 26.5, 14.5. HRMS (ESI): m/z calculated for $C_{30}H_{26}N_2O_3^+$ [M-H]$^+$: 463.2016, found 463.2015.

5f - *ethyl 3-(prop-2-en-1-yl)-3-azatetracyclo-[11.4.0.0^{2,6}.0^{7,12}]-heptadeca-1(13),2(6),4,7(12),8,10,14,16-octaene-5-carboxylate.*

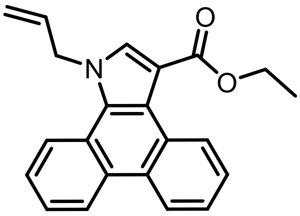

Following general procedure C, reaction for 1,5 h gave compound **5f** (78%) as a white solid; mp – 120-121 °C. **^{1}H NMR (250 MHz, CDCl$_3$)** δ 9.77 – 9.70 (m, 1H), 8.82 (dt, J = 6.4, 3.6 Hz, 1H), 8.75 – 8.67 (m, 1H), 8.31 – 8.17 (m, 1H), 7.88 (s, 1H), 7.70 (ddd, J = 8.4, 6.9, 1.5 Hz, 1H), 7.65 – 7.56 (m, 3H), 6.27 (ddt, J = 17.2, 10.5, 4.2 Hz, 1H), 5.37 (ddt, J = 10.5, 1.8, 1.2 Hz, 1H), 5.25 (dt, J = 4.1, 2.0 Hz, 2H), 5.09 (ddt, J = 17.2, 2.5, 1.2 Hz, 1H), 4.46 (q, J = 7.1 Hz, 2H), 1.48 (t, J = 7.1 Hz, 3H). **^{13}C NMR (63 MHz, CDCl$_3$)** δ 165.4, 136.0, 132.6, 129.7, 129.5, 128.4, 128.0, 127.3, 126.9, 126.4, 125.1, 124.9, 124.0, 123.3, 122.8, 121.6, 121.2, 118.3, 110.6, 60.2, 53.3, 14.6. HRMS (ESI): m/z calculated for $C_{22}H_{20}NO_2^+$ [M-H]$^+$: 330.1489, found 330.1487.

5g - *ethyl 3-(prop-2-yn-1-yl)-3-azatetracyclo-[11.4.0.0^{2,6}.0^{7,12}]-heptadeca-1(13),2(6),4,7(12),8,10,14,16-octaene-5-carboxylate.*

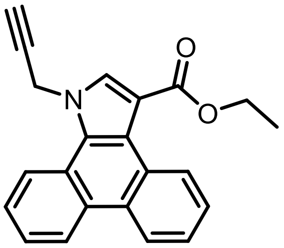

Following general procedure C, reaction for 2 h gave compound **5g** (73% yield) as a white solid; mp – 127-128 °C. **^{1}H NMR (250 MHz, CDCl$_3$)** δ 9.74 – 9.65 (m, 1H), 8.93 – 8.77 (m, 1H), 8.75 – 8.66 (m, 1H), 8.53 – 8.39 (m, 1H), 7.99 (s, 1H), 7.88 – 7.54 (m, 4H), 5.32 (d, J = 2.5 Hz, 2H), 4.45 (q, J = 7.1 Hz, 2H), 1.48 (t, J = 7.1 Hz, 3H). **^{13}C**

NMR (63 MHz, CDCl3) δ 165.2, 135.2, 129.8, 129.3, 128.3, 128.0, 127.3, 126.9, 126.5, 125.3, 125.1, 124.0, 123.2, 122.8, 121.6, 121.4, 111.0, 77.2, 76.0, 60.3, 41.2, 14.6. HRMS (ESI): m/z calculated for $C_{22}H_{18}NO_2^+$ [M-H]$^+$: 328.1332, found 328.1331.

5h - *ethyl 3-phenyl-3-azatetracyclo-[11.4.0.0^{2,6}.0^{7,12}]-heptadeca-1(13),2(6),4,7(12),8,10,14,16-octaene-5-carboxylate.*

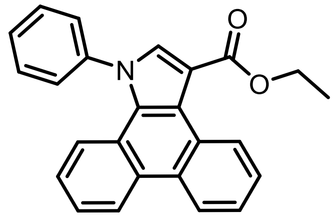

Following general procedure C, reaction for 14 h gave compound **5h** (5% yield) as a yellow oil. **^{1}H NMR (500 MHz, CDCl3)** δ 9.67 (dd, J = 8.2, 1.4 Hz, 1H), 8.65 (dd, J = 12.0, 8.3 Hz, 2H), 7.85 (s, 1H), 7.63 (ddd, J = 8.2, 6.9, 1.3 Hz, 1H), 7.57 – 7.51 (m, 4H), 7.48 – 7.38 (m, 3H), 7.23 (dd, J = 8.4, 1.3 Hz, 1H), 7.14 (ddd, J = 8.3, 6.9, 1.2 Hz, 1H), 4.37 (q, J = 7.1 Hz, 2H), 1.36 (t, J = 7.1 Hz, 3H). **^{13}C NMR (126 MHz, CDCl3)** δ 165.4, 141.4, 136.3, 130.2, 130.0, 129.8, 129.2, 128.3, 128.3, 127.4, 127.2, 127.0, 125.8, 125.3, 125.1, 123.8, 123.1, 122.9, 121.8, 120.9, 111.8, 60.3, 14.5. HRMS (ESI): m/z calculated for $C_{25}H_{19}NO_2^+$ [M-H]$^+$: 366.1493, found 366.1485.

5j - *ethyl 3-cyclopropyl-3-azatetracyclo[11.4.0.0^{2,6}.0^{7,12}]heptadeca-1(13),2(6),4,7(12),8,10,14,16-octaene-5-carboxylate*

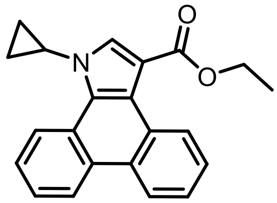

Following general procedure C, reaction for 5 h gave compound **5j** (60% yield) as a white solid; mp – 142-143 °C. **^{1}H NMR (250 MHz, CDCl3)** δ 9.71 (dd, J = 8.4, 1.3 Hz, 1H), 9.13 – 9.01 (m, 1H), 8.89 – 8.76 (m, 1H), 8.72 (dd, J = 8.2, 1.5 Hz, 1H), 7.95 (s, 1H), 7.74 – 7.57 (m, 5H), 4.46 (q, J = 7.1 Hz, 3H), 3.90 (ddd, J = 9.3, 7.0, 4.0 Hz, 1H), 1.49 (t, J = 7.1 Hz, 4H), 1.42 – 1.24 (m, 5H). **^{13}C NMR (63 MHz, CDCl3)** δ 165.4, 135.1, 130.9, 129.6, 128.4, 127.9, 127.3, 126.8, 126.1, 125.1, 124.9, 123.9, 123.8, 122.8, 122.3,

120.8, 110.0, 60.2, 33.1, 14.6, 10.1. HRMS (ESI): m/z calculated for $C_{22}H_{19}NO_2^+$ [M-H]$^+$: 330.1489, found 330.1487.

5k - *ethyl 3-(1-phenylethyl)-3-azatetracyclo-[11.4.0.0^{2,6}.0^{7,12}]-heptadeca-1(13),2(6),4,7(12),8,10,14,16-octaene-5-carboxylate.*

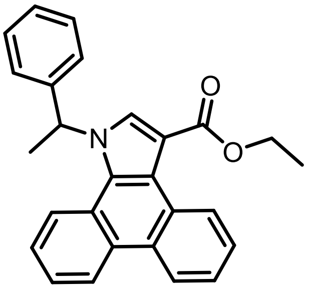

Following general procedure C, reaction for 5 h gave compound **5k** (60%) as a yellow solid; mp – 165-166 °C. **^{1}H NMR (250 MHz, $CDCl_3$)** δ 9.76 (dd, J = 8.2, 1.4 Hz, 1H), 8.78 (dd, J = 8.4, 1.4 Hz, 1H), 8.71 (dd, J = 8.4, 1.4 Hz, 1H), 8.24 – 8.13 (m, 2H), 7.91 – 7.10 (m, 9H), 6.44 (q, J = 6.9 Hz, 1H), 4.50 (q, J = 7.1 Hz, 2H), 2.09 (d, J = 7.0 Hz, 3H), 1.51 (t, J = 7.1 Hz, 3H). **^{13}C NMR (63 MHz, $CDCl_3$)** δ 165.6, 142.2, 132.1, 130.3, 129.9, 129.2, 128.4, 128.1, 127.8, 127.3, 126.9, 126.3, 125.8, 125.2, 124.8, 124.0, 123.4, 122.9, 122.0, 121.2, 110.9, 60.3, 58.5, 24.1, 14.6. HRMS (ESI): m/z calculated for $C_{27}H_{24}NO_2^+$ [M-H]$^+$: 394.1807, found 394.1798.

5m - *ethyl 3-cyclohexyl-3-azatetracyclo-[11.4.0.0^{2,6}.0^{7,12}]-heptadeca-1(13),2(6),4,7(12),8,10,14,16-octaene-5-carboxylate.*

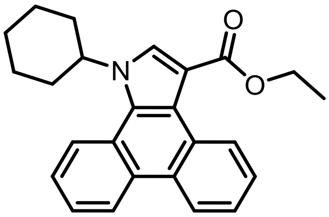

Following general procedure C, reaction for 12 h gave compound **5m** (95% yield) as a white solid; mp – 161-163 °C. **^{1}H NMR (500 MHz, $CDCl_3$)** δ 9.58 (dd, J = 8.3, 1.4 Hz, 1H), 8.73 (dd, J = 8.3, 1.5 Hz, 1H), 8.60 (dd, J = 8.4, 1.3 Hz, 1H), 8.23 – 8.11 (m, 1H), 7.99 (s, 1H), 7.67 – 7.41 (m, 5H), 4.85 (tt, J = 11.7, 3.3 Hz, 1H), 4.36 (q, J = 7.1 Hz, 2H), 2.38 – 2.30 (m, 2H), 2.02 – 1.93 (m, 2H), 1.78 (qd, J = 12.6, 3.4 Hz, 3H), 1.55 (qt, J = 13.3, 3.5 Hz, 2H), 1.31 (qt, J = 13.2, 3.9 Hz, 1H). **^{13}C NMR (126 MHz, $CDCl_3$)** δ

165.6, 131.0, 129.9, 129.5, 128.5, 127.9, 127.3, 126.8, 126.5, 125.0, 124.7, 124.3, 123.8, 122.8, 121.4, 121.1, 110.3, 60.2, 58.8, 34.6, 26.0, 25.6, 14.6. HRMS (ESI): m/z calculated for $C_{25}H_{26}NO_2^+$ [M-H]$^+$: 372.1963, found 372.1955.

5n - *ethyl 3-(3-azidopropyl)-3-azatetracyclo[11.4.0.0^{2,6}.0^{7,12}]heptadeca-1(13),2(6),4,7(12),8,10,14,16-octaene-5-carboxylate.*

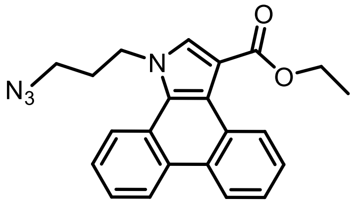

Following general procedure C, reaction for 12 h gave compound **5n** (50% yield) as a beige solid; mp – 88-90 °C. **^{1}H NMR (250 MHz, $CDCl_3$)** δ 9.74 (dd, J = 8.4, 1.3 Hz, 1H), 8.75 (dt, J = 7.1, 3.5 Hz, 1H), 8.71 – 8.64 (m, 1H), 8.15 – 8.04 (m, 1H), 7.78 – 7.66 (m, 3H), 7.66 – 7.51 (m, 4H), 4.60 – 4.31 (m, 5H), 3.25 (t, J = 6.2 Hz, 3H), 2.17 – 2.04 (m, 3H), 1.49 (t, J = 7.1 Hz, 4H). **^{13}C NMR (63 MHz, $CDCl_3$)** δ 165.2, 136.3, 129.6, 128.6, 128.4, 127.9, 127.4, 126.9, 126.6, 125.2, 124.9, 124.1, 123.3, 122.8, 121.7, 120.8, 110.2, 60.2, 48.4, 48.1, 29.2, 14.6. HRMS (ESI): m/z calculated for $C_{22}H_{20}N_4O_2^+$ [M-H]$^+$: 373.1659, found 373.1658.

5o – Ethyl 1-(2-(1H-indole-3-yl)ethyl)-1H-dibenzo[e,g]indole-3-carboxilate.

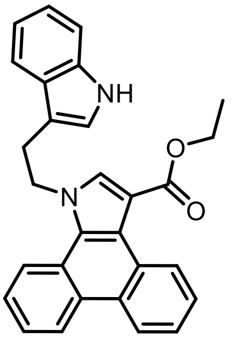

Following general procedure C, reaction for 4 h gave compound **5o** (64% yield) as a beige solid; mp – 181-183 °C. **^{1}H NMR (250 MHz, $CDCl_3$)** δ 9.73 (dd, J = 8.1, 1.6 Hz, 1H), 8.93 – 8.83 (m, 1H), 8.82 – 8.71 (m, 1H), 8.51 – 8.43 (m, 1H), 8.05 (s, 1H), 7.76 – 7.58 (m, 7H), 7.46 – 7.39 (m, 1H), 7.34 – 7.16 (m, 3H), 6.78 (d, J = 2.3 Hz, 1H), 4.92 (t, J = 7.3 Hz, 2H), 4.39 (q, J = 7.1 Hz, 2H), 3.48 (t, J = 7.3 Hz, 2H), 1.64 (s, 2H), 1.40 (t, J = 7.1 Hz, 3H). **^{13}C NMR (63 MHz, $CDCl_3$)** δ 165.4, 136.5, 136.3, 129.7, 128.8, 128.5,

128.0, 127.3, 127.0, 126.9, 126.7, 125.1, 124.9, 124.2, 123.7, 122.9, 122.5, 122.4, 121.7, 121.1, 119.8, 118.4, 111.4, 111.3, 109.8, 60.1, 52.2, 26.5, 14.5. HRMS (ESI): m/z calculated for $C_{29}H_{24}N_2O_2Na^+$ [M-Na]$^+$: 455.1730, found 455.1716.

6a - *ethyl 7-[(1-benzyl-1H-1,2,3-triazol-4-yl)methyl]-7H-acenaphthyleno[1,2-b]pyrrole-9-carboxylate.*

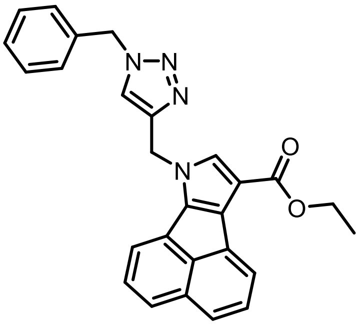

Following general procedure D, reaction for 12 h gave compound **6a** (90% yield) as a yellow solid; mp – 133-135 °C. **^{1}H NMR (500 MHz, CDCl$_3$)** δ 7.99 (d, J = 6.8 Hz, 1H), 7.62 – 7.55 (m, 2H), 7.44 (dd, J = 8.2, 6.9 Hz, 1H), 7.34 – 7.26 (m, 3H), 7.23 – 7.14 (m, 4H), 7.04 (dd, J = 7.4, 2.2 Hz, 2H), 5.37 (s, 2H), 5.32 (s, 2H), 4.33 (q, J = 7.1 Hz, 2H), 1.38 (t, J = 7.2 Hz, 3H). **^{13}C NMR (126 MHz, CDCl$_3$)** δ 164.8, 144.0, 138.3, 134.2, 132.3, 130.1, 129.4, 129.1, 128.8, 128.6, 128.4, 128.0, 127.9, 127.0, 126.2, 125.9, 123.4, 122.1, 119.0, 111.9, 60.2, 54.3, 45.2, 14.7. HRMS (ESI): m/z calculated for $C_{27}H_{23}N_4O_2^+$ [M-H]$^+$: 435.1821, found 435.1813.

6b - *ethyl 3-[(1-benzyl-1H-1,2,3-triazol-4-yl)methyl]-3-azatetracyclo[11.4.0.0^{2,6}.0^{7,12}]heptadeca-1(13),2(6),4,7(12),8,10,14,16-octaene-5-carboxylate.*

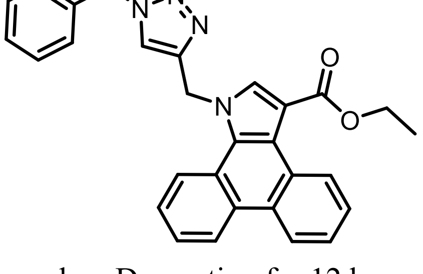

Following general procedure D, reaction for 12 h gave compound **6b** (74% yield) as a gray solid; mp – 180-181 °C. **^{1}H NMR (400 MHz, DMSO-d$_6$)** δ 9.61 (dd, J = 8.0, 1.7 Hz, 1H), 8.92 – 8.86 (m, 1H), 8.85 – 8.76 (m, 1H), 8.51 – 8.43 (m, 1H), 8.37 (s, 1H),

8.02 (s, 1H), 7.74 – 7.51 (m, 4H), 7.34 – 7.21 (m, 3H), 7.10 (dd, J = 6.6, 3.1 Hz, 2H), 6.11 (s, 2H), 5.51 (s, 2H), 4.36 (q, J = 7.1 Hz, 2H), 1.38 (t, J = 7.1 Hz, 3H). **^{13}C NMR (101 MHz, DMSO-d$_6$)** δ 165.2, 144.2, 138.1, 136.5, 129.2, 129.2, 129.1, 128.4, 128.2, 128.0, 127.9, 127.2, 127.1, 127.1, 125.7, 125.7, 124.3, 124.1, 123.7, 123.2, 122.5, 120.9, 109.8, 60.3, 53.2, 47.1, 14.9. HRMS (ESI): m/z calculated for $C_{29}H_{25}N_4O_2^+$ [M-H]$^+$: 461.1979, found 461.1969.

7a - *ethyl 7-[3-(4-phenyl-1H-1,2,3-triazol-1-yl)propyl]-7H-acenaphthyleno[1,2-b]pyrrole-9-carboxylate.*

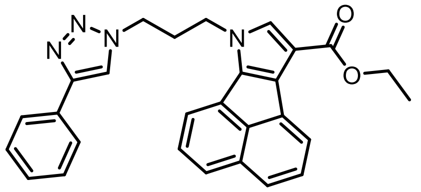

Following general procedure D, reaction for 12 h gave compound **7a** (90% yield) as a yellow solid; mp – 110-112 °C. **^{1}H NMR (250 MHz, CDCl$_3$)** δ 8.12 (dd, J = 6.9, 0.9 Hz, 1H), 7.81 – 7.66 (m, 5H), 7.57 (dd, J = 8.3, 6.8 Hz, 1H), 7.45 – 7.35 (m, 5H), 7.28 (s, 1H), 4.45 (q, J = 7.2 Hz, 4H), 4.35 (t, J = 6.4 Hz, 2H), 2.72 (p, J = 5.8 Hz, 2H), 1.50 (t, J = 7.1 Hz, 3H). **^{13}C NMR (63 MHz, DMSO-d$_6$)** δ 164.3, 147.0, 138.2, 132.5, 132.0, 131.6, 131.3, 129.5, 129.3, 128.5, 128.5, 128.3, 127.8, 127.7, 126.6, 126.3, 125.6, 123.2, 122.0, 119.8, 110.9, 60.0, 47.4, 46.3, 30.9, 15.0. HRMS (ESI): m/z calculated for $C_{28}H_{24}N_4O_2^+$ [M-H]$^+$: 449.1972, found 449.1970.

7b - *ethyl 3-[3-(4-phenyl-1H-1,2,3-triazol-1-yl)propyl]-3-azatetracyclo[11.4.0.0^{2,6}.0^{7,12}]heptadeca-1(13),2(6),4,7(12),8,10,14,16-octaene-5-carboxylate.*

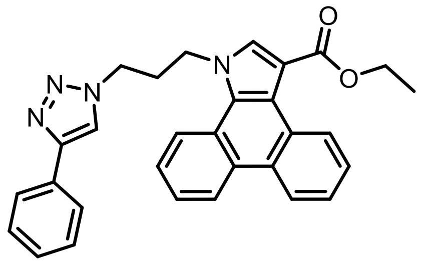

Following general procedure D, reaction for 9 h gave compound **7b** (82% yield) as a white solid; mp – 206-207 °C. **^{1}H NMR (600 MHz, DMSO-d$_6$)** δ 9.60 (dd, J = 8.2, 1.5 Hz, 1H), 8.97 – 8.87 (m, 1H), 8.82 (dd, J = 8.4, 1.5 Hz, 1H), 8.65 (s, 1H), 8.27 – 8.18

(m, 2H), 7.86 (dd, J = 8.2, 1.3 Hz, 2H), 7.69 – 7.54 (m, 5H), 7.50 – 7.41 (m, 2H), 7.40 – 7.29 (m, 1H), 4.87 (t, J = 7.4 Hz, 2H), 4.59 (t, J = 6.8 Hz, 2H), 4.34 (q, J = 7.1 Hz, 2H), 2.54 (p, J = 6.3, 5.8 Hz, 2H), 1.38 (t, J = 7.1 Hz, 3H). **^{13}C NMR (151 MHz, DMSO-d$_6$)** δ 165.1, 147.0, 137.7, 131.3, 129.4, 129.2, 128.7, 128.4, 128.2, 127.9, 127.6, 127.1, 127.1, 125.7, 125.7, 125.6, 124.6, 123.7, 123.4, 122.2, 121.7, 121.0, 109.6, 60.3, 48.6, 47.4, 31.0, 14.9. HRMS (ESI): m/z calculated for $C_{30}H_{27}N_4O_2^+$ [M-H]$^+$: 475.2129, found 475.2131.

8a - *ethyl 3,13-diazaheptacyclo[16.7.1.0^{2,17}.0^{3,15}.0^{6,14}.0^{7,12}.0^{22,26}]hexacosa-1(26),2(17),7(12),8,10,15,18,20,22,24-decaene-16-carboxylate.*

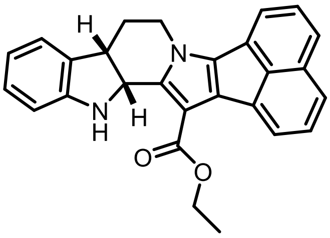

Following general procedure E, reaction for 15 minutes gave compound **8a** (94% yield) as a orange solid; mp – 221-223 °C. **^{1}H NMR (500 MHz, Benzene-d$_6$)** δ 8.36 (d, J = 6.8 Hz, 1H), 7.56 (dd, J = 11.8, 8.2 Hz, 2H), 7.50 (dd, J = 8.2, 6.8 Hz, 1H), 7.28 (dd, J = 8.2, 6.9 Hz, 1H), 7.08 – 6.97 (m, 4H), 6.84 – 6.75 (m, 1H), 6.47 (d, J = 7.6 Hz, 1H), 5.73 (s, 1H), 5.25 (d, J = 8.1 Hz, 1H), 4.31 (q, J = 7.2 Hz, 2H), 3.50 (dt, J = 12.3, 4.8 Hz, 1H), 3.12 (ddd, J = 12.3, 9.8, 4.0 Hz, 1H), 2.97 (ddd, J = 10.0, 8.0, 4.3 Hz, 1H), 1.80 (dtd, J = 14.3, 9.9, 4.4 Hz, 1H), 1.45 – 1.38 (m, 1H), 1.20 (t, J = 7.1 Hz, 3H). **^{13}C NMR (126 MHz, C_6D_6)** δ 165.6, 150.8, 139.7, 136.7, 133.6, 132.8, 130.4, 129.7, 129.4, 128.4, 126.6, 125.9, 125.8, 123.6, 118.4, 118.1, 109.7, 108.7, 59.9, 55.7, 42.2, 38.1, 25.1, 14.4. HRMS (ESI): m/z calculated for $C_{27}H_{23}N_2O_2^+$ [M-H]$^+$: 407.1754, found 407.1749.

8b – *Ethyl 2,12-diazaheptacyclo[14.12.0.0^{2,14}.0^{5,13}.0^{6,11}.0^{17,22}.0^{23,28}]octacosa-1(16),6(11),7,9,14,17(22),18,20,23(28),24,26-undecaene-15-carboxylate.*

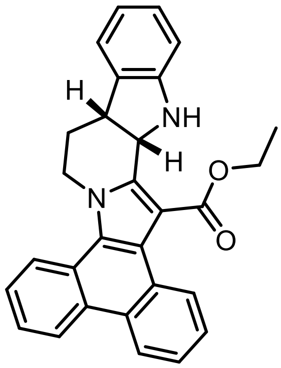

Following general procedure E, reaction for 3 h gave compound **8b** (79% yield) as a beige solid; mp – 210-212 °C. **^{1}H NMR (500 MHz, CDCl$_3$)** δ 8.70 (dt, J = 8.1, 2.3 Hz, 1H), 8.60 (dd, J = 7.9, 1.7 Hz, 1H), 8.39 – 8.22 (m, 1H), 7.55 – 7.45 (m, 2H), 7.17 (s, 0H), 7.03 (td, J = 7.6, 1.3 Hz, 1H), 6.73 (td, J = 7.4, 1.0 Hz, 1H), 6.62 (d, J = 7.7 Hz, 0H), 5.29 (s, 1H), 5.24 (d, J = 8.2 Hz, 1H), 4.68 (ddd, J = 11.1, 6.2, 4.5 Hz, 1H), 4.60 – 4.48 (m, 1H), 4.43 (dq, J = 10.8, 7.2 Hz, 1H), 3.64 (td, J = 8.7, 4.1 Hz, 1H), 2.32 (dtd, J = 13.4, 8.7, 4.4 Hz, 1H), 2.16 (ddt, J = 14.3, 6.2, 4.2 Hz, 1H), 1.42 (t, J = 7.1 Hz, 2H). **^{13}C NMR (126 MHz, CDCl$_3$)** δ 166.9, 150.3, 140.3, 130.1, 129.9, 129.3, 128.4, 128.2, 127.7, 126.3, 126.3, 126.3, 124.9, 124.8, 124.2, 123.8, 123.6, 123.2, 122.0, 119.6, 118.8, 109.8, 109.4, 60.9, 56.3, 46.2, 38.2, 26.4, 14.4. HRMS (ESI): m/z calculated for $C_{29}H_{25}N_2O_2^+$ [M-H]$^+$: 433.1910, found 433.1896.

9 – *ethyl 2,12-diazaheptacyclo[14.12.0.0^{2,14}.0^{5,13}.0^{6,11}.0^{17,22}.0^{23,28}]octacosa-1(16),3,5(13),6(11),7,9,14,17,19,21,23(28),24,26-tridecaene-15-carboxylate.*

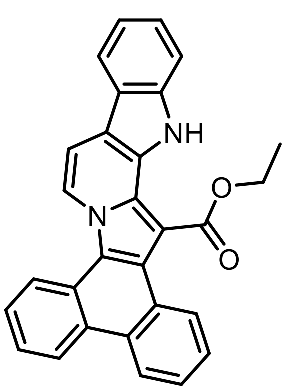

Following general procedure F, reaction for 12 h gave compound **9** (74% yield) as a dark green solid; mp – 184-186 °C. **^{1}H NMR (500 MHz, TFA – d)** δ 9.89 (d, J = 6.4 Hz, 1H), 9.17 – 9.04 (m, 1H), 9.01 – 8.90 (m, 1H), 8.89 – 8.83 (m, 1H), 8.80 (d, J = 6.4 Hz, 1H), 8.49 (d, J = 8.1 Hz, 1H), 8.22 – 8.17 (m, 1H), 8.09 – 7.88 (m, 6H), 7.68 (ddd, J = 7.9, 5.9, 1.8 Hz, 1H), 4.55 (dq, J = 10.7, 7.0 Hz, 1H), 4.37 (dq, J = 10.8, 7.0 Hz, 1H), 1.25 (t, J = 7.0 Hz, 3H). **^{13}C NMR (126 MHz, CF_3CO_2D)** δ 168.3, 145.2, 135.9, 134.5, 134.3, 134.0, 133.1, 132.4, 131.2, 129.6, 128.9, 128.7, 127.2, 126.7, 125.9, 124.7, 124.5, 123.5, 123.5, 122.8, 120.6, 120.4, 119.7, 116.6, 112.8, 65.5, 11.9. HRMS (ESI): m/z calculated for $C_{29}H_{21}N_2O_2^+$ $[M-H]^+$: 429.1598, found 429.1583.

NMR Spectra

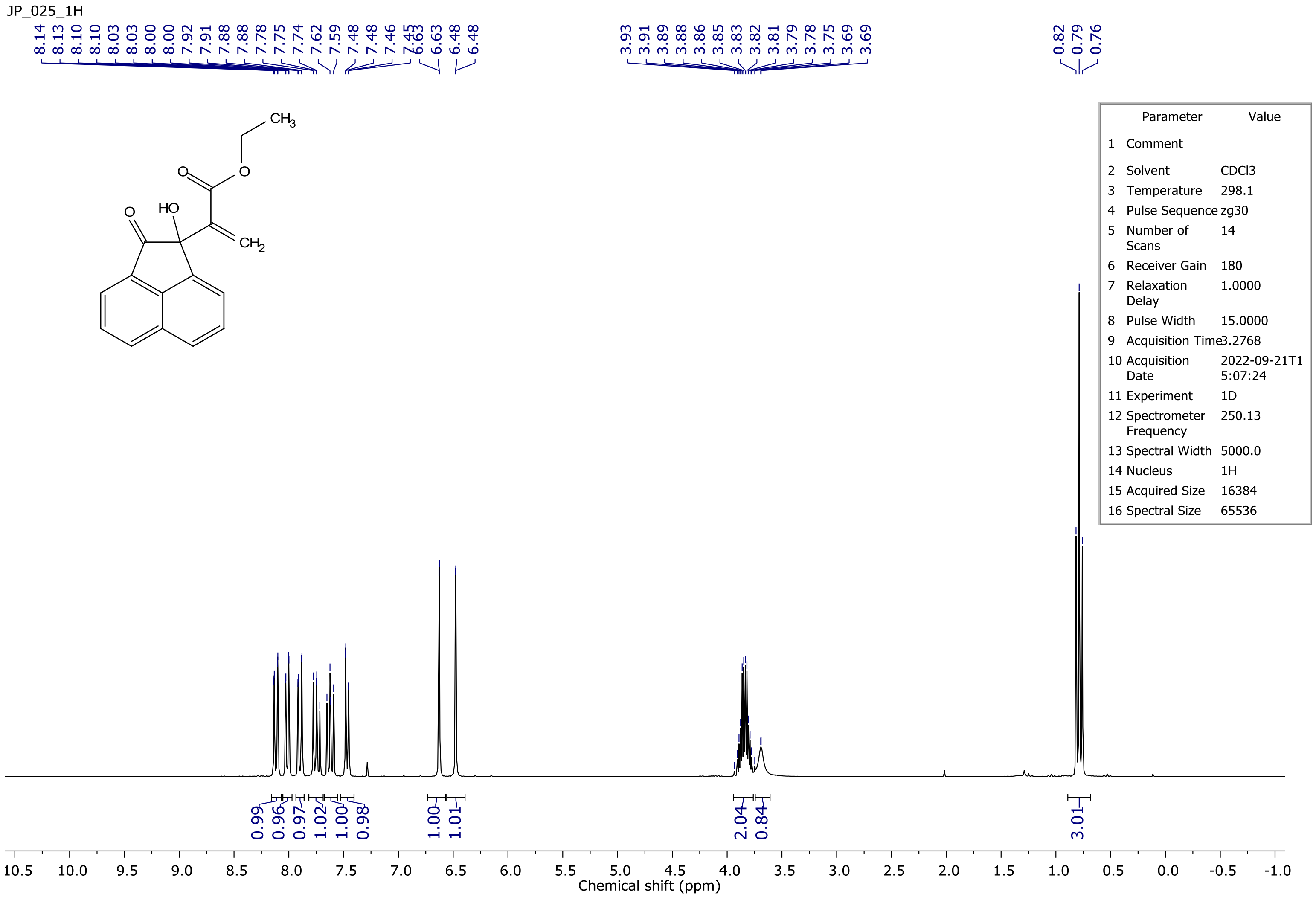

Figure S1 – ^{1}H NMR, 250 Hz, $CDCl_3$ (compound **2a**).

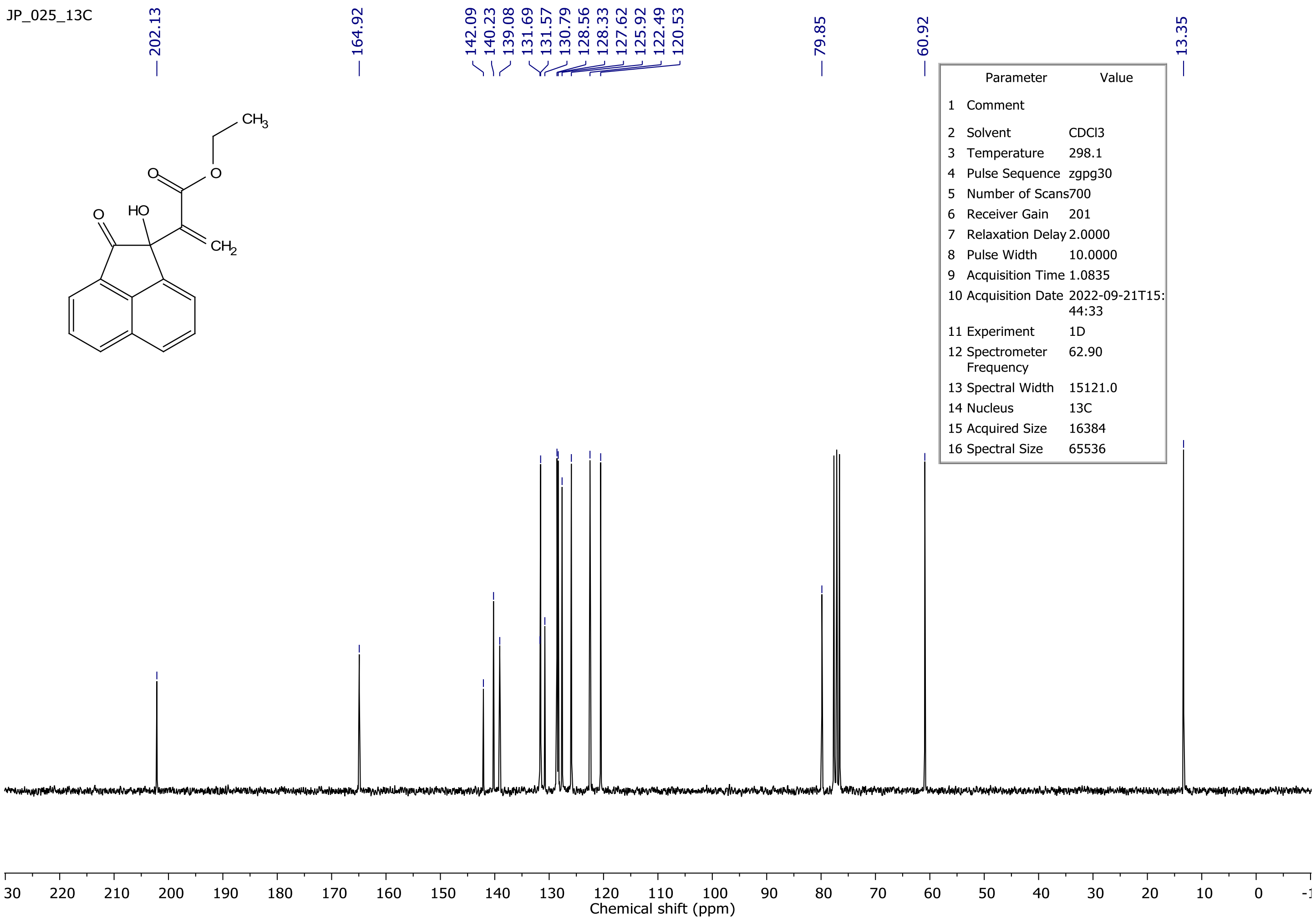

Figure S2 – ^{13}C NMR, 63 Hz, $CDCl_3$ (compound **2a**).

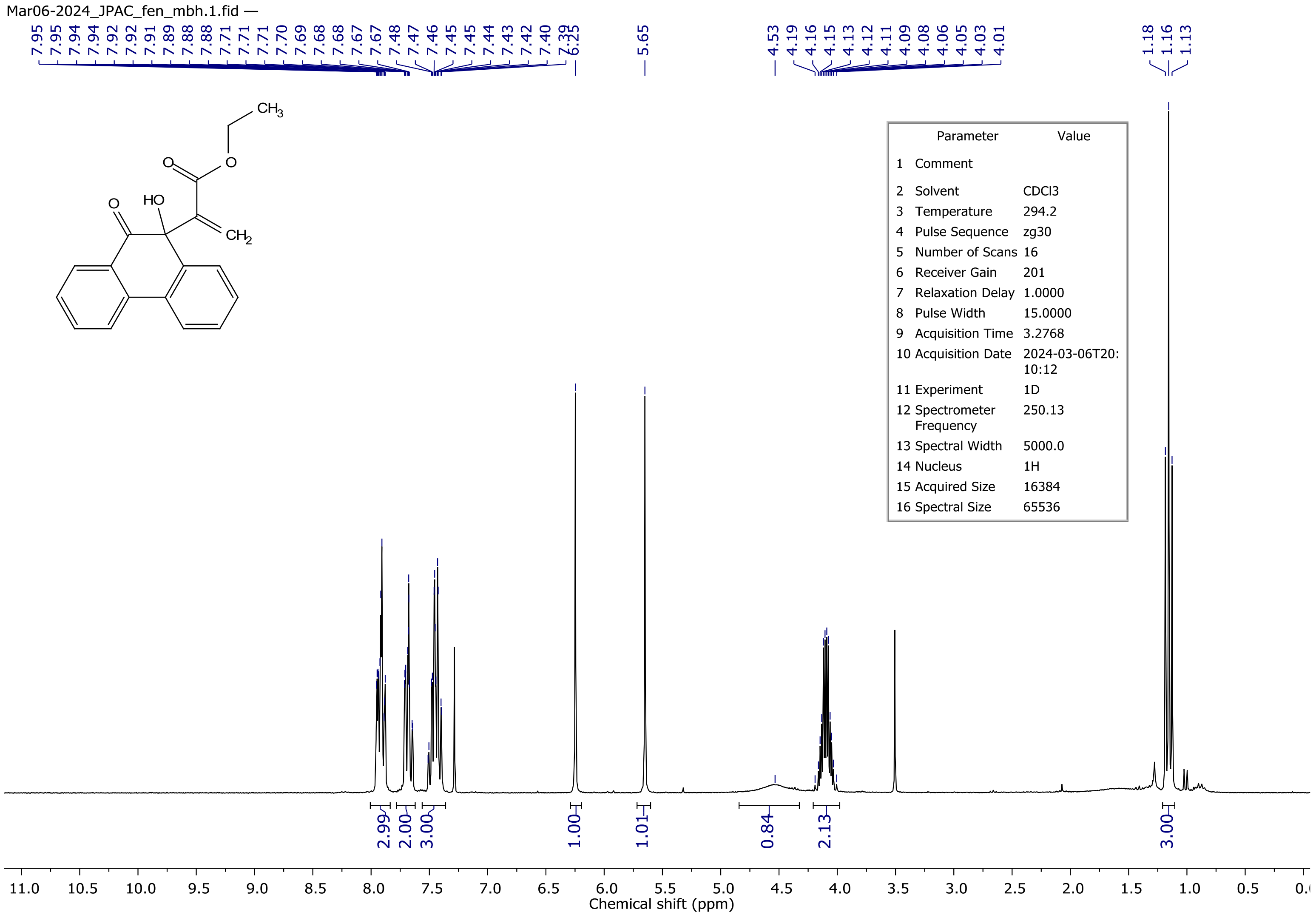

Figure S3 – ^{1}H NMR, 250 Hz, $CDCl_3$ (compound **2b**).

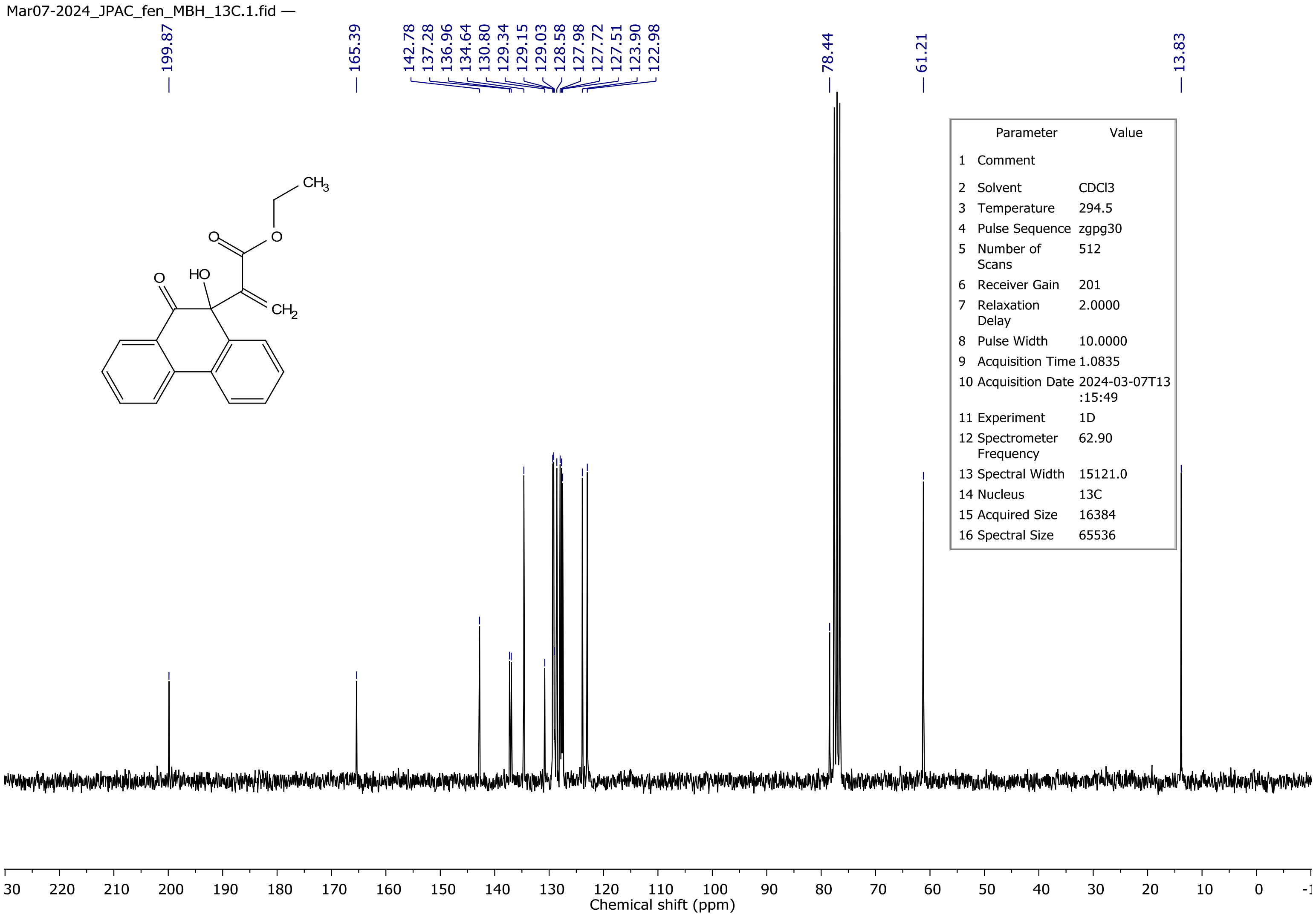

Figure S4 – ^{13}C NMR, 63 Hz, $CDCl_3$ (compound **2b**).

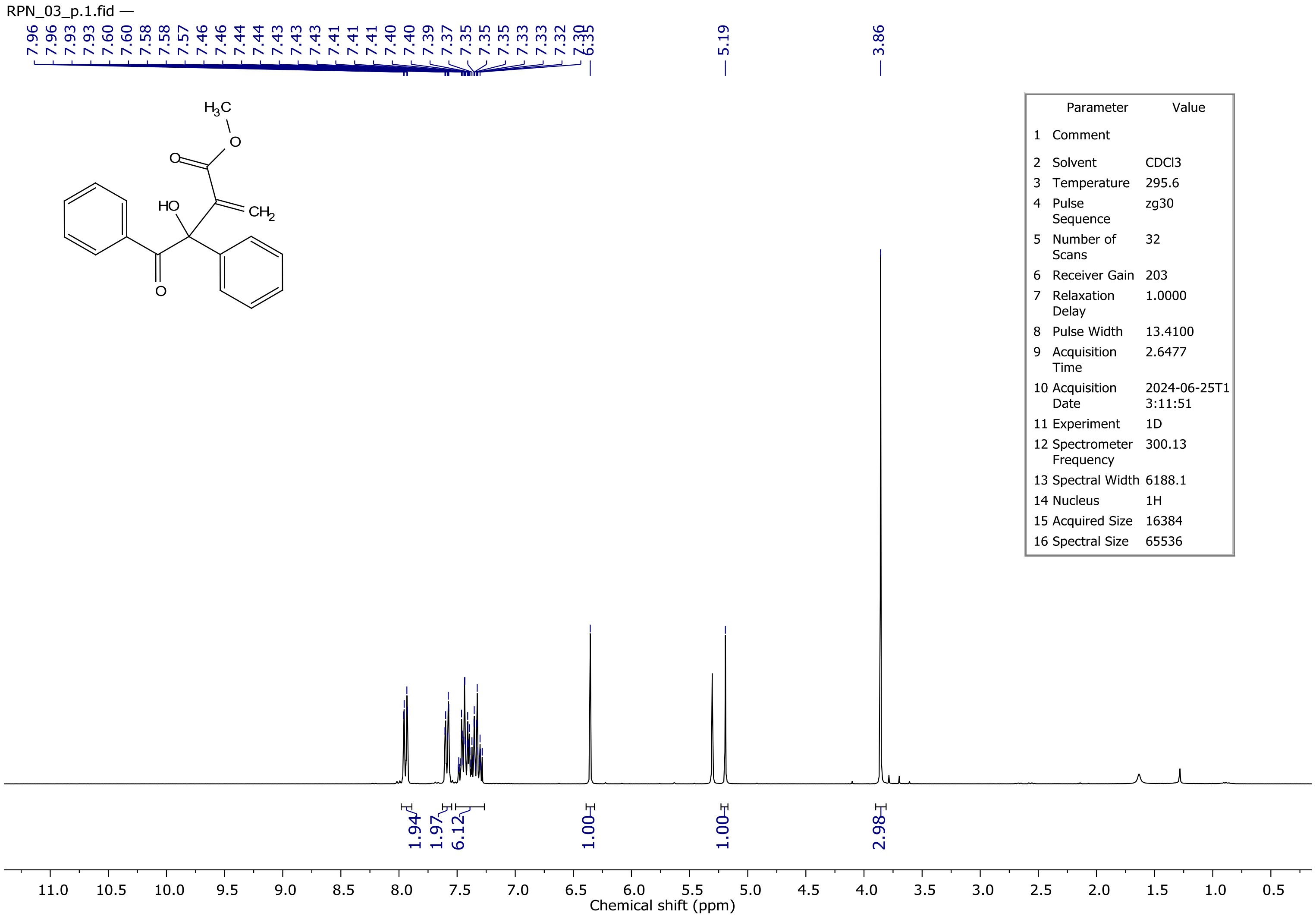

	Parameter	Value
1	Comment	
2	Solvent	CDCl3
3	Temperature	295.6
4	Pulse Sequence	zg30
5	Number of Scans	32
6	Receiver Gain	203
7	Relaxation Delay	1.0000
8	Pulse Width	13.4100
9	Acquisition Time	2.6477
10	Acquisition Date	2024-06-25T13:11:51
11	Experiment	1D
12	Spectrometer Frequency	300.13
13	Spectral Width	6188.1
14	Nucleus	1H
15	Acquired Size	16384
16	Spectral Size	65536

Figure S5 – ^{1}H NMR, 250 Hz, $CDCl_3$ (compound **2d**).

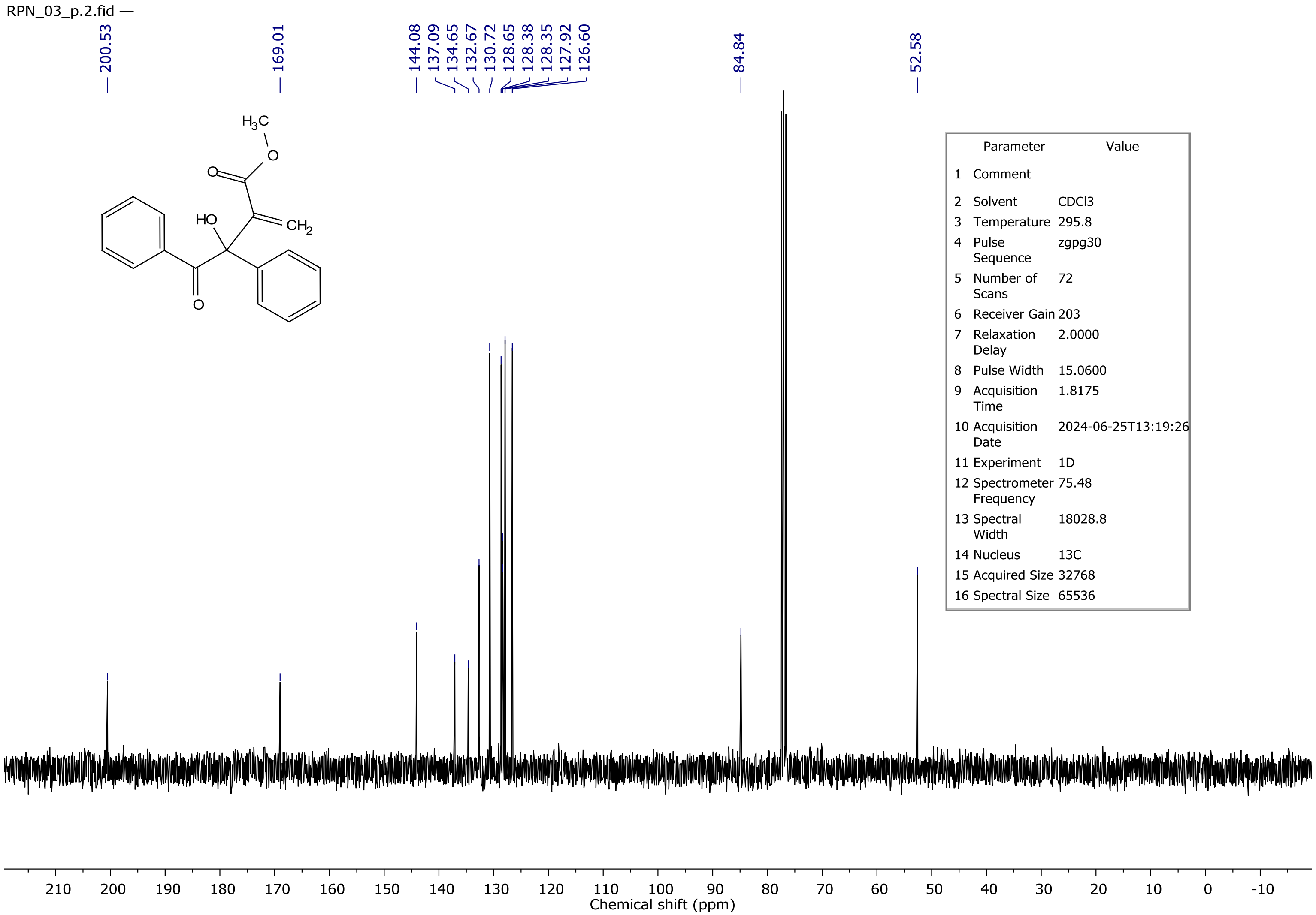

Figure S6 – ^{13}C NMR, 63 Hz, $CDCl_3$ (compound **2d**).

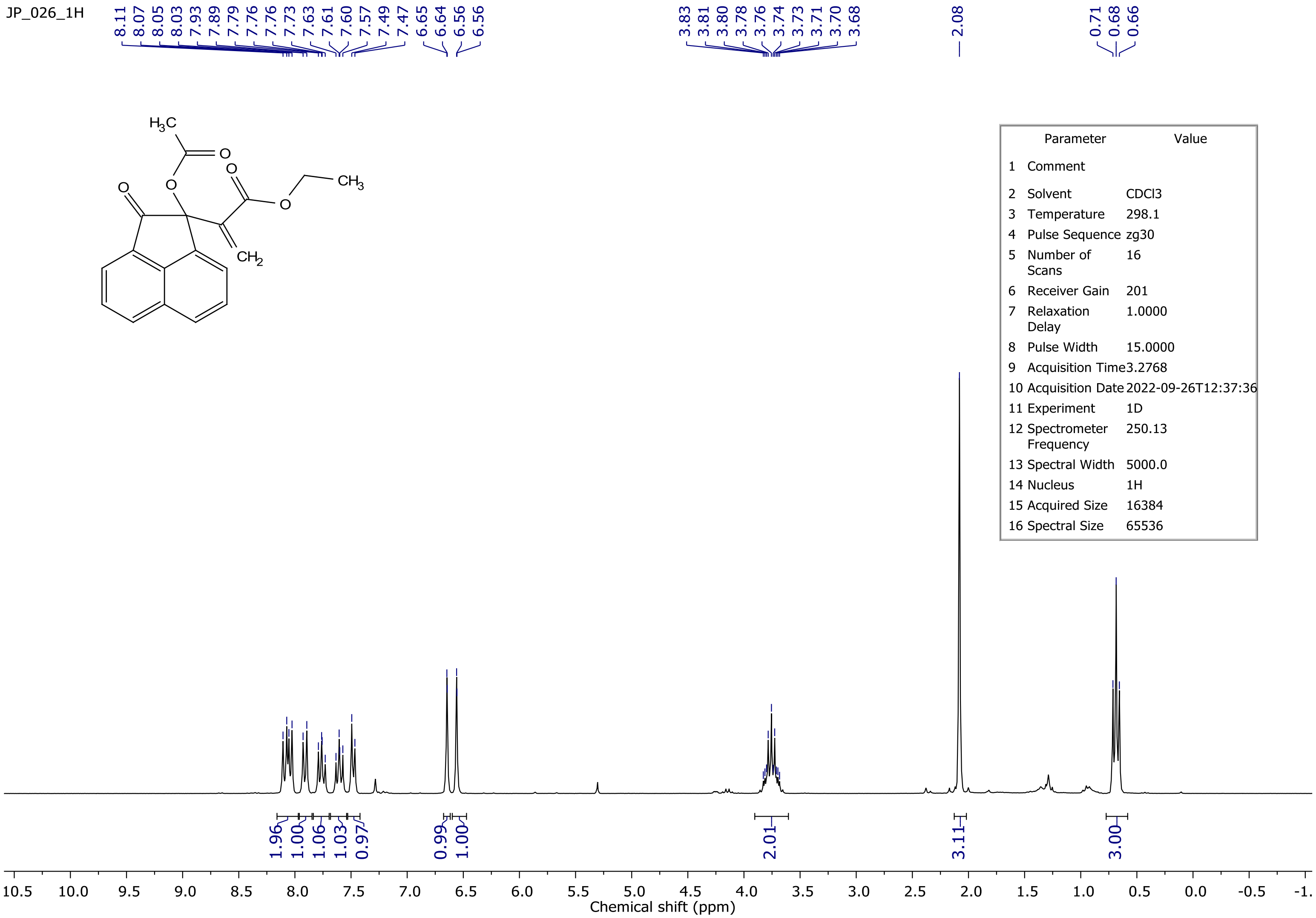

	Parameter	Value
1	Comment	
2	Solvent	CDCl3
3	Temperature	298.1
4	Pulse Sequence	zg30
5	Number of Scans	16
6	Receiver Gain	201
7	Relaxation Delay	1.0000
8	Pulse Width	15.0000
9	Acquisition Time	3.2768
10	Acquisition Date	2022-09-26T12:37:36
11	Experiment	1D
12	Spectrometer Frequency	250.13
13	Spectral Width	5000.0
14	Nucleus	1H
15	Acquired Size	16384
16	Spectral Size	65536

Figure S7 – ^{1}H NMR, 250 Hz, $CDCl_3$ (compound **3a**).

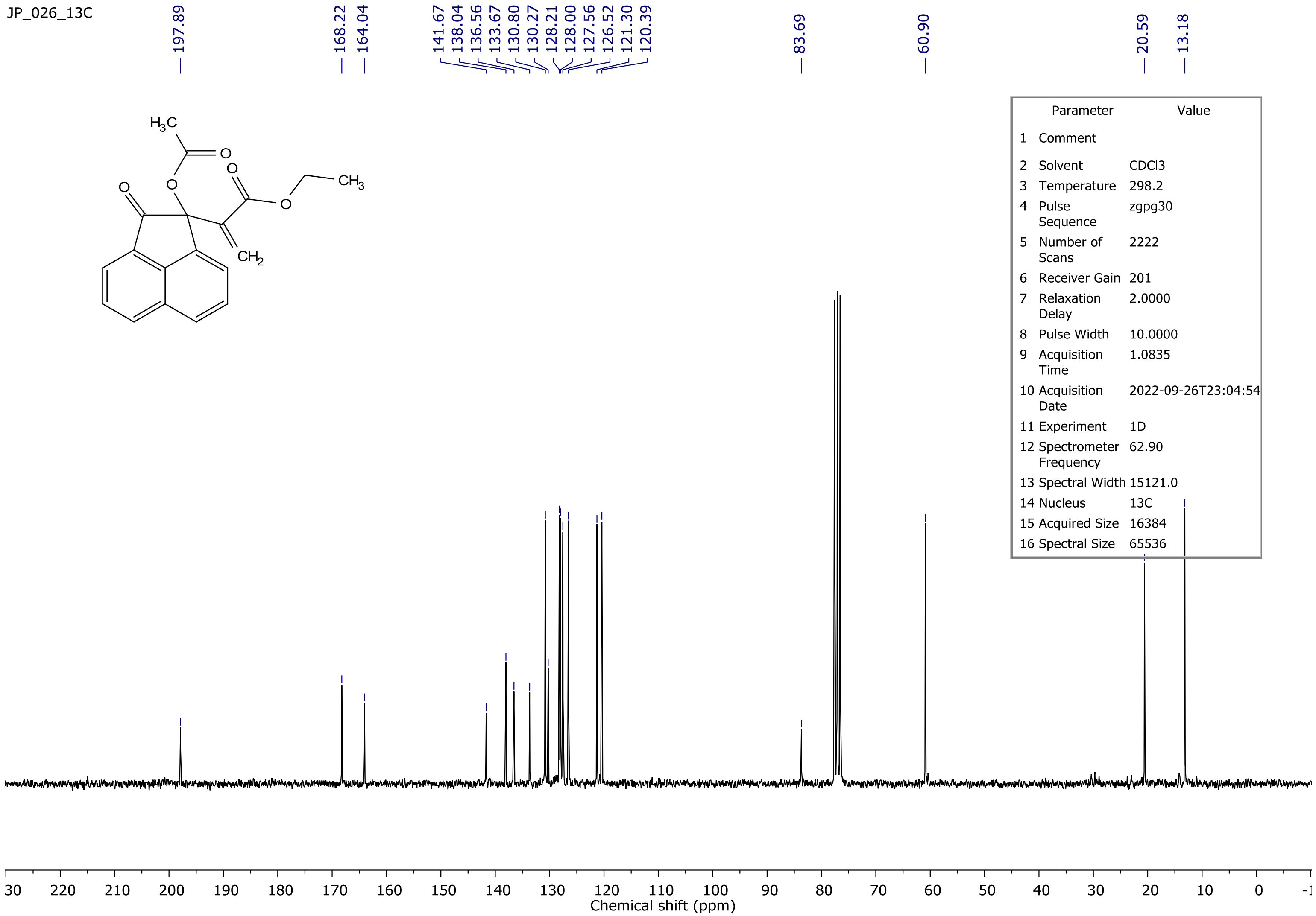

Figure S8 – ^{13}C NMR, 63 Hz, $CDCl_3$ (compound **3a**).

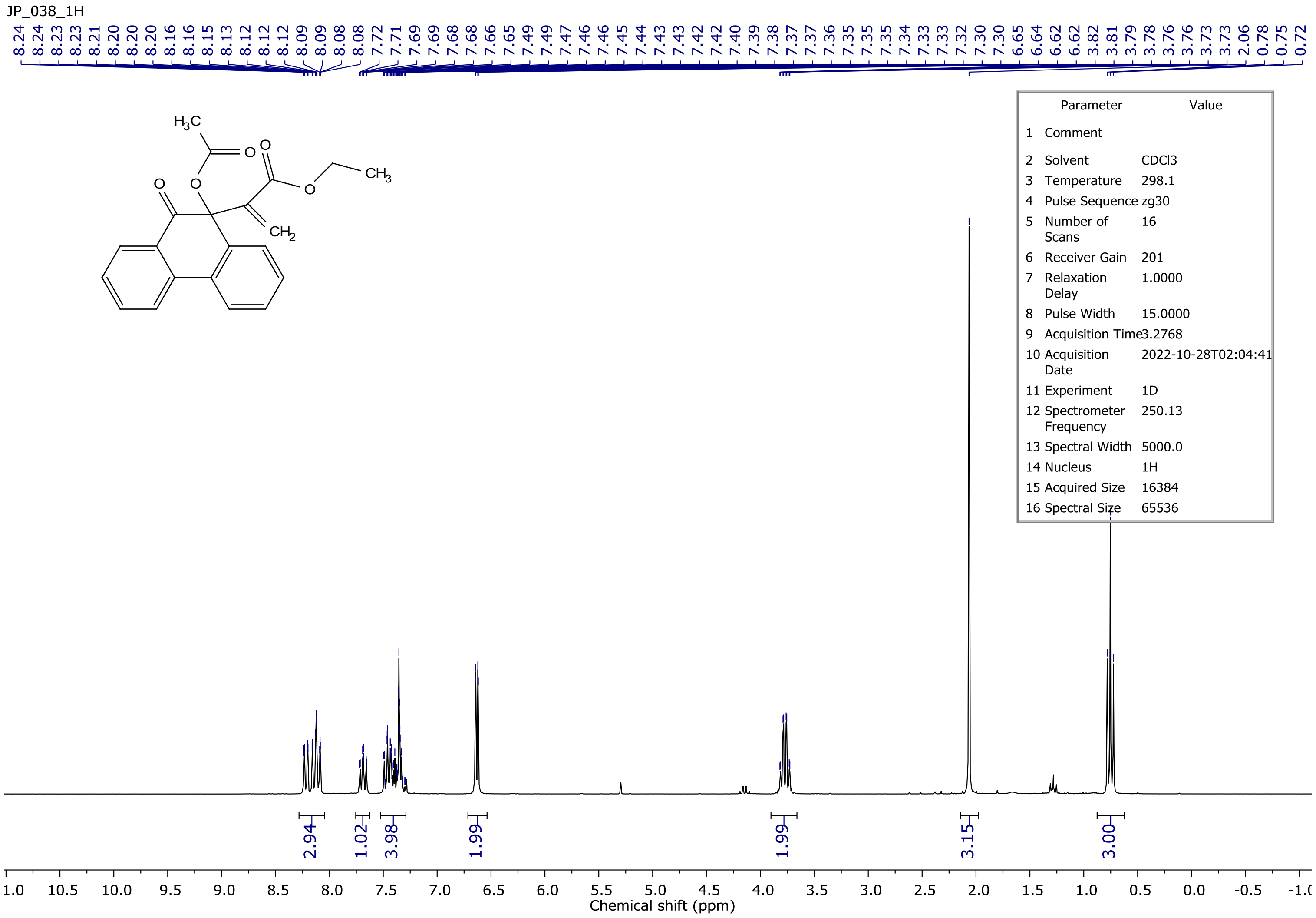

Figure S9 – ^{1}H NMR, 250 Hz, $CDCl_3$ (compound **3b**).

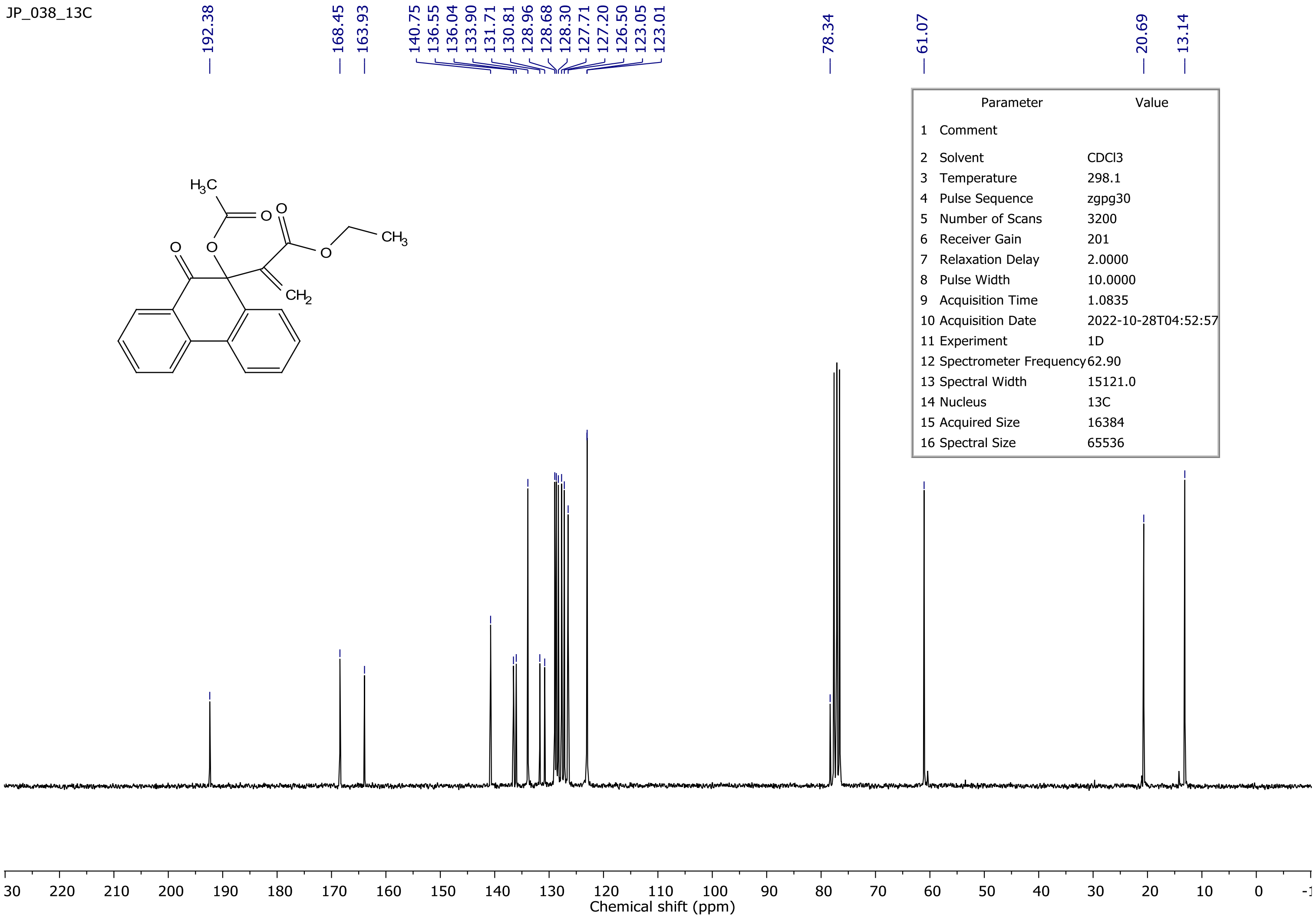

Figure S10 – ^{13}C NMR, 63 Hz, $CDCl_3$ (compound **3b**).

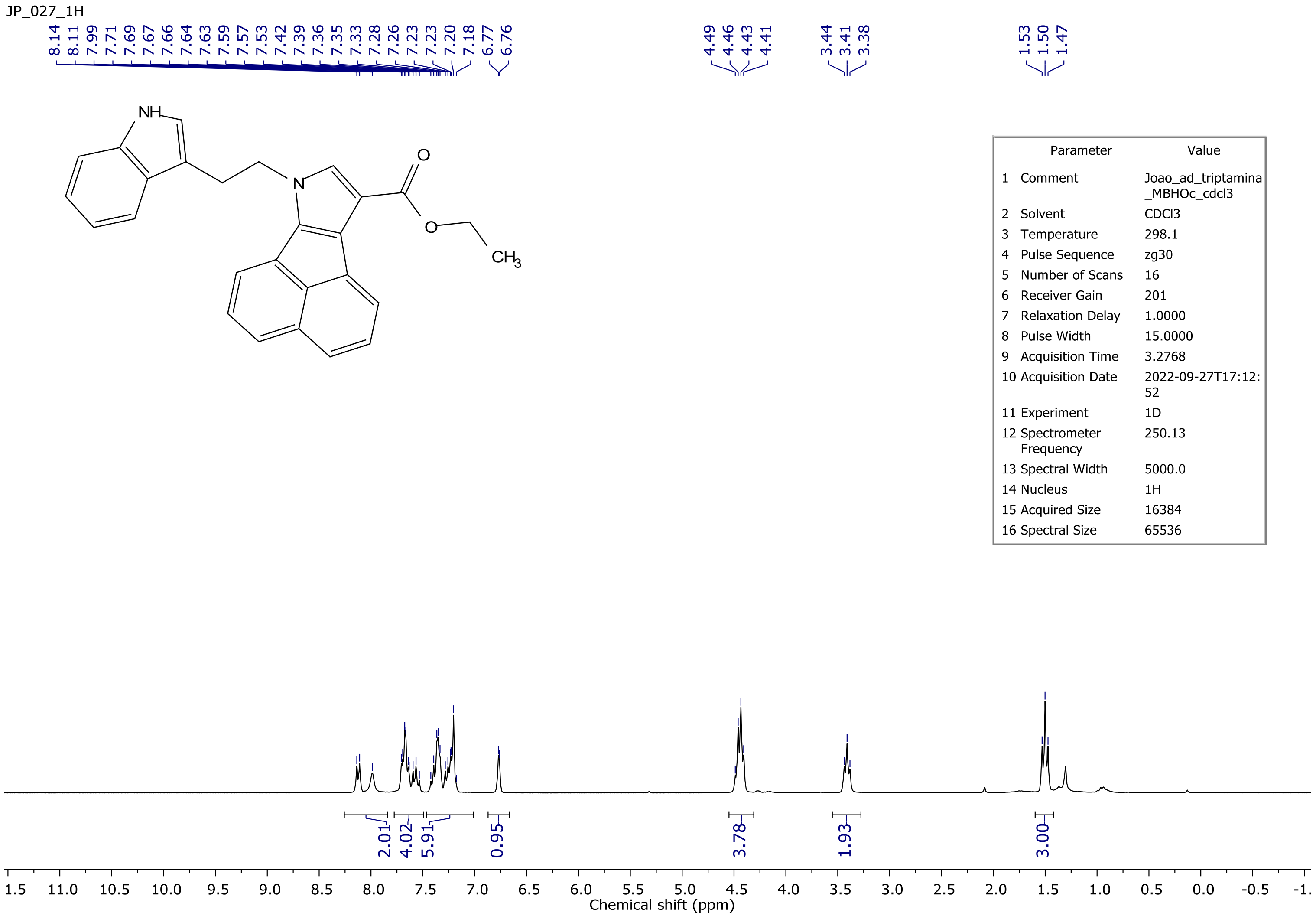

	Parameter	Value
1	Comment	Joao_ad_triptamina_MBHOc_cdcl3
2	Solvent	CDCl3
3	Temperature	298.1
4	Pulse Sequence	zg30
5	Number of Scans	16
6	Receiver Gain	201
7	Relaxation Delay	1.0000
8	Pulse Width	15.0000
9	Acquisition Time	3.2768
10	Acquisition Date	2022-09-27T17:12:52
11	Experiment	1D
12	Spectrometer Frequency	250.13
13	Spectral Width	5000.0
14	Nucleus	1H
15	Acquired Size	16384
16	Spectral Size	65536

Figure S11 – ^{1}H NMR, 250 Hz, $CDCl_3$ (compound **4a**).

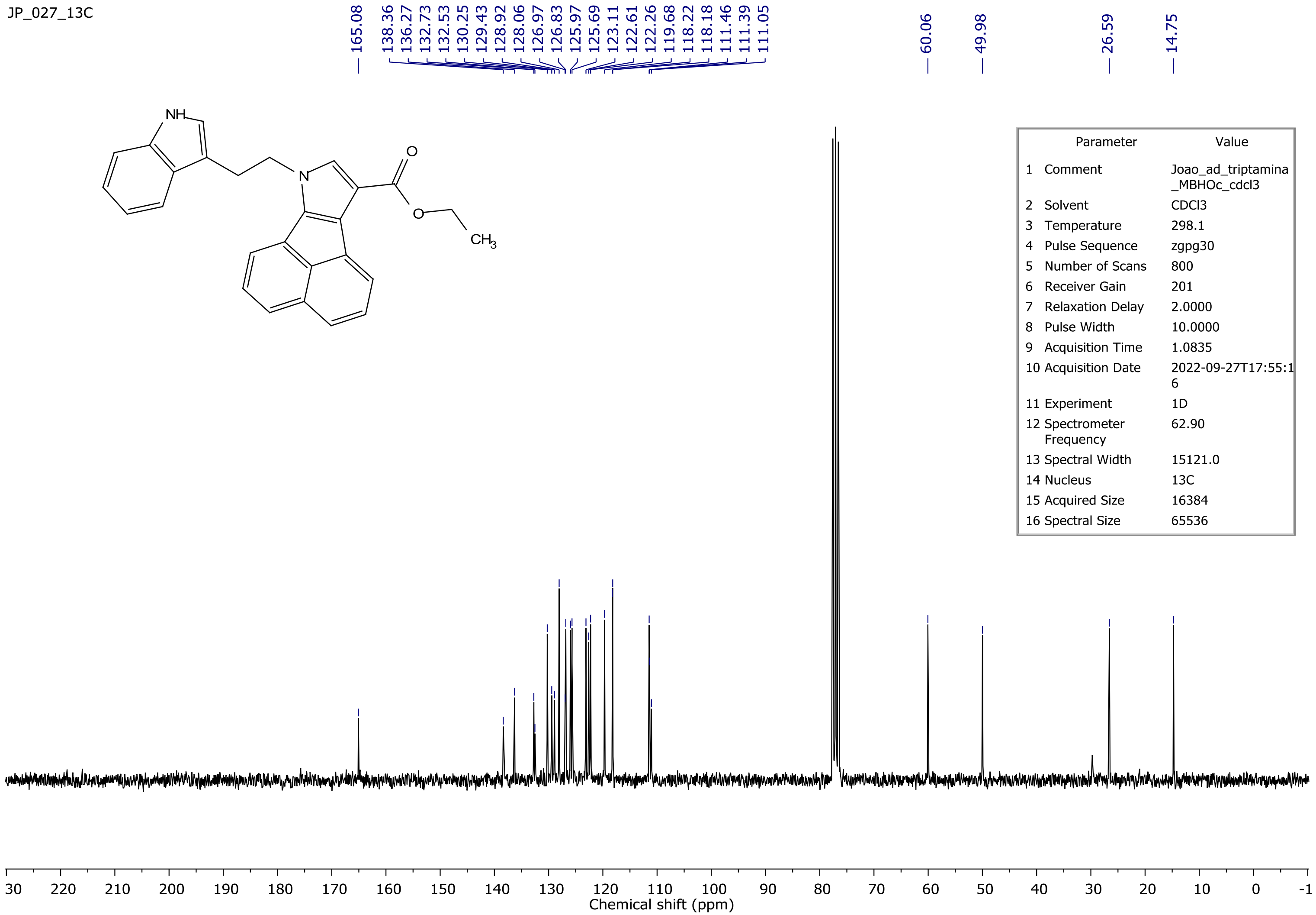

	Parameter	Value
1	Comment	Joao_ad_triptamina_MBHOc_cdcl3
2	Solvent	CDCl3
3	Temperature	298.1
4	Pulse Sequence	zgpg30
5	Number of Scans	800
6	Receiver Gain	201
7	Relaxation Delay	2.0000
8	Pulse Width	10.0000
9	Acquisition Time	1.0835
10	Acquisition Date	2022-09-27T17:55:16
11	Experiment	1D
12	Spectrometer Frequency	62.90
13	Spectral Width	15121.0
14	Nucleus	13C
15	Acquired Size	16384
16	Spectral Size	65536

Figure S12 – ^{13}C NMR, 63 Hz, $CDCl_3$ (compound **4a**).

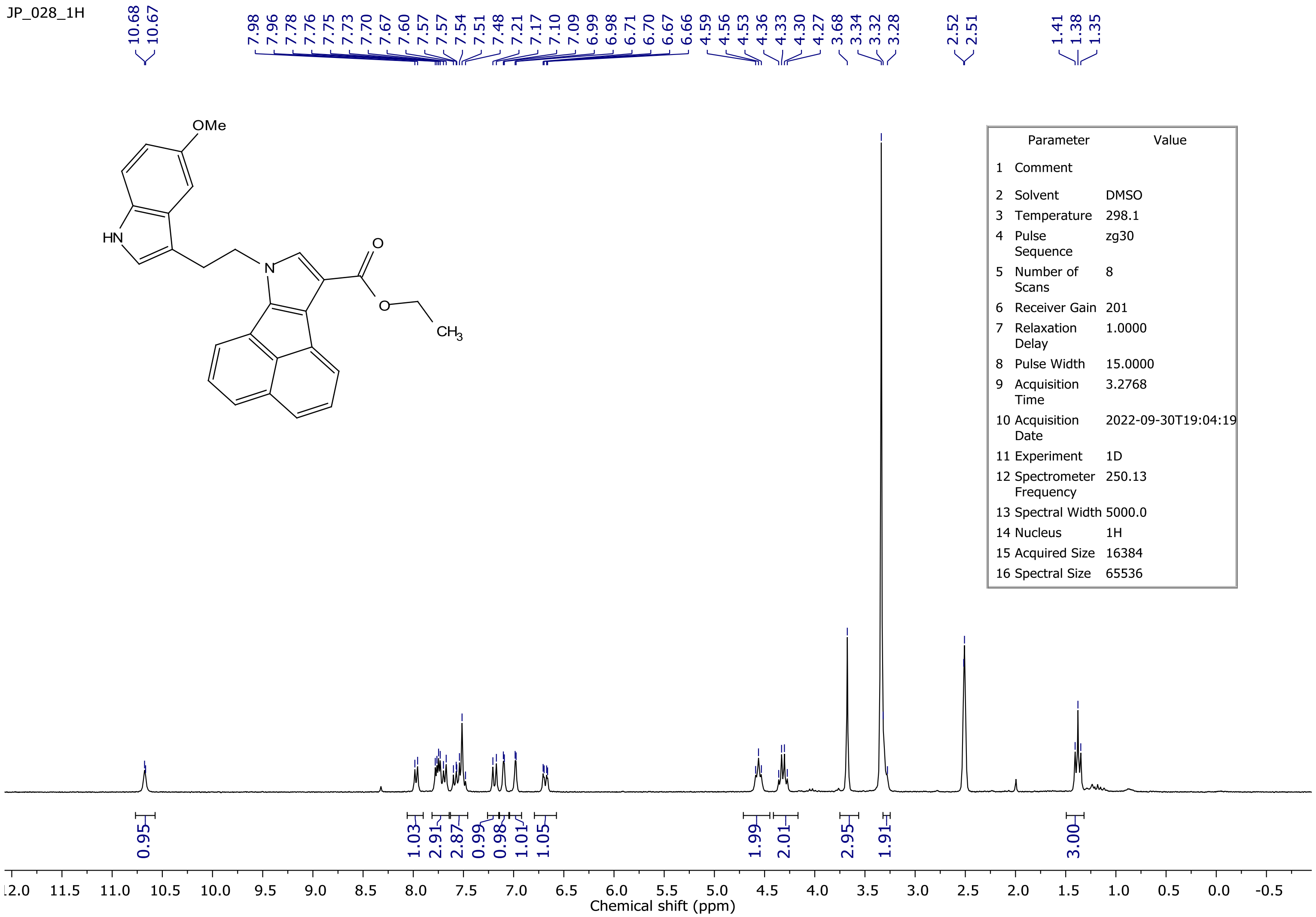

Figure S13 – ^{1}H NMR, 250 Hz, DMSO-d$_6$ (compound **4b**).

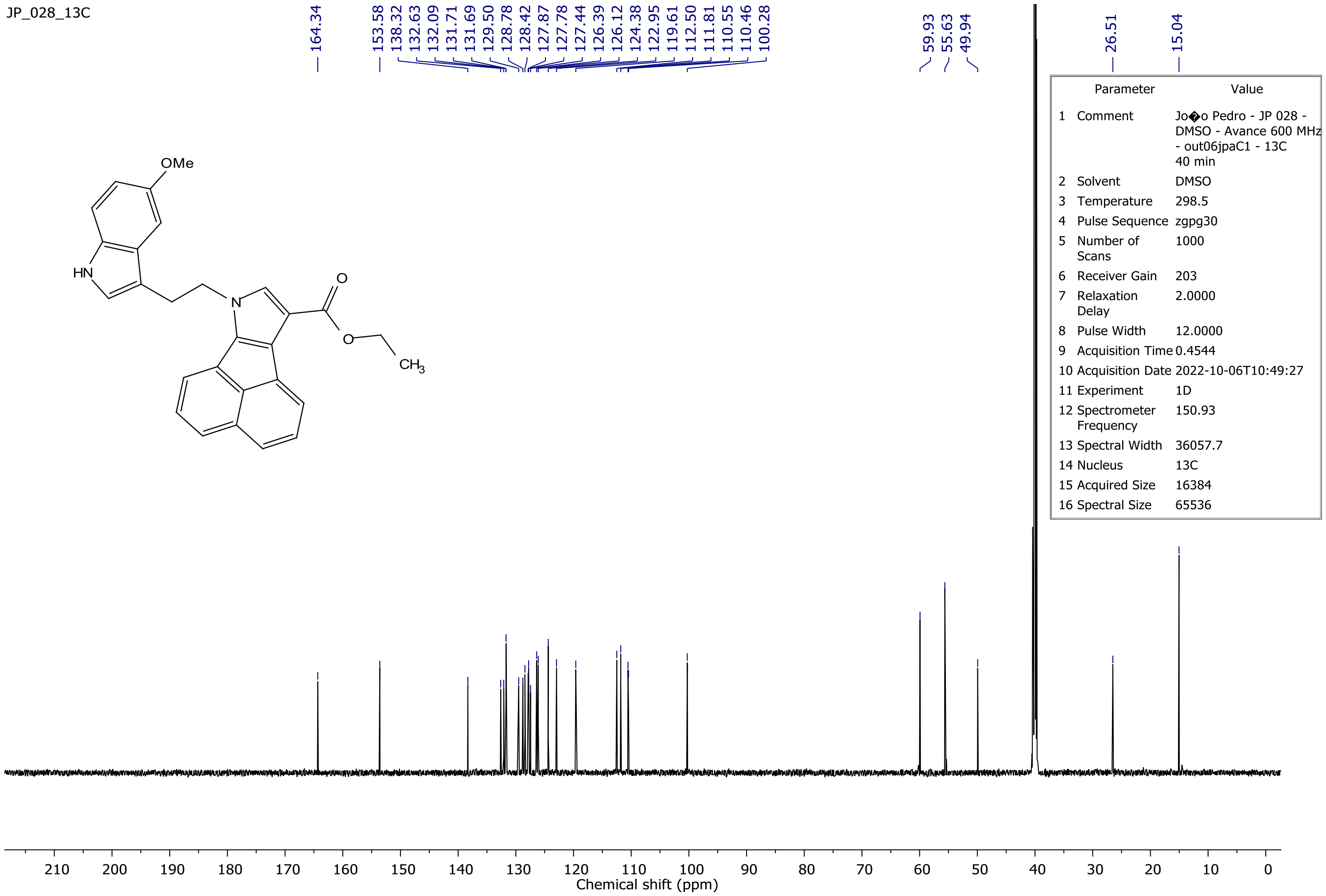

Figure S14 – ^{13}C NMR, 63 Hz, DMSO-d_6 (compond **4b**).

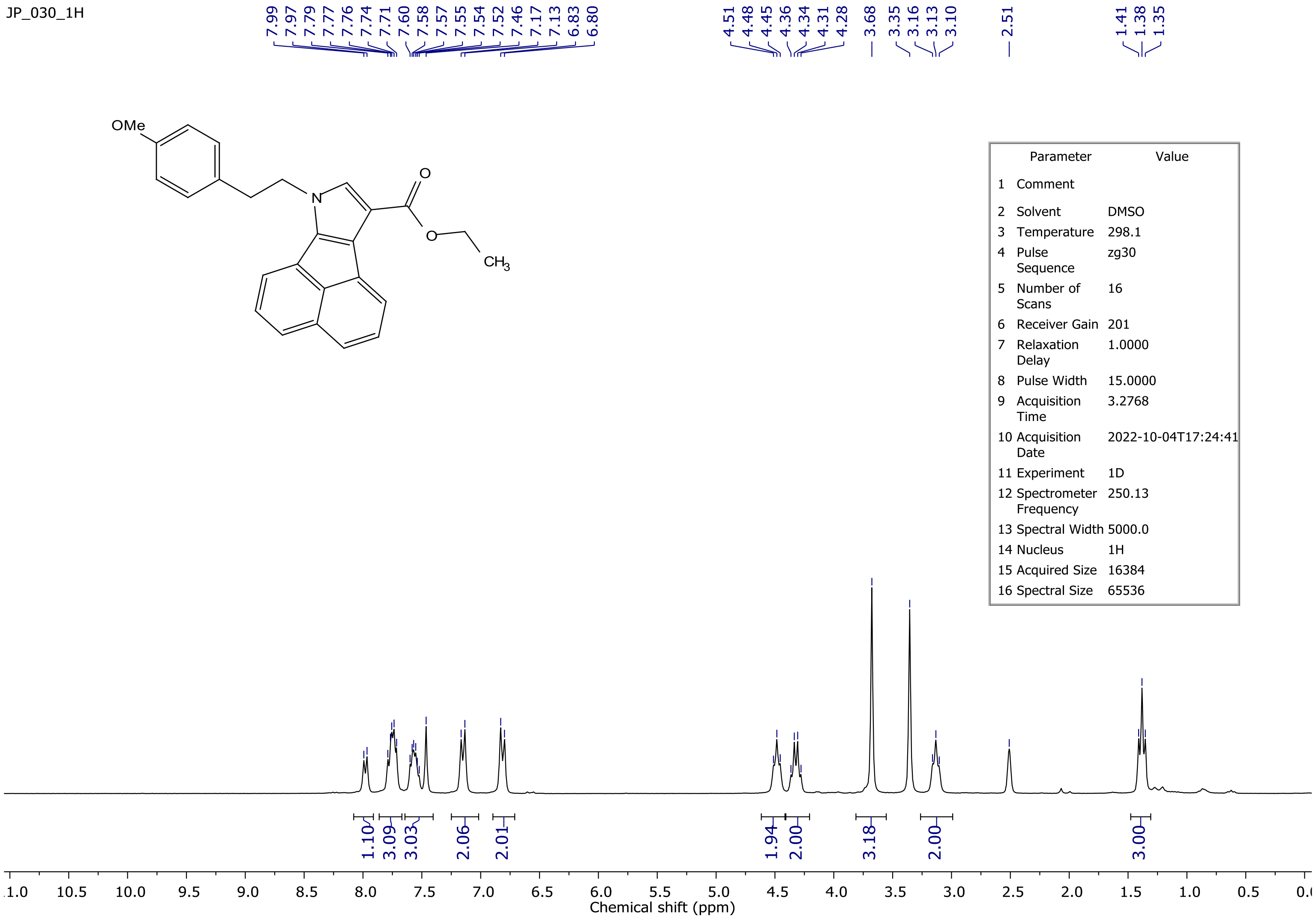

Figure S15 – ^{1}H NMR, 250 Hz, DMSO-d_6 (compound **4c**).

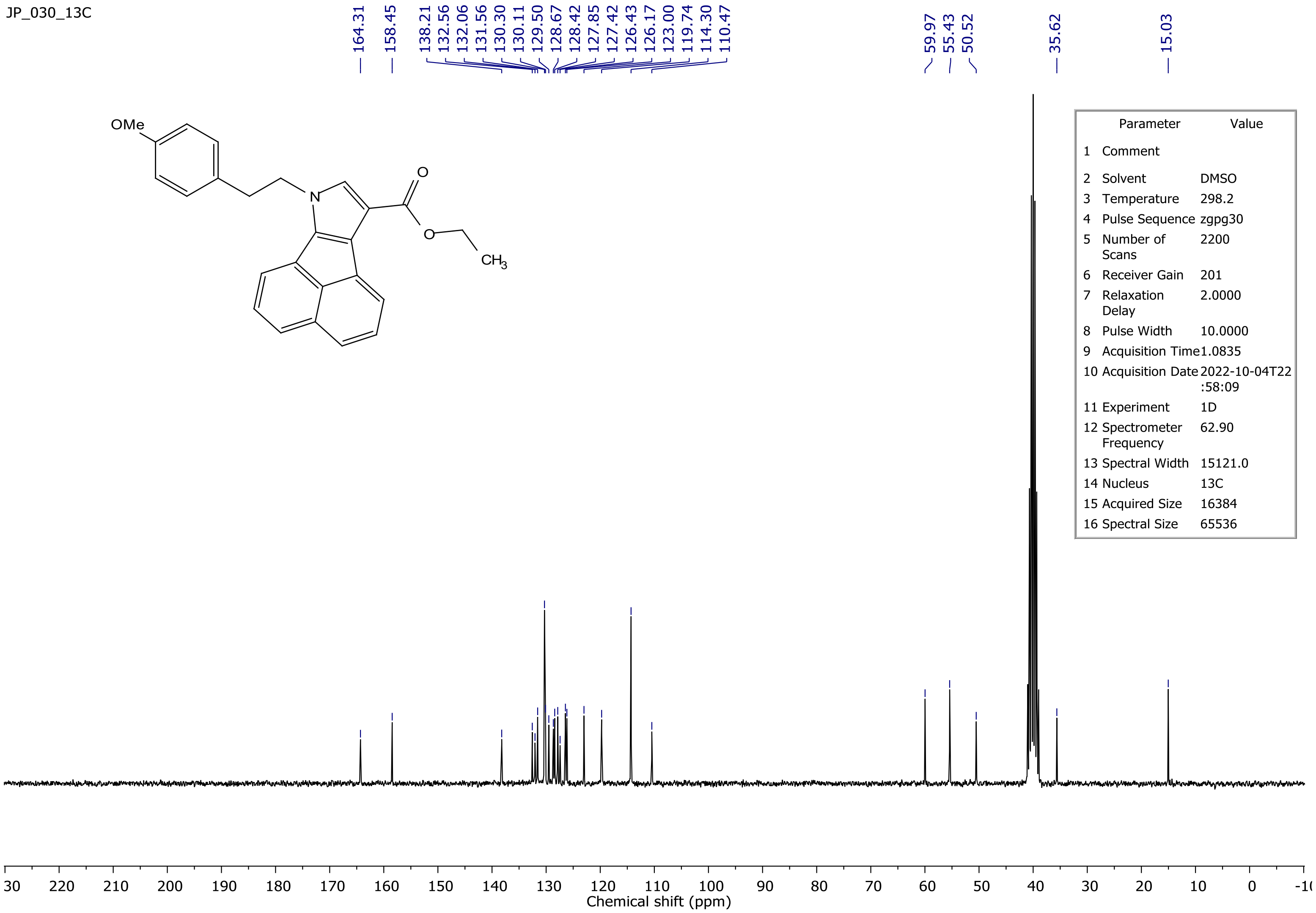

Figure S16 – ^{13}C NMR, 63 Hz, DMSO-d_6 (compound **4c**).

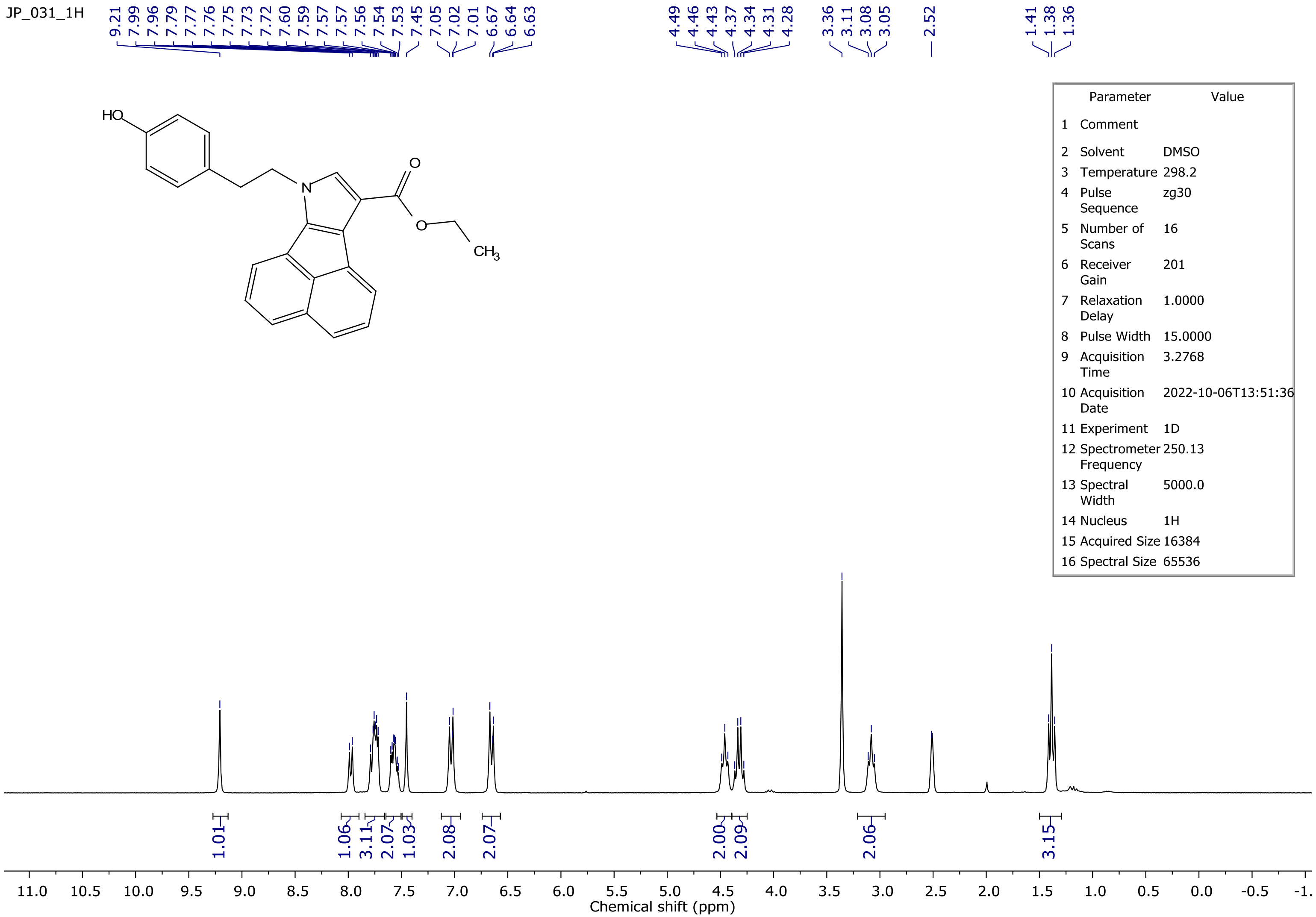

Figure S17 – ^{1}H NMR, 250 Hz, DMSO-d$_6$ (compound **4d**).

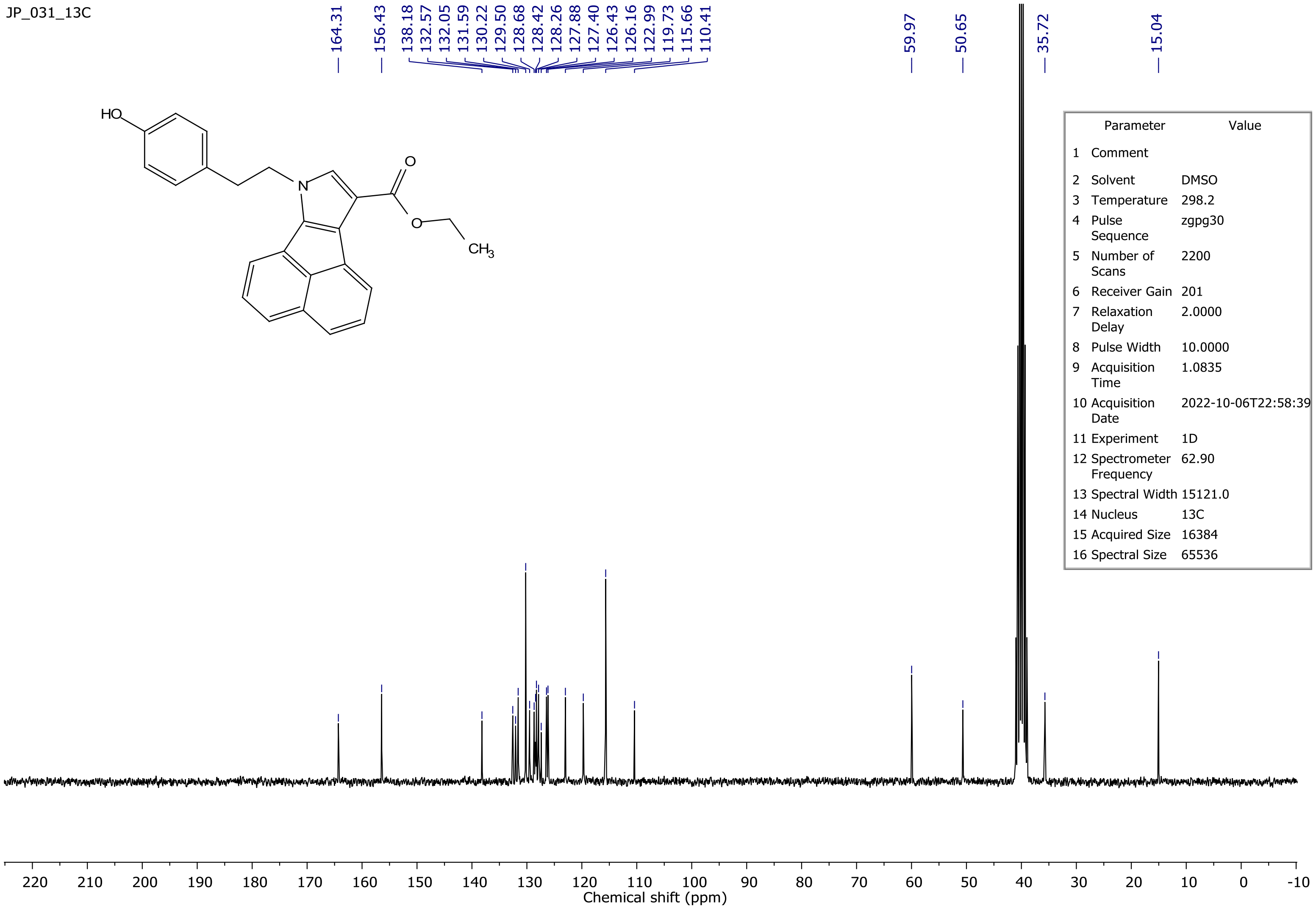

	Parameter	Value
1	Comment	
2	Solvent	DMSO
3	Temperature	298.2
4	Pulse Sequence	zgpg30
5	Number of Scans	2200
6	Receiver Gain	201
7	Relaxation Delay	2.0000
8	Pulse Width	10.0000
9	Acquisition Time	1.0835
10	Acquisition Date	2022-10-06T22:58:39
11	Experiment	1D
12	Spectrometer Frequency	62.90
13	Spectral Width	15121.0
14	Nucleus	13C
15	Acquired Size	16384
16	Spectral Size	65536

Figure S18 – ^{13}C NMR, 63 Hz, DMSO-d$_6$ (compound **4d**).

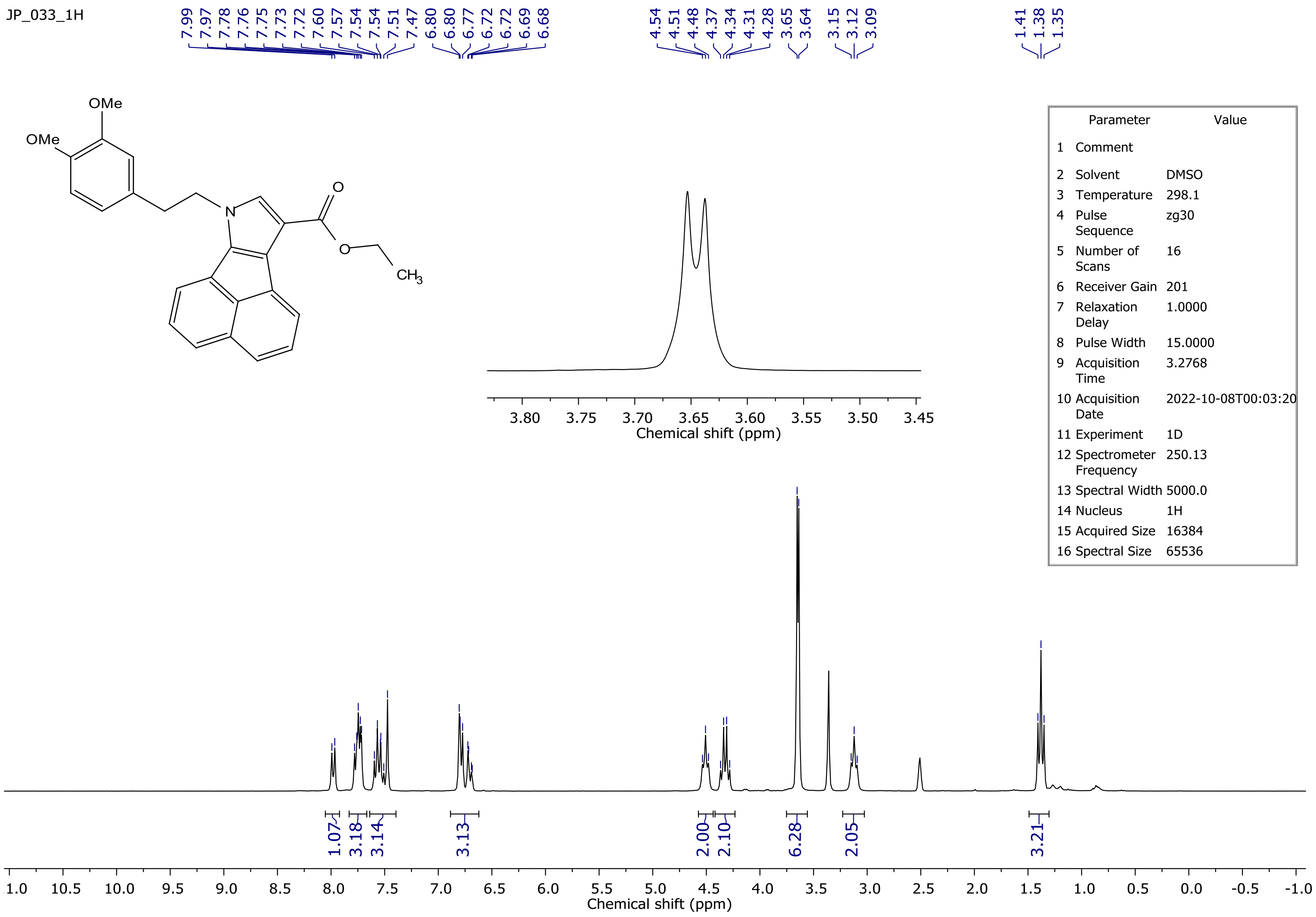

Figure S19 – ^{1}H NMR, 250 Hz, DMSO-d$_6$ (compound **4e**).

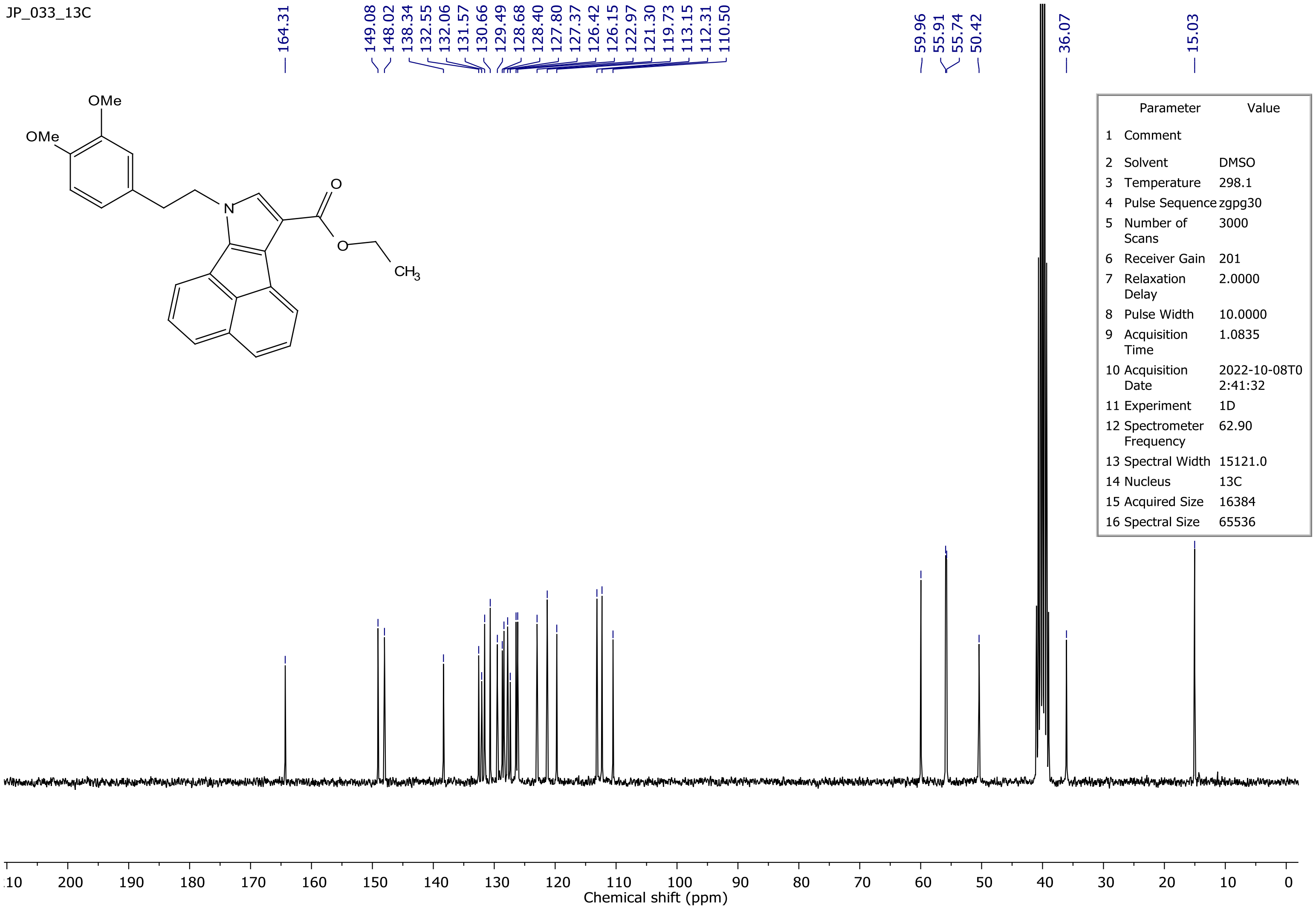

	Parameter	Value
1	Comment	
2	Solvent	DMSO
3	Temperature	298.1
4	Pulse Sequence	zgpg30
5	Number of Scans	3000
6	Receiver Gain	201
7	Relaxation Delay	2.0000
8	Pulse Width	10.0000
9	Acquisition Time	1.0835
10	Acquisition Date	2022-10-08T02:41:32
11	Experiment	1D
12	Spectrometer Frequency	62.90
13	Spectral Width	15121.0
14	Nucleus	13C
15	Acquired Size	16384
16	Spectral Size	65536

Figure S20 – ^{13}C NMR, 63 Hz, DMSO-d_6 (compound **4e**).

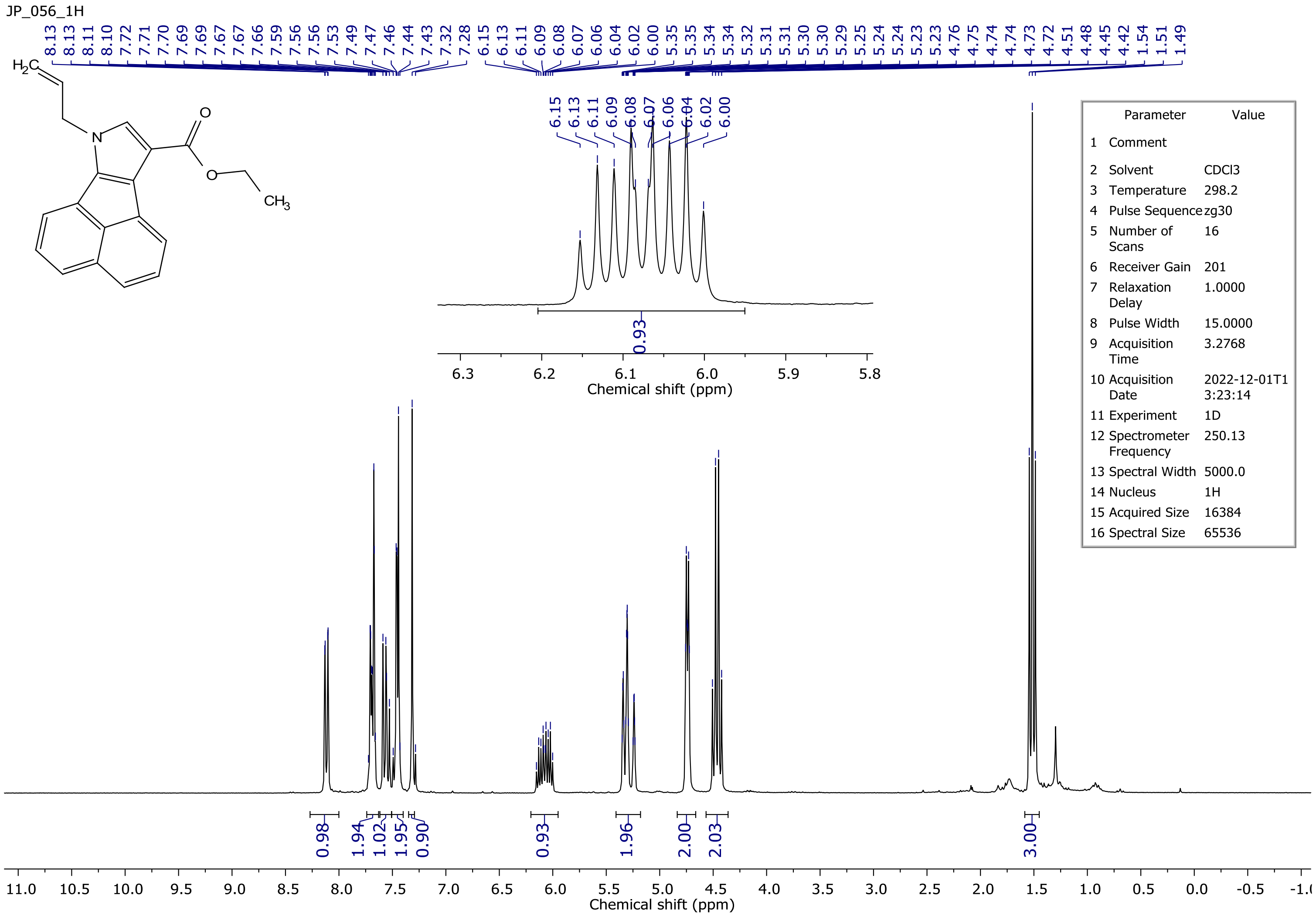

Figure S21 – ^{1}H NMR, 250 Hz, $CDCl_3$ (compound 4f).

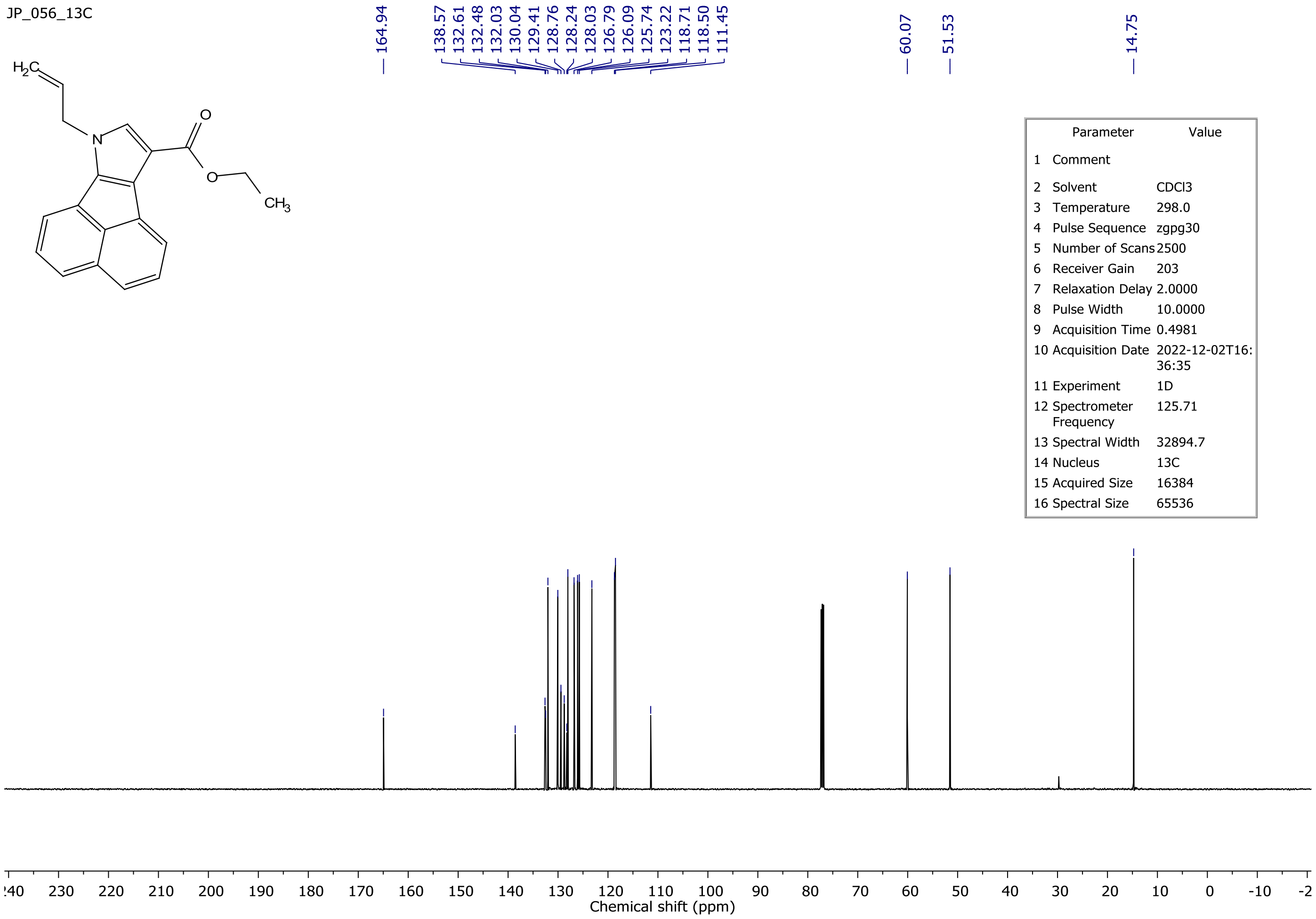

Figure S22 – ^{13}C NMR, 63 Hz, CDCl3 (compound **4f**).

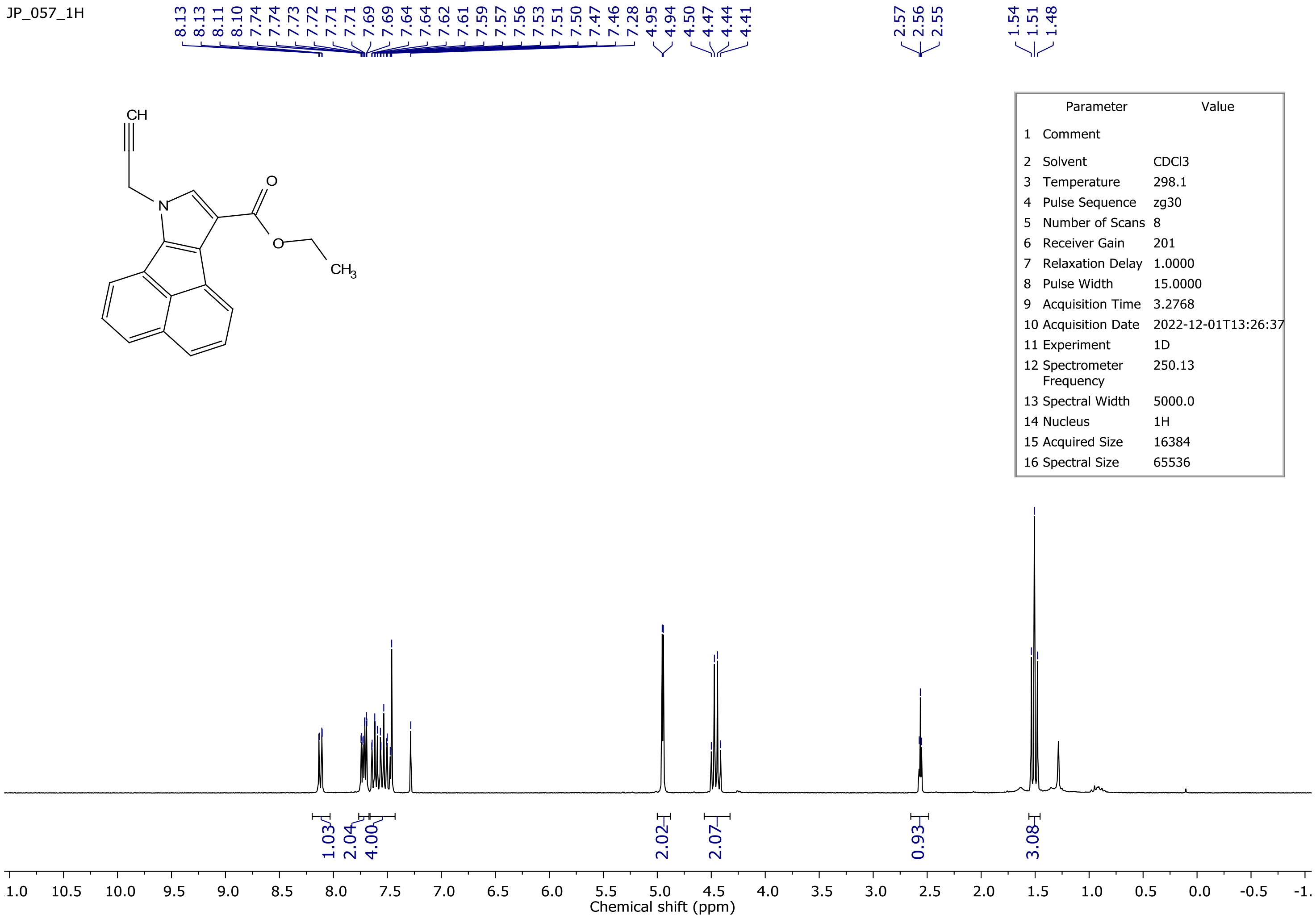

Figure S23 – ^{1}H NMR, 250 Hz, $CDCl_3$ (compound **4g**).

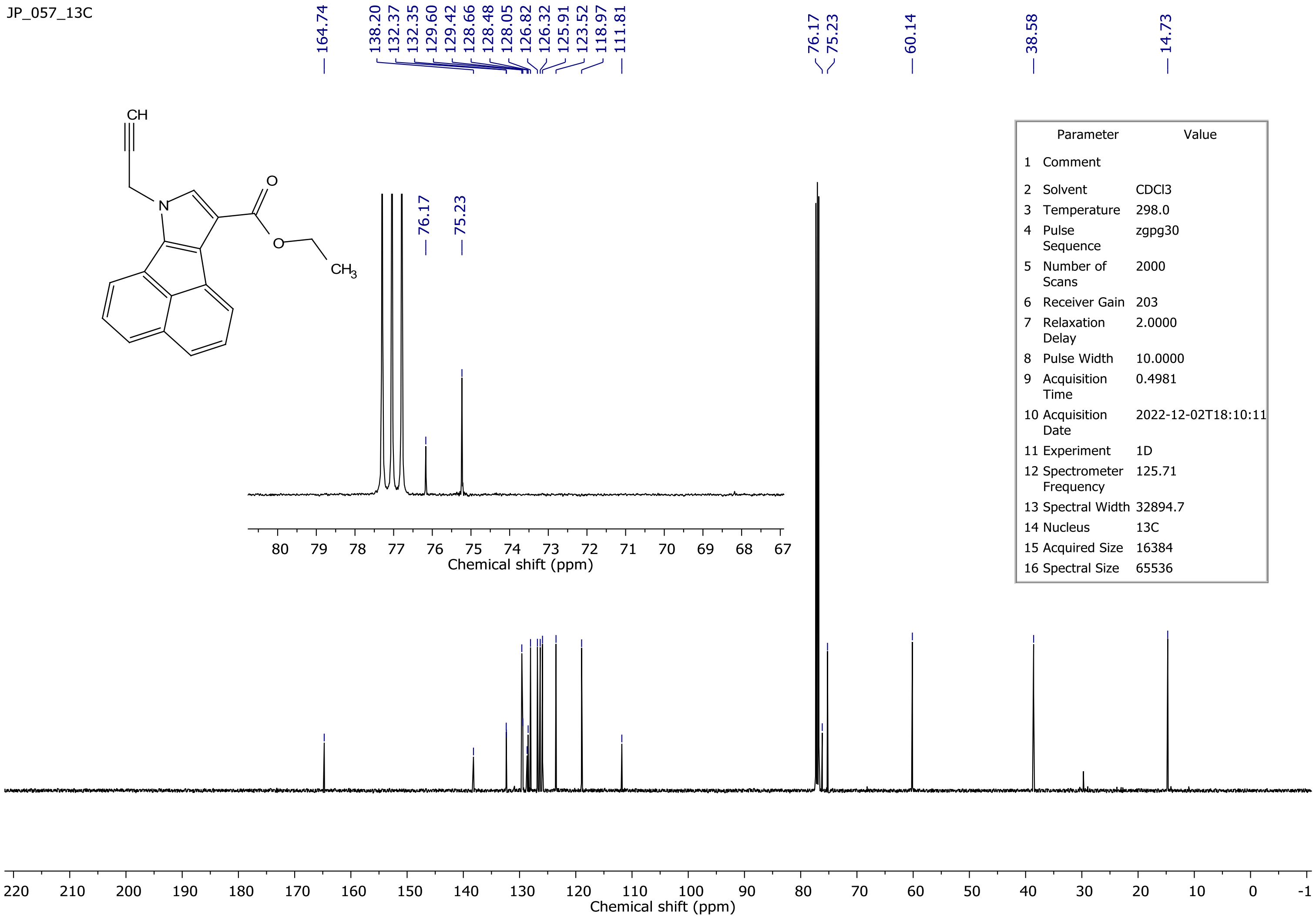

Figure S24 – ^{13}C NMR, 63 Hz, CDCl3 (compound **4g**).

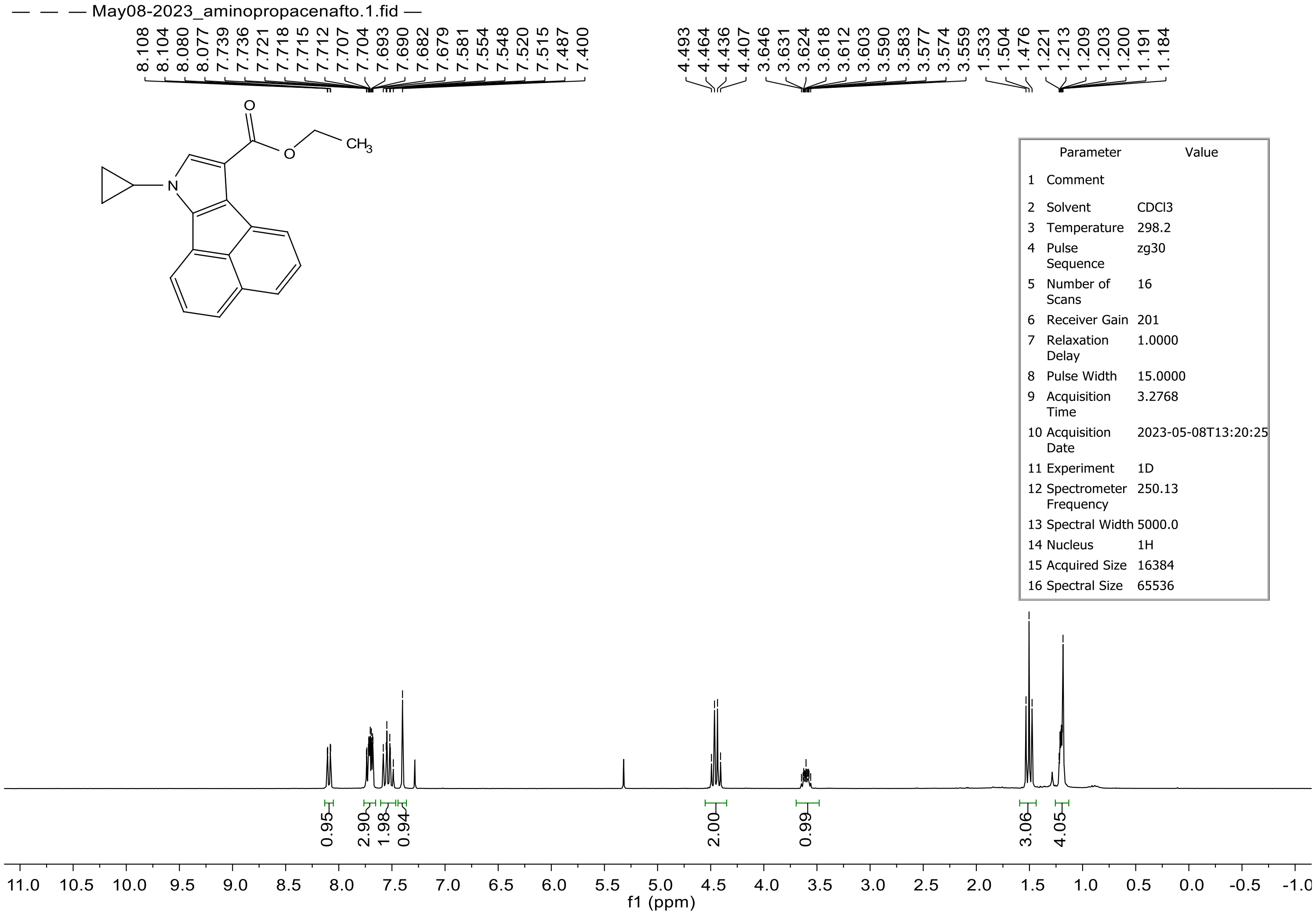

Figure S25 – ^{1}H NMR, 250 Hz, $CDCl_3$ (compound **4j**).

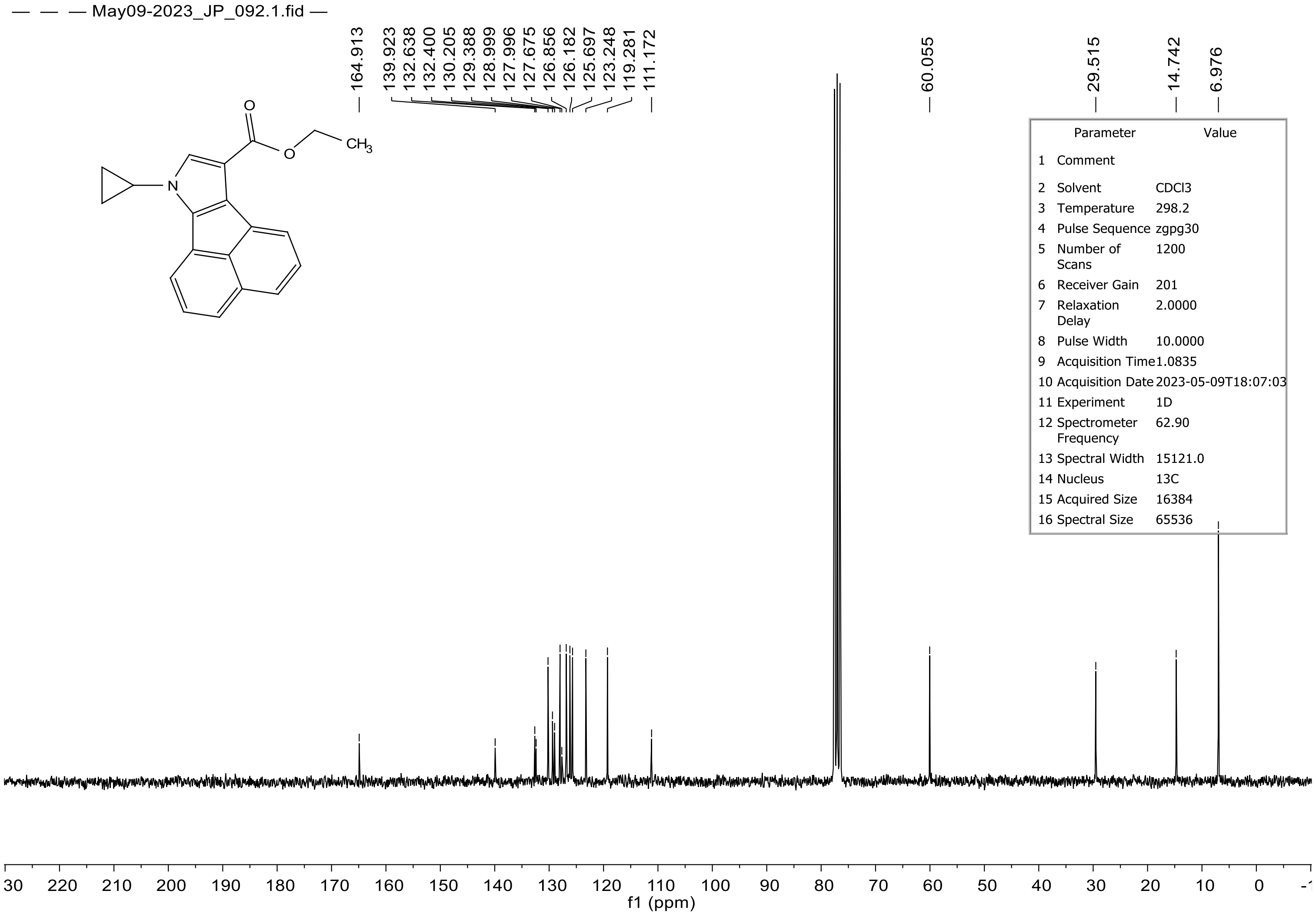

Figure S26 – ^{13}C NMR, 63 Hz, CDCl3 (compound **4j**).

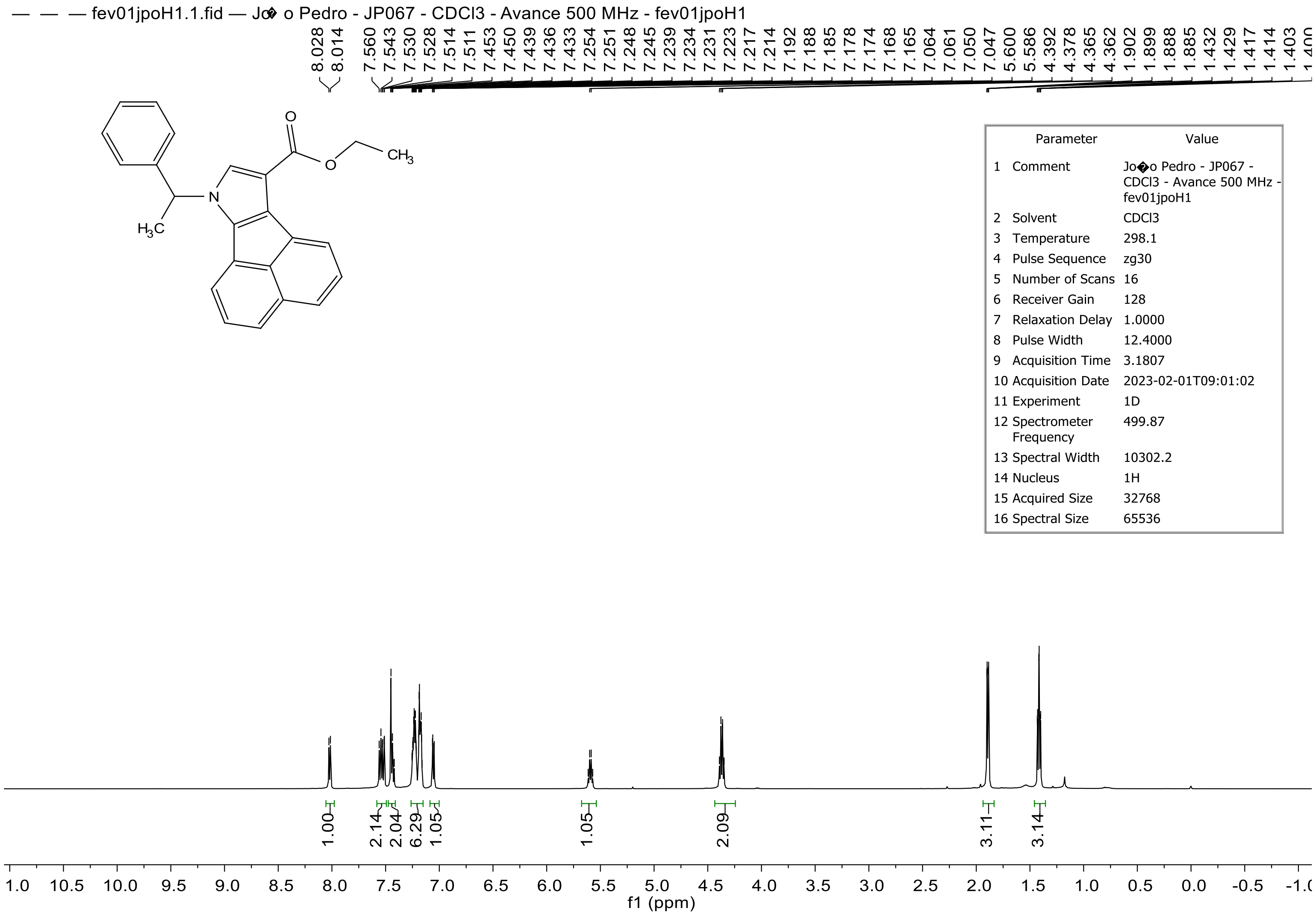

Figure S27 – ^{1}H NMR, 500 Hz, $CDCl_3$ (compound **4k**).

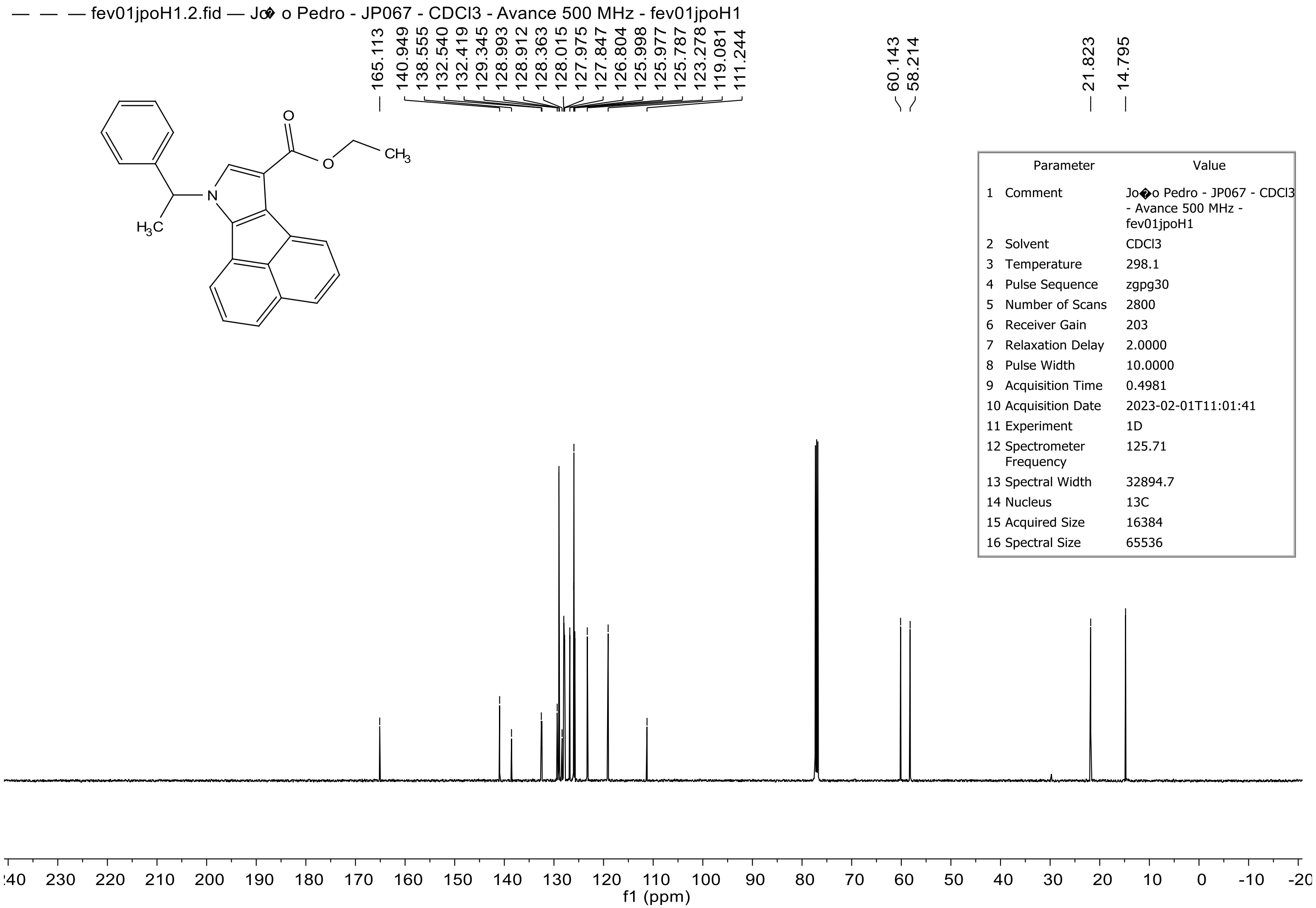

Figure S28 – ^{13}C NMR, 125 Hz, $CDCl_3$ (compound **4k**).

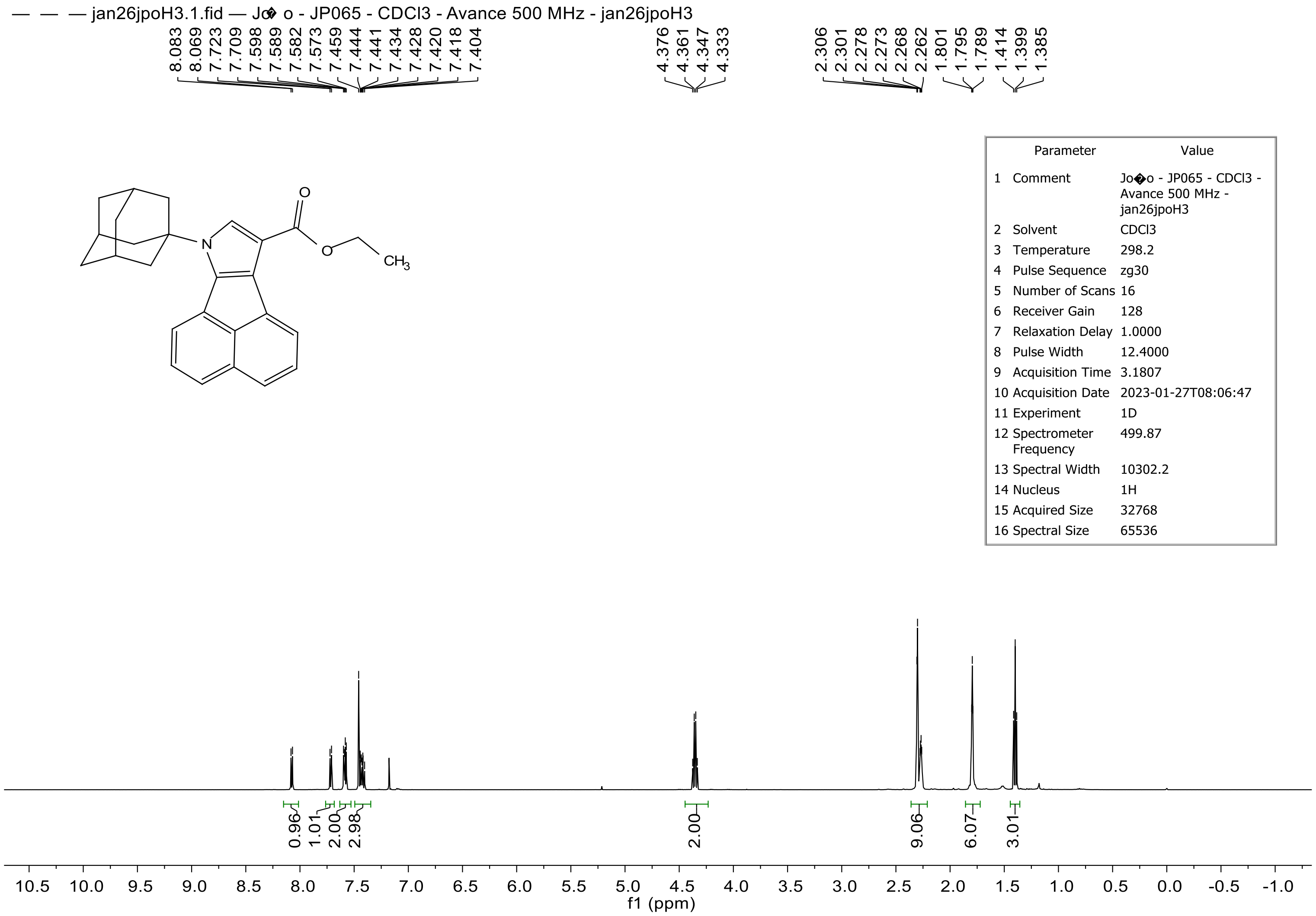

	Parameter	Value
1	Comment	Jo�o - JP065 - CDCl3 - Avance 500 MHz - jan26jpoH3
2	Solvent	CDCl3
3	Temperature	298.2
4	Pulse Sequence	zg30
5	Number of Scans	16
6	Receiver Gain	128
7	Relaxation Delay	1.0000
8	Pulse Width	12.4000
9	Acquisition Time	3.1807
10	Acquisition Date	2023-01-27T08:06:47
11	Experiment	1D
12	Spectrometer Frequency	499.87
13	Spectral Width	10302.2
14	Nucleus	1H
15	Acquired Size	32768
16	Spectral Size	65536

Figure S29 – ^{1}H NMR, 500 Hz, $CDCl_3$ (compound **4l**).

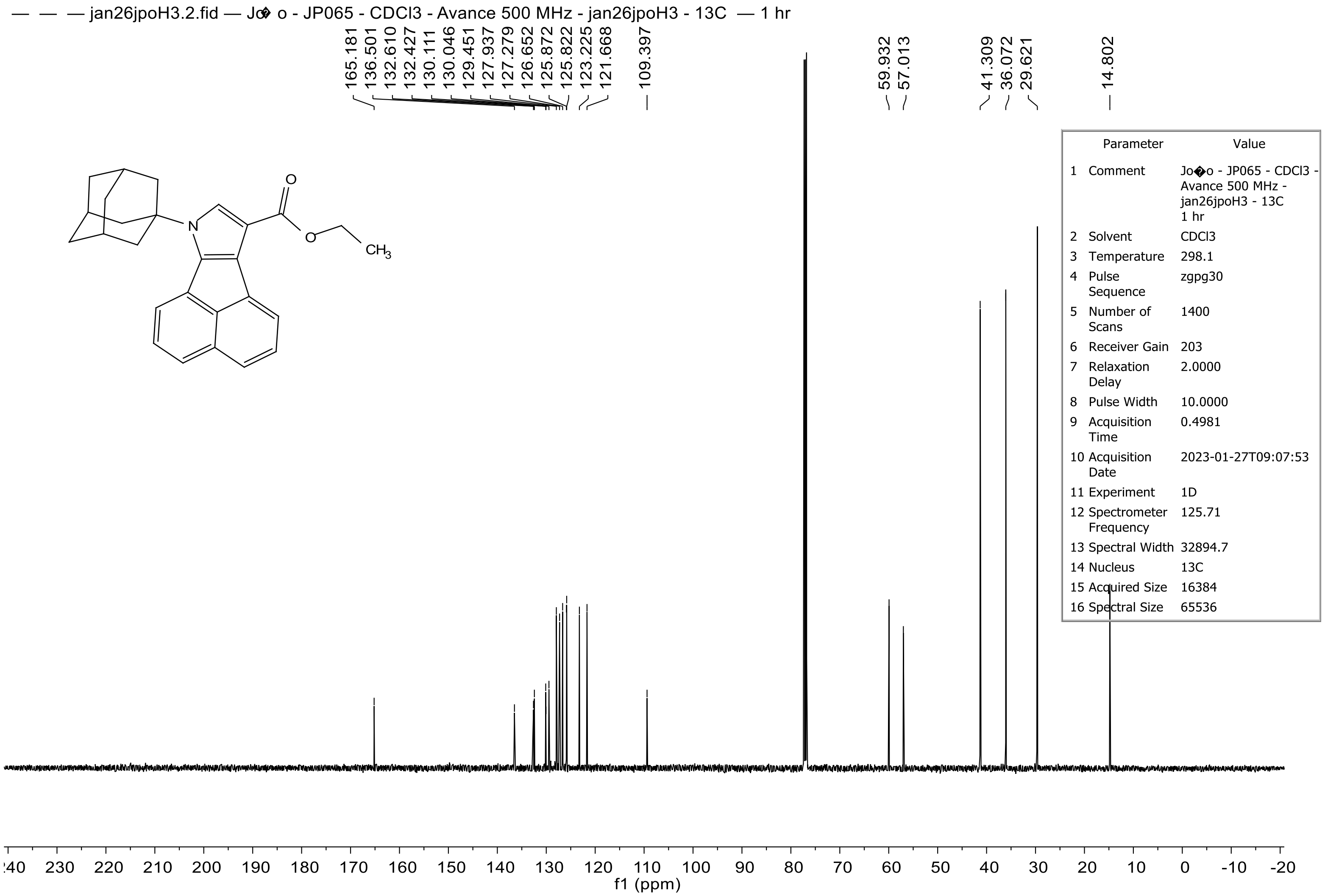

Figure S30 – ^{13}C NMR, 125 Hz, $CDCl_3$ (compound **4l**).

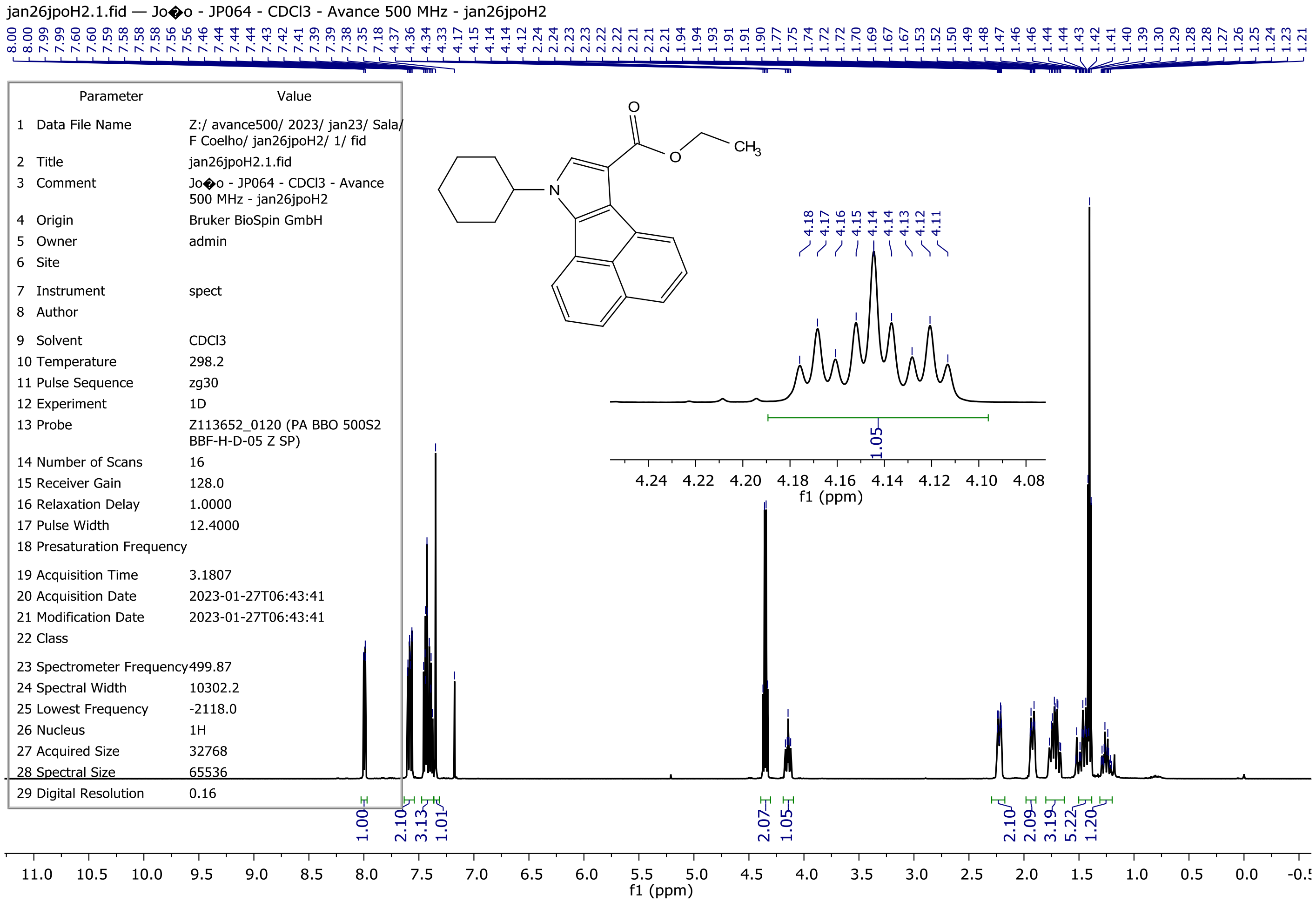

Figure S31 – ^{1}H NMR, 500 Hz, $CDCl_3$ (compound **4m**).

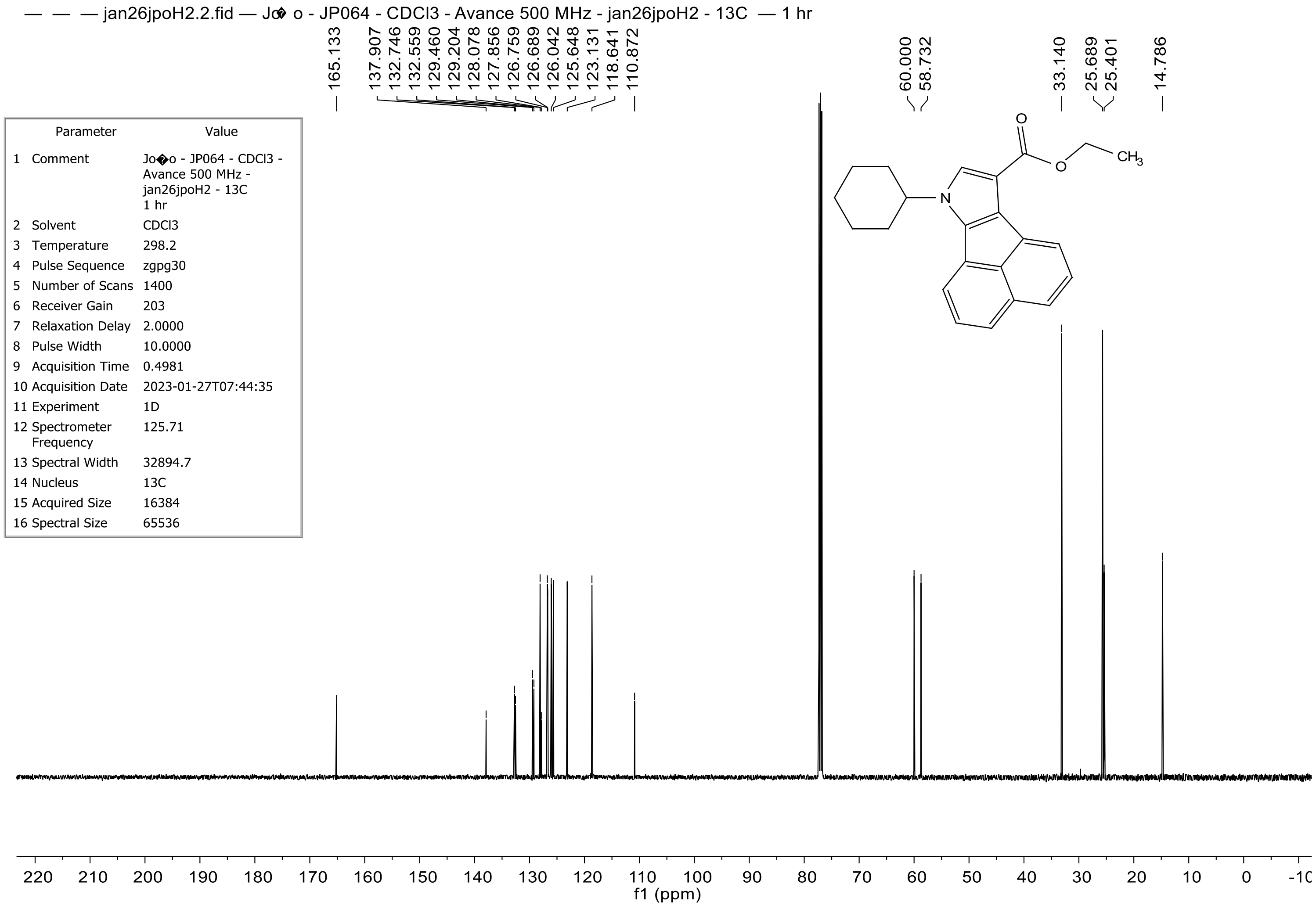

	Parameter	Value
1	Comment	Jo�o - JP064 - CDCl3 - Avance 500 MHz - jan26jpoH2 - 13C 1 hr
2	Solvent	CDCl3
3	Temperature	298.2
4	Pulse Sequence	zgpg30
5	Number of Scans	1400
6	Receiver Gain	203
7	Relaxation Delay	2.0000
8	Pulse Width	10.0000
9	Acquisition Time	0.4981
10	Acquisition Date	2023-01-27T07:44:35
11	Experiment	1D
12	Spectrometer Frequency	125.71
13	Spectral Width	32894.7
14	Nucleus	13C
15	Acquired Size	16384
16	Spectral Size	65536

Figure S32 – ^{13}C NMR, 125 Hz, $CDCl_3$ (compound **4m**).

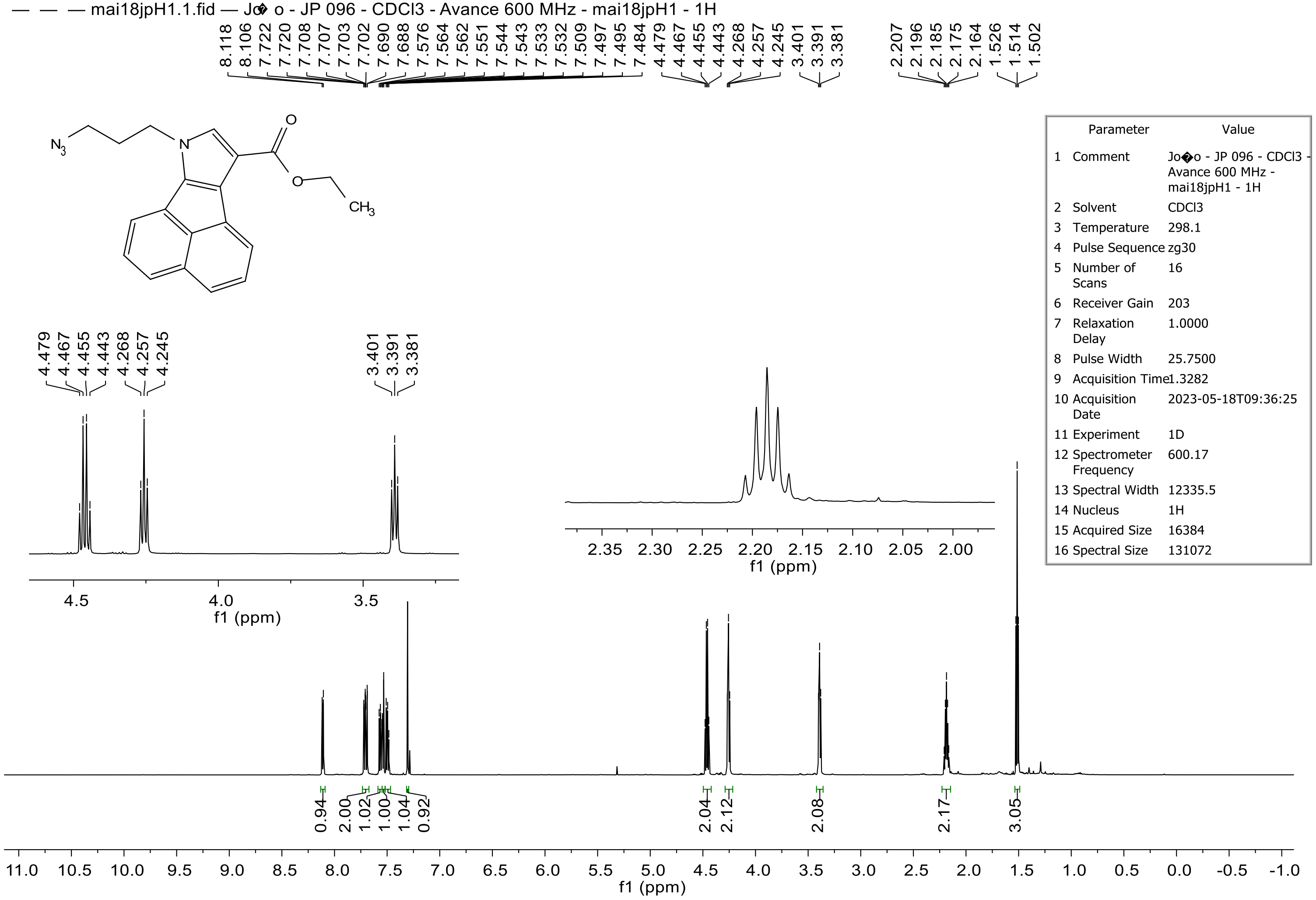

Figure S33 – ^{1}H NMR, 600 Hz, $CDCl_3$ (compound **4n**).

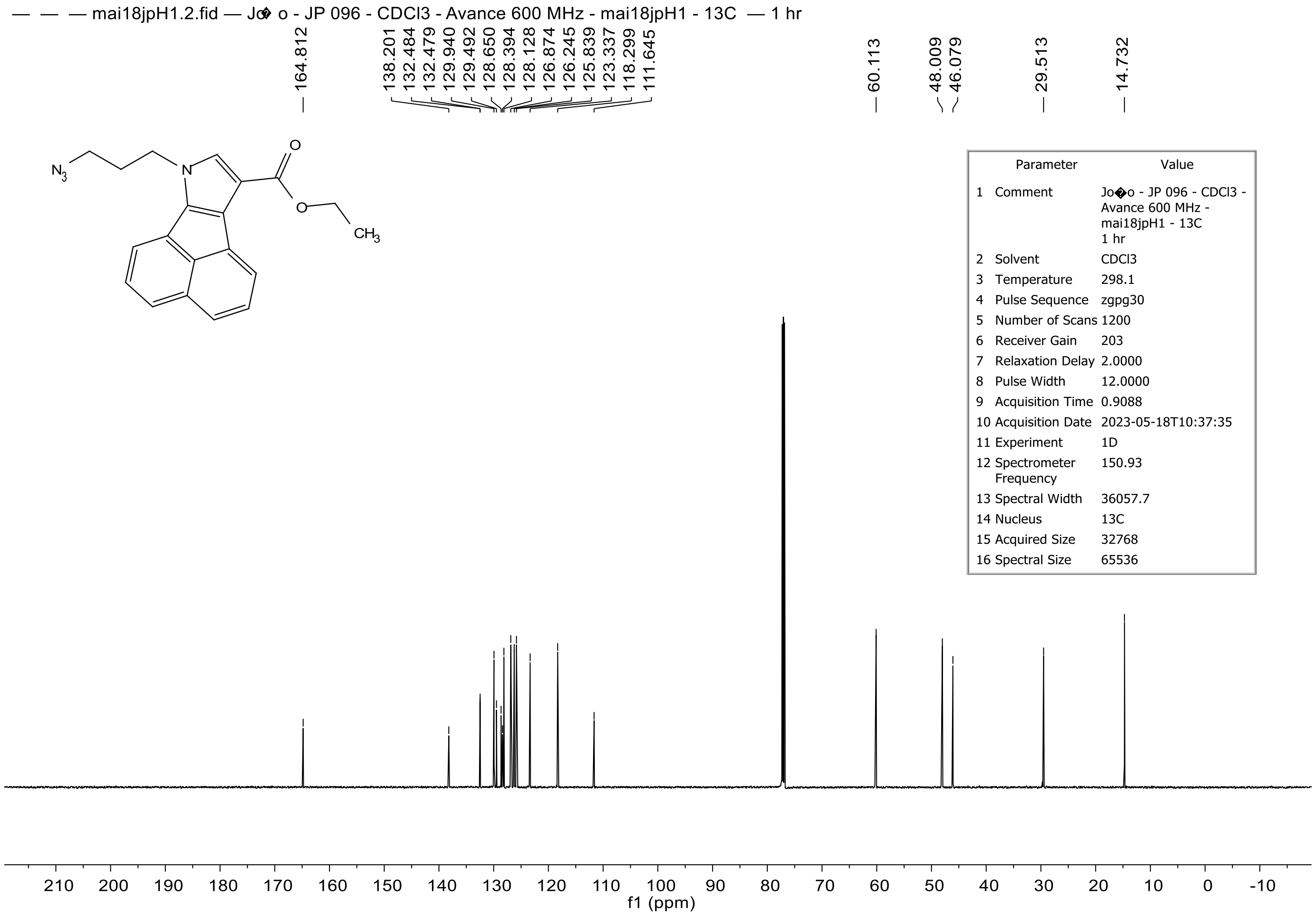

Figure S34 – ^{13}C NMR, 150 Hz, $CDCl_3$ (compound **4n**).

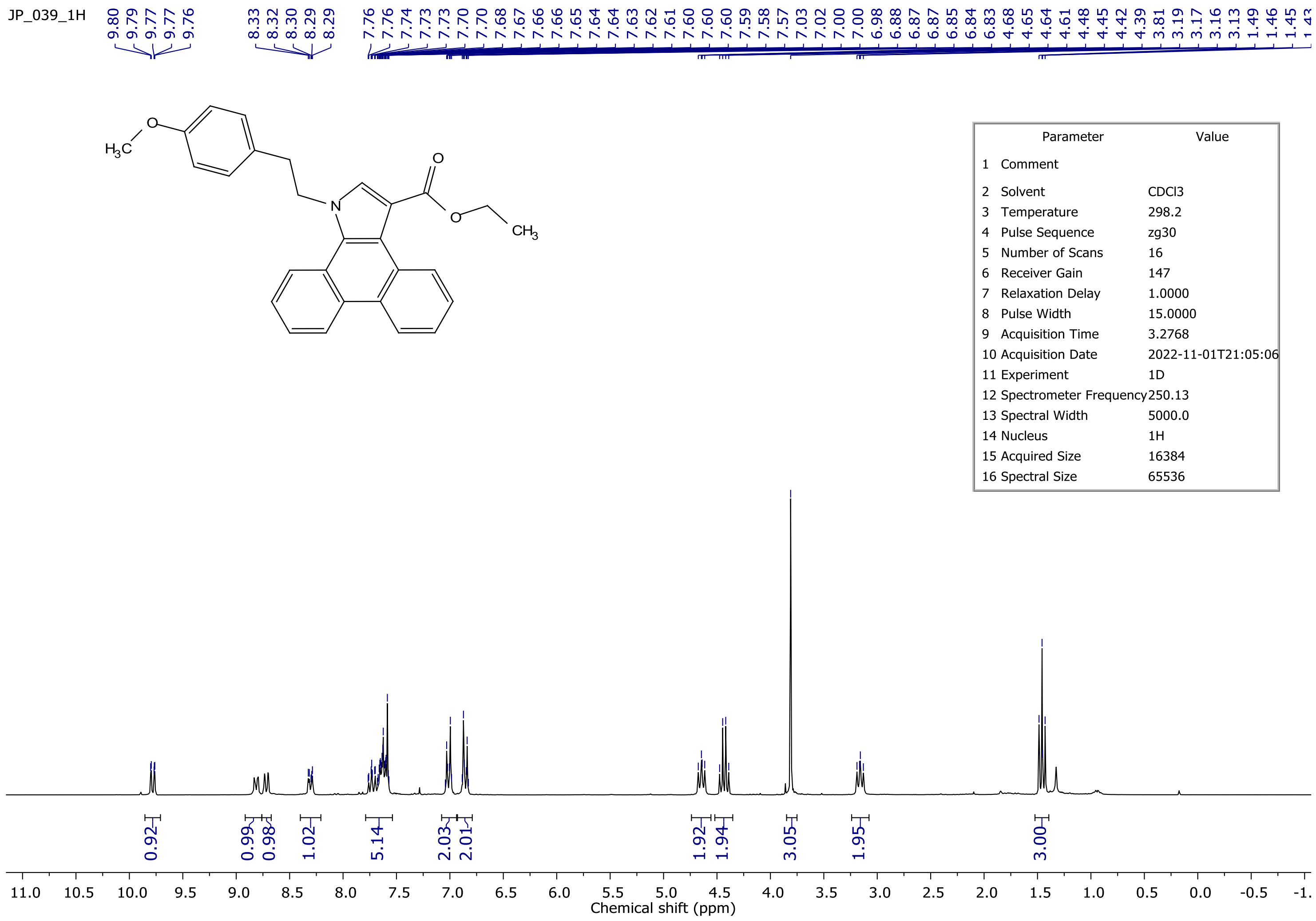

Figure S35 – ^{1}H NMR, 250 Hz, $CDCl_3$ (compound **5a**).

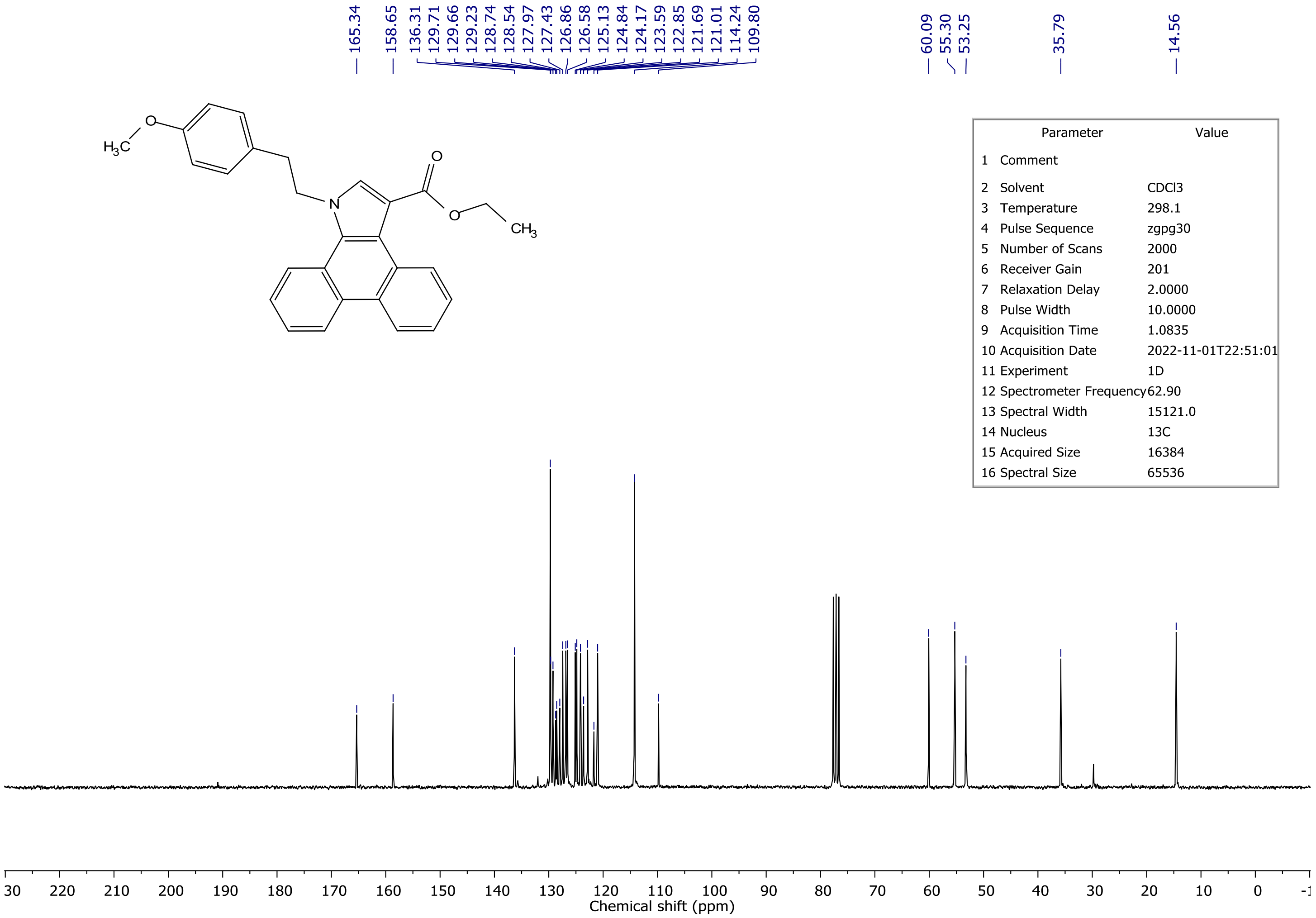

Figure S36 – ^{13}C NMR, 63 Hz, $CDCl_3$ (compound **5a**).

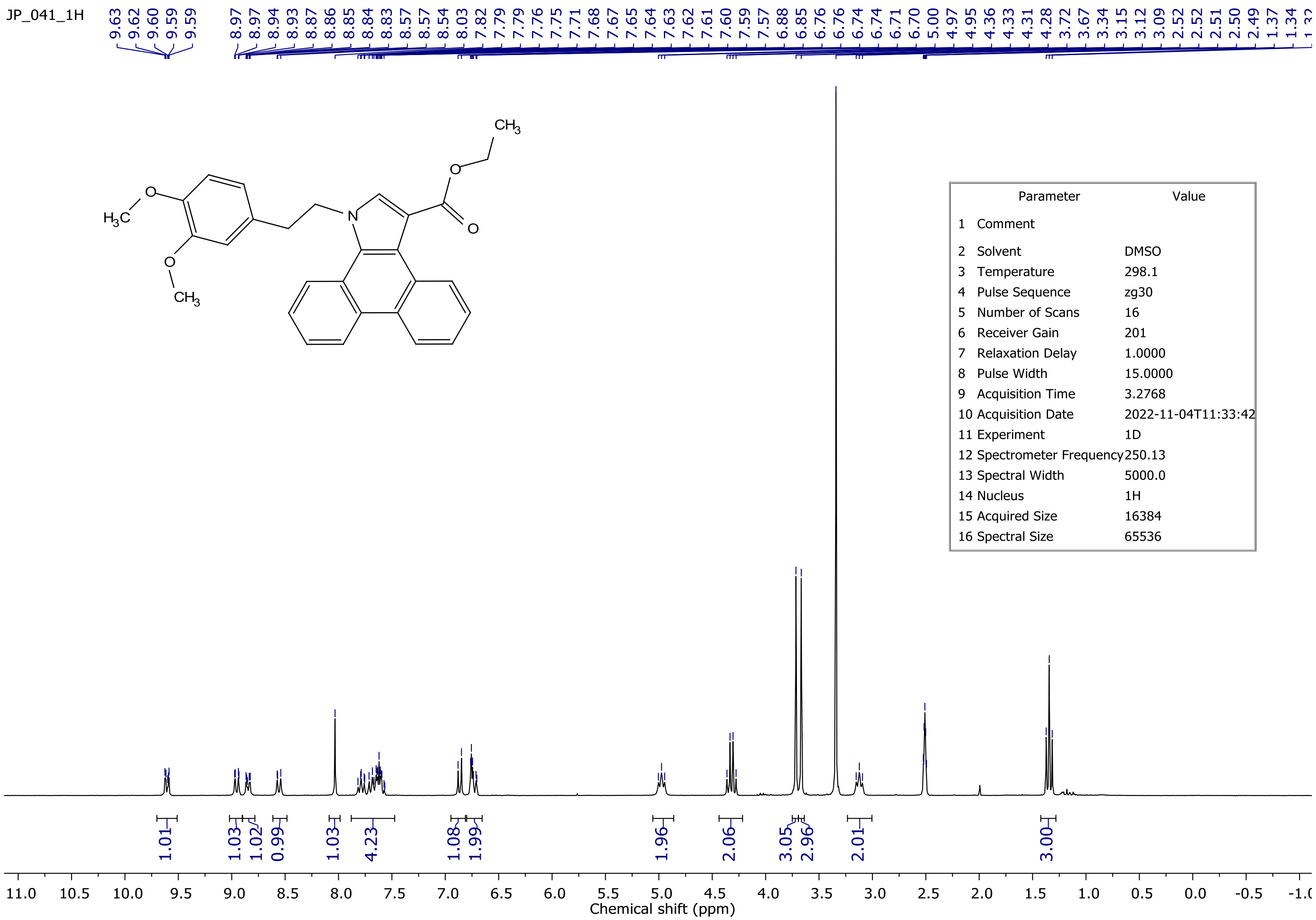

Figure S37 – ^{1}H NMR, 250 Hz, DMSO-d_6 (compound **5b**).

JP_041_13C

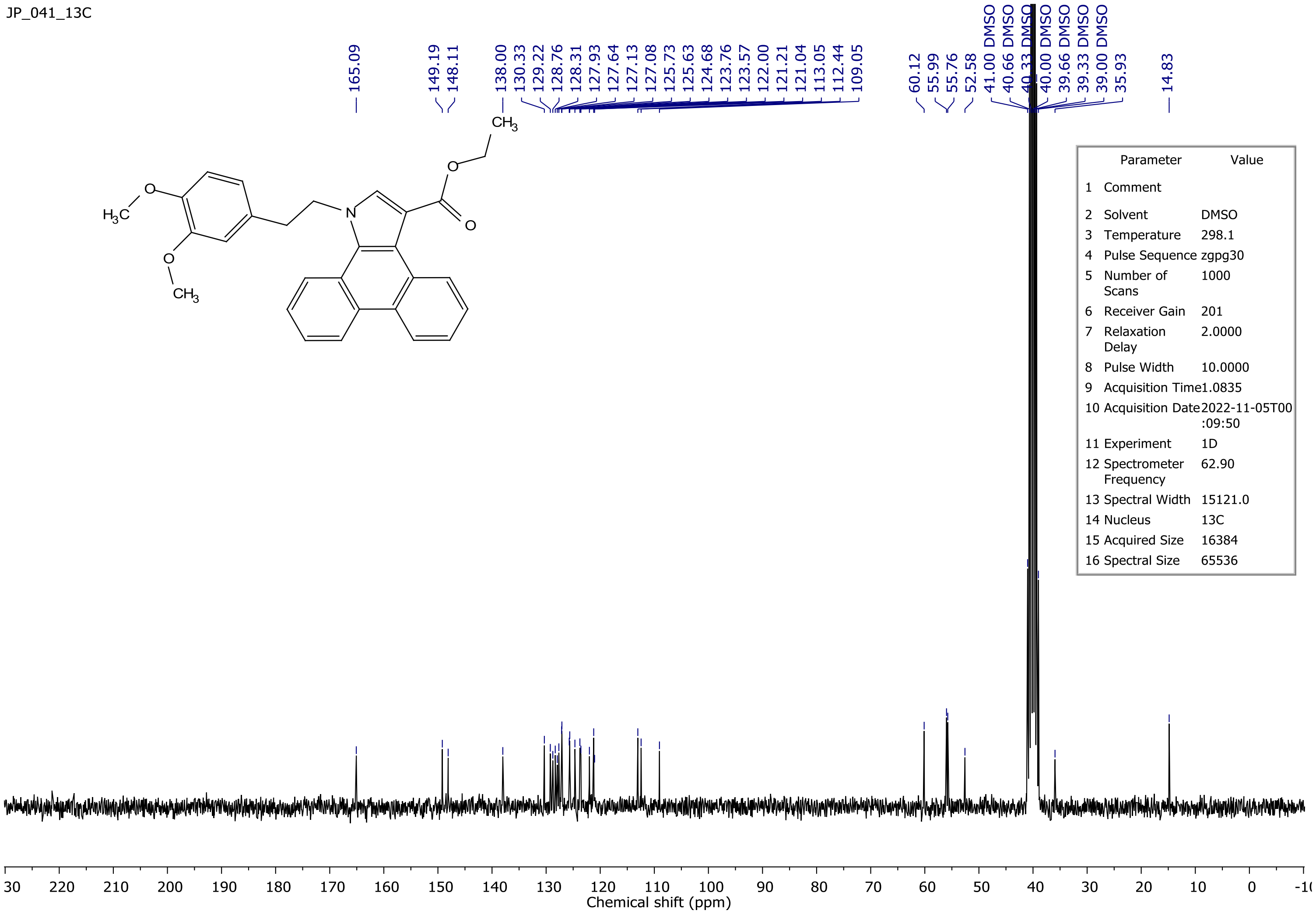

Parameter	Value
1 Comment	
2 Solvent	DMSO
3 Temperature	298.1
4 Pulse Sequence	zgpg30
5 Number of Scans	1000
6 Receiver Gain	201
7 Relaxation Delay	2.0000
8 Pulse Width	10.0000
9 Acquisition Time	1.0835
10 Acquisition Date	2022-11-05T00:09:50
11 Experiment	1D
12 Spectrometer Frequency	62.90
13 Spectral Width	15121.0
14 Nucleus	13C
15 Acquired Size	16384
16 Spectral Size	65536

Figure S38 – ^{13}C NMR, 63 Hz, DMSO-d$_6$ (compound **5b**).

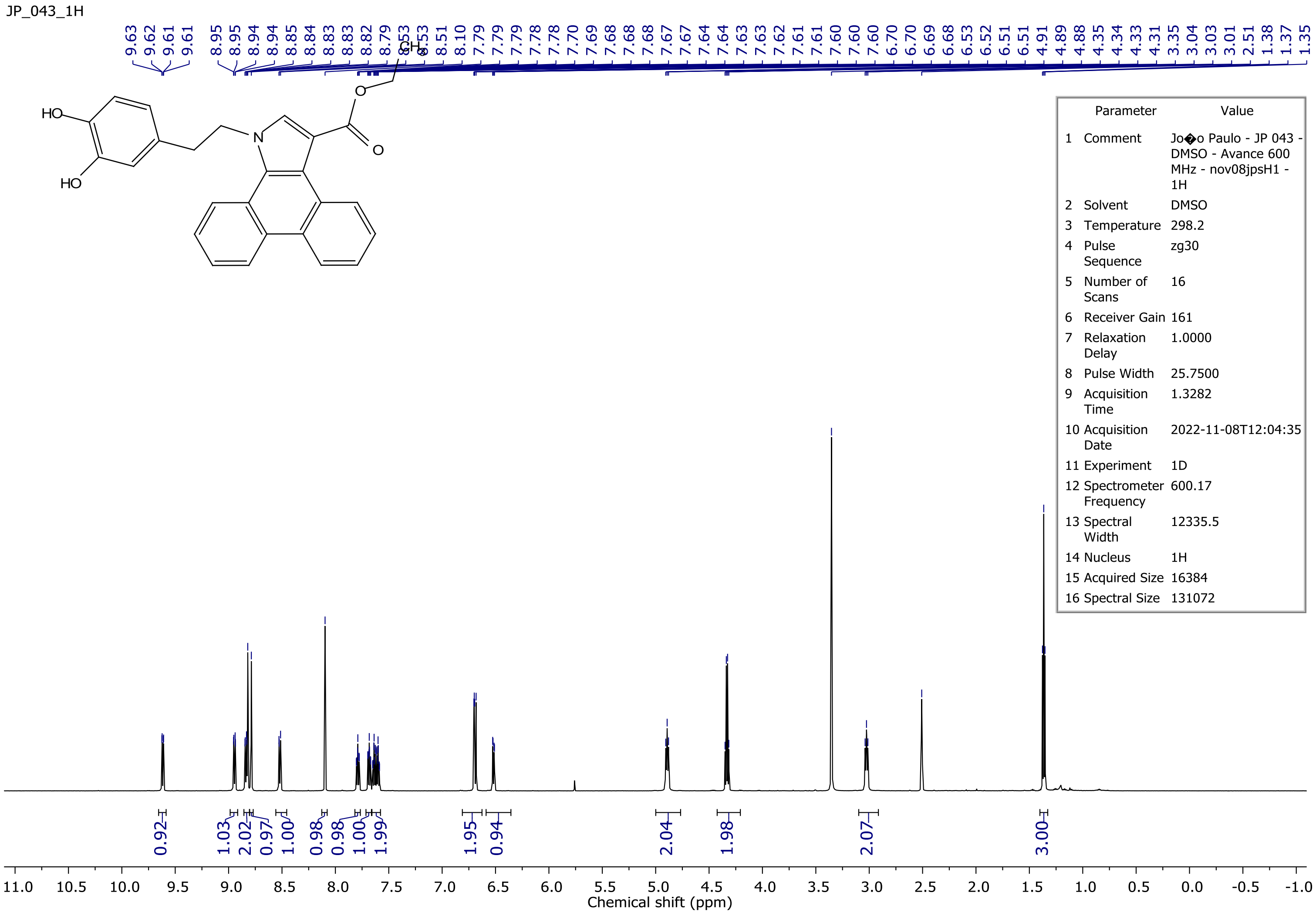

Figure S39 – ^{1}H NMR, 250 Hz, DMSO-d$_6$ (compound **5c**).

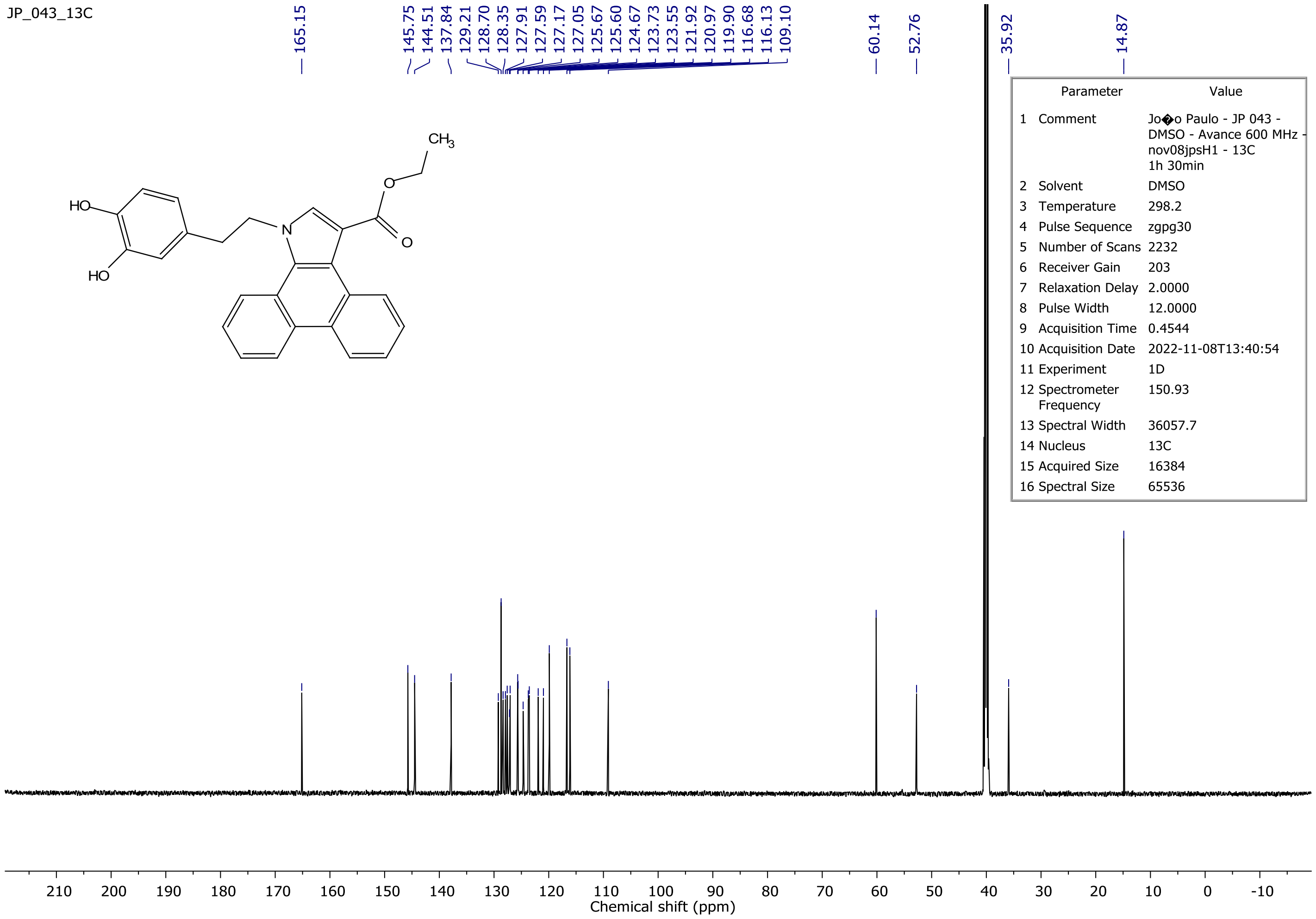

Figure S40 – ^{13}C NMR, 63 Hz, DMSO-d_6 (compound **5c**).

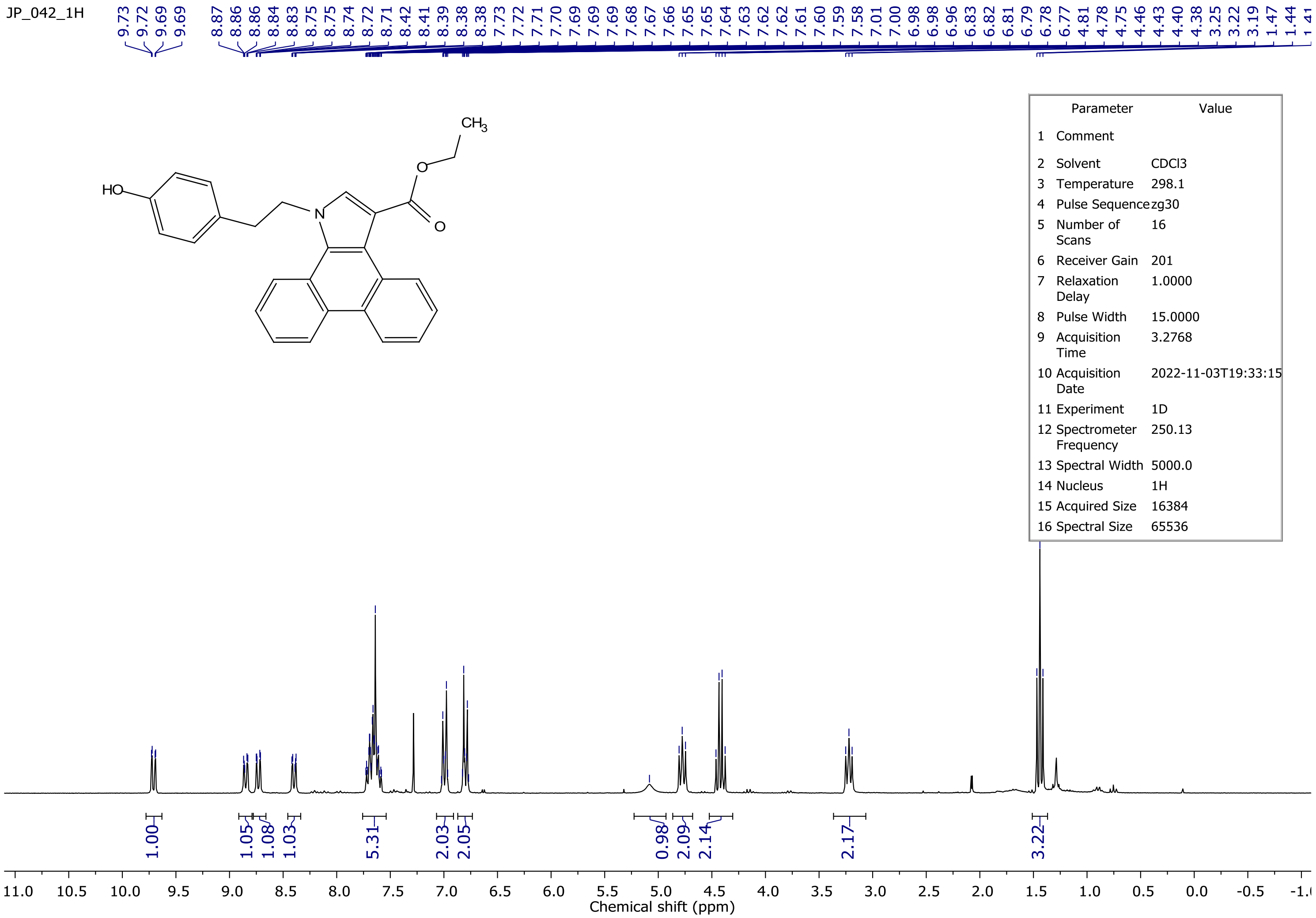

Figure S41 – ^{1}H NMR, 250 Hz, $CDCl_3$ (compound **5d**).

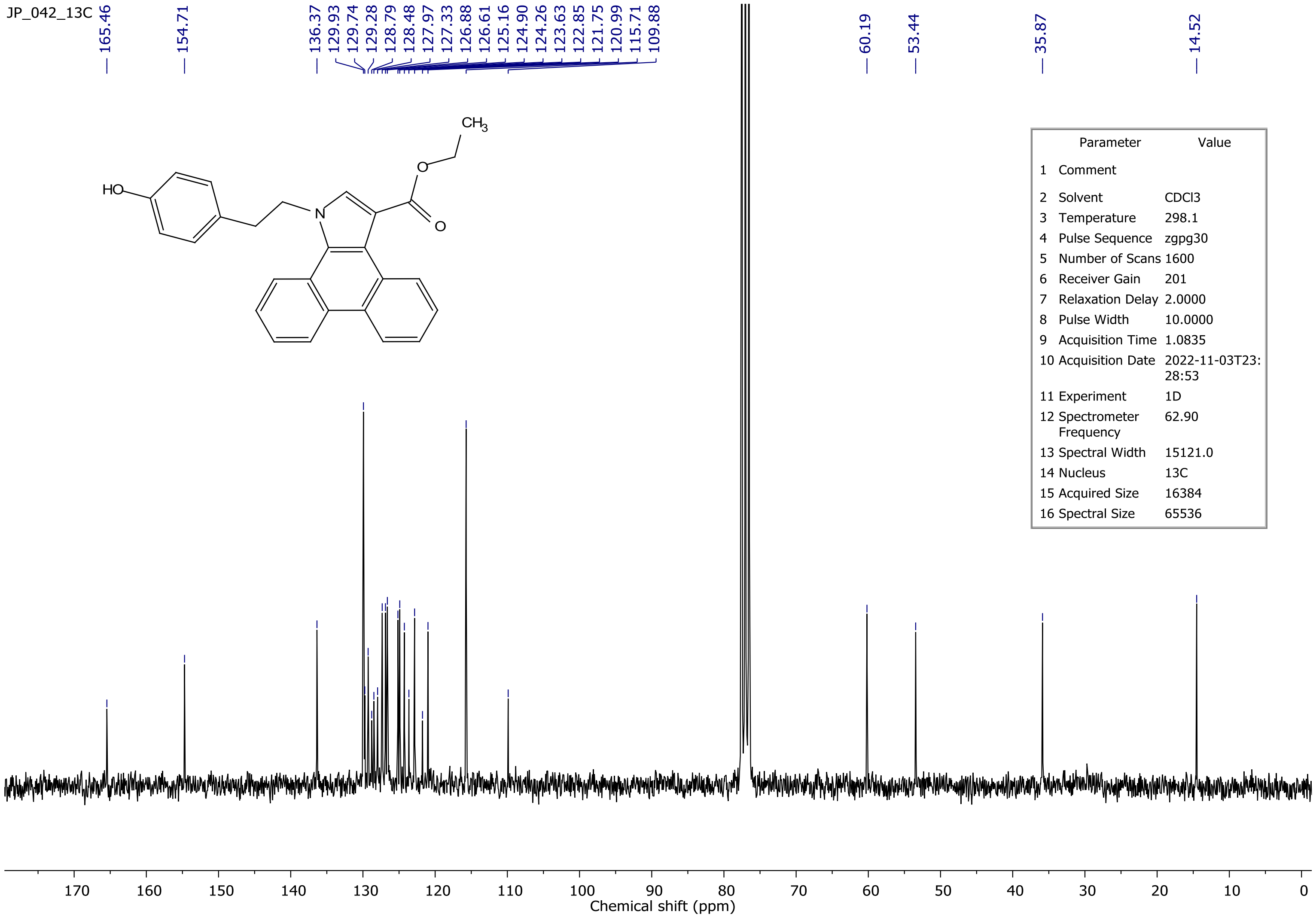

Figure S42 – ^{13}C NMR, 63 Hz, $CDCl_3$ (compound **5d**).

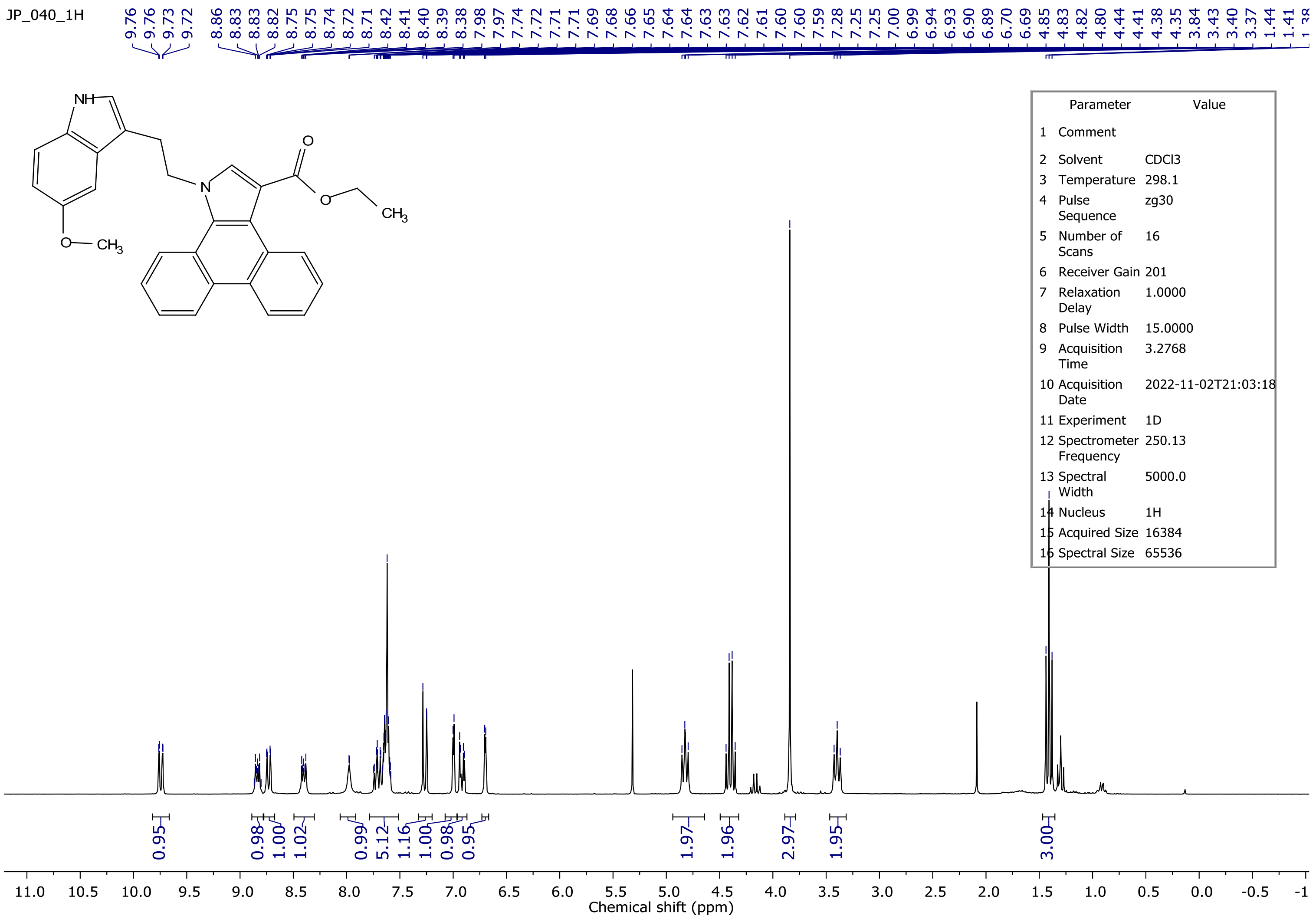

Figure S43 – ^{1}H NMR, 250 Hz, $CDCl_3$ (compound **5e**).

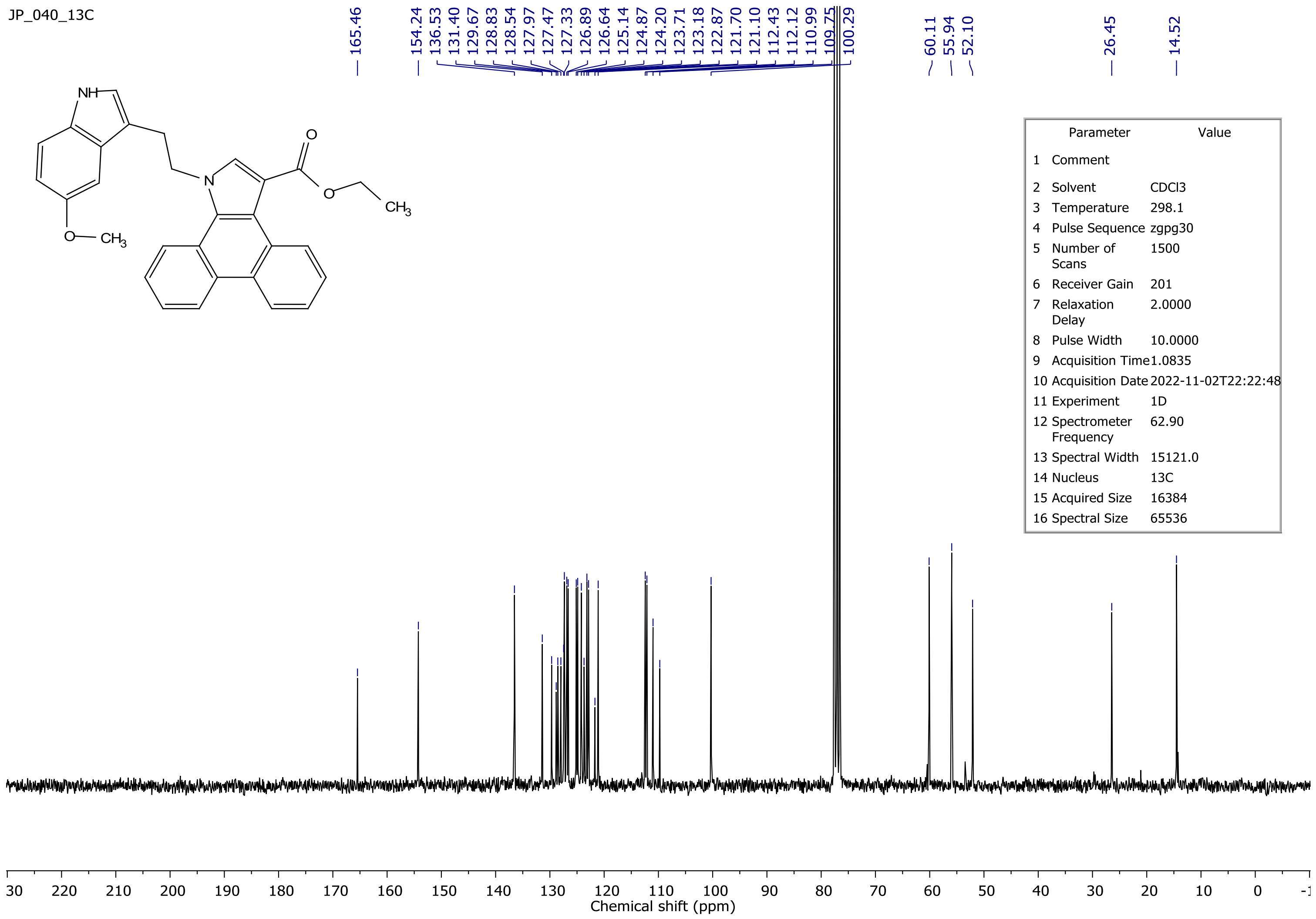

Figure S44 – ^{13}C NMR, 63 Hz, $CDCl_3$ (compound **5e**).

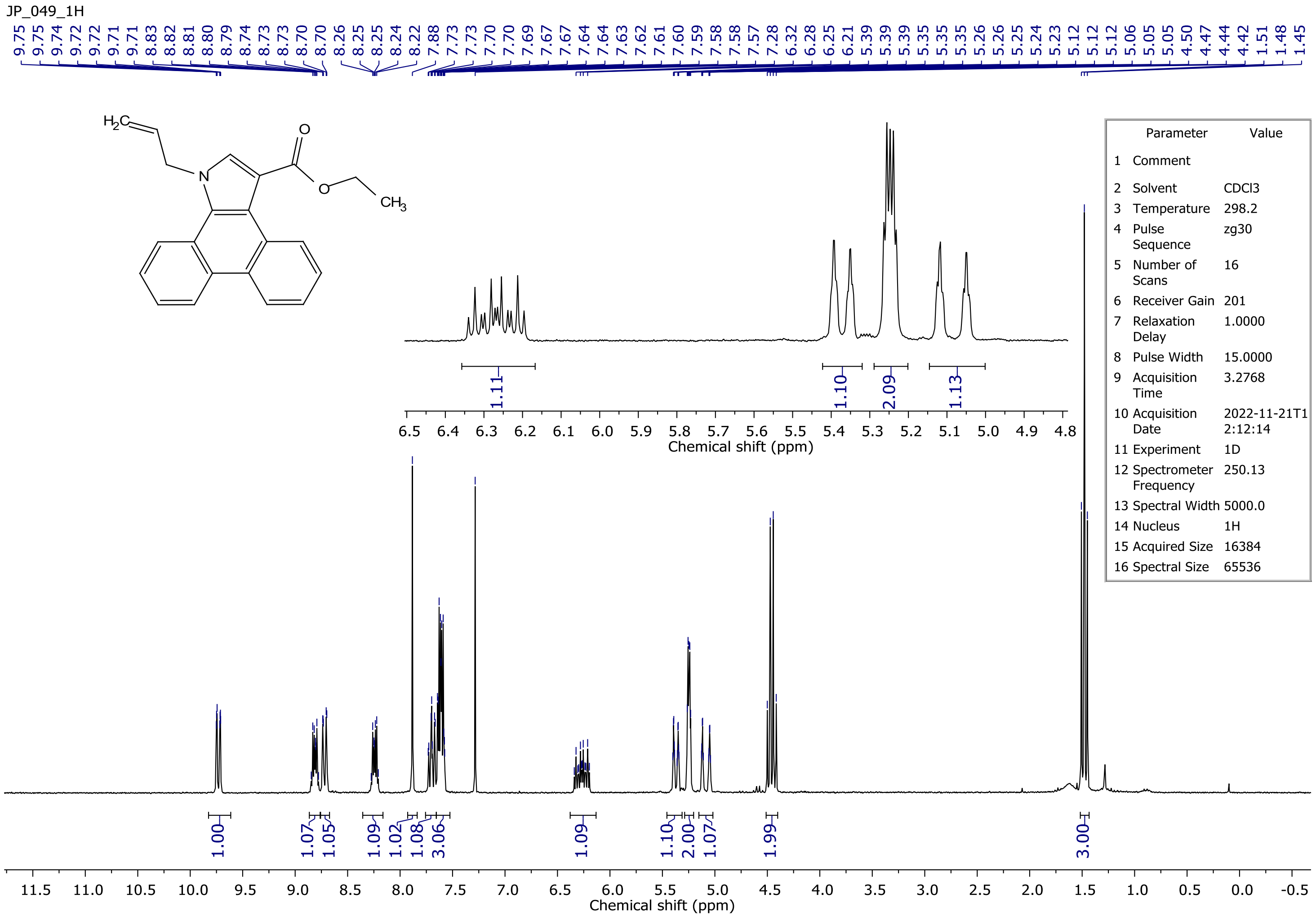

Figure S45 – ^{1}H NMR, 250 Hz, $CDCl_3$ (compound **5f**).

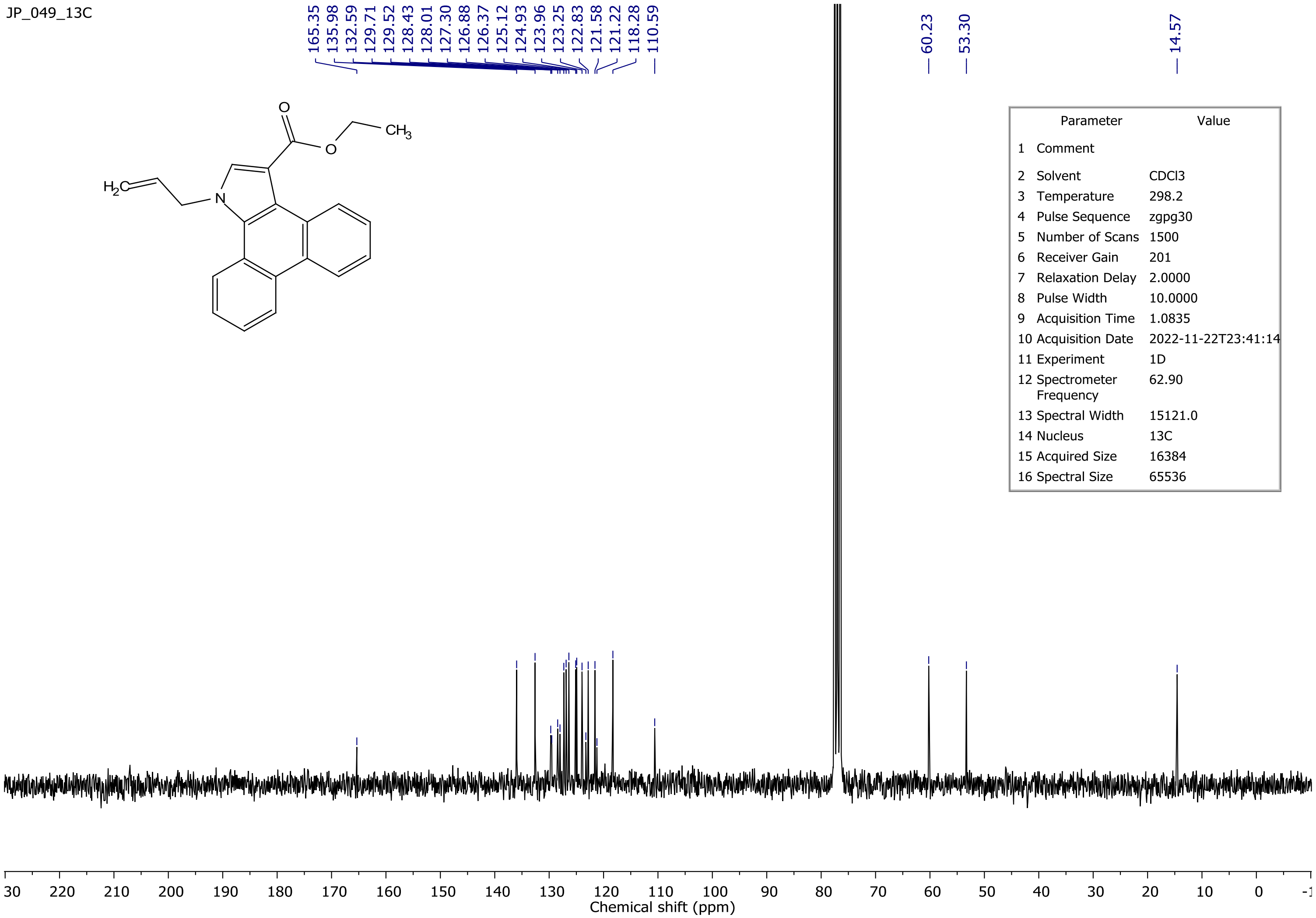

Figure S46 – ^{13}C NMR, 63 Hz, $CDCl_3$ (compound **5f**).

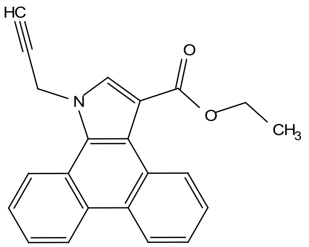

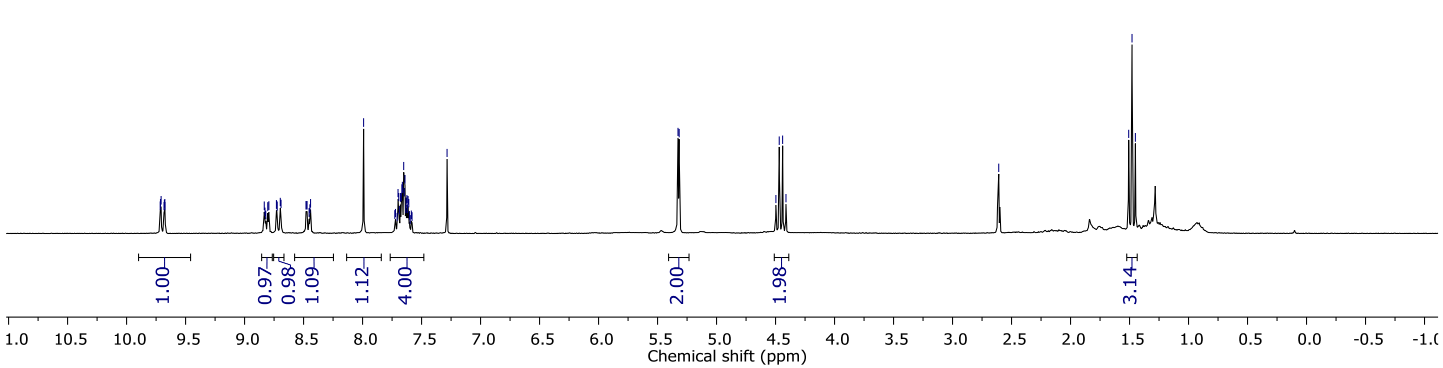

	Parameter	Value
1	Comment	
2	Solvent	CDCl3
3	Temperature	298.1
4	Pulse Sequence	zg30
5	Number of Scans	16
6	Receiver Gain	201
7	Relaxation Delay	1.0000
8	Pulse Width	15.0000
9	Acquisition Time	3.2768
10	Acquisition Date	2022-11-21T12:06:30
11	Experiment	1D
12	Spectrometer Frequency	250.13
13	Spectral Width	5000.0
14	Nucleus	1H
15	Acquired Size	16384
16	Spectral Size	65536

Figure S47 – ^{1}H NMR, 250 Hz, $CDCl_3$ (compound **5g**).

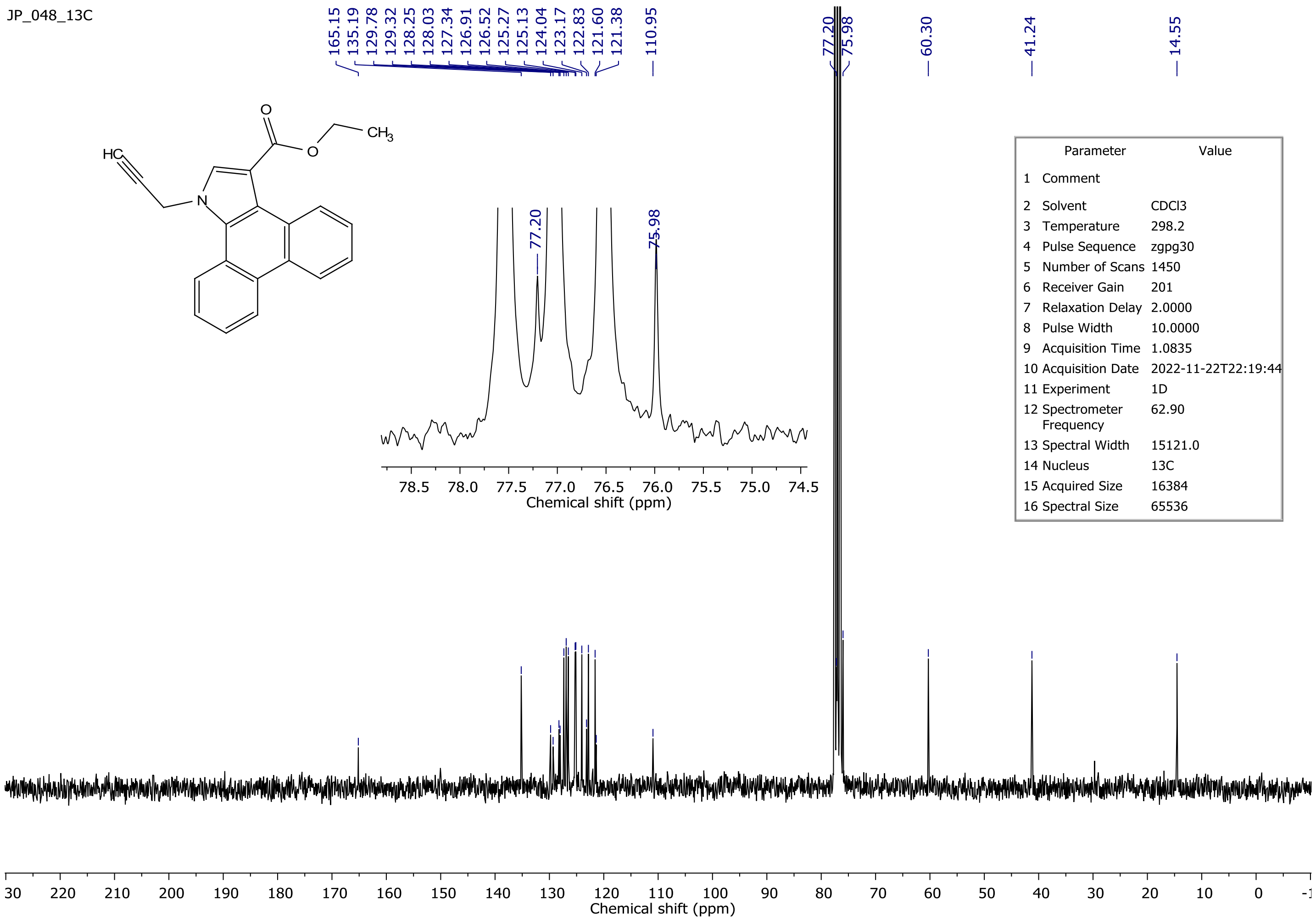

Figure S48 – ^{13}C NMR, 63 Hz, $CDCl_3$ (compound **5g**).

— — — jan31jpoH1.1.fid — Jo� o - JP059 - CDCl3 - Avance 500 MHz - jan31jpoH1

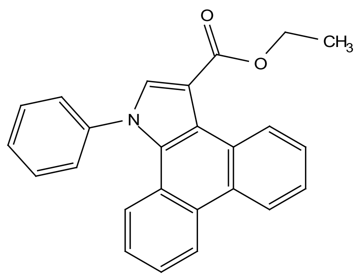

	Parameter	Value
1	Comment	Jo�o - JP059 - CDCl3 - Avance 500 MHz - jan31jpoH1
2	Solvent	CDCl3
3	Temperature	298.1
4	Pulse Sequence	zg30
5	Number of Scans	16
6	Receiver Gain	203
7	Relaxation Delay	1.0000
8	Pulse Width	12.4000
9	Acquisition Time	3.1807
10	Acquisition Date	2023-02-01T05:54:53
11	Experiment	1D
12	Spectrometer Frequency	499.87
13	Spectral Width	10302.2
14	Nucleus	1H
15	Acquired Size	32768
16	Spectral Size	65536

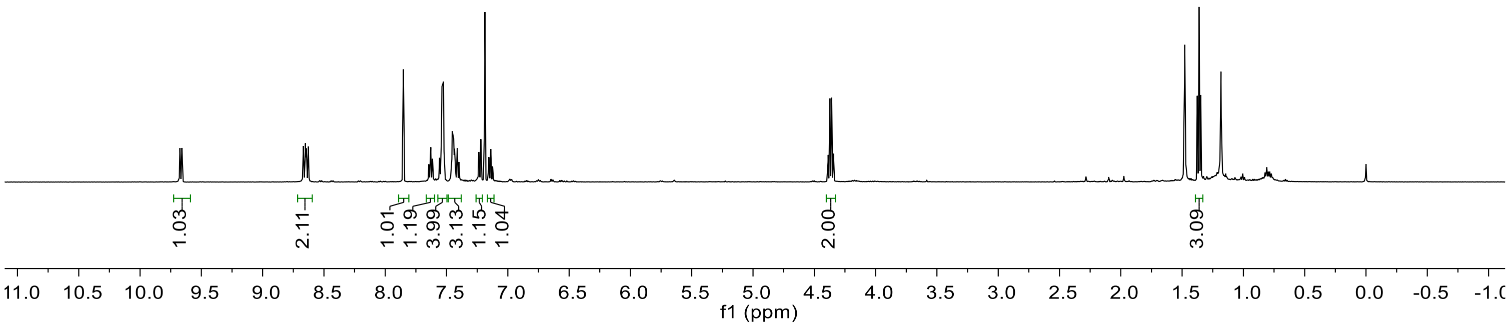

Figure S49 – ^{1}H NMR, 500 Hz, $CDCl_3$ (compound **5h**).

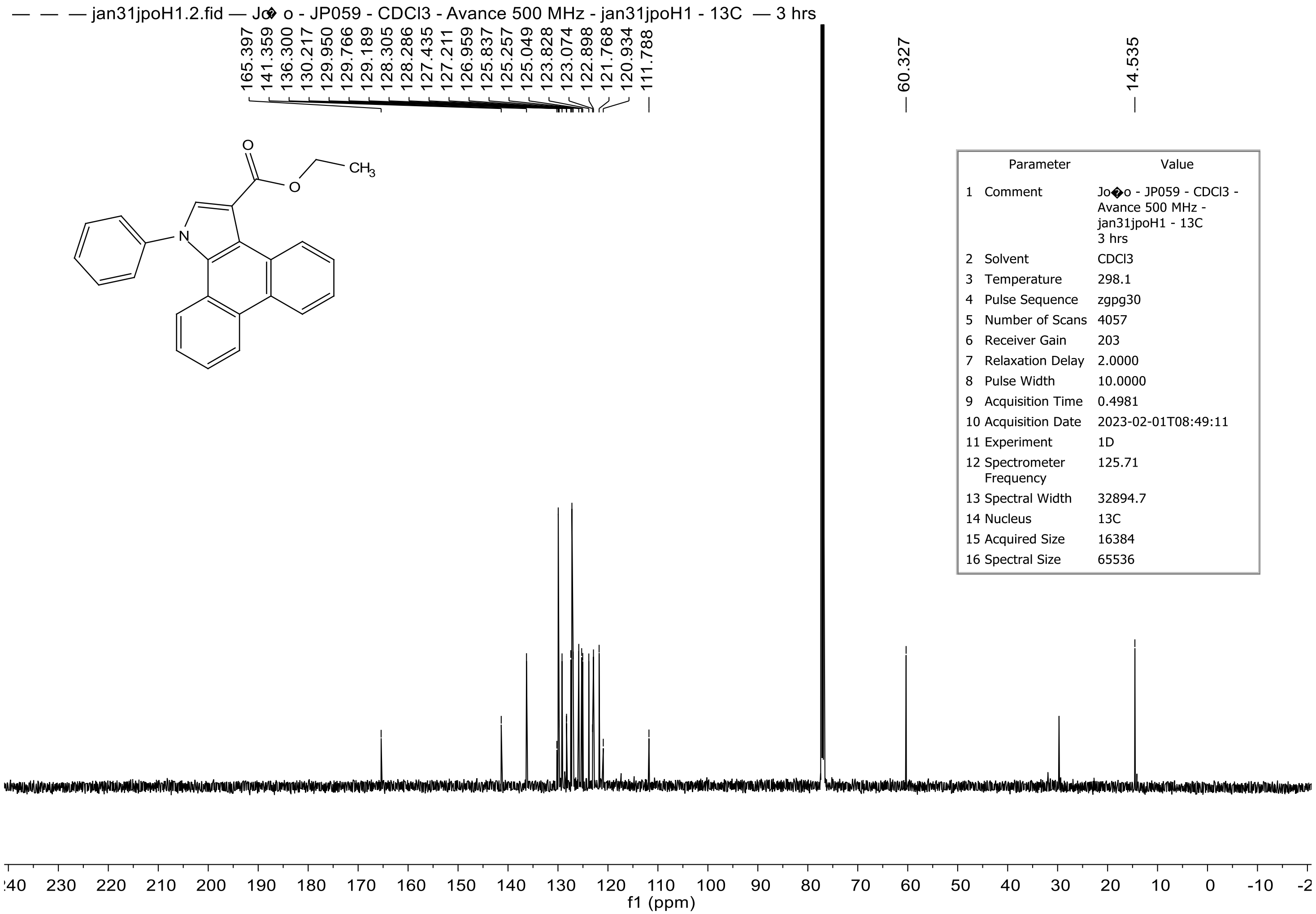

Figure S50 – ^{13}C NMR, 150 Hz, $CDCl_3$ (compound **5g**).

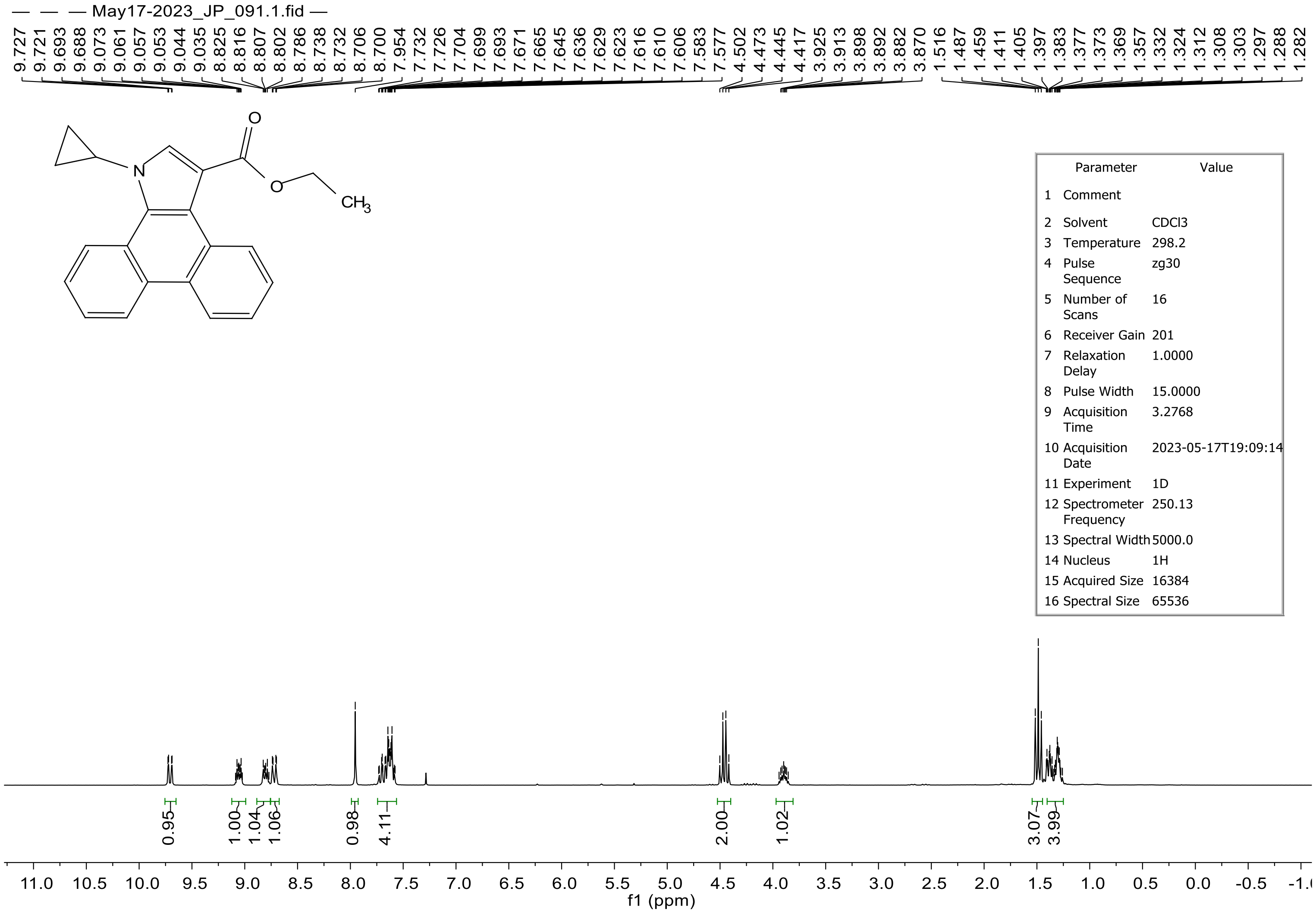

Figure S51 – ^{1}H NMR, 250 Hz, $CDCl_3$ (compound **5j**).

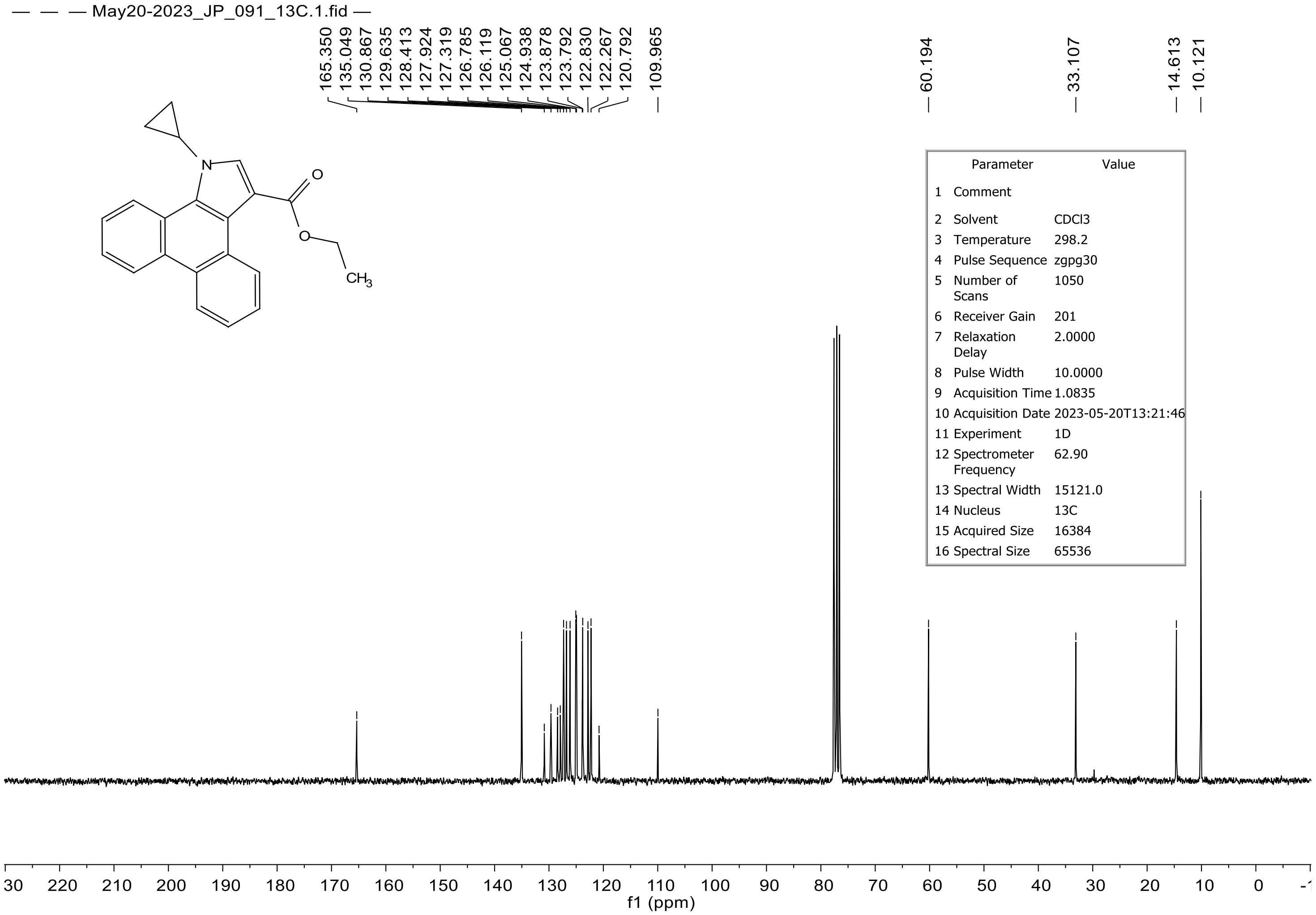

Figure S52 – ^{13}C NMR, 63 Hz, $CDCl_3$ (compound **5j**).

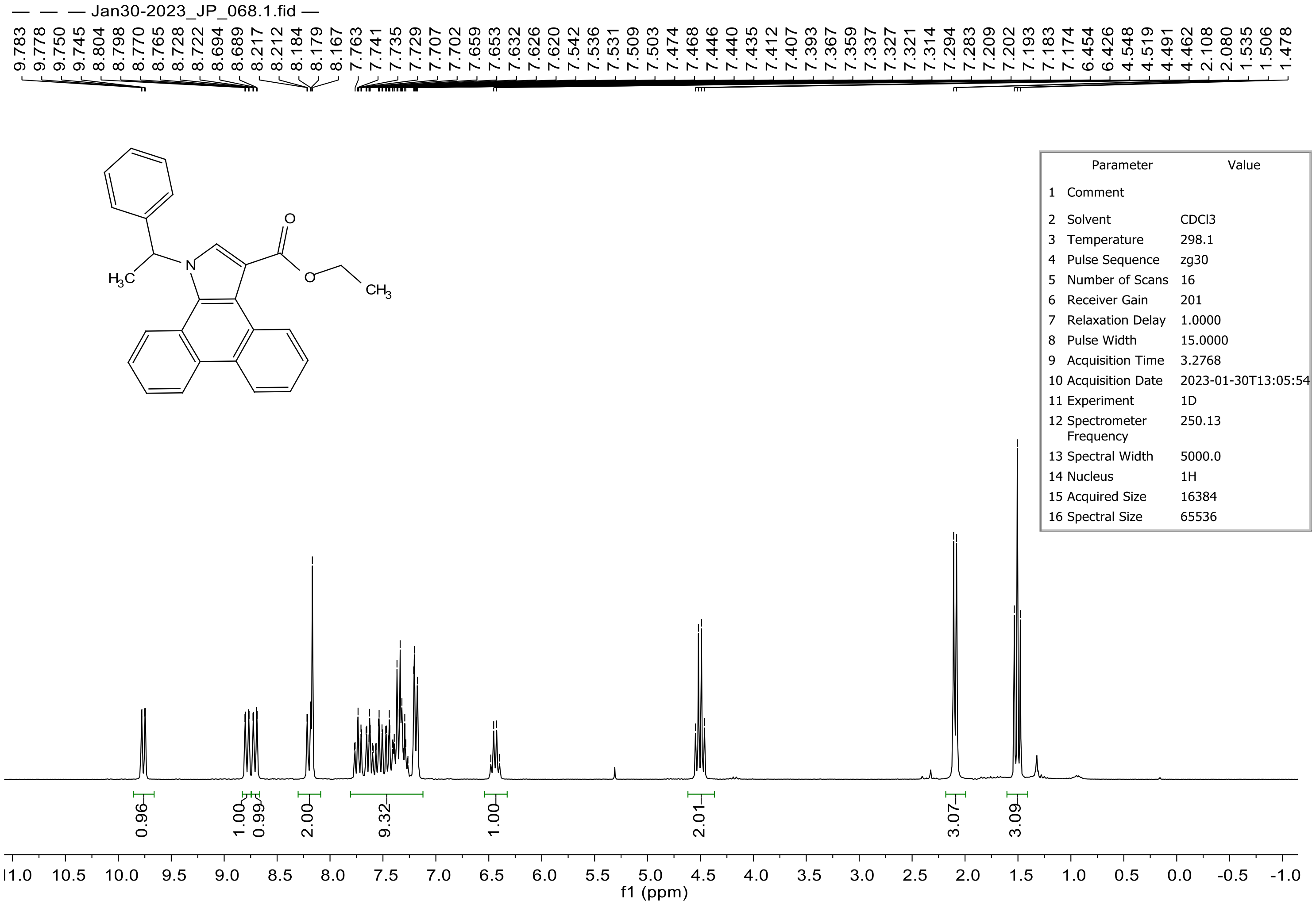

Figure S53 – ^{1}H NMR, 250 Hz, $CDCl_3$ (compound **5k**).

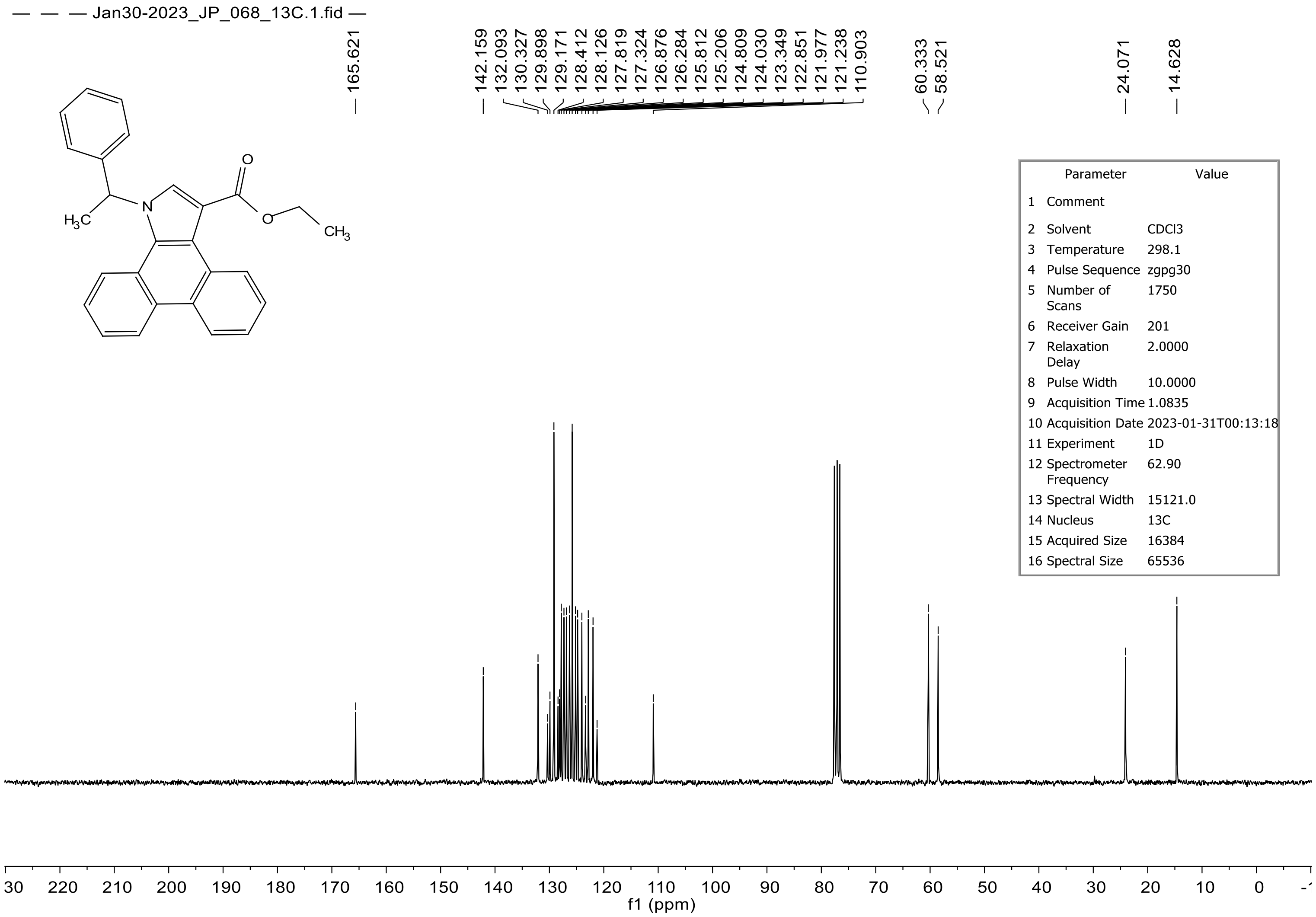

Figure S54 – ^{13}C NMR, 63 Hz, $CDCl_3$ (compound **5k**).

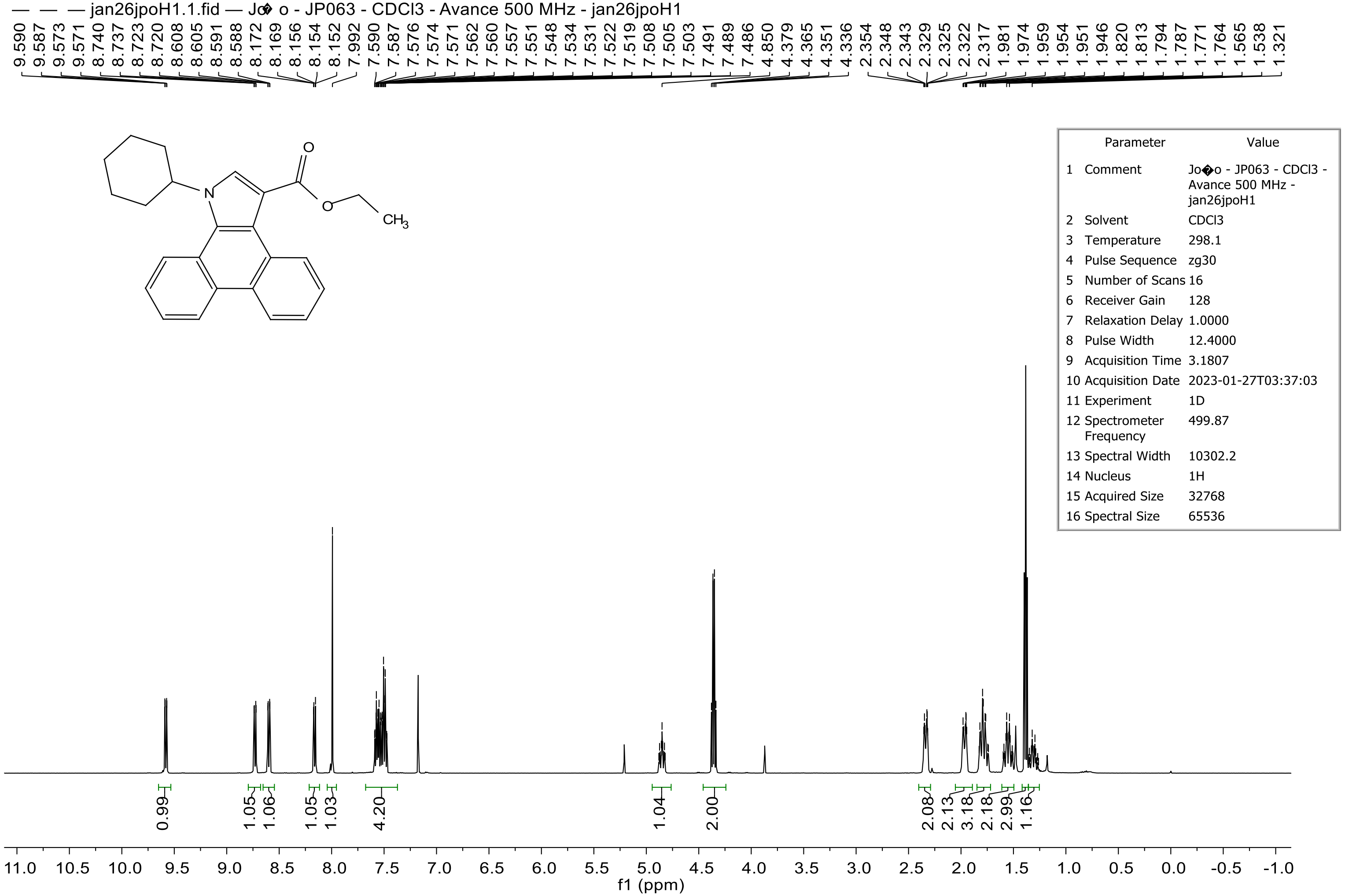

Parameter	Value
1 Comment	Jo�o - JP063 - CDCl3 - Avance 500 MHz - jan26jpoH1
2 Solvent	CDCl3
3 Temperature	298.1
4 Pulse Sequence	zg30
5 Number of Scans	16
6 Receiver Gain	128
7 Relaxation Delay	1.0000
8 Pulse Width	12.4000
9 Acquisition Time	3.1807
10 Acquisition Date	2023-01-27T03:37:03
11 Experiment	1D
12 Spectrometer Frequency	499.87
13 Spectral Width	10302.2
14 Nucleus	1H
15 Acquired Size	32768
16 Spectral Size	65536

Figure S55 – ^{1}H NMR, 500 Hz, $CDCl_3$ (compound **5m**).

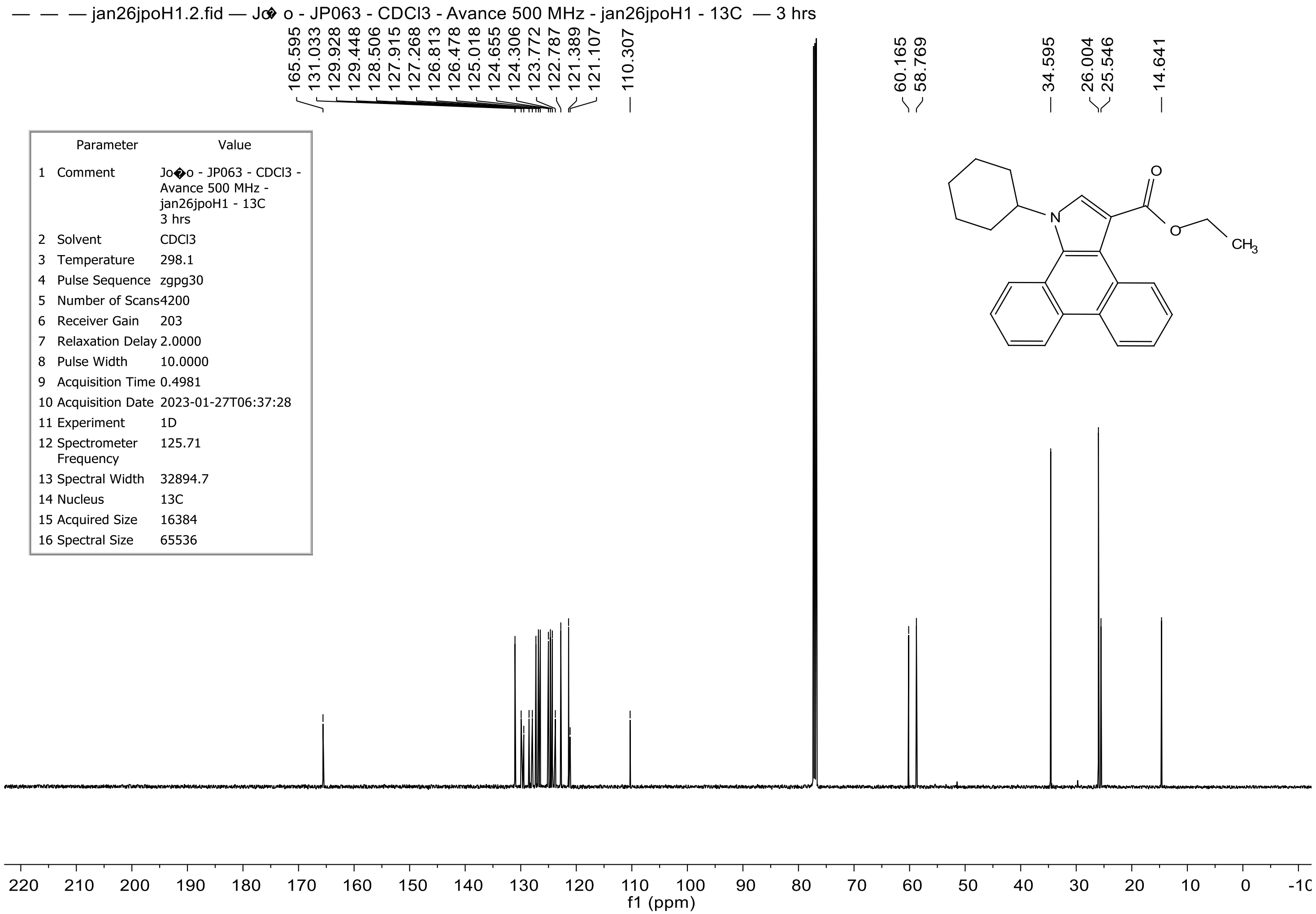

Figure S56 – ^{13}C NMR, 150 Hz, $CDCl_3$ (compound **5m**).

— — — May20-2023_JP_097.1.fid —

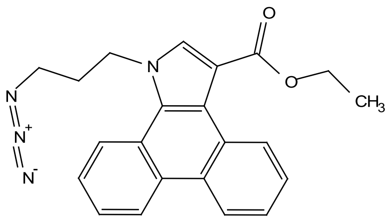

	Parameter	Value
1	Comment	
2	Solvent	CDCl3
3	Temperature	298.1
4	Pulse Sequence	zg30
5	Number of Scans	16
6	Receiver Gain	180
7	Relaxation Delay	1.0000
8	Pulse Width	15.0000
9	Acquisition Time	3.2768
10	Acquisition Date	2023-05-20T11:26:41
11	Experiment	1D
12	Spectrometer Frequency	250.13
13	Spectral Width	5000.0
14	Nucleus	1H
15	Acquired Size	16384
16	Spectral Size	65536

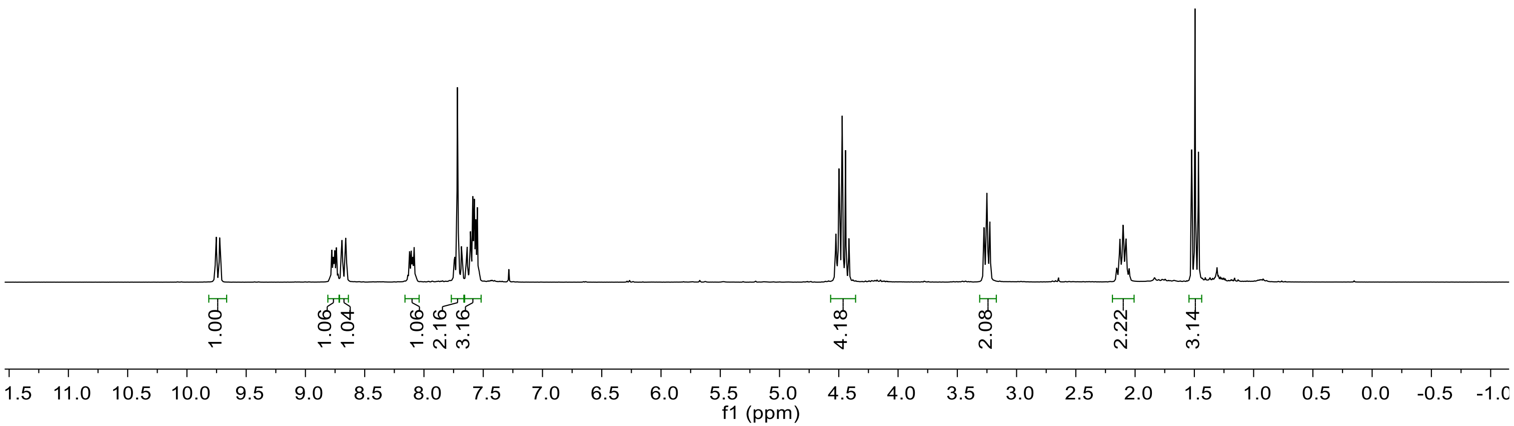

Figure S57 – ^{1}H NMR, 250 Hz, $CDCl_3$ (compound **5n**).

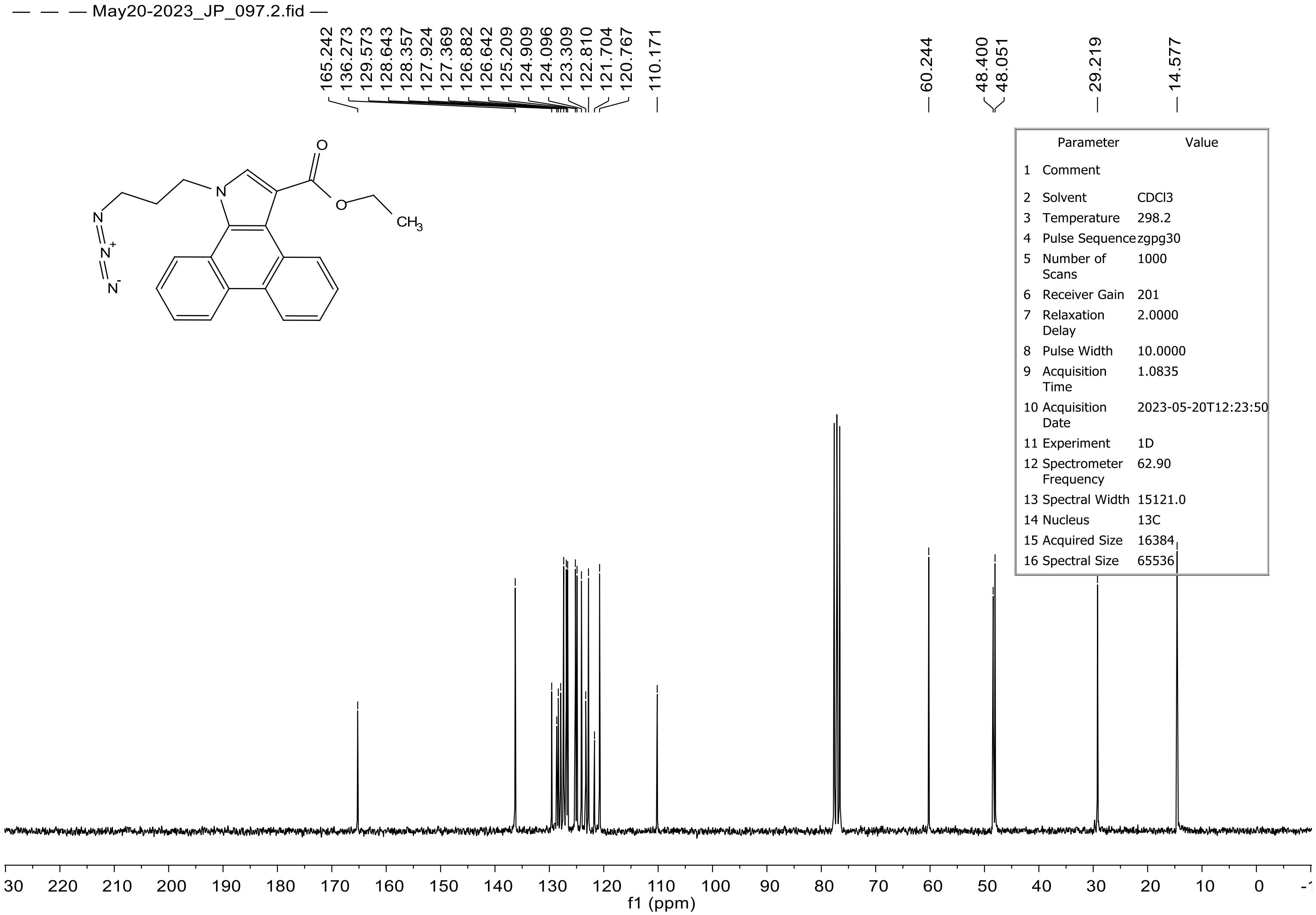

Figure S58 – ^{13}C NMR, 63 Hz, $CDCl_3$ (compound **5n**).

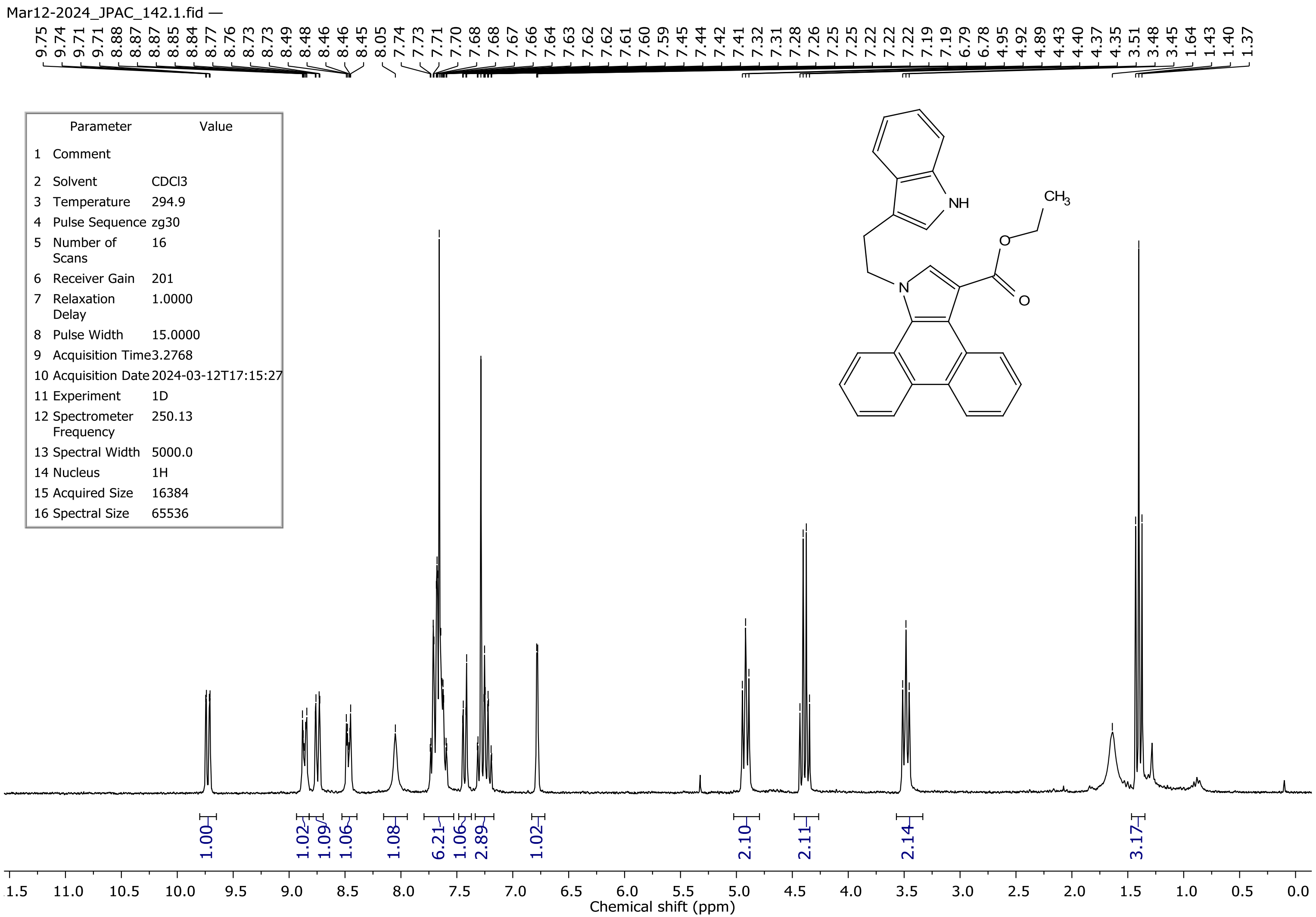

Figure S59 – ^{1}H NMR, 250 Hz, $CDCl_3$ (compound **5o**).

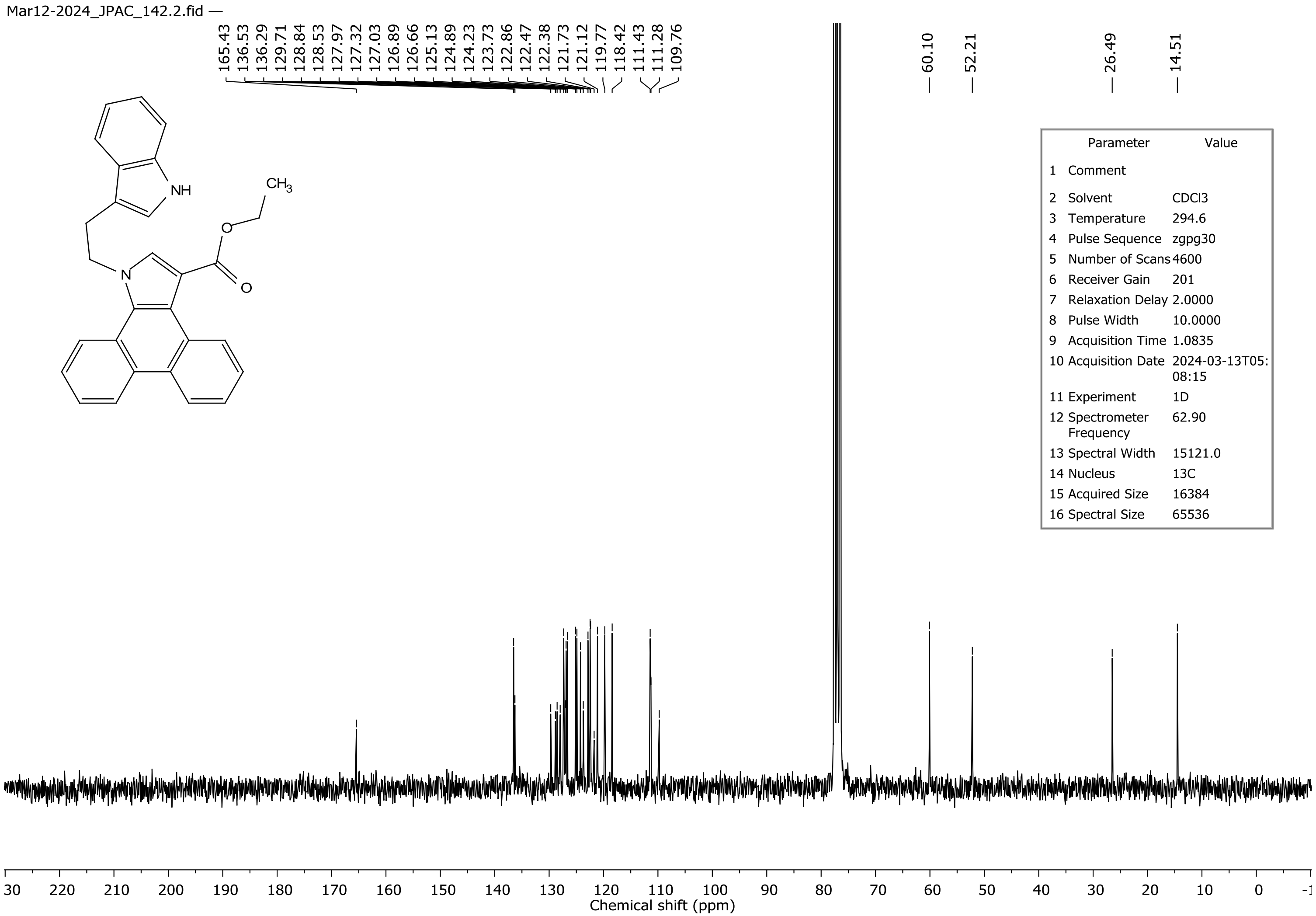

Figure S60 – ^{13}C NMR, 63 Hz, $CDCl_3$ (compound **5o**).

— — — abr28jpH1.1.fid — Jo� o Paulo - JP-089 - CDCl3 - Avance 500 MHz - abr28jpH1 - 1H

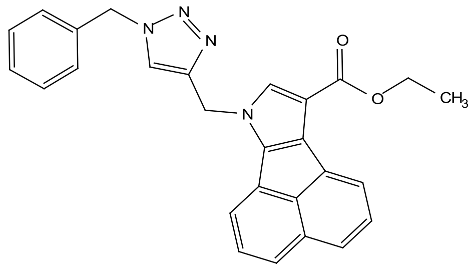

	Parameter	Value
1	Comment	Jo�o Paulo - JP-089 - CDCl3 - Avance 500 MHz - abr28jpH1 - 1H
2	Solvent	CDCl3
3	Temperature	298.1
4	Pulse Sequence	zg30
5	Number of Scans	16
6	Receiver Gain	128
7	Relaxation Delay	1.0000
8	Pulse Width	12.4000
9	Acquisition Time	3.1807
10	Acquisition Date	2023-04-29T00:25:22
11	Experiment	1D
12	Spectrometer Frequency	499.87
13	Spectral Width	10302.2
14	Nucleus	1H
15	Acquired Size	32768
16	Spectral Size	65536

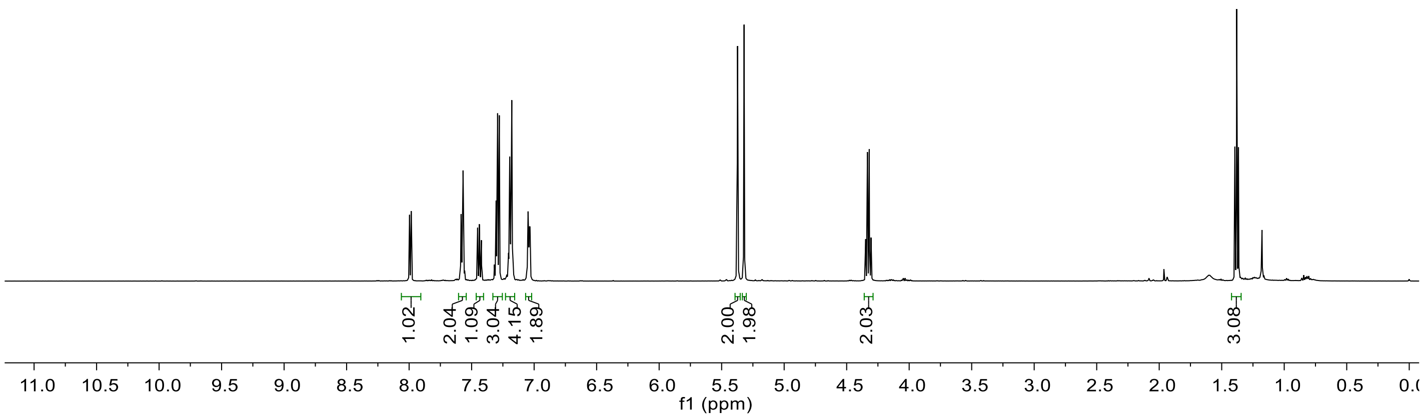

Figure S61 – ^{1}H NMR, 500 Hz, $CDCl_3$ (compound **6a**).

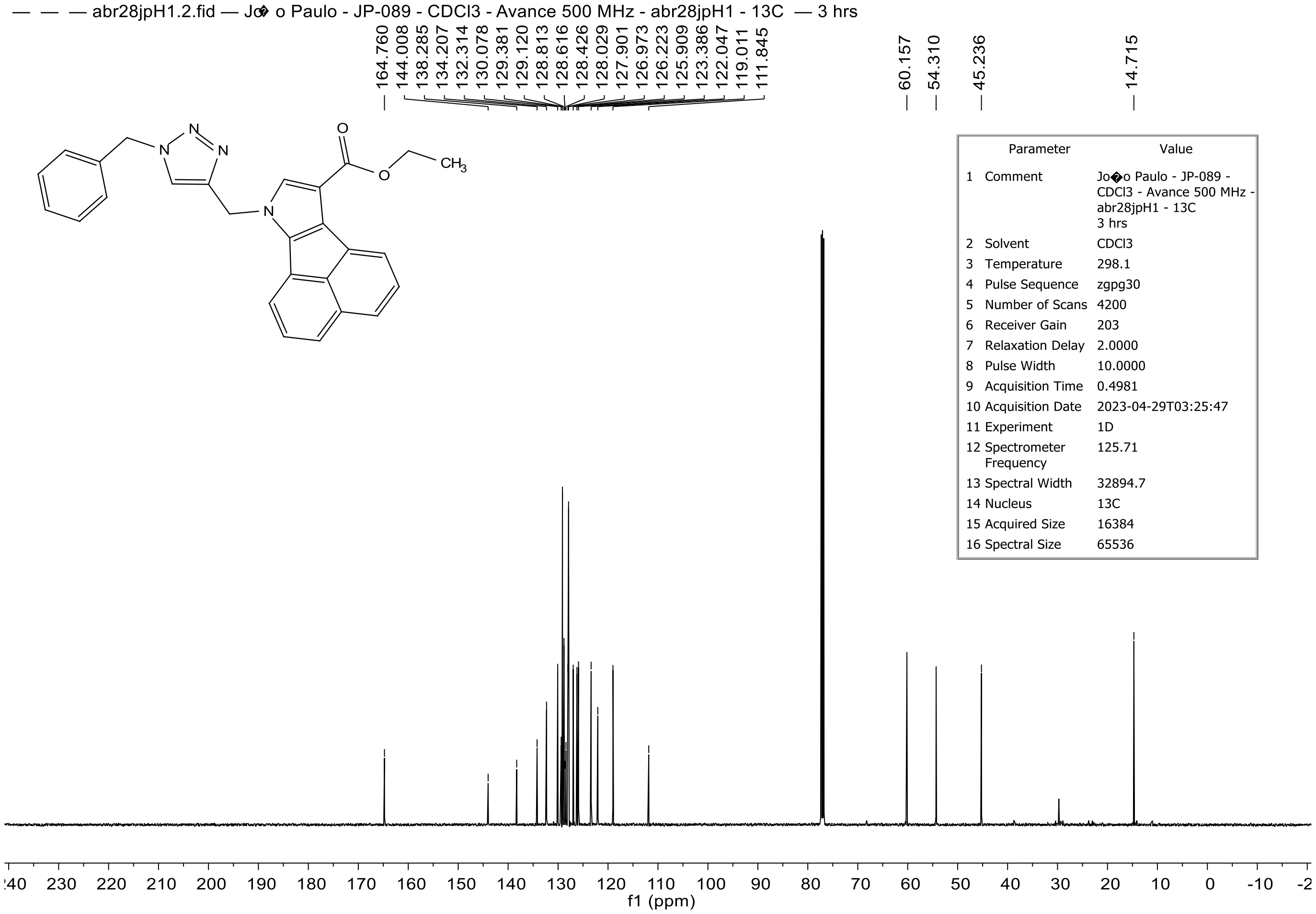

Figure S62 – ^{13}C NMR, 150 Hz, $CDCl_3$ (compound **6a**).

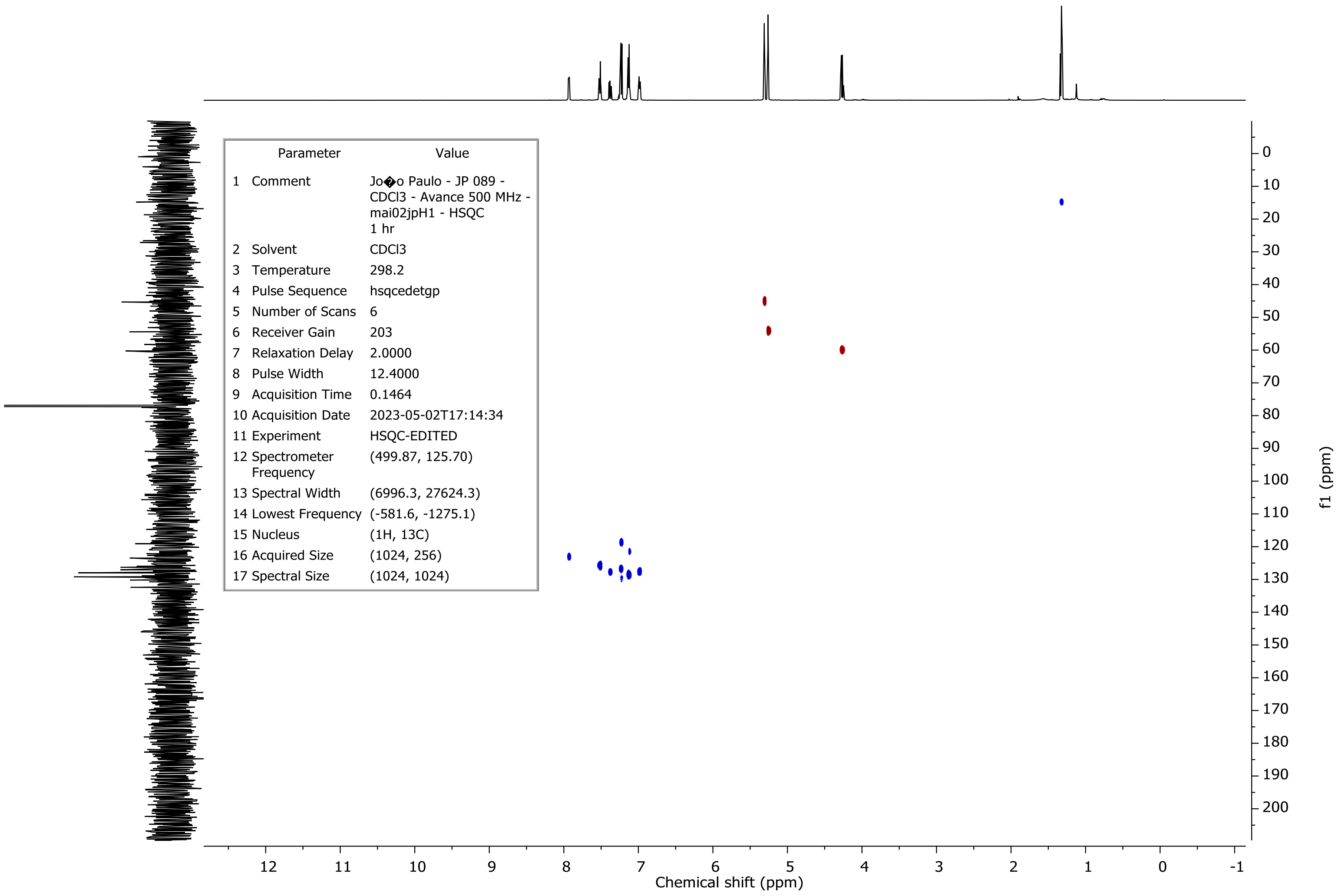

Figure S63 – HSQC of compound **6a**.

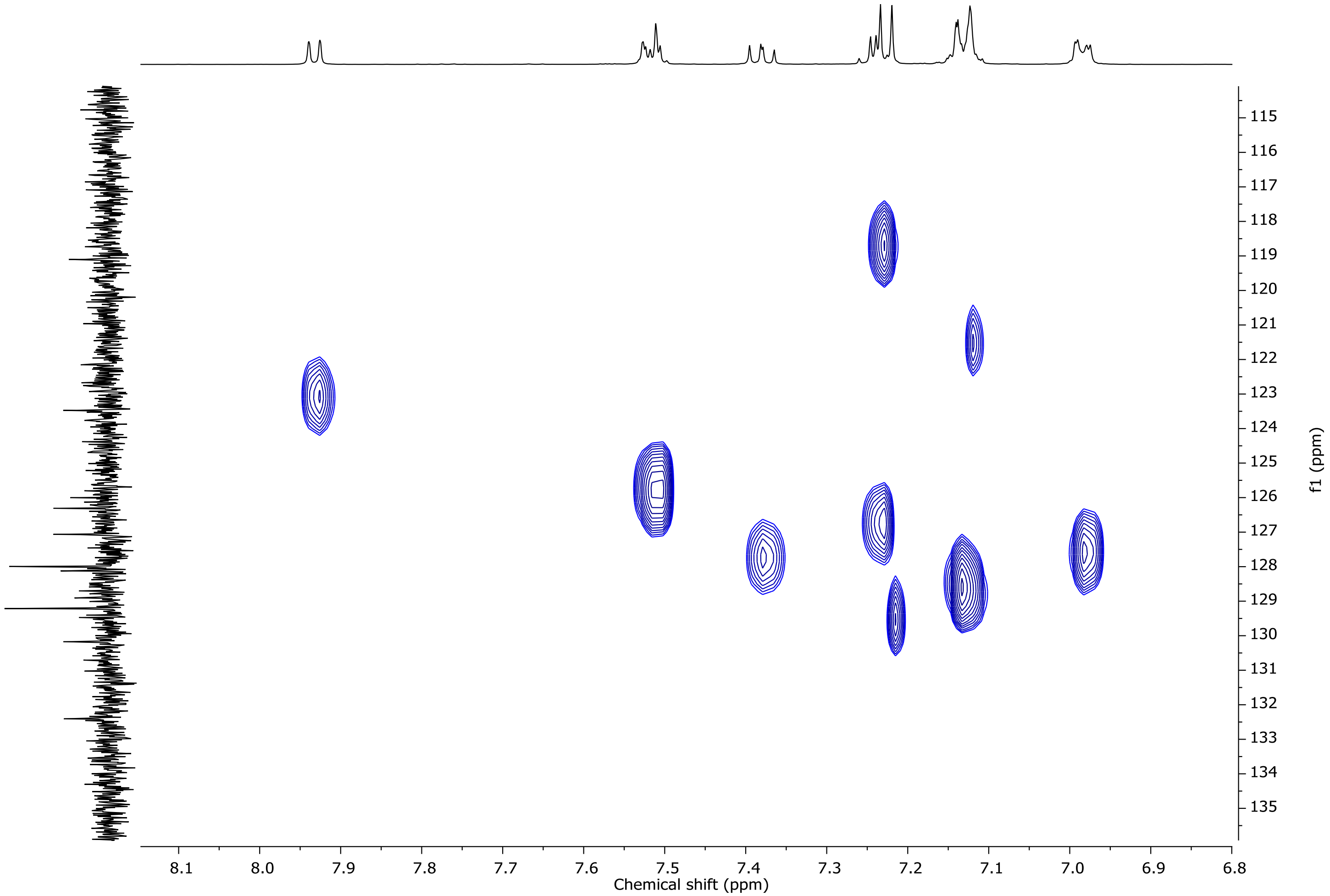

Figure S64 – Zoom of figure **S63** (compound **6a**).

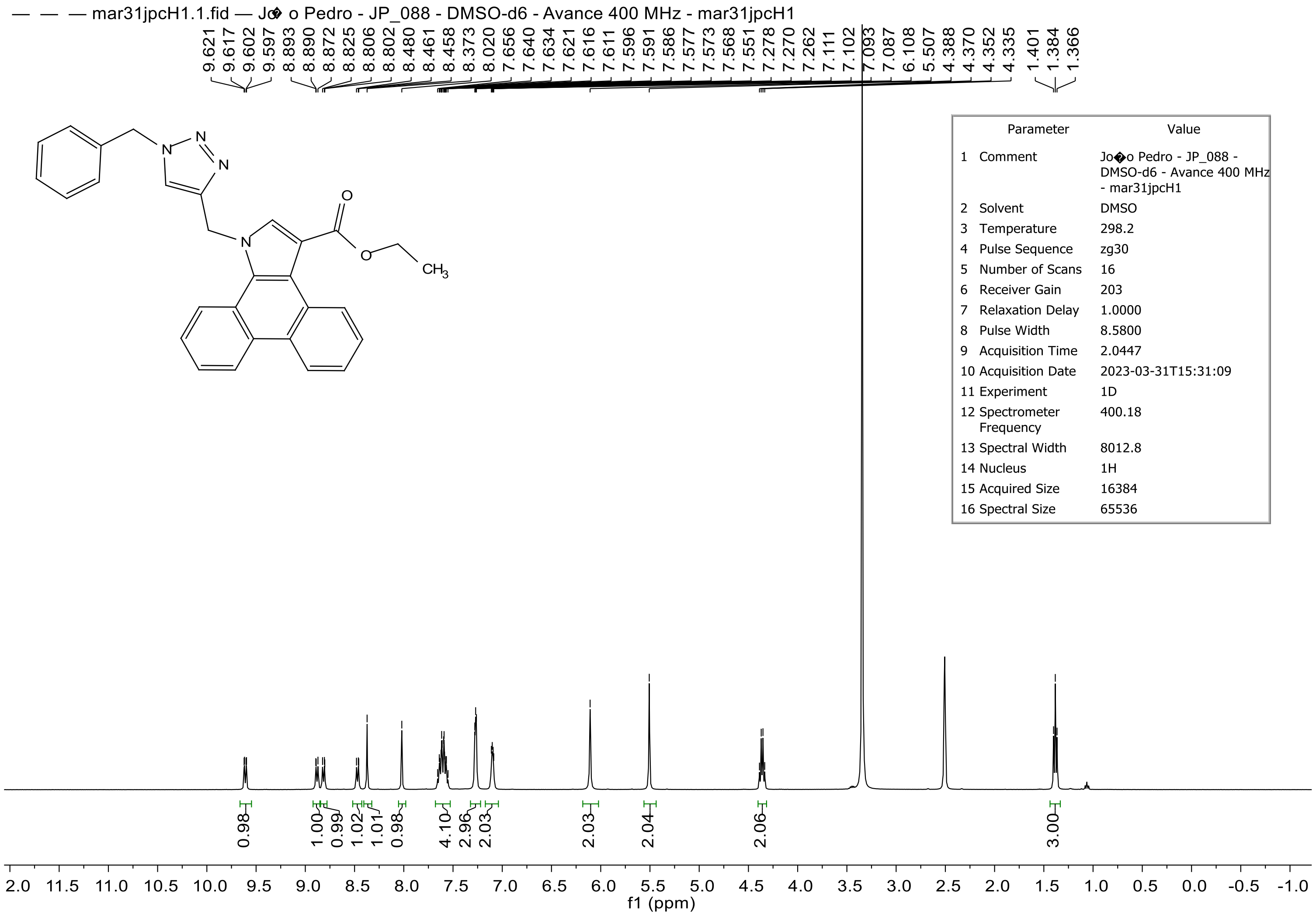

Figure S65 – ^{1}H NMR, 400 Hz, DMSO-d$_6$ (compound **6b**).

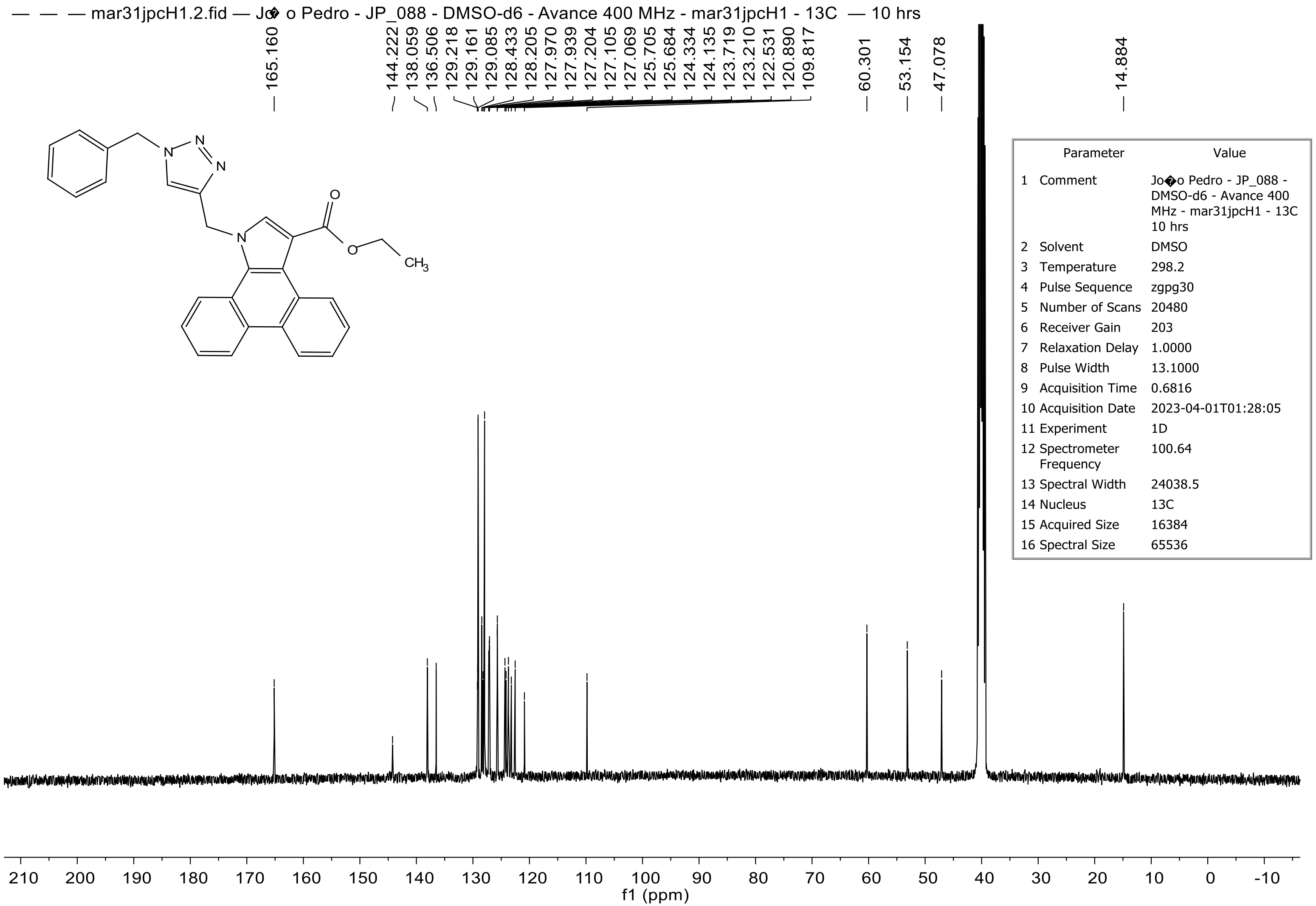

Figure S66 – ^{13}C NMR, 125 Hz, DMSO-d$_6$ (compound **6b**).

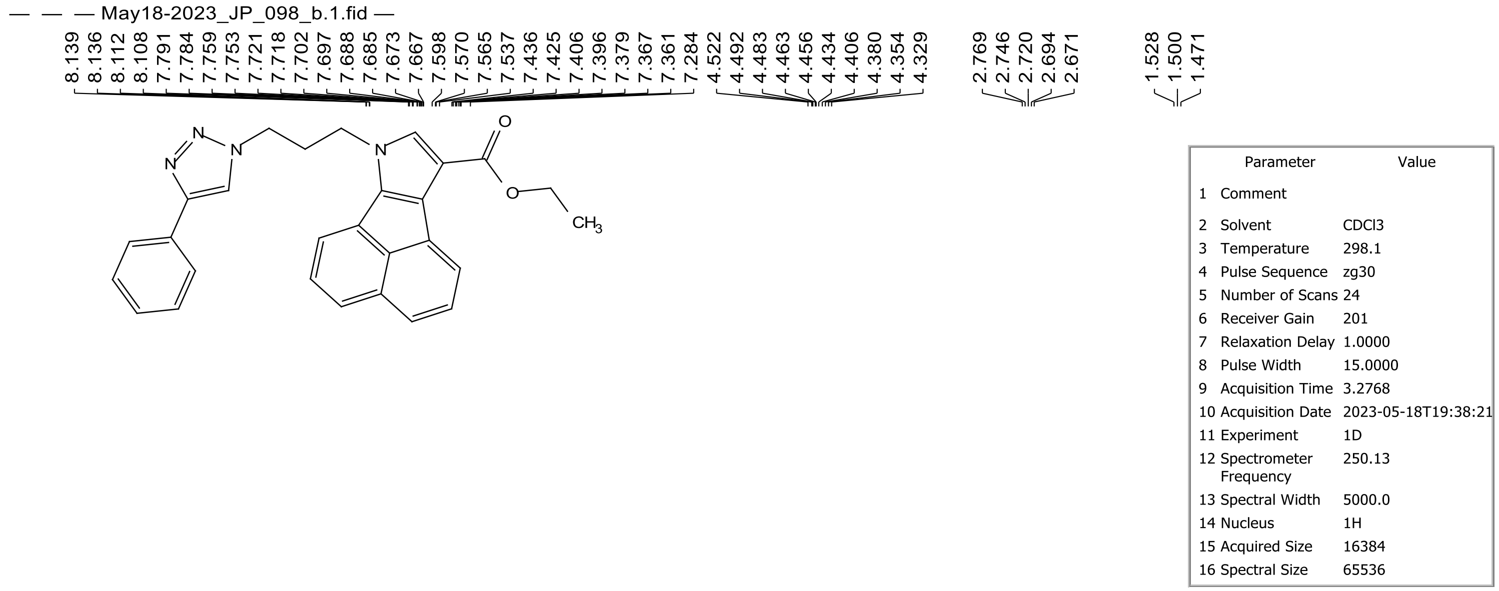

	Parameter	Value
1	Comment	
2	Solvent	CDCl3
3	Temperature	298.1
4	Pulse Sequence	zg30
5	Number of Scans	24
6	Receiver Gain	201
7	Relaxation Delay	1.0000
8	Pulse Width	15.0000
9	Acquisition Time	3.2768
10	Acquisition Date	2023-05-18T19:38:21
11	Experiment	1D
12	Spectrometer Frequency	250.13
13	Spectral Width	5000.0
14	Nucleus	1H
15	Acquired Size	16384
16	Spectral Size	65536

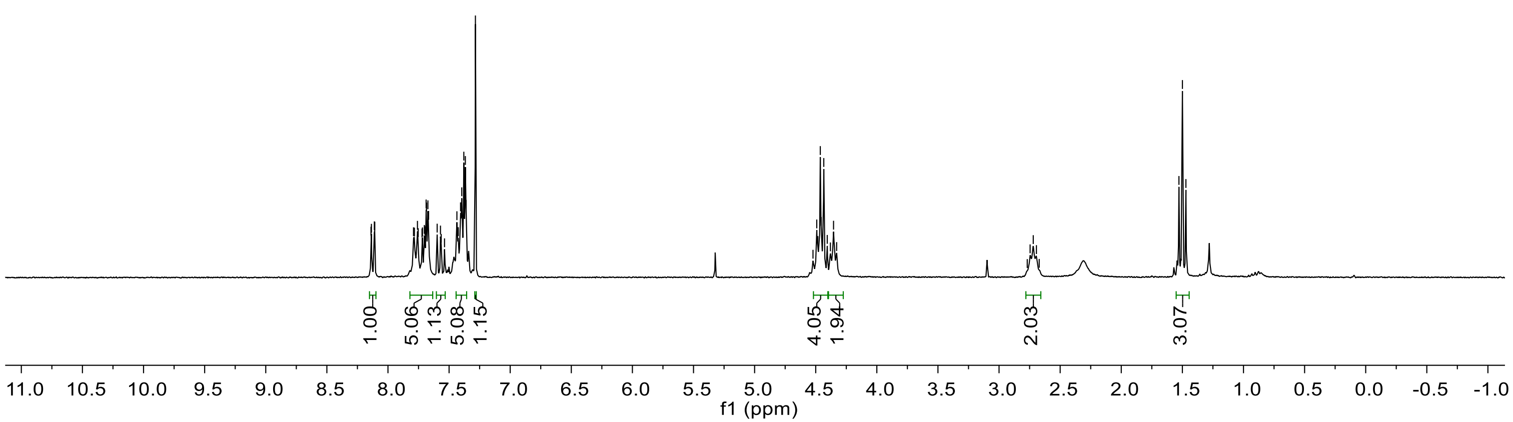

Figure S67 – ^{1}H NMR, 250 Hz, $CDCl_3$ (compound **7a**).

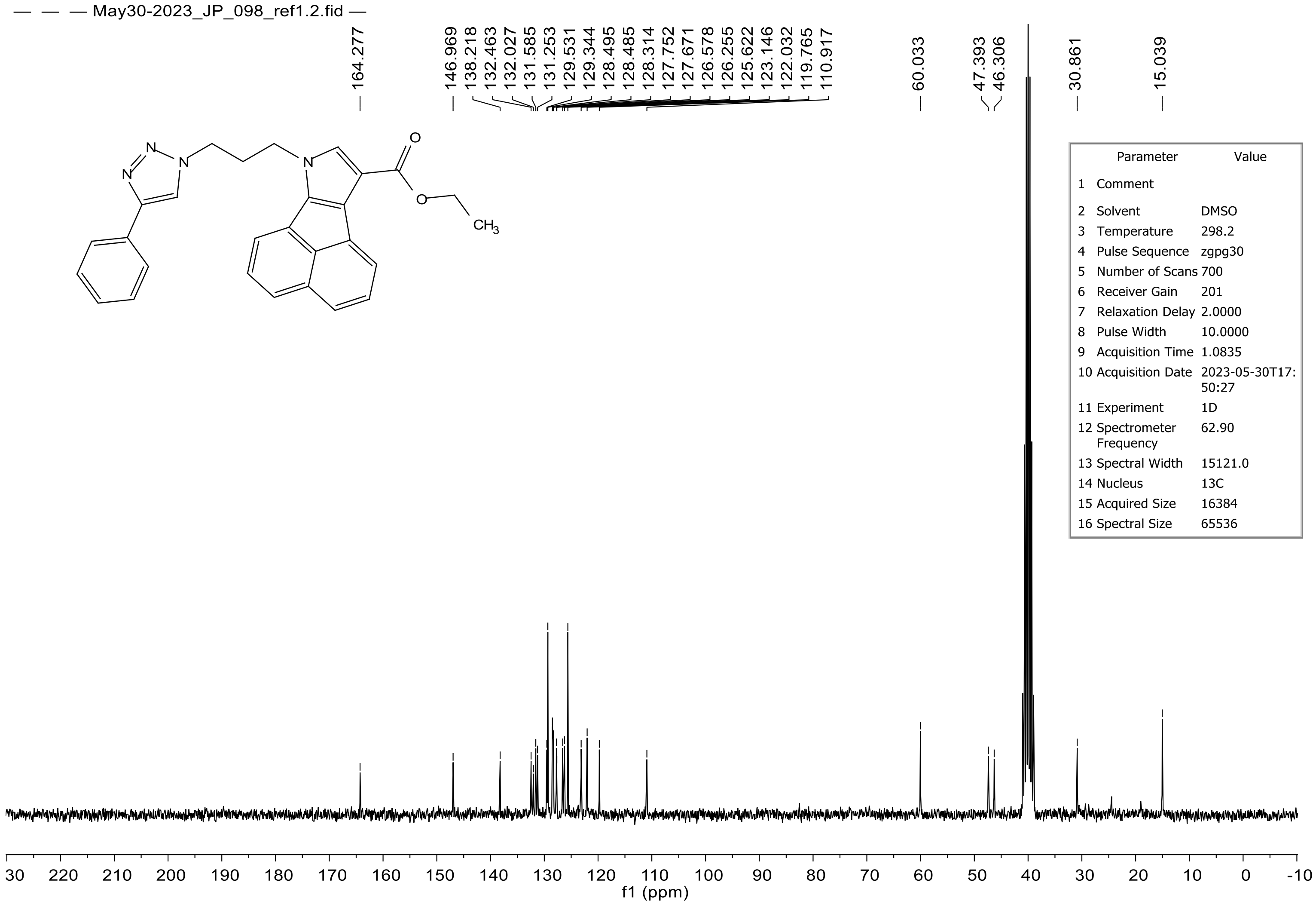

	Parameter	Value
1	Comment	
2	Solvent	DMSO
3	Temperature	298.2
4	Pulse Sequence	zgpg30
5	Number of Scans	700
6	Receiver Gain	201
7	Relaxation Delay	2.0000
8	Pulse Width	10.0000
9	Acquisition Time	1.0835
10	Acquisition Date	2023-05-30T17:50:27
11	Experiment	1D
12	Spectrometer Frequency	62.90
13	Spectral Width	15121.0
14	Nucleus	13C
15	Acquired Size	16384
16	Spectral Size	65536

Figure S68 – ^{13}C NMR, 63 Hz, DMSO-d_6 (compound **7a**).

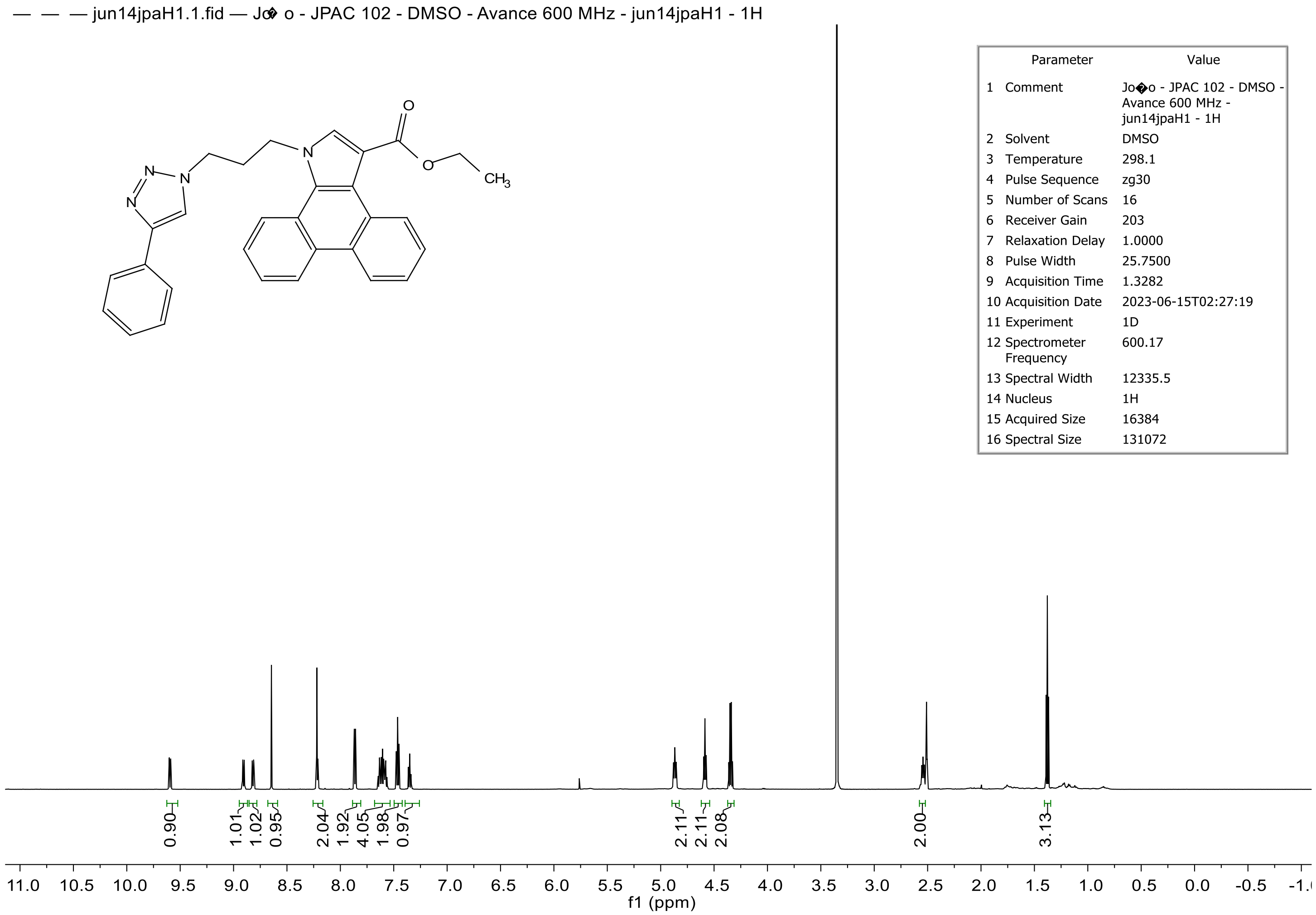

Figure S69 – ^{1}H NMR, 600 Hz, $CDCl_3$ (compound **7b**).

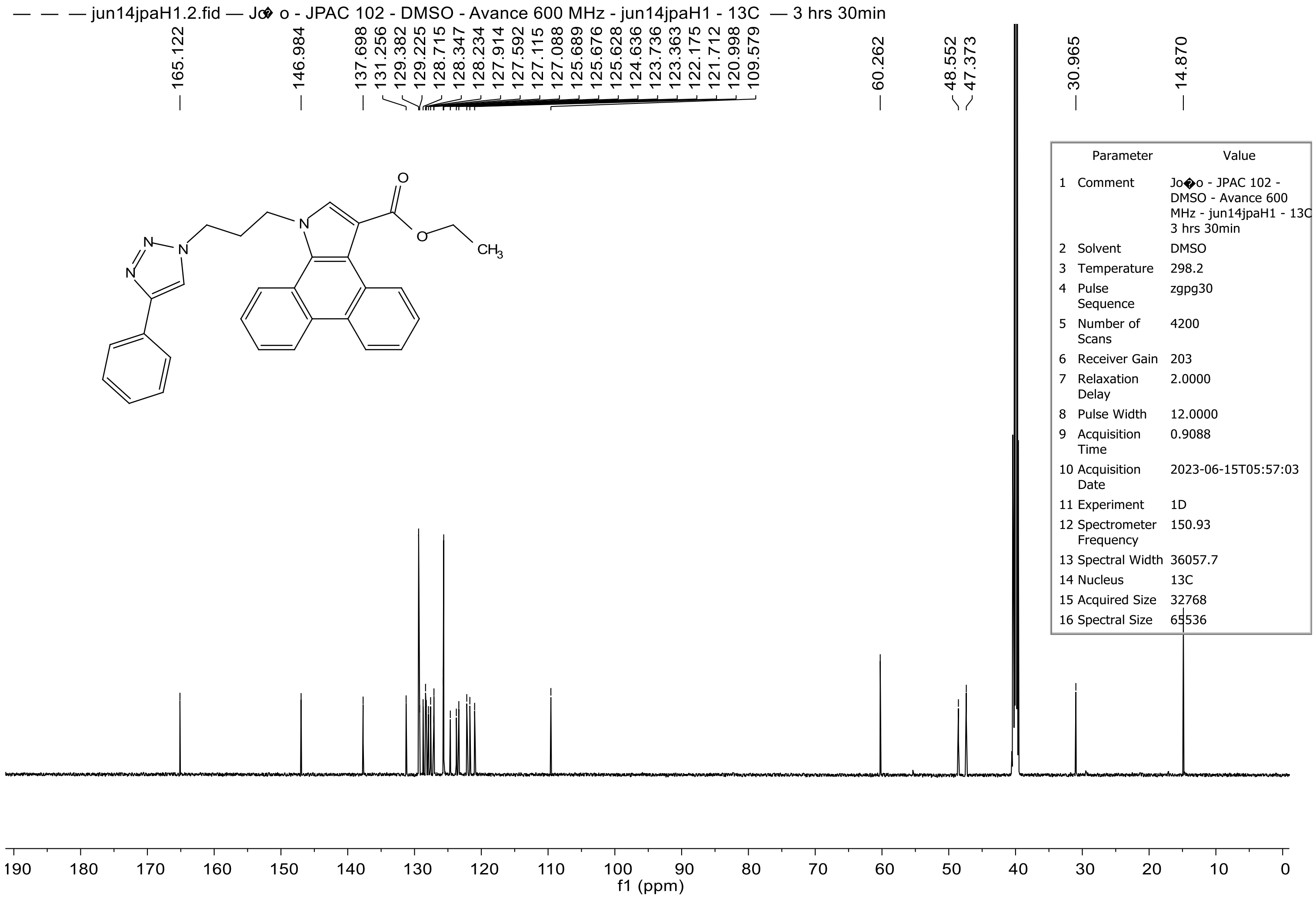

Figure S70 – ^{13}C NMR, 175 Hz, $CDCl_3$ (compound **7b**).

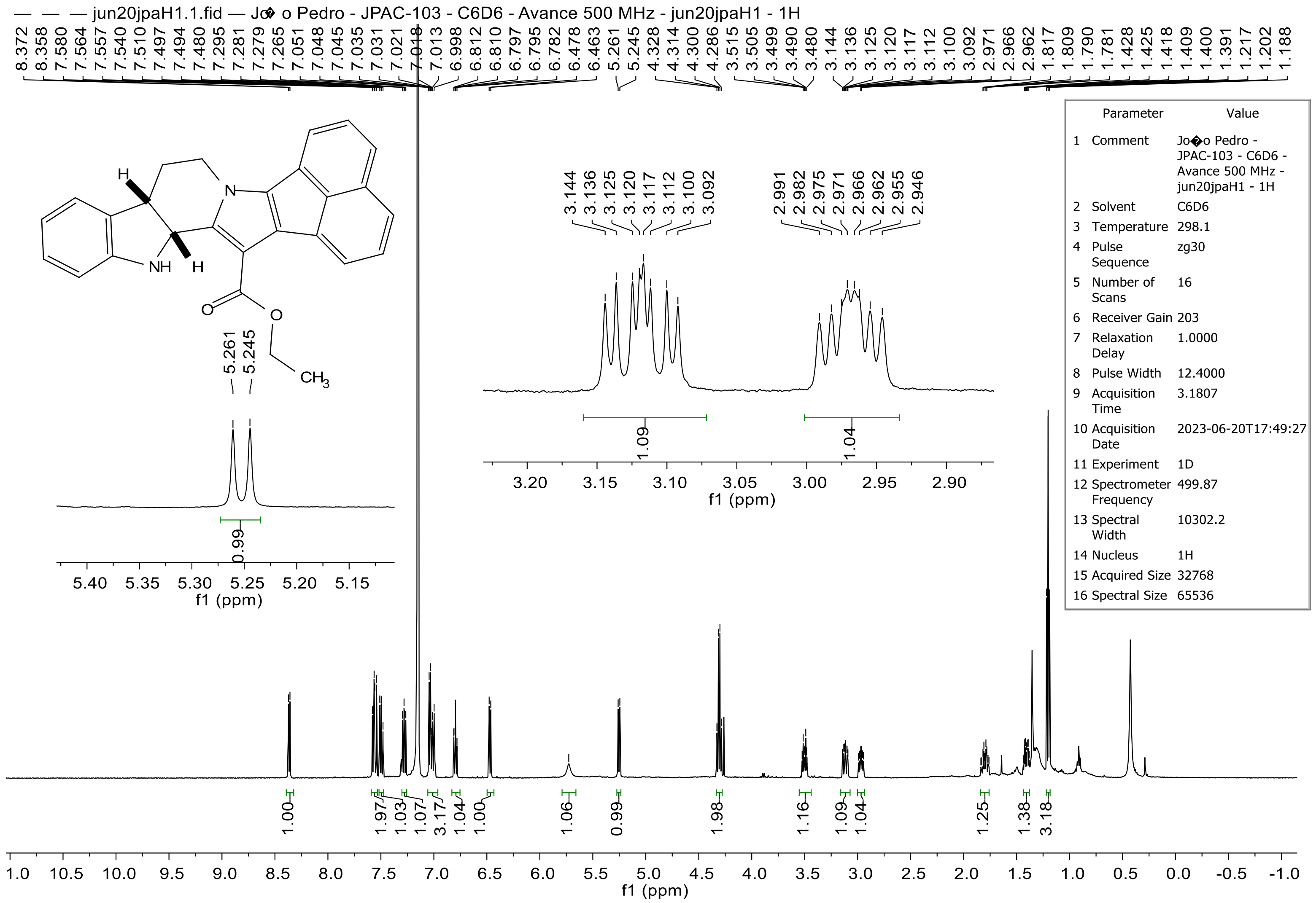

Figure S71 – ^{1}H NMR, 500 Hz, C_6D_6 (compound **8a**).

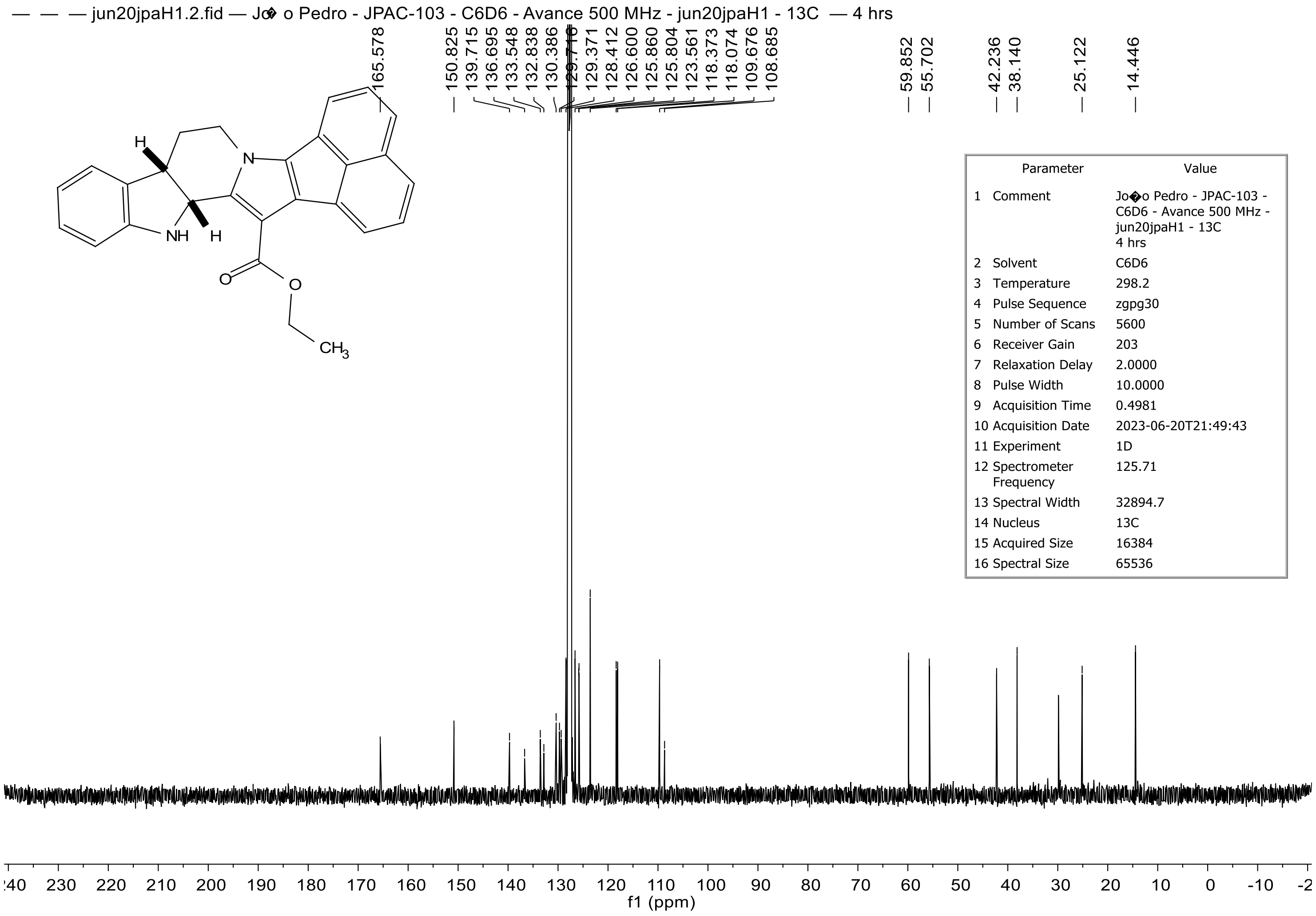

Figure S72 – ^{13}C NMR, 150 Hz, C_6D_6 (compound **8a**).

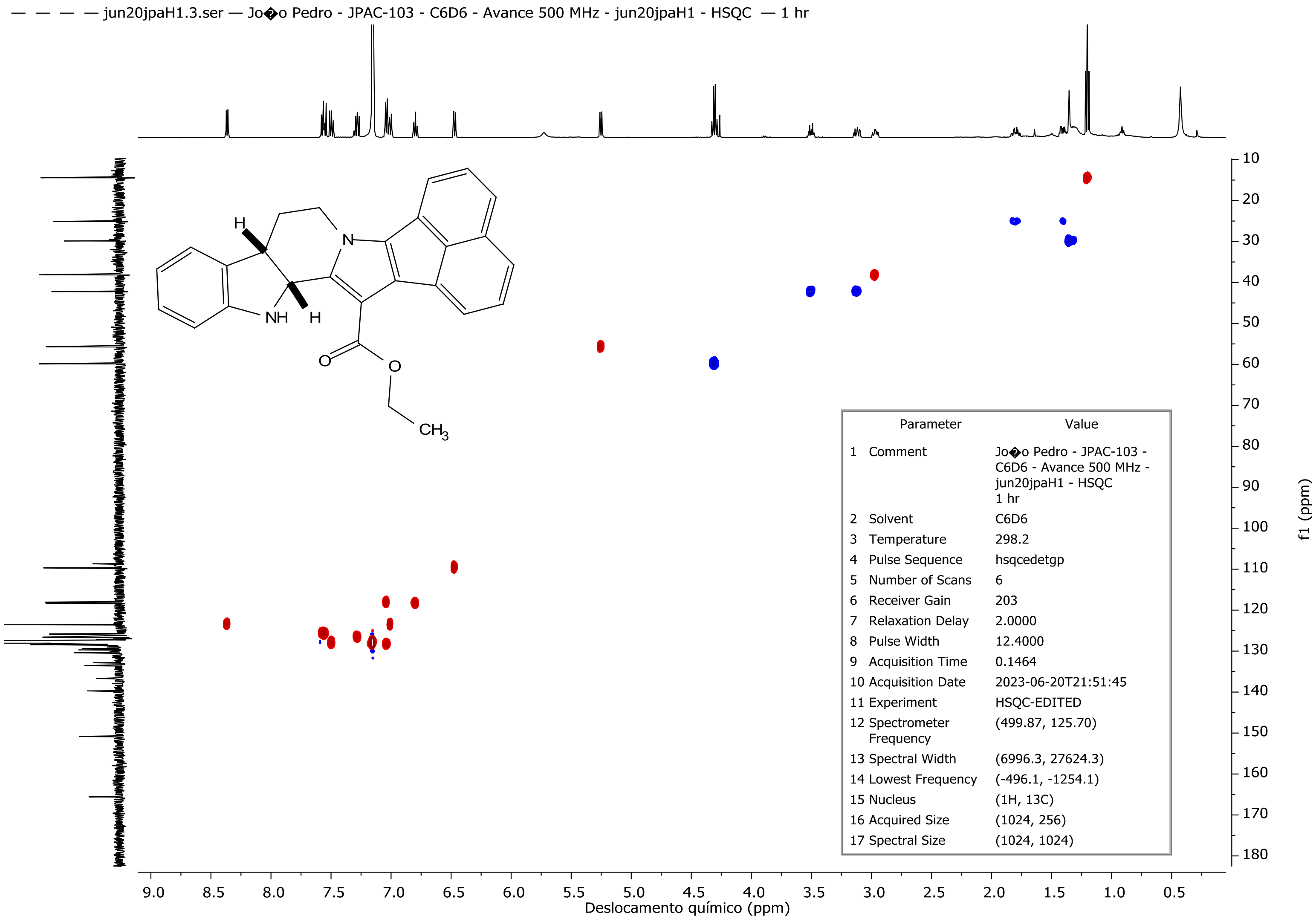

Figure S73 – HSQC of compound **8a**.

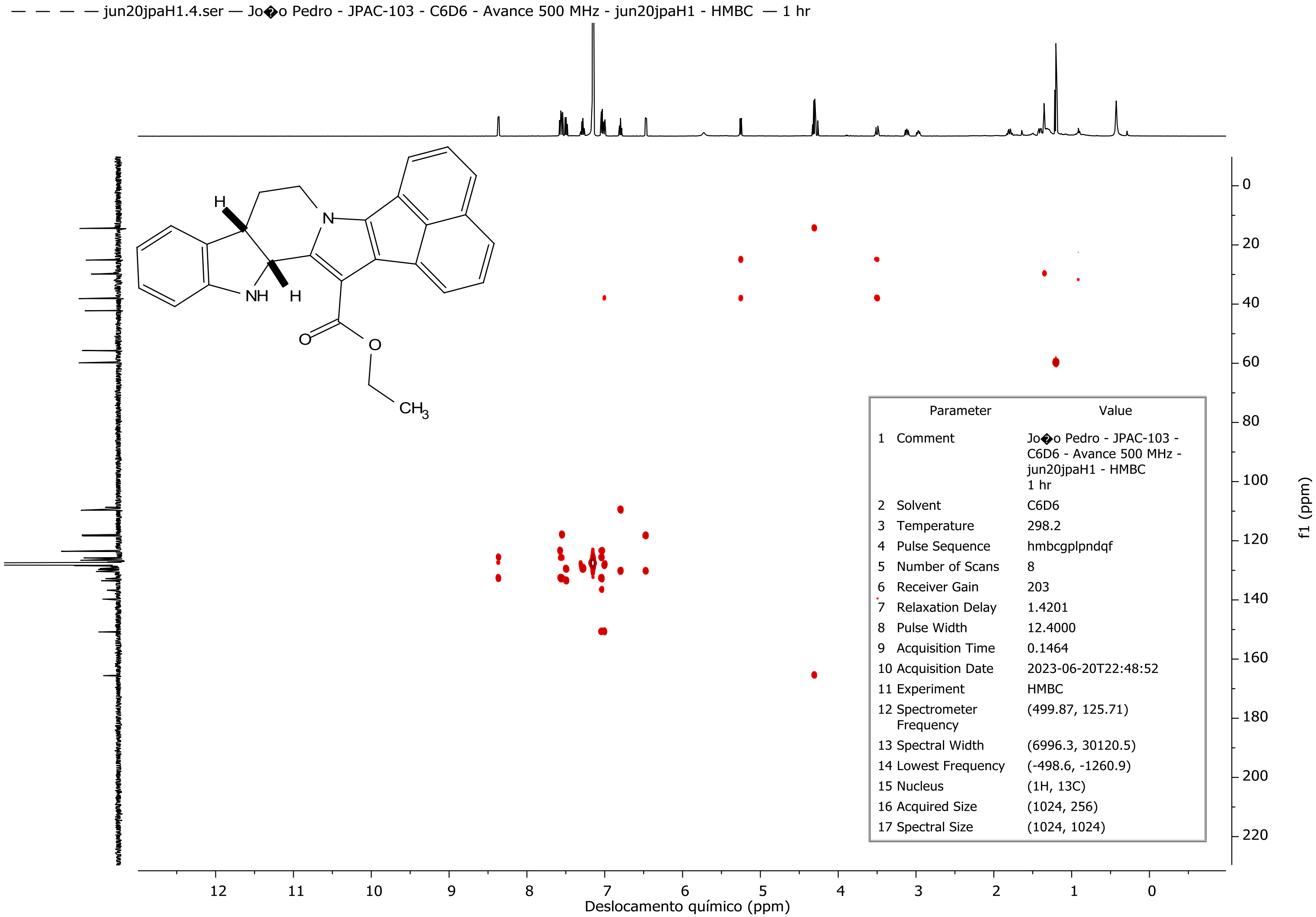

Figure S74 – HMBC of compound **8a**.

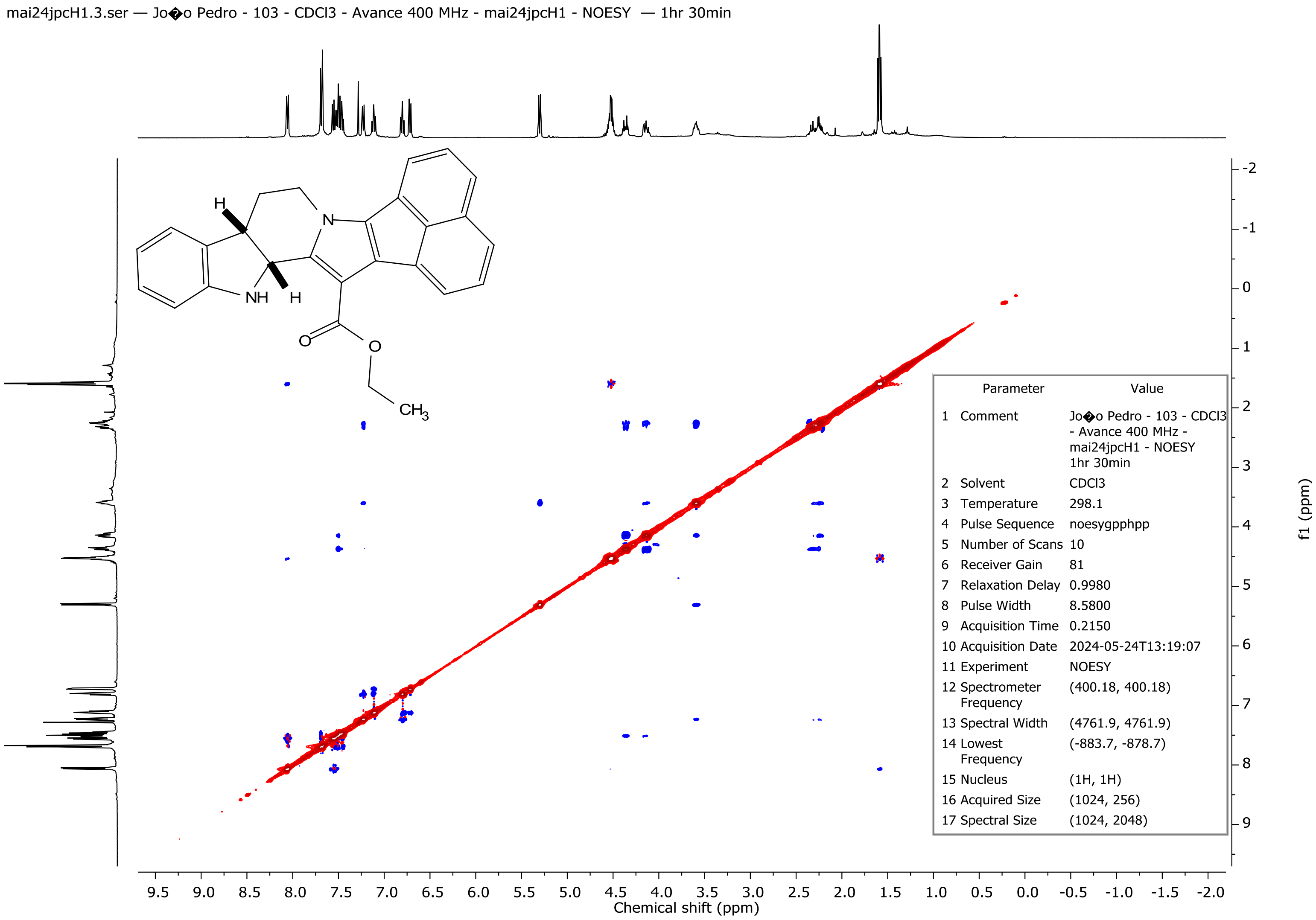

Figure S75 – NOESY of compound **8a**.

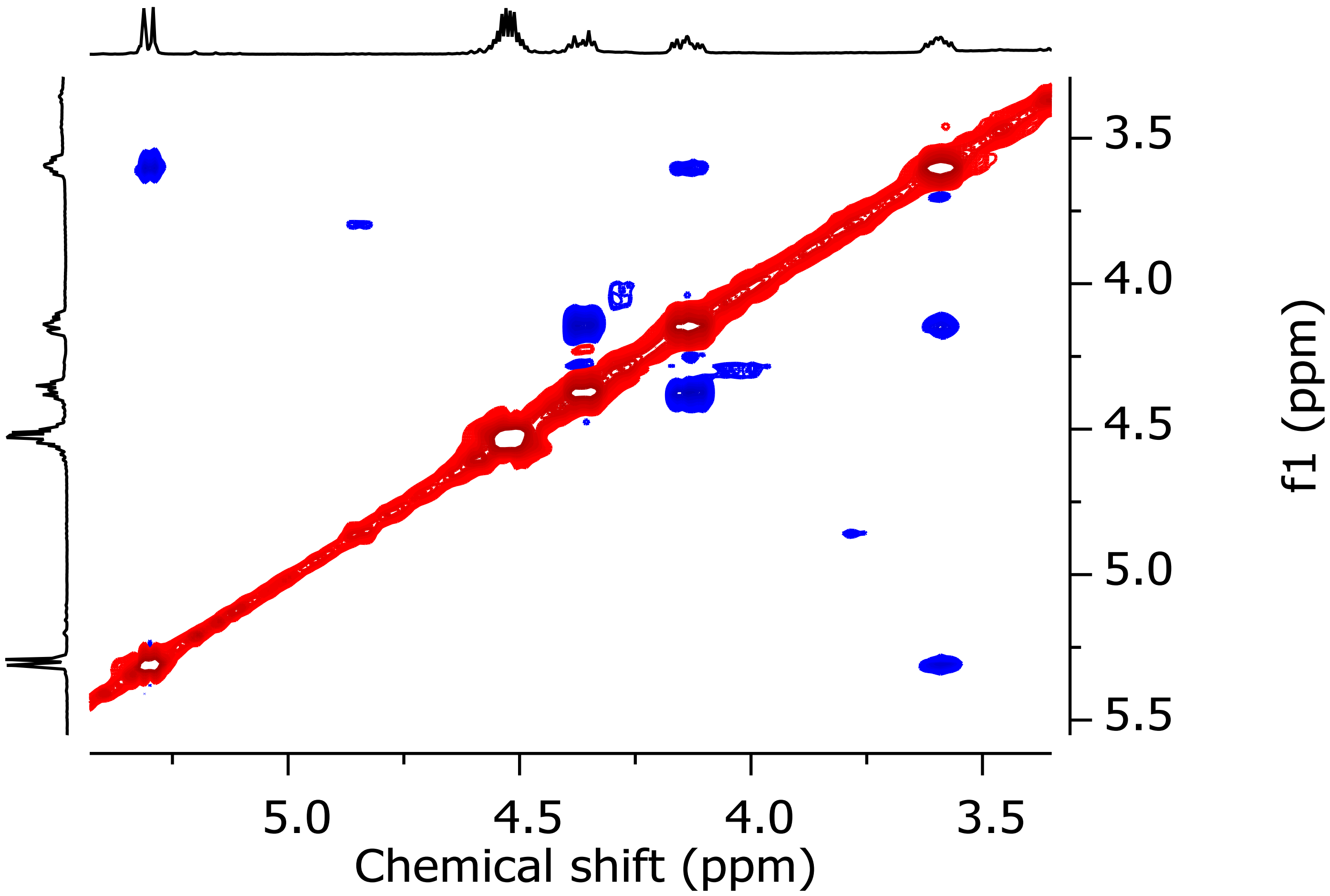

Figure S76 – Zoom of figure **S75** (coumpound **8a**).

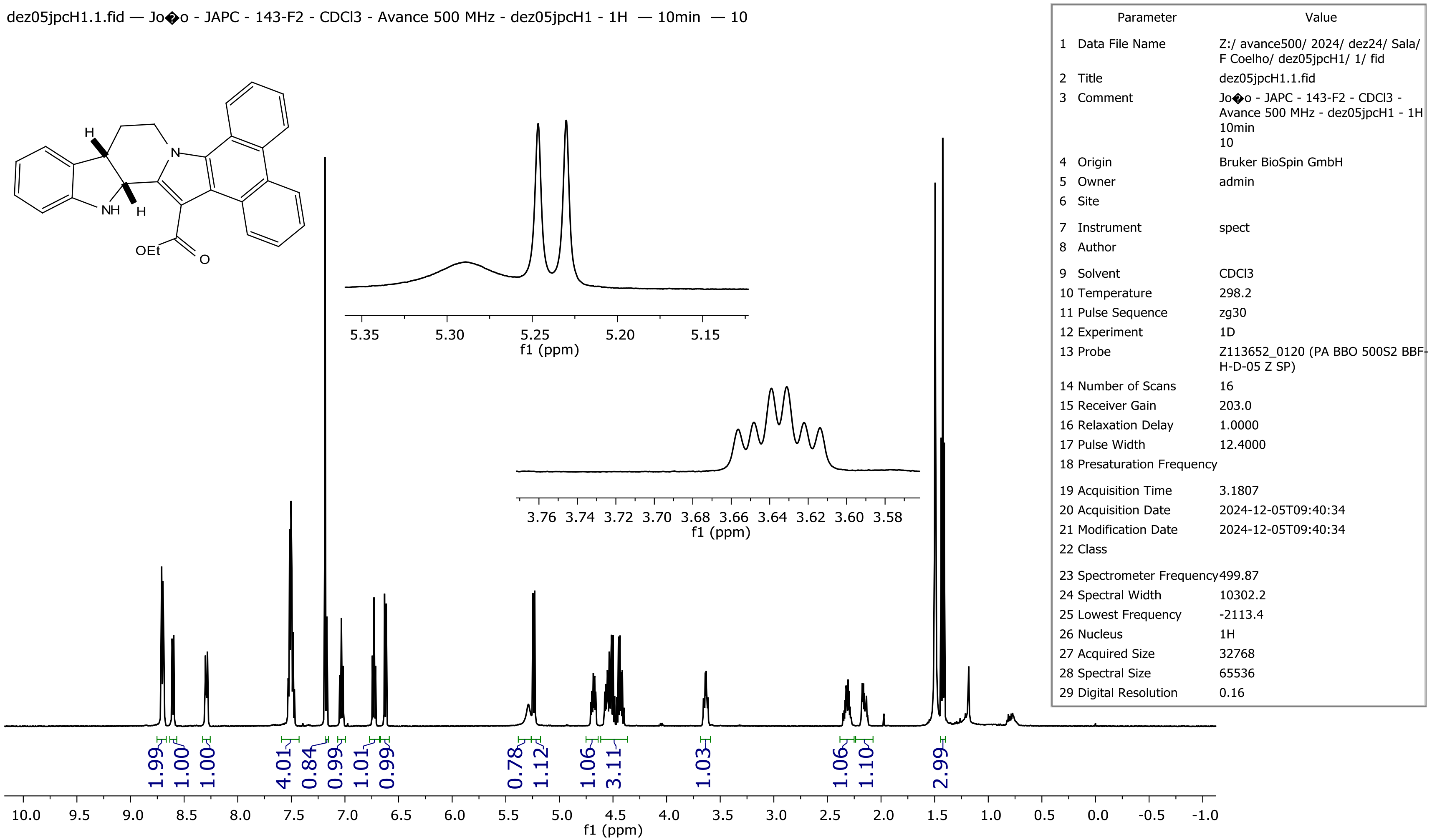

	Parameter	Value
1	Data File Name	Z:/ avance500/ 2024/ dez24/ Sala/ F Coelho/ dez05jpcH1/ 1/ fid
2	Title	dez05jpcH1.1.fid
3	Comment	Jo�o - JAPC - 143-F2 - CDCl3 - Avance 500 MHz - dez05jpcH1 - 1H 10min 10
4	Origin	Bruker BioSpin GmbH
5	Owner	admin
6	Site	
7	Instrument	spect
8	Author	
9	Solvent	CDCl3
10	Temperature	298.2
11	Pulse Sequence	zg30
12	Experiment	1D
13	Probe	Z113652_0120 (PA BBO 500S2 BBF-H-D-05 Z SP)
14	Number of Scans	16
15	Receiver Gain	203.0
16	Relaxation Delay	1.0000
17	Pulse Width	12.4000
18	Presaturation Frequency	
19	Acquisition Time	3.1807
20	Acquisition Date	2024-12-05T09:40:34
21	Modification Date	2024-12-05T09:40:34
22	Class	
23	Spectrometer Frequency	499.87
24	Spectral Width	10302.2
25	Lowest Frequency	-2113.4
26	Nucleus	1H
27	Acquired Size	32768
28	Spectral Size	65536
29	Digital Resolution	0.16

Figure S77 – ^{1}H NMR, 500 Hz, $CDCl_3$ (compound **8b**).

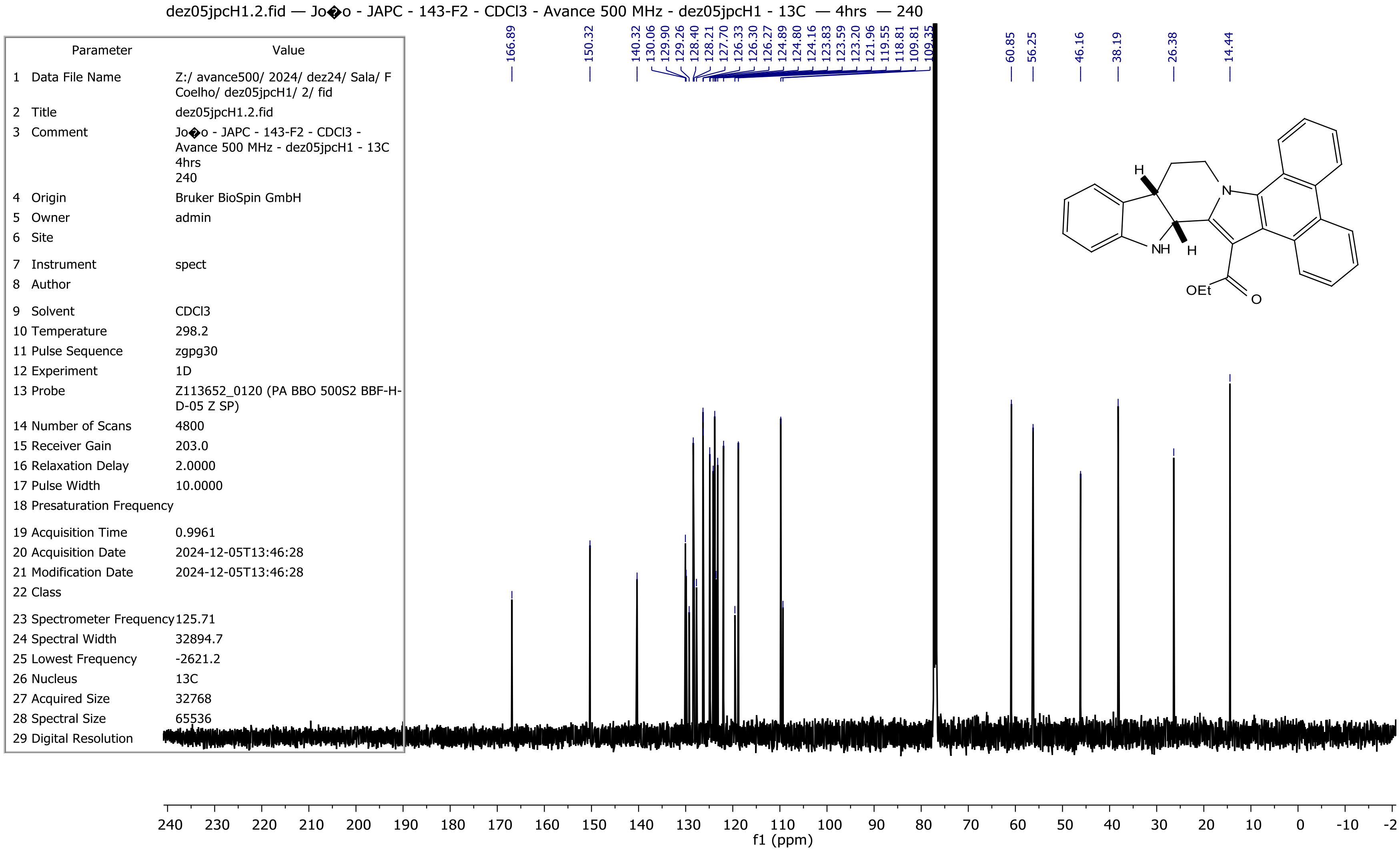

	Parameter	Value
1	Data File Name	Z:/ avance500/ 2024/ dez24/ Sala/ F Coelho/ dez05jpcH1/ 2/ fid
2	Title	dez05jpcH1.2.fid
3	Comment	Jo�o - JAPC - 143-F2 - CDCl3 - Avance 500 MHz - dez05jpcH1 - 13C 4hrs 240
4	Origin	Bruker BioSpin GmbH
5	Owner	admin
6	Site	
7	Instrument	spect
8	Author	
9	Solvent	CDCl3
10	Temperature	298.2
11	Pulse Sequence	zgpg30
12	Experiment	1D
13	Probe	Z113652_0120 (PA BBO 500S2 BBF-H-D-05 Z SP)
14	Number of Scans	4800
15	Receiver Gain	203.0
16	Relaxation Delay	2.0000
17	Pulse Width	10.0000
18	Presaturation Frequency	
19	Acquisition Time	0.9961
20	Acquisition Date	2024-12-05T13:46:28
21	Modification Date	2024-12-05T13:46:28
22	Class	
23	Spectrometer Frequency	125.71
24	Spectral Width	32894.7
25	Lowest Frequency	-2621.2
26	Nucleus	13C
27	Acquired Size	32768
28	Spectral Size	65536
29	Digital Resolution	

Figure S78 – ^{13}C NMR, 125 Hz, $CDCl_3$ (compound **8b**).

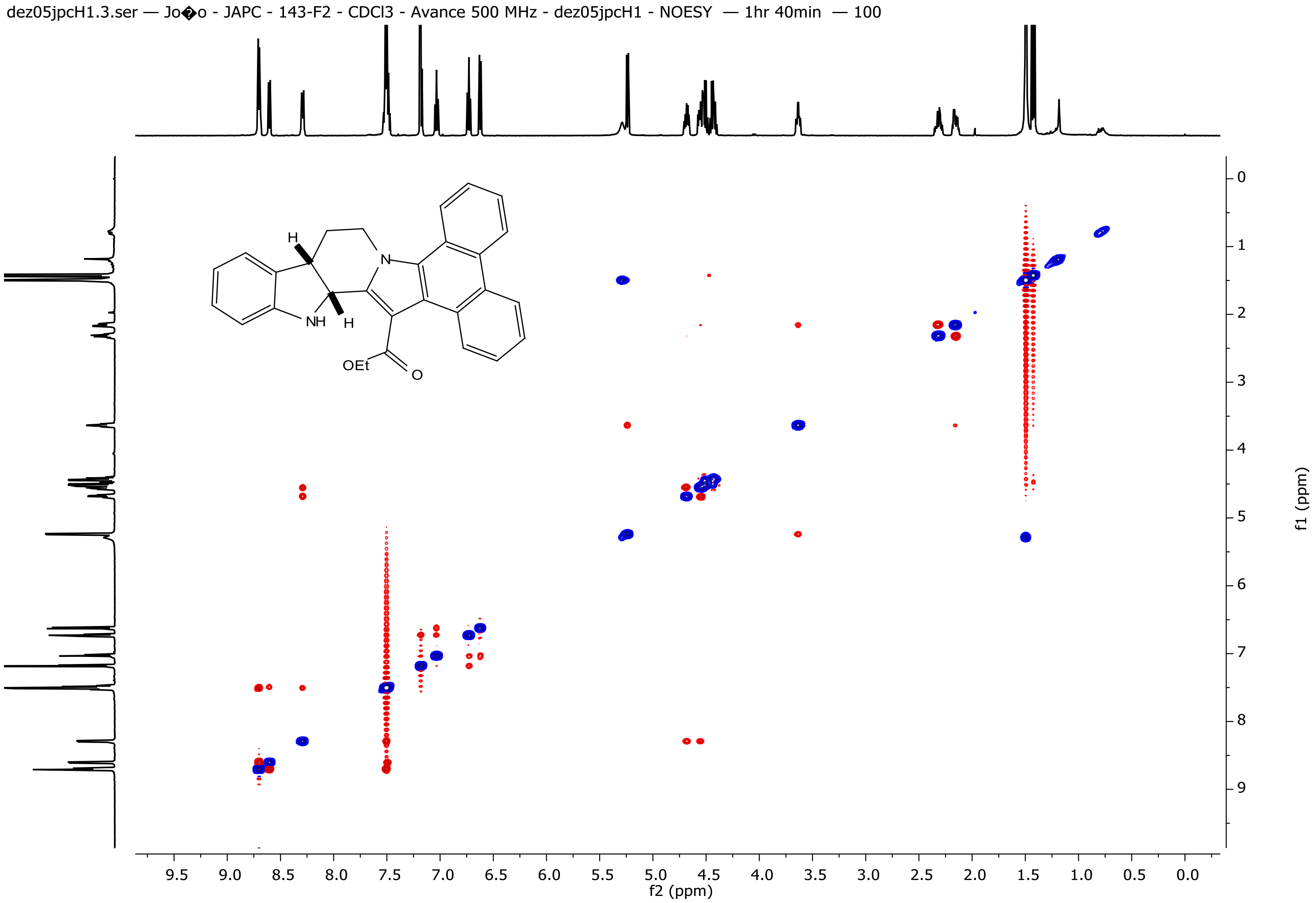

	Parameter	Value
1	Data File Name	Z:/ avance500/ 2024/ dez24/ Sala/ F Coelho/ dez05jpcH1/ 3/ ser
2	Title	dez05jpcH1.3.ser
3	Comment	Jo�o - JAPC - 143-F2 - CDCl3 - Avance 500 MHz - dez05jpcH1 - NOESY 1hr 40min 100
4	Origin	Bruker BioSpin GmbH
5	Owner	admin
6	Site	
7	Instrument	spect
8	Author	
9	Solvent	CDCl3
10	Temperature	298.2
11	Pulse Sequence	noesygpph
12	Experiment	NOESY
13	Probe	Z113652_0120 (PA BBO 500S2 BBF-H-D-05 Z SP)
14	Number of Scans	8
15	Receiver Gain	144.0
16	Relaxation Delay	1.9570
17	Pulse Width	12.4000
18	Presaturation Frequency	
19	Acquisition Time	0.2007
20	Acquisition Date	2024-12-05T13:48:27
21	Modification Date	2024-12-05T15:29:33
22	Class	
23	Spectrometer Frequency	(499.87, 499.87)
24	Spectral Width	(5102.0, 5102.0)
25	Lowest Frequency	(-170.9, -170.9)
26	Nucleus	(1H, 1H)
27	Acquired Size	(1024, 256)
28	Spectral Size	(1024, 1024)
29	Digital Resolution	(4.98, 4.98)

Figure S79 – NOESY of compound **8b**.

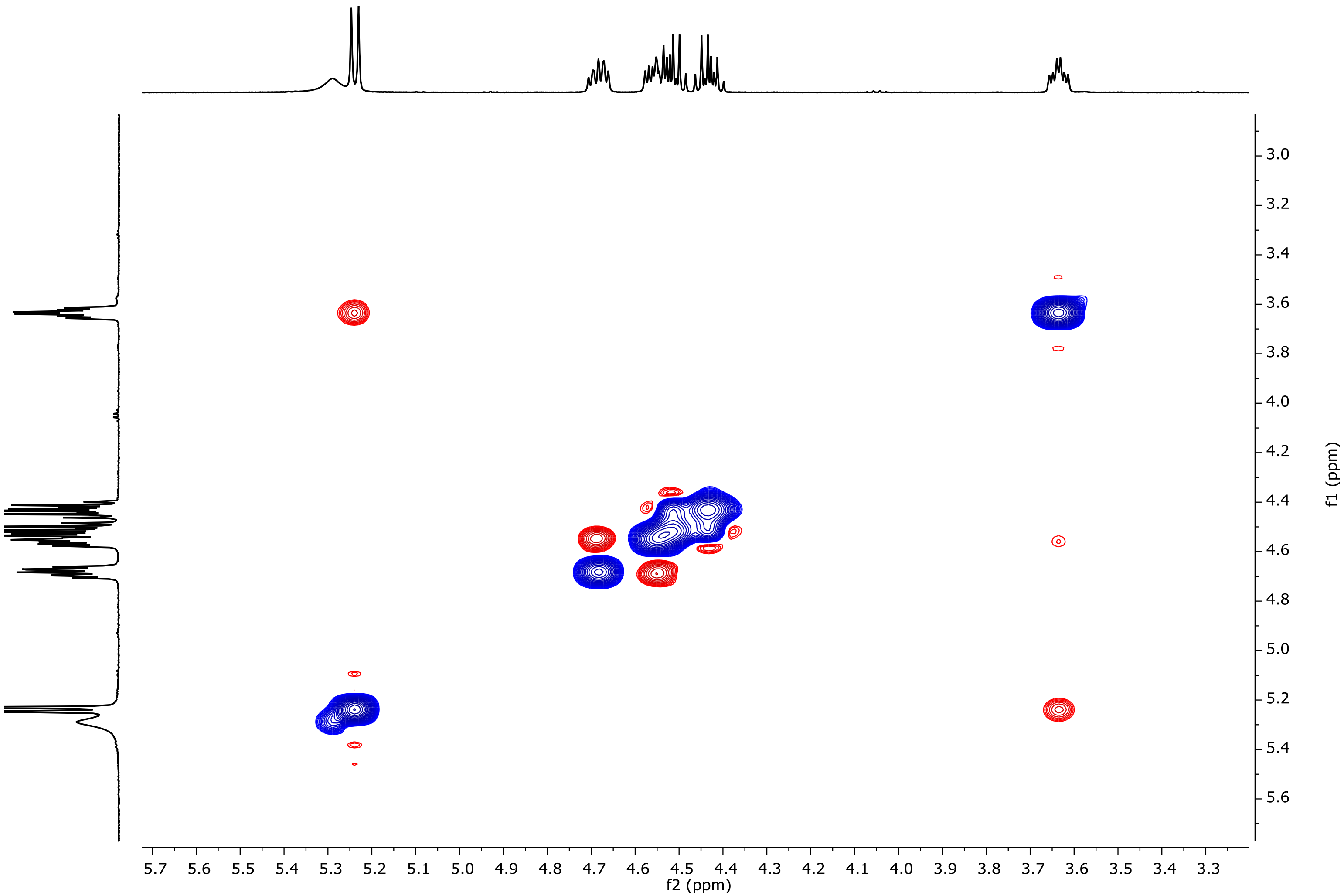

Figure S80 – Expansion of figure **S79** (compound **8b**).

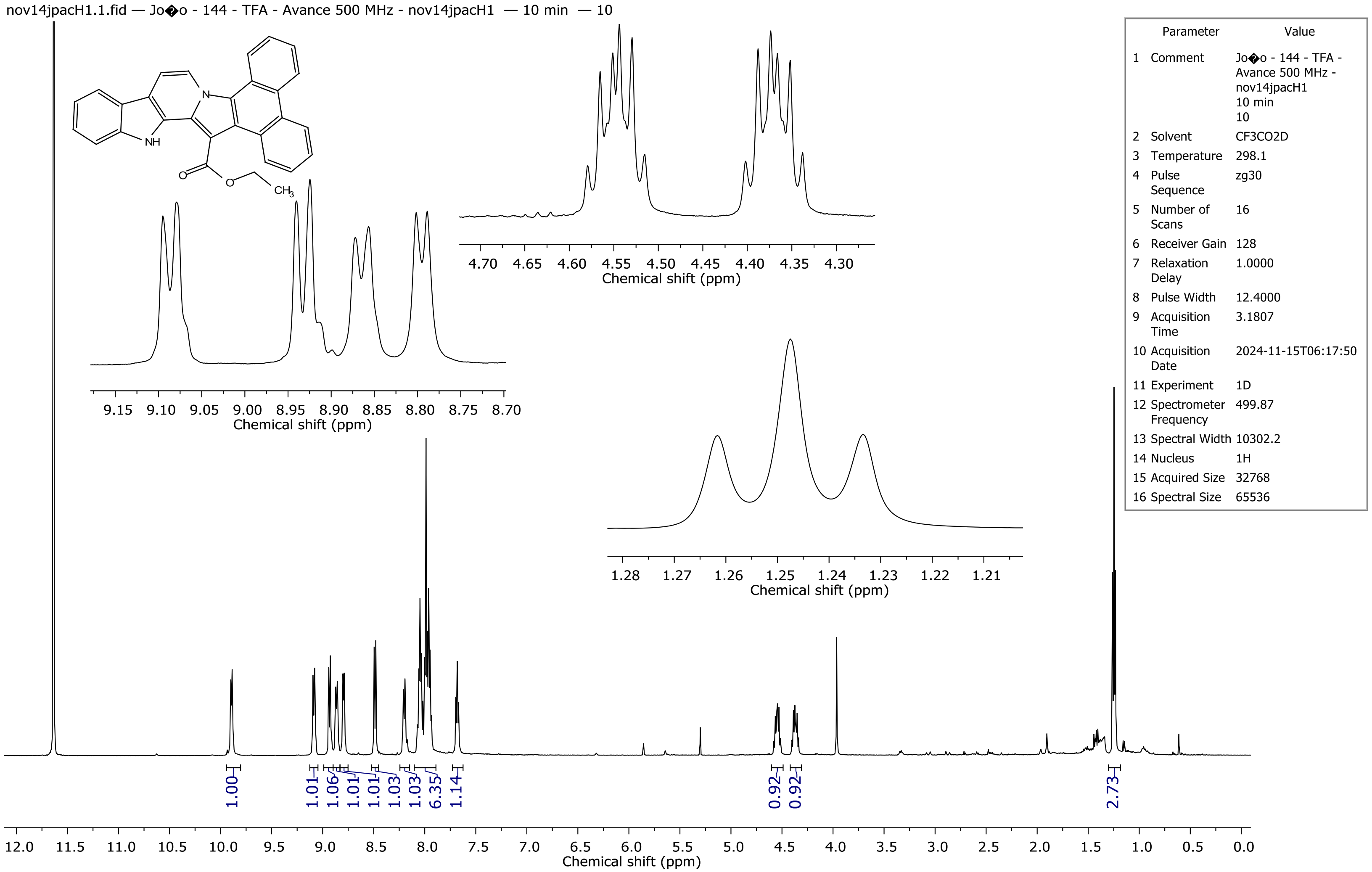

Figure S81 – ^{1}H NMR, 500 Hz, TFA – d (compound **9**).

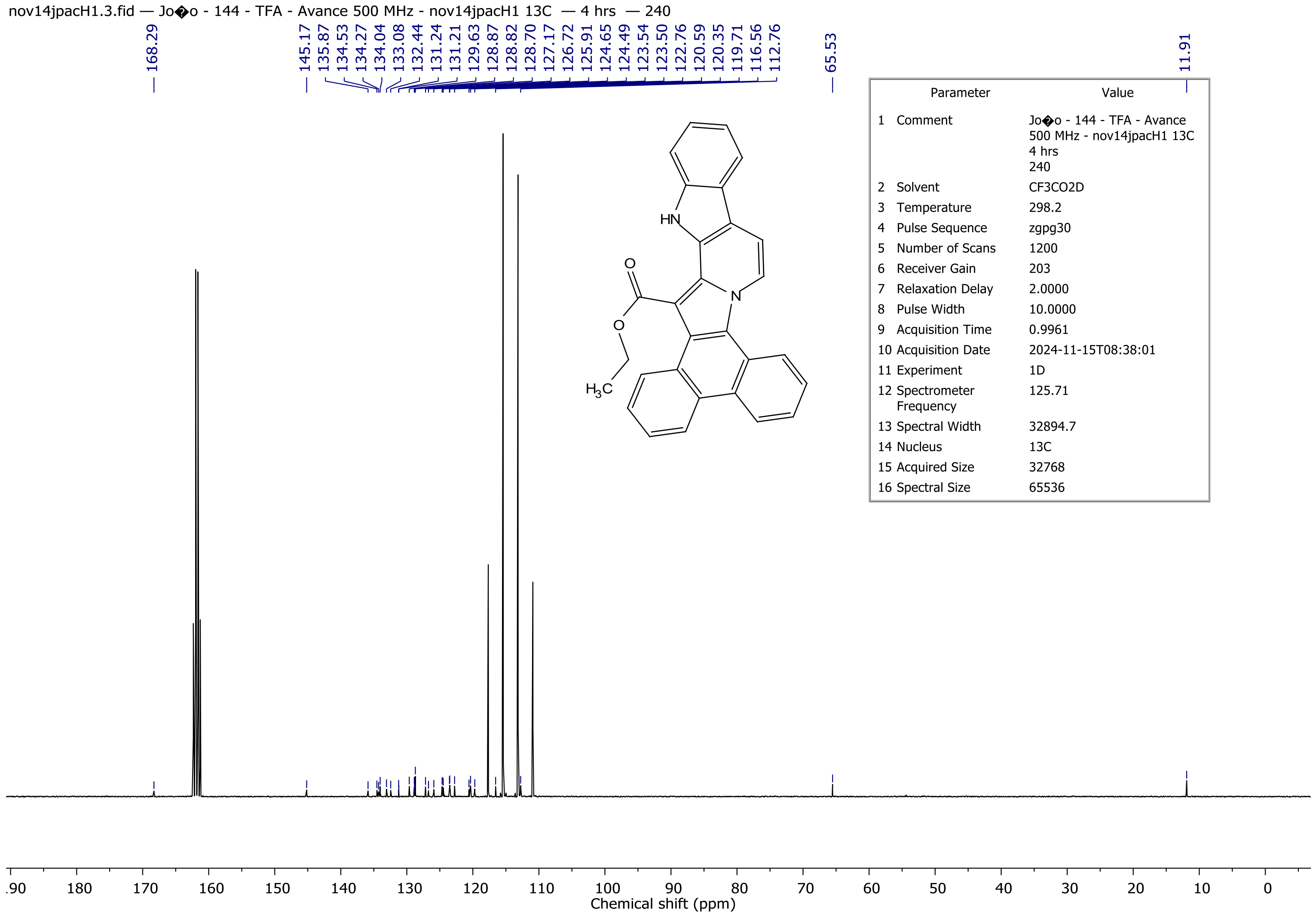

Figure S82 – ^{13}C NMR, 125 Hz, TFA – d (compound **9**).

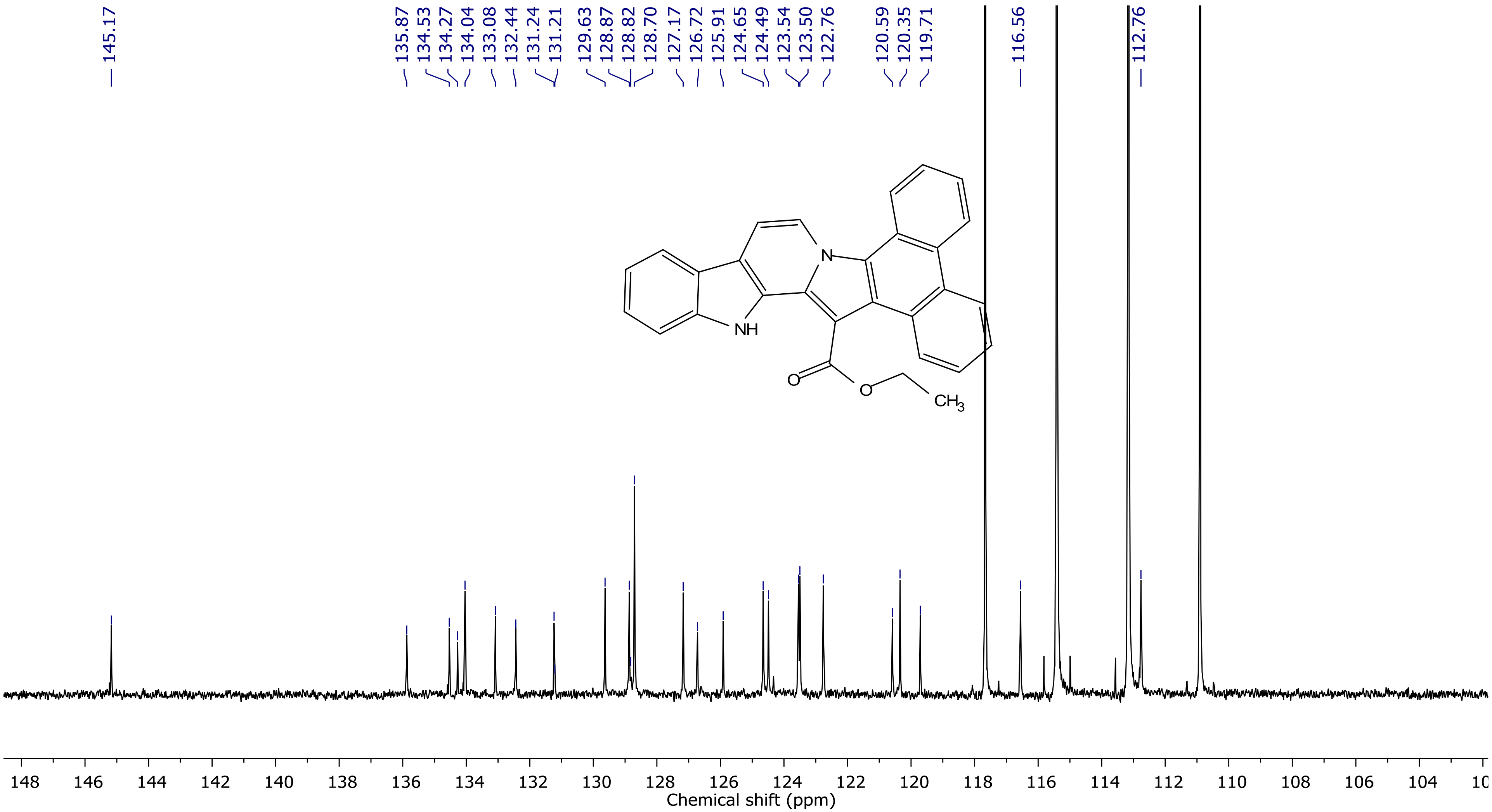

Figure S83 – Zoom of figure **S82** (compound **9**).

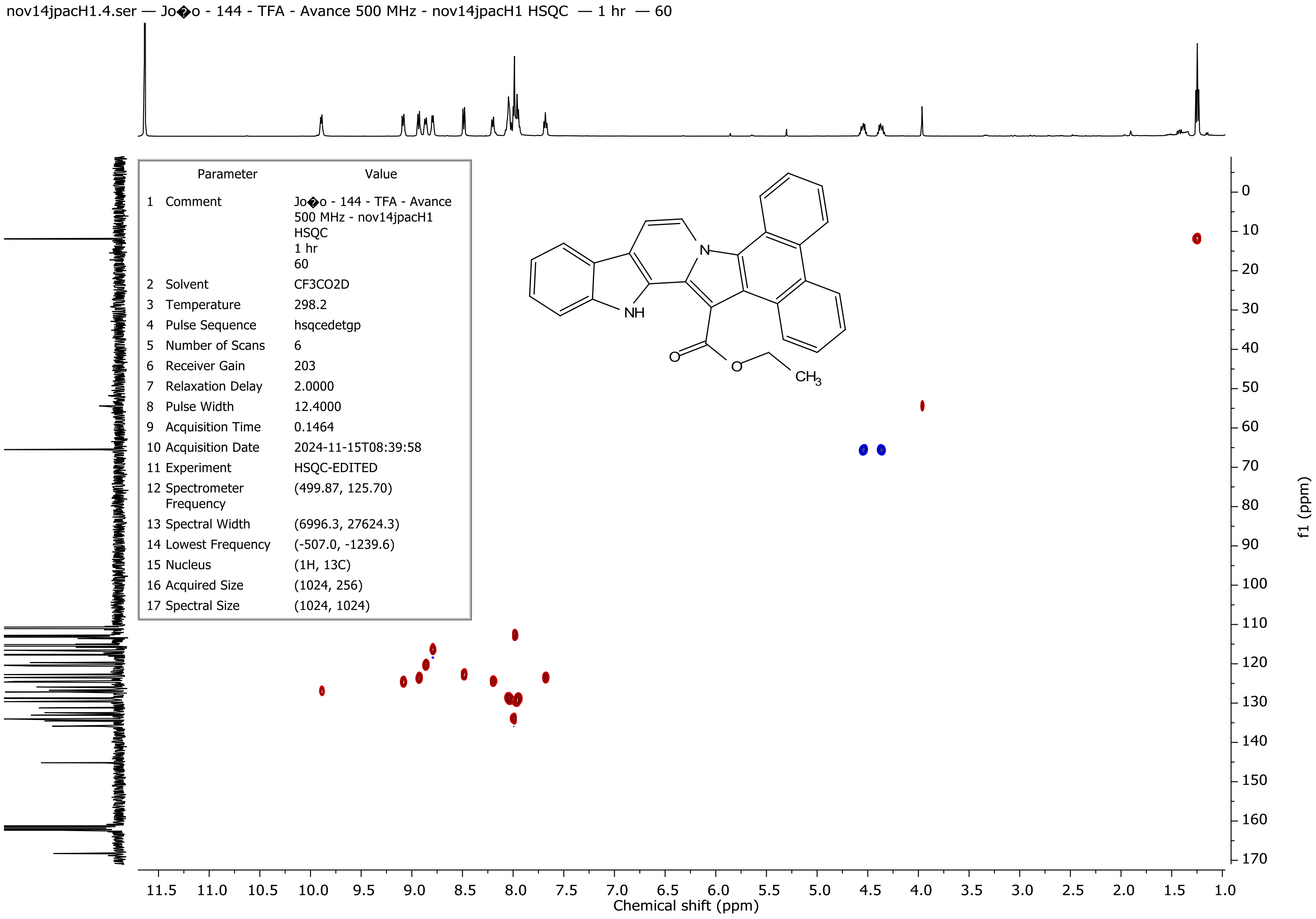

Figure S84 – HSQC of compound **9**.

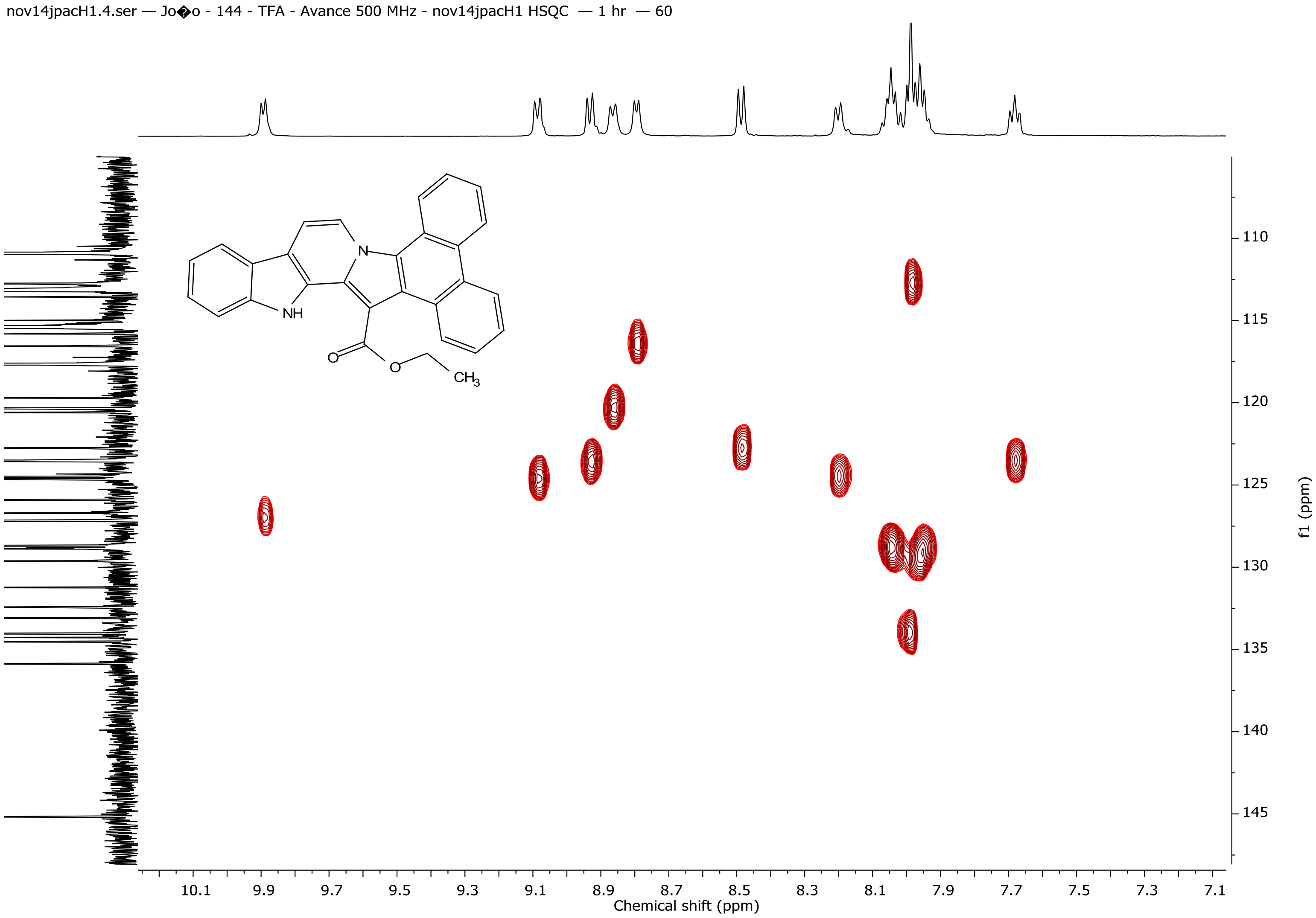

Figure S85 – Zoom of figure **S84** (compound **9**).

Copies of dose-response curves for synthesized compounds

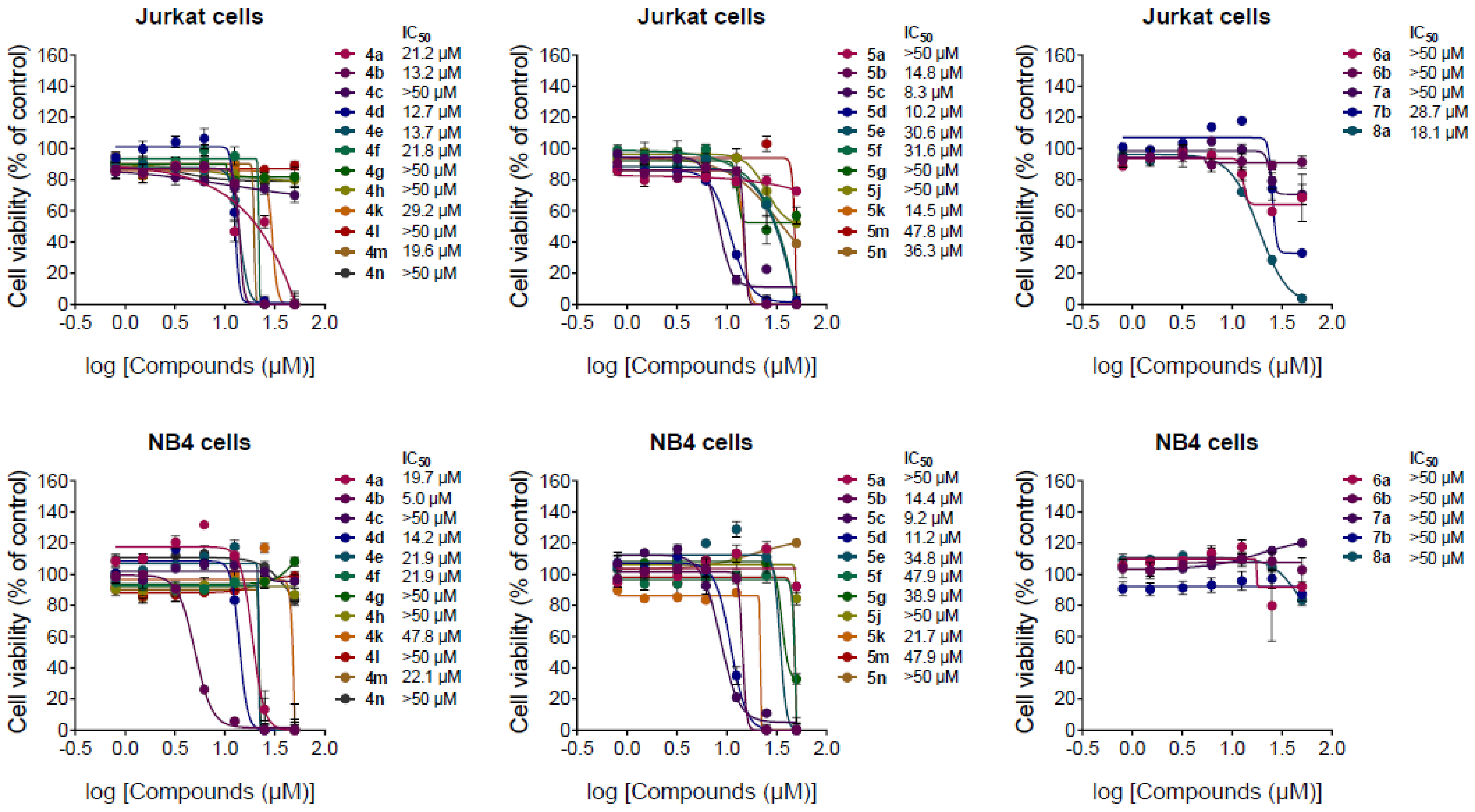

Figure S86 – Dose-response curves for compound **4(a,b,c,d,e,f,g,h,k,l,m,n)**, **5 (a,b,c,d,e,f,g,j,k,m,n)**, **6 (a,b)**, **7 (a,b) and 8a** (0.8–50 µM) after 72 h of exposure in Jurkat and NB4 cells.

Crystallographic details

Experimental – 5g and 8a

Single crystals of $C_{22}H_{17}NO_2$ **5g** and **8a** were obtained by crystallization from a mixture EtOAc/hexanes. Suitable crystals of these compounds were selected and analysed on a **Bruker APEX-II CCD** diffractometer. The crystals were kept at 119.99K during data collection. Using Olex2,[2] the structure was solved with the SHELXT structure solution program using Intrinsic Phasing and refined with the SHELXL refinement package using Least Squares minimisation.[3]

[2] Dolomanov, O. V.; Bourhis, L. J.; Gildea, R. J.; Howard, J. A. K.; Puschmann, H. *J. Appl. Cryst.* **2009**, *42*, 339.

[3] Sheldrick, G. M.; *SHELXL-2014, Program for Structure Refinement*; University of Göttingen, Göttingen, Germany, 2014; Sheldrick, G. M.; *Acta Crystallogr. C Struct. Chem.* **2015**, *71*, 3.

Crystal structure determination of 5g:

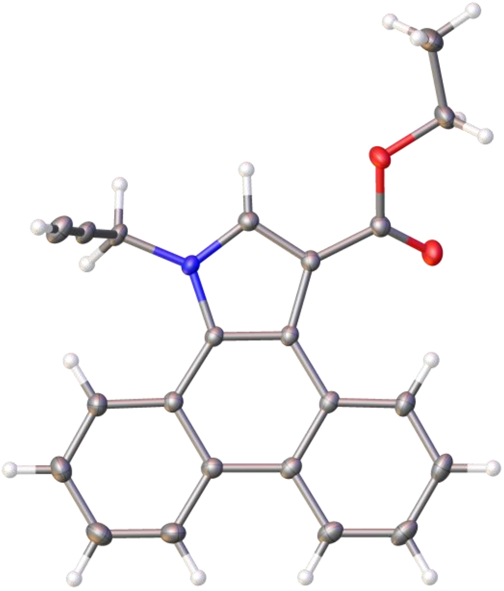

Crystal Data for $C_{22}H_{17}NO_2$ (*M* =327.36 g/mol): triclinic, space group P-1 (no. 2), a = 9.1680(8) Å, b = 9.5785(8) Å, c = 10.7959(9) Å, α = 64.633(2)°, β =73.009(2)°, γ = 84.495(2)°, V = 818.78(12) Å^3, Z = 2, T = 119.99 K, μ(MoKα) = 0.085 mm^{-1}, *Dcalc* = 1.328 g/cm3, 17023 reflections measured (4.648° ≤ 2Θ ≤ 58.254°),4399 unique (*R*int = 0.0207, Rsigma = 0.0191) which were used in all calculations. The final *R*1 was 0.0393 (I > 2σ(I)) and *wR*2 was 0.1119 (all data).

Table 1. Crystal data and structure refinement for 5g.

**CCDC number**	2483262
**Empirical formula**	$C_{22}H_{17}N_2O_2$
**Formula weight**	327.36
**Temperature/K**	119.99
**Crystal system**	triclinic
**Space group**	P-1
**a/Å**	9.1680(8)
**b/Å**	9.5785(8)
**c/Å**	10.7959(9)
**α/°**	64.63(2)

**β/°**	73.009(2)
**γ/°**	84.495(2)
**Volume/Å³**	818.78(12)
**Z**	2
**ρcalcg/cm³**	1.328
**μ/mm⁻¹**	0.085
**F(000)**	344
**Crystal size/mm³**	0.234 × 0.116 × 0.105
**Radiation**	MoKα (λ = 0.71073)
**2Θ range for data collection/°**	4.648 to 58.254
**Index ranges**	-11 ≤ h ≤12, -13 ≤ k ≤13, -14 ≤ l ≤16
**Reflections collected**	17023
**Independent reflections**	4399 [R_{int} = 0.0207, R_{sigma} = 0.0191]
**Data/restraints/parameters**	4399 / 0 / 227
**Goodness-of-fit on F^2**	1.047
**Final R indexes [I>=2σ (I)]**	R_1 = 0.0393, wR_2 = 0.1053
**Final R indexes [all data]**	R_1 = 0.0470, wR_2 = 0.1119
**Largest diff. peak/hole/e Å^{-3}**	0.36/-0.29

Crystal structure determination of 8a:

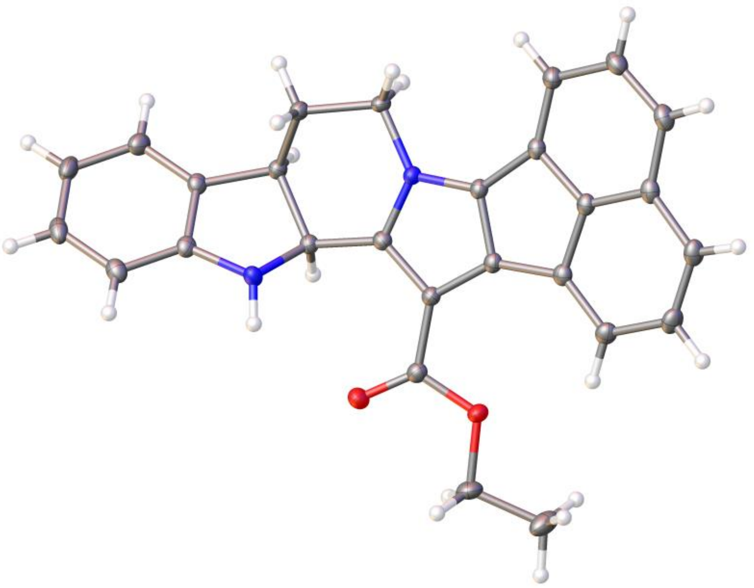

Crystal Data for $C_{27}H_{22}N_2O_2$ (M =406.46 g/mol): triclinic, space group P-1 (no. 2), a = 8.3455(10) Å, b = 10.5930(13) Å, c = 12.2884(15) Å, α = 74.858(3)°, β = 70.412(3)°,γ = 79.700(3)°, V = 983.0(2) Å^3, Z = 2, T = 120.00 K, μ(MoKα) = 0.087 mm^{-1}, Dcalc = 1.373 g/cm3, 18349 reflections measured (3.604° ≤ 2Θ ≤ 56.552°), 4876 unique (Rint = 0.0261,Rsigma = 0.0247) which were used in all calculations. The final R1 was 0.0405 (I > 2σ(I)) and wR2 was 0.1342 (all data).

Table 2. Crystal data and structure refinement for 8a.

**CCDC number**	2483254
**Empirical formula**	$C_{27}H_{22}N_2O_2$
**Formula weight**	406.46
**Temperature/K**	120.00
**Crystal system**	triclinic
**Space group**	P-1
**a/Å**	8.3455(10)
**b/Å**	10.5930(13)
**c/Å**	12.2884(15)
**α/°**	74.858(3)
**β/°**	70.412(3)

**γ/°**	79.700(3)
**Volume/Å3**	983.0(2)
**Z**	2
**ρcalc g/cm3**	1.373
**μ/mm-1**	0.087
**F(000)**	428.0
**Crystal size/mm3**	0.364 × 0.167 × 0.11
**Radiation**	MoKα (λ = 0.71073)
**2Θ range for data collection/°**	3.604 to 56.552
**Index ranges**	-11 ≤ h ≤ 11, -14 ≤ k ≤ 14, -16 ≤ l ≤ 16
**Reflections collected**	18349
**Independent reflections**	4876 [Rint = 0.0261, Rsigma = 0.0247]
**Data/restraints/parameters**	4876/0/282
**Goodness-of-fit on F2**	1.026
**Final R indexes [I>=2σ(I)]**	R1 = 0.0405, wR2 = 0.1232
**Final R indexes [all data]**	R1 = 0.0519, wR2 = 0.1342
**Largest diff. peak/hole / e Å-3**	0.40/-0.24
